# Supplementary material for: Dual sequence definition increases the data storage capacity of sequence-defined macromolecules
Source: Commun Chem. 2020 May 20;3:63. doi: 10.1038/s42004-020-0308-z (PMC9814518; doi:10.1038/s42004-020-0308-z)
Supplement: Supplementary file 1 — Supplementary Information [file 42004_2020_308_MOESM1_ESM.pdf]

Supplementary Information

Dual sequence definition increases the data storage capacity of sequence-defined macromolecules

Katharina S. Wetzel[1‡], Maximiliane Frölich[1‡], Susanne C. Solleder[1], Roman Nickisch[1], Philipp Treu[1], Michael A. R. Meier[1, 2, *]

[1] Laboratory of Applied Chemistry, Institute of Organic Chemistry (IOC), Karlsruhe Institute of Technology (KIT), Straße am Forum 7, 76131 Karlsruhe, Germany.

[2] Laboratory of Applied Chemistry, Institute of Biological and Chemical Systems – Functional Molecular Systems (IBCS-FMS), Karlsruhe Institute of Technology (KIT), Hermann-von-Helmholtz-Platz 1, 76344 Eggenstein-Leopoldshafen, Germany.

[*] Laboratory of Applied Chemistry, Institute of Organic Chemistry (IOC), Karlsruhe Institute of Technology (KIT), Straße am Forum 7, 76131 Karlsruhe, Germany.

Email: m.a.r.meier@kit.edu; Web: www.meier-michael.com

[‡] These authors contributed equally to this work.

Table of contents

1 Supplementary Methods 3

1.1 Materials 3

1.2 Instrumentation 4

1.3 Experimental procedures 6

1.3.1 Monomer syntheses 6

1.3.1.1 Synthesis of monomer M1 6

1.3.1.2 Synthesis of monomer M2 12

1.3.1.3 Synthesis of monomer M3 16

1.3.1.4 Evaluation of the reactivity of monomer M3 22

1.3.1.5 Synthesis of monomer M4 25

1.3.1.6 Synthesis of monomer M5 30

1.3.1.7 Evaluation of the reactivity of monomer M5 36

1.3.1.8 Synthesis of monomer M6 40

1.3.1.9 Evaluation of the reactivity of monomer M6 46

1.3.1.10 Synthesis of monomer M7 50

1.3.1.11 Evaluation of the reactivity of monomer M7 56

1.3.1.12 Synthesis of monomer M8 60

1.3.1.13 Synthesis of monomer M9 66

1.3.1.14 Evaluation of the reactivity of monomer M9 72

1.3.2 Summary of the monomer syntheses 76

1.3.3 Overview of the established library of selectable monomers and aldehyde components 77

1.3.4 Oligomer synthesis 78

1.3.4.1 Backbone variation 78

1.3.4.2 Summary of the synthesis of the backbone defined oligomer – SEC and ESI-MS characterisation 106

1.3.4.3 Dual sequence-definition 108

1.3.4.4 Summary of the synthesis of the dual sequence-defined oligomer – SEC and ESI-MS characterisation 128

1.3.5 Sequential read-out by ESI-MS/MS 129

1.3.5.1 Different types of fragmentation 129

1.3.5.2 Sequencing rules 130
1.3.5.2.1 Calculation of the mass of the molecule 130
1.3.5.2.2 Fragmentation 130
1.3.5.2.3 Calculation example for the dual sequence-defined pentamer DS5 131
1.3.5.3 Side chain defined pentamer S5 133
1.3.5.4 Side chain defined decamer S10 134
1.3.5.5 Backbone defined pentamer B5 135
1.3.5.6 Backbone defined heptamer B7 136
1.3.5.7 Dual sequence-defined pentamer DS5 137
1.3.5.7.1 Fragments with start and end block 137
1.3.5.7.2 Middle fragments (without start and end block) 138
1.3.5.7.3 Fragmentation next to the ester 139
1.4 Supplementary References 140

1 Supplementary Methods

1.1 Materials

The following chemicals were used as received from the following suppliers unless otherwise noted: stearic acid **1** (98%, sigma aldrich), isobutyraldehyde **2a** (98%, Sigma-Aldrich), isovaleraldehyde **2d** (≥ 98%, VWR), dodecanal **2g** (≥ 95%, VWR), octanal **2h** (99%, Sigma-Aldrich), 2-phenylpropanal **2j** (98%, Fisher Scientific), 11-aminoundecanoic acid **3a** (97%, Sigma-Aldrich), 6-aminohexanoic acid **3b** (≥ 99%, VWR), 12-aminododecanoic acid **3c** (98%, ChemPur), β-alanine **3d** (99%, Sigma-Aldrich), 4-aminobutyric acid **3e** (≥ 99%, Sigma-Aldrich), 4-(4-aminophenyl)butanoic acid **3f** (≥ 95%, ChemPur), 3-(4-aminophenyl)propanoic acid **3g** (97%, ChemPur), 3-aminophenyl acetic acid **3h** (97%, Sigma-Aldrich), 4-(aminomethyl)benzoic acid **3i** (97%, Sigma-Aldrich), benzyl alcohol **4** (99%, Sigma-Aldrich), thionyl chloride **5** (99%, Sigma-Aldrich), trimethyl orthoformate **7** (99%, Sigma-Aldrich), phosphoryl trichloride **9** (99%, Sigma-Aldrich), diisopropylamine **12** (> 99.5%, Sigma-Aldrich), palladium on activated charcoal **13** (10% palladium basis, Sigma-Aldrich), hydrogen (99,999%, Air Liquide), 1,8-diazabicyclo[5.4.0]undec-7-ene **14** (DBU, 98%, Sigma-Aldrich), TLC silica gel F_{254} (Sigma-Aldrich), Silica gel 60 (0.040 - 0.063, Sigma-Aldrich and Rocc), cerium(IV)-sulfate (99%, Sigma-Aldrich), phosphomolybdic acid hydrate (99%, Sigma-Aldrich), sodium carbonate (98%, Sigma-Aldrich), sodium

hydrogen carbonate (> 95%, Sigma-Aldrich), sodium sulfate (> 99%, anhydrous, Sigma-Aldrich), magnesium sulfate (≥ 99%, Carl Roth), DMSO-d6 (≥ 99.8%, Euriso-top), MeOH-d4 (≥ 99.8%, Euriso-top), CDCl3 (≥ 99.8%, Euriso-top), dichloromethane (DCM, HPLC grade ≥ 99.9%, Sigma-Aldrich), methanol (HPLC grade 99.8%, Acros Organics), tetrahydrofuran (THF, 99.5%, extra dry over molecular sieves, Acros Organics), ethanol (analytical reagent grade, Fisher Scientific), diethyl ether (analytical reagent grade, Fisher Scientific), cyclohexane (technical grade), ethyl acetate (technical grade). All solvents were used without further purification, unless otherwise noted. Water, when used in the synthesis, was de-ionised.

1.2 Instrumentation

NMR ^{1}H spectra were recorded on a Bruker Avance 300 NMR instrument at 300 MHz for ^{1}H NMR and 75 MHz for ^{13}C NMR, on a Bruker Avance 400 NMR instrument at 400 MHz for ^{1}H NMR and 101 MHz for ^{13}C NMR or on a Bruker AVANCE DRX at 500 MHz for ^{1}H NMR and 125 MHz for ^{13}C-NMR. $CDCl_3$, DMSO-d_6 or CD_3OD were used as solvents. Chemical shifts are presented in parts per million (δ) relative to the resonance signal at 7.26 ppm (^{1}H, $CDCl_3$) and 77.16 ppm (^{13}C, $CDCl_3$), 2.50 ppm (^{1}H, DMSO-d_6) and 39.51 ppm (^{13}C, DMSO-d_6), or 3.31 ppm (^{1}H, CD_3OD) and 49.00 ppm (^{13}C, CD_3OD), respectively. Coupling constants (J) are reported in Hertz (Hz). All measurements were recorded in a standard fashion at 25 °C unless otherwise stated. Full assignment of structures was aided by 2D NMR analysis (COSY, HSQC and HMBC).

Size Exclusion Chromatography (SEC) measurements were performed on a SHIMADZU Size Exclusion Chromatography (SEC) system equipped with a SHIMADZU isocratic pump (LC-20AD), a SHIMADZU refractive index detector (24°C) (RID-20A), a SHIMADZU autosampler (SIL-20A) and a VARIAN column oven (510, 50°C). For separation, a three-column setup was used with one SDV 3 µm, 8×50 mm precolumn and two SDV 3 µm, 1000 Å, 3×300 mm columns supplied by PSS, Germany. Tetrahydrofuran (THF) stabilized with 250 ppm butylated hydroxytoluene (BHT, ≥99.9%) supplied by SIGMA-ALDRICH was used at a flow rate of 1.0 $mL\cdot min^{-1}$. Calibration was carried out by injection of eight poly(methylmethacrylate) standards ranging from 102 to 58300 kDa.

SEC-ESI-MS spectra were recorded on a Q Exactive (Orbitrap) mass spectrometer (Thermo Fisher Scientific, San Jose, CA, USA) equipped with a HESI II probe. The instrument was calibrated in the *m/z* range 74–1822 using premixed calibration solutions (Thermo Scientific). A constant spray voltage of 4.6 kV, a dimensionless sheath gas of 8, and a dimensionless auxiliary gas flow rate of 2 were applied. The capillary temperature and the S-lens RF level were set to 320 °C and 62.0, respectively. The Q Exactive was coupled to an UltiMate 3000 UHPLC System (Dionex, Sunnyvale, CA, USA) consisting of

a pump (LPG 3400SD), autosampler (WPS 3000TSL), and a thermostated column department (TCC 3000SD). Separation was performed on two mixed bed size exclusion chromatography columns (Polymer Laboratories, Mesopore 250 × 4.6 mm, particle diameter 3 µm) with precolumn (Mesopore 50 × 4.6 mm) operating at 30 °C. THF at a flow rate of 0.30 mL·min^{-1} was used as eluent. The mass spectrometer was coupled to the column in parallel to a RI-detector (RefractoMax520, ERC, Japan). 0.27 mL·min^{-1} of the eluent were directed through the RI-detector and 30 µL·min^{-1} infused into the electrospray source after post-column addition of a 100 µM solution of sodium iodide in methanol at 20 µL·min^{-1} by a micro-flow HPLC syringe pump (Teledyne ISCO, Model 100DM). A 20 µL aliquot of a polymer solution with a concentration of 2 mg·mL^{-1} was injected onto the HPLC system.

Orbitrap Electrospray-Ionisation Mass Spectrometry (ESI-MS) mass spectra were recorded on a Q Excative (Orbitrap) mass spectrometer (Thermo Fisher Scientific, San Jose, CA, USA) equipped with an atmospheric pressure ionisation source operating in the nebuliser assisted electrospray mode. The instrument was calibrated in the m/z-range 150-2000 using a standard mixture containing caffeine, Met-Arg-Phe-Ala acetate (MRFA) and a mixture of fluorinated phosphazenes (Ultramark 1621, all from Sigma Aldrich). A constant spray voltage of 3.5 kV, a dimensionless sheath gas of 6, and a sweep gas flow rate of 2 were applied. The capillary voltage and the S-lens RF level were set to 68.0 V and 320°C, respectively.

MS/MS experiments were performed with a collision energy value between NCE 15 and 20. Nitrogen was used as a collision gas. The measurements were performed using an acquire time of 10 scans.

Electron ionisation (EI) For the measurements that were performed with the electron ionisation (EI) method, an instrument by Finnigan, model MAT 90 (70 eV), was used with 3-nitrobenzyl alcohol (3-NBA) as matrix. For the interpretation of the spectra, molecular peaks $[M]^+$, peaks of pseudo molecular ions $[M+H]^+$ and characteristic fragment peaks are indicated with their mass to charge ratio (*m/z*) and their intensity in percent, relative to the most intense peak (100%).

Fast atom bombardment (FAB) mass spectra were recorded on a *Finnigan* MAT 95 instrument. The protonated molecular ion is expressed by the term: $[(M+H)]^+$.

Infrared spectra (IR) were recorded on a Bruker Alpha-p instrument in a frequency range from 3998 to 374 cm^{-1} applying KBr and Attenuated Total Reflection (ATR) technology.

All **thin layer chromatography** experiments were performed on silica gel coated aluminium foil (silica gel 60 F_{254}, Sigma-Aldrich). Compounds were visualized by staining with Seebach-solution (mixture of phosphomolybdic acid hydrate, cerium(IV)-sulfate, sulfuric acid and water).

1.3 Experimental procedures

1.3.1 Monomer syntheses

1.3.1.1 Synthesis of monomer M1

M1 was synthesised according to a previously reported procedure:[1]

Esterification

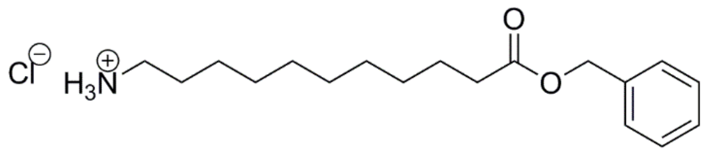

*Supplementary Figure 1. Chemical structure of compound **6a**.*

11-Aminoundecanoic acid **3a** (15.0 g, 74.8 mmol, 1.00 eq.) was suspended in 75 mL THF. Subsequently, benzyl alcohol **4** (93.4 mL, 97.1 g, 898 mmol, 12.0 eq.) was added and the suspension was cooled to 0 °C. Thionyl chloride **5** (16.6 mL, 27.4 g, 232 mmol, 3.10 eq.) was added dropwise. After addition of the thionyl chloride **5**, the solution was warmed to room temperature and stirred overnight. The yellow solution was then poured into 500 mL of diethyl ether and stored in the freezer for one hour. The product was filtered off and another 500 mL diethyl ether were added. Then, the suspension was stored in the freezer for another hour. The product was filtered off and dried under high vacuum. 11-(Benzyloxy)-11-oxoundecan-1-amonium chloride **6a** was obtained in a yield of 95% (20.7 g, 71.1 mmol) as a white solid.

^{1}H-NMR: (300 MHz, CD_3OD) δ /ppm: 7.33 (s, 5H, CH aromatic, [1]); 5.09 (s, 2H, CH_2, [2]); 2,90 (t, *J* = 7.5 Hz, 2H, CH_2, [3]); 2.34 (t, *J* = 7,3 Hz, 2H, CH_2, [4]); 1.77 – 1.48 (m, 4H, 2 CH_2, [5]); 1.31 (m, 12H, 6 CH_2, [6]).

^{13}C NMR (75 MHz, CD_3OD) δ /ppm: 175.2, 137.7, 129.5, 129.5, 129.2, 129.2, 67.1, 40.8, 35.0, 30.4, 30.3, 30.2, 30.1, 28.5, 27.4, 26.0.

HRMS-FAB-MS of $[C_{18}H_{30}NO_2]^+$: calculated: 292.2271, found: 292.2271.

IR (ATR platinum diamond): ν /cm^{-1} = 2916.5, 2847.7, 1737.2, 1601.6, 1527.7, 1495.8, 1462.9, 1385.9, 1359.8, 1332.2, 1307.4, 1278.9, 1246.1, 1208.4, 1152.0, 1043.3, 992.7, 959.9, 810.1, 742.7, 722.1, 695.3, 580.3, 509.3, 416.3.

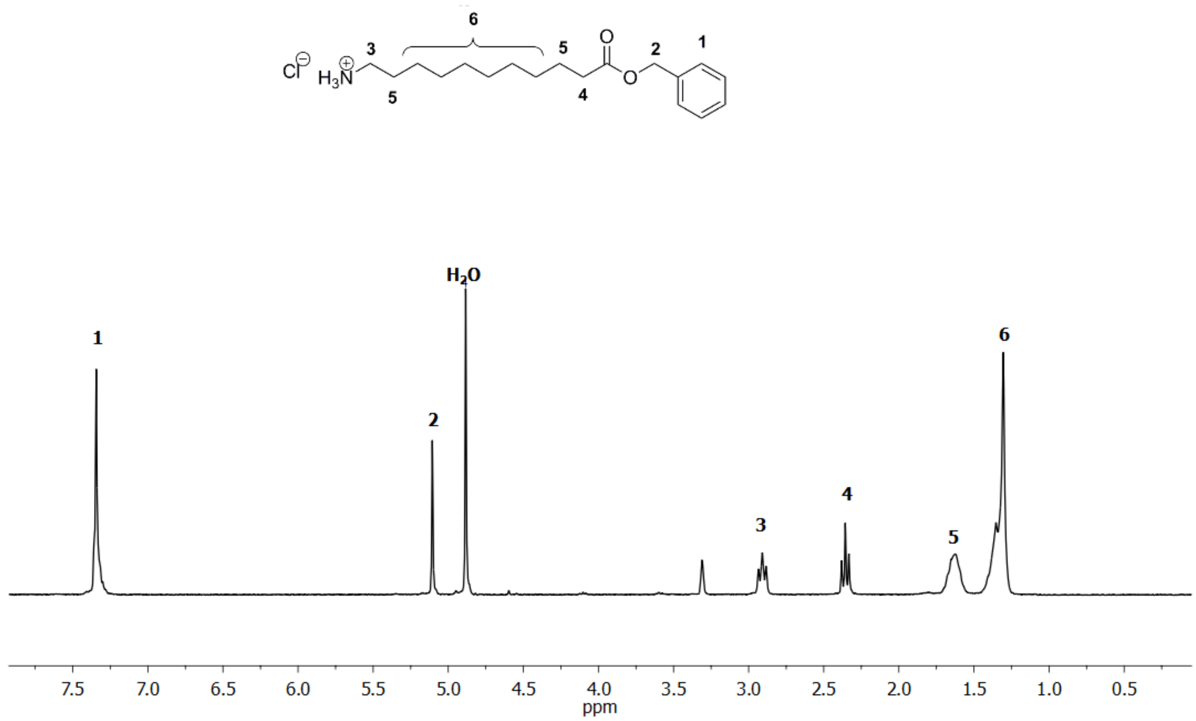

*Supplementary Figure 2. Proton NMR of compound **6a** measured in CD_3OD.*

N-Formylation

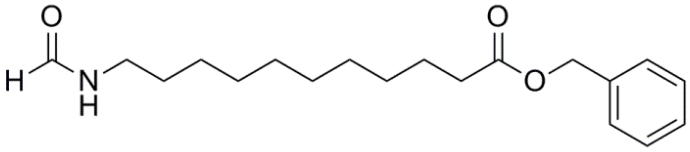

*Supplementary Figure 3. Chemical structure of compound **8a**.*

11-(Benzyloxy)-11-oxoundecan-1-amonium chloride **6a** (21.3 g, 64.7 mmol, 1.00 eq.) was dissolved in trimethyl orthoformate **7** (70.7 mL, 68.7 g, 647 mmol, 10.0 eq.) and heated to 100 °C. The reaction mixture was refluxed overnight at 100 °C. Trimethyl orthoformate **7** was then removed under reduced pressure and the crude product **8a** (21.9 g) was used without further purification.

^{1}H NMR (300 MHz, $CDCl_3$) δ /ppm: 8.22 – 7.97 (m, 1H, CH, [1]), 7.44 – 7.18 (m, 5H, CH aromatic, [2]), 5.61 (s, 1H, NH, [3]), 5.10 (s, 2H, CH_2, [4]), 3.42 – 3.11 (m, 2H, CH_2, [5]), 2.41 – 2.28 (t, *J* = 7.5 Hz, 2H, CH_2, [6]), 1.91 – 1.41 (m, 4H, 2CH_2, [7]), 1.40 – 1.09 (m, 12H, 6 CH_2, [8]).

^{13}C NMR (75 MHz, $CDCl_3$) δ /ppm: 173.8, 164.7, 161.3, 136.2, 128.6, 128.2, 66.1, 41.8, 38.2, 34.4, 31.3, 29.5, 29.4, 29.3, 29.2, 29.1, 26.9, 26.4, 25.0.

HRMS-FAB-MS of $[C_{19}H_{30}NO_3]^+$: calculated: 320.2220, found: 320.2222.

IR (ATR platinum diamond): ν / cm^{-1} = 3264.9, 3068.1, 2913.6, 2848.1, 1732.5, 1651.1, 1555.4, 1470.5, 1449.3, 1417.1, 1379.2, 1329.5, 1299.2, 1267.1, 1233.0, 1199.5, 1159.1, 1054.8, 996.7, 938.2, 866.1, 825.1, 806.2, 752.8, 718.3, 695.3, 608.9, 519.5, 487.1, 451.5.

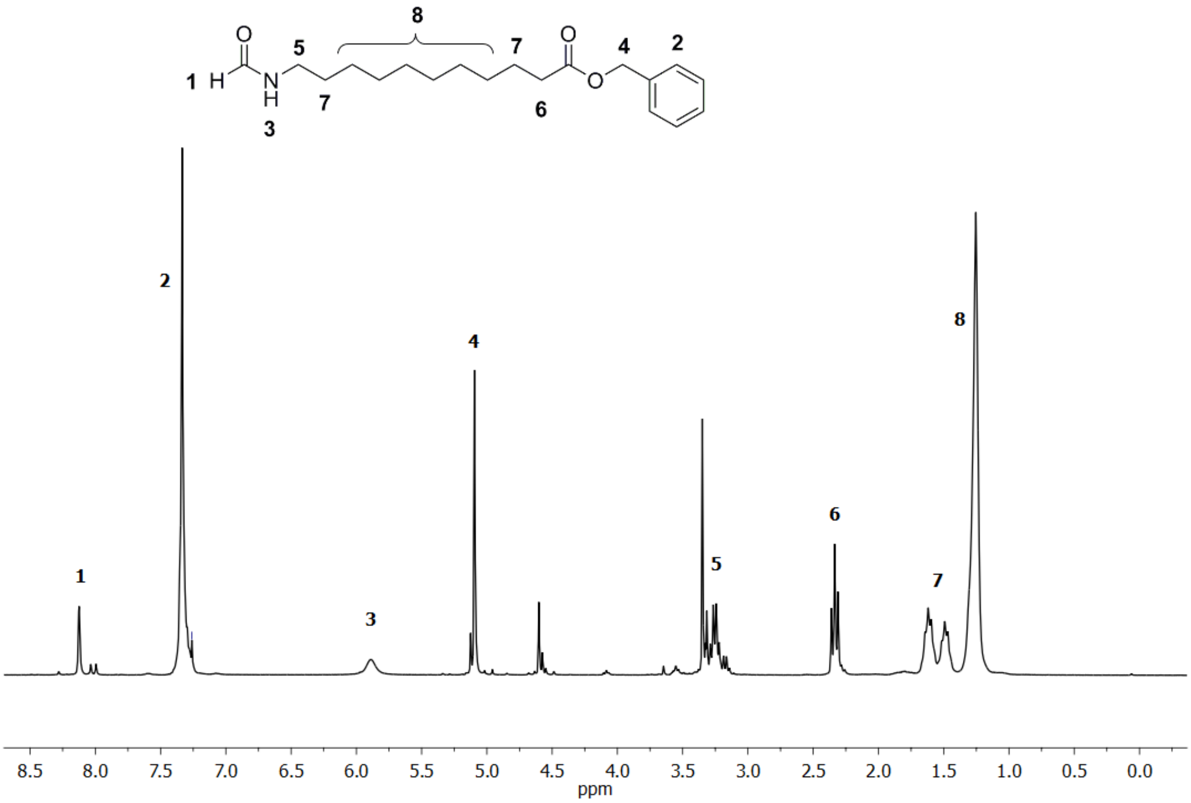

*Supplementary Figure 4. Proton NMR of compound **8a** measured in $CDCl_3$.*

Dehydration

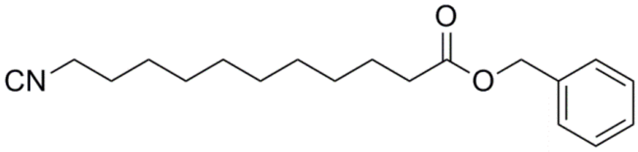

Supplementary Figure 5. Chemical structure of compound ***M1****.*

Benzyl-11-formamidoundecanoate **8a** (19.6 g, 61.2 mmol, 1.00 eq.) was dissolved in 185 mL DCM (0.33 M). Diisopropylamine **12** (26.7 mL, 19.2 g, 0.190 mol, 3.00 eq.) was added and the reaction mixture was cooled to 0 °C in an ice bath. Subsequently, phosphoryl trichloride **9** (6.89 mL, 11.3 g, 73.5 mmol, 1.20 eq.) was added dropwise and the reaction mixture was then stirred at room temperature for two hours. Subsequently, the mixture was cooled in an ice bath and the reaction was quenched by addition of sodium carbonate solution (20%, 75 mL) at 0 °C. After stirring for 30 minutes at room temperature, 50 mL DCM and 50 mL water were added. After separation of the aqueous phase, the organic layer was washed with water (3 × 80 mL) and brine (80 mL). The organic layer was dried over sodium sulfate and the solvent was removed under reduced pressure. The crude product was purified by column chromatography (hexane / ethyl acetate 19:1 → 8:1). The product **M1** was obtained as slightly yellow oil in a yield of 67% (12.3 g, 41.0 mmol).

^{1}H NMR (300 MHz, $CDCl_3$) δ /ppm: 7.40 – 7.28 (m, 5H, CH aromatic, ^{1}), 5.11 (s, 2H, CH_2, ^{2}), 3.47 – 3.25 (m, 2H, CH_2, ^{3}), 2.35 (t, J = 7.5 Hz, 2H, CH_2, ^{4}), 1.77 – 1.52 (m, 4H, 2 CH_2, ^{5}), 1.51 – 0.99 (m, 12H, 6 CH_2, ^{6}).

^{13}C NMR (75 MHz, $CDCl_3$) δ /ppm: 173.8, 155.8, 155.7, 155.6, 136.2, 128.6, 128.3, 66.2, 41.7, 41.6, 41.6, 34.4, 29.4, 29.3, 29.2, 29.2, 28.8, 26.4, 25.0.

HRMS-FAB-MS of $[C_{19}H_{28}NO_2]^+$: calculated: 302.2115, found: 302.2113.

IR (ATR platinum diamond): ν / cm^{-1} = 3032.1, 2924.6, 2853.5, 2145.5 (isocyanide), 1732.4, 1496.9, 1454.3, 1380.2, 1350.0, 1211.7, 1160.2, 1100.4, 1001.0, 735.3, 696.7, 578.4, 494.9.

***R*$_f$**: (hexane / ethyl acetate 5:1) = 0.55.

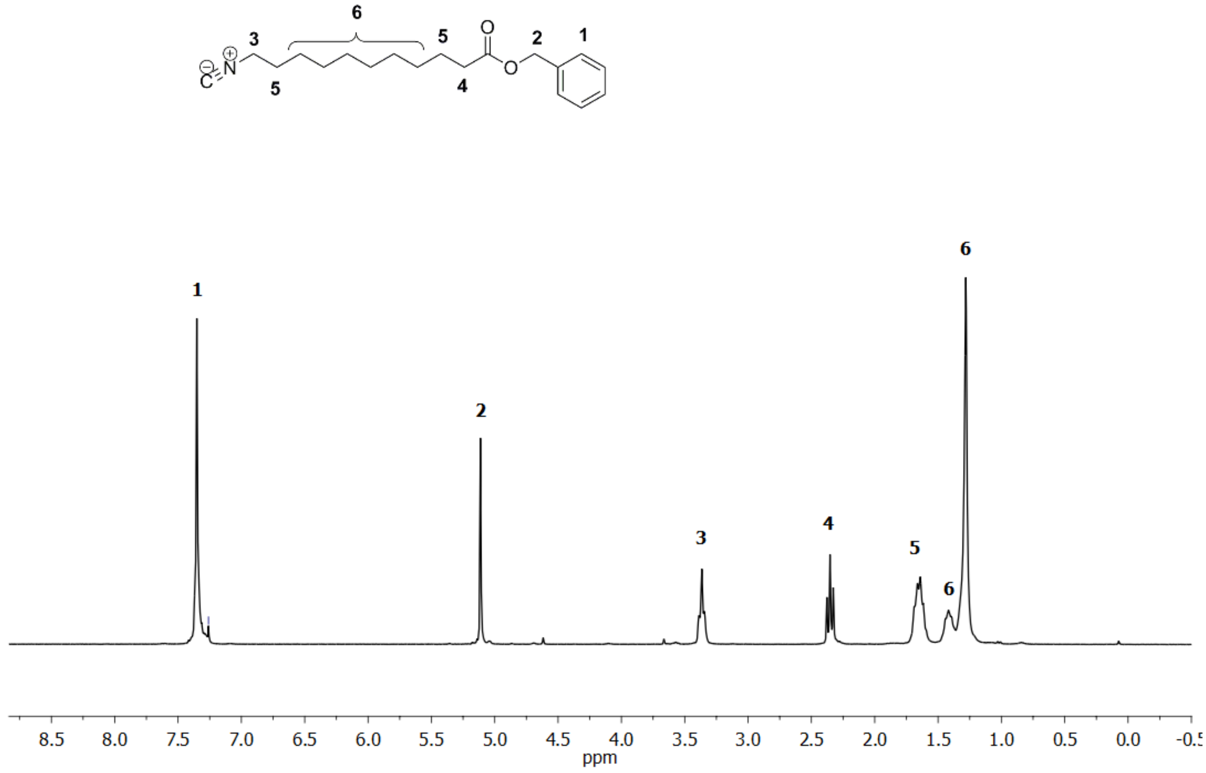

*Supplementary Figure 6. Proton NMR of compound **M1** measured in $CDCl_3$.*

1.3.1.2 Synthesis of monomer M2

Esterification

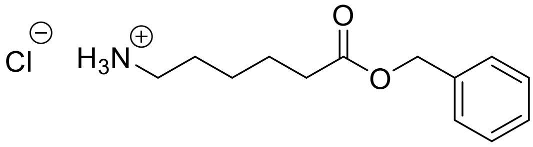

*Supplementary Figure 7. Chemical structure of compound **6b**.*

6-Aminohexanoic acid **3b** (1.98 g, 15.0 mmol, 1.00 eq.) was suspended in 10 mL THF and benzyl alcohol **4** (20.3 mL, 20.9 g, 194 mmol, 12.9 eq.) was added. The suspension was cooled in an ice bath and subsequently thionyl chloride **5** (3.37 mL, 5.53 g, 46.5 mmol, 3.10 eq.) was added dropwise at 0 °C. After addition of the thionyl chloride **5**, the solution was warmed to room temperature and stirred overnight. The yellow solution was then poured into 200 mL diethyl ether and stored in the freezer for one hour. The product was then filtered off and dried under high vacuum. 6-(Benzyloxy)-6-oxohexane-1-ammoniumchloride **6b** was obtained as a white solid in a yield of 96% (3.71 g, 14.4 mmol).

^{1}H-NMR (300 MHz, CD_3OD) δ/ppm: 7.51 – 7.13 (m, 5H, CH aromatic, ^{1}), 5.11 (s, 2H, CH_2, ^{2}), 2.90 (t, J = 7.6 Hz, 2H, CH_2, ^{3}), 2.41 (t, J = 7.3 Hz, 2H, CH_2, ^{4}), 1.84 – 1.54 (m, 4H, CH_2, ^{5}), 1.54 – 1.25 (m, 2H, CH_2, ^{6}).

^{13}C-NMR (75 MHz, CD_3OD) δ/ppm: 174.8, 137.7, 129.5, 129.2, 67.2, 40.5, 34.6, 28.2, 26.8, 25.4.

HRMS FAB-MS of $[C_{13}H_{20}NO_2]^+$: calculated: 222.1489, found: 222.1489.

IR (ATR platinum diamond): ν/cm^{-1} = 3383.3, 3031.0, 2940.1, 1731.7, 1605.1, 1497.1, 1467.6, 1454.4, 1387.4, 1356.4, 1311.1, 1248.2, 1214.9, 1166.3, 1143.5, 1045.2, 1013.3, 964.0, 937.9, 827.1, 748.1, 695.7, 578.8, 520.4, 474.2.

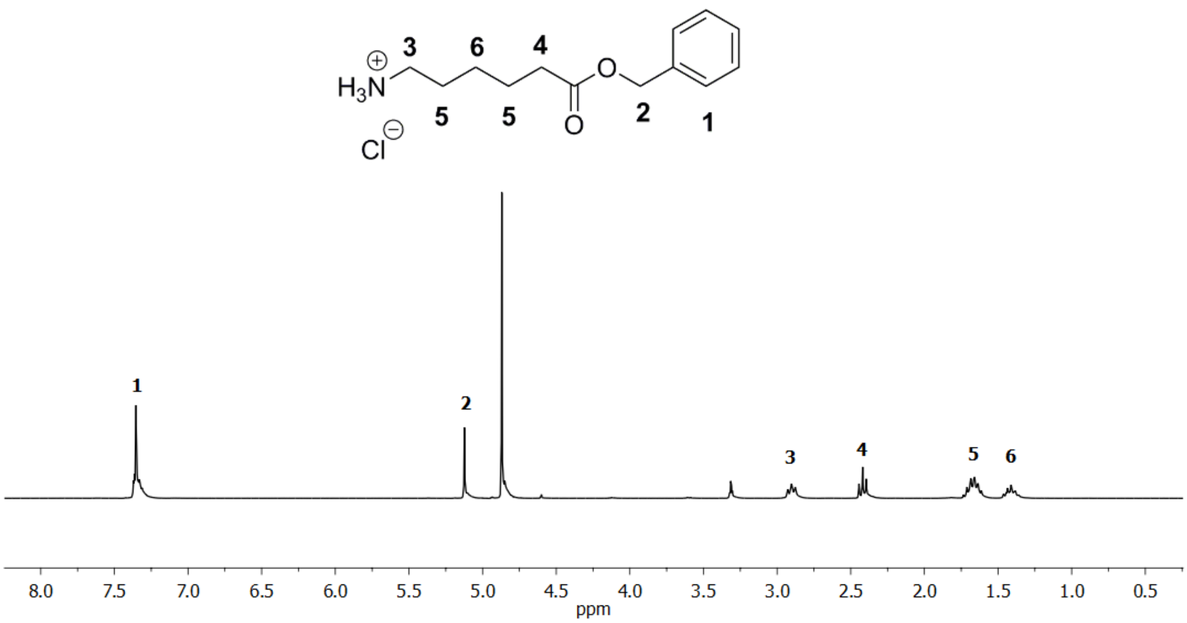

*Supplementary Figure 8. Proton NMR of compound **6b** measured in CD_3OD.*

N-Formylation

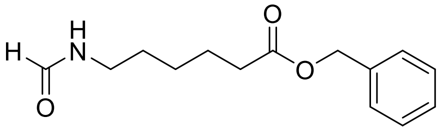

*Supplementary Figure 9. Chemical structure of compound **8b**.*

6-(Benzyloxy)-6-oxohexane-1-ammoniumchloride **6b** (3.29 g, 12.7 mmol, 1.00 eq.) was dissolved in trimethyl orthoformate **7** (14.1 mL, 13.6 g, 127 mmol, 10.1 eq.) and stirred for 24 hours at 100 °C. The excess of trimethyl orthoformate **7** was removed under reduced pressure and the product was purified by column chromatography (hexane/ethyl acetate 3:1 → 0:1). The yellowish liquid **8b** was obtained in a yield of 73% (2.31 g, 9.27 mmol).

^{1}H-NMR (300 MHz, $CDCl_3$) δ/ppm: 8.13 (s, 1H, CH, [1]), 7.45 – 7.27 (m, 5H, CH aromatic, [2]), 5.59 (s, 1H, NH, [3]), 5.11 (s, 2H, CH_2, [4]), 3.41 – 3.06 (m, 2H, CH_2, [5]), 2.37 (t, J = 7.3 Hz, 2H, CH_2, [6]), 1.78 – 1.59 (m, 2H, CH_2, [7]), 1.59 – 1.44 (m, 2H, CH_2, [7]), 1.42 – 1.24 (m, 2H, CH_2, [7]).

^{13}C-NMR (75 MHz, $CDCl_3$) δ/ppm: 173.4, 164.7, 161.3, 136.1, 128.6, 128.3, 66.2, 41.5, 37.9, 34.1, 30.9, 29.1, 26.3, 25.9, 24.4, 24.4.

HRMS-FAB-MS of $[C_{14}H_{20}NO_3]^+$: calculated: 250.1438, found: 250.1437.

IR (ATR platinum diamond): ν/cm^{-1} = 3291.8, 3032.8, 2934.5, 2859.8, 1730.0, 1658.2, 1528.3, 1454.5, 1382.6, 1213.2, 1154.0, 1100.2, 1000.9, 736.8, 697.2, 497.6.

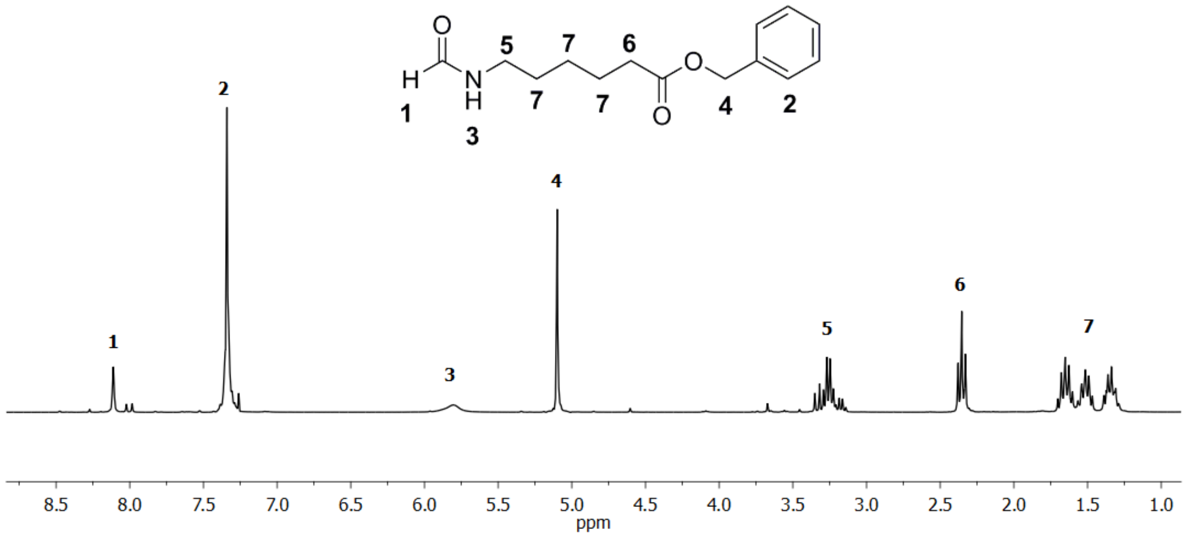

*Supplementary Figure 10. Proton NMR of compound **8b** measured in $CDCl_3$.*

Dehydration

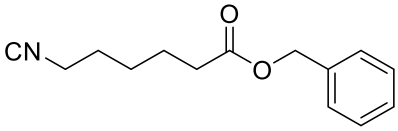

*Supplementary Figure 11. Chemical structure of compound **M2**.*

Benzyl-6-formamidohexanoate **8b** (1.70 g, 6.84 mmol, 1.00 eq.) was dissolved in 20 mL DCM (0.34 M), diisopropylamine **12** (2.98 mL, 2.15 g, 21.2 mmol, 3.10 eq.) was added and the reaction mixture was cooled to 0 °C. Subsequently, phosphoryl trichloride **9** (0.829 mL, 1.36 g, 8.89 mmol, 1.30 eq.) was added dropwise and the reaction mixture was then stirred at room temperature for two hours. The reaction was quenched by addition of a 20% solution of sodium carbonate (9.0 mL) at 0 °C. After stirring this mixture for 30 minutes, 20 mL water and 20 mL DCM were added. The aqueous phase was separated and the organic layer was washed with water (3 x 20 mL) and brine (20 mL). The combined organic layers were dried over sodium sulfate and the solvent was evaporated under reduced pressure. The crude product was then purified by column chromatography (hexane/ethyl acetate 9:1 →3:1). The product monomer **M2** was obtained as brown oil in a yield of 74% (1.17 g, 5.06 mmol).

^{1}H-NMR (300 MHz, $CDCl_3$) δ/ppm: 7.50 – 7.29 (m, 5H, CH aromatic, [1]), 5.12 (s, 2H, CH_2, [2]), 3.47 – 3.27 (m, 2H, CH_2, [3]), 2.39 (t, *J* = 7.4 Hz, 2H, CH_2, [4]), 1.80 – 1.58 (m, 4H, CH_2, [5]), 1.55 – 1.36 (m, 2H, CH_2, [6]).

^{13}C-NMR (75 MHz, $CDCl_3$) δ/ppm: 173.1, 156.1, 136.0, 128.6, 128.3, 66.3, 41.4, 34.0, 28.8, 25.9, 24.1.

HRMS-EI-MS of $[C_{14}H_{17}NO_2]^{\cdot+}$: calculated: 231.1254, found: 231.1255.

IR (ATR platinum diamond): ν/cm^{-1} = 3023.3, 2943.9, 2863.6, 2146.5, 1730.2, 1496.6, 1454.1, 1382.3, 1351.9, 1257.5, 1152.2, 1093.8, 1001.3, 737.4, 697.6, 578.7, 504.9, 454.9.

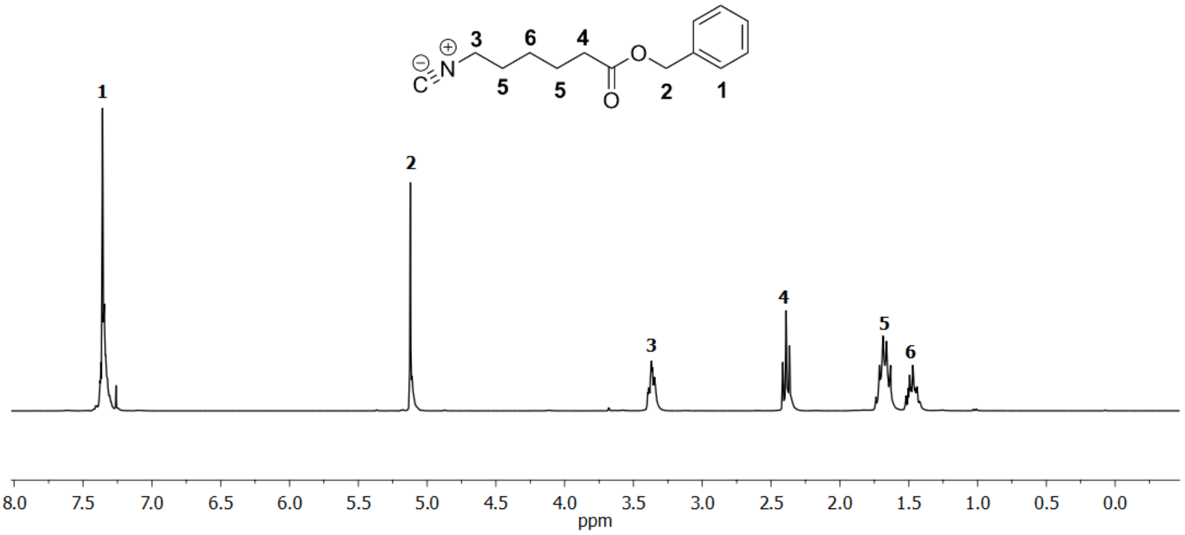

*Supplementary Figure 12. Proton NMR of compound **M2** measured in $CDCl_3$.*

1.3.1.3 Synthesis of monomer M3

Esterification

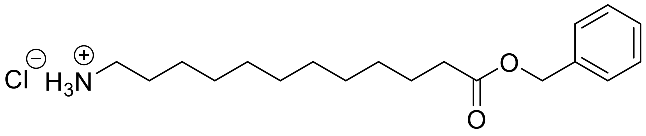

*Supplementary Figure 13. Chemical structure of compound **6c**.*

12-Aminododecanic acid **3c** (13.2 g, 61.5 mmol, 1.00 eq.) was suspended in THF (16 mL). After the addition of benzyl alcohol **4** (95.4 mL, 99.7 g, 922 mmol, 15.0 eq.), the suspension was cooled with an ice bath to 0 °C and thionyl chloride **5** (17.9 mL, 29.2 g, 246 mmol, 4.00 eq.) was added dropwise. Afterwards, the suspension was stirred at room temperature for 24 hours. Subsequently, 500 mL of diethyl ether were added, and the solution was stored in the freezer for 2 hours. The mixture was filtered and 500 mL of diethyl ether were added to the precipitate. The mixture was stored in the freezer for another 2 hours and the precipitate was filtered off and dried under reduced pressure. A 1:1 mixture of the desired ammonium salt **6c** (14.1 g, 46.1 mmol) and the unreacted starting material was obtained as a white solid in a yield of 75%. The mixture was used without purification.

^{1}H-NMR (300 MHz, CD_3OD) δ/ppm: 7.34 – 7.25 (m, 5H, CH aromatic, ^{1}), 5.07 (s, 2H, CH_2, ^{2}), 2.87 (t, J = 7.7 Hz, 2H, CH_2, ^{3}), 2.32 (t, J = 7.3 Hz, 2H, CH_2, ^{4}), 1.68 – 1.51 (m, 4H, CH_2, ^{5}), 1.41 – 1.21 (m, 14H, CH_2, ^{6}).

^{13}C-NMR (126 MHz, CD_3OD) δ/ppm: 175.19, 137.71, 129.51, 129.16, 67.07, 40.77, 35.04, 30.49, 30.47, 30.42, 30.29, 30.17, 30.07, 28.54, 27.43, 26.02.

HRMS-FAB-MS of $[C_{19}H_{32}O_2N]^+$ calculated: 306.2428 found: 306.2431.

IR (ATR platinum diamond): ν/cm^{-1} = 3197.3, 3021.6, 2914.0, 2847.6, 1734.6, 1583.4, 1517.9, 1497.2, 1470.5, 1414.7, 1390.8, 1362.3, 1326.9, 1294.8, 1265.3, 1235.9, 1205.0, 1175.5, 1145.4, 1116.4, 1096.6, 1028.3, 1001.7, 960.7, 929.6, 908.7, 858.2, 824.0, 776.1, 726.2, 694.0, 609.2, 575.5, 507.7, 485.3, 460.2, 434.6.

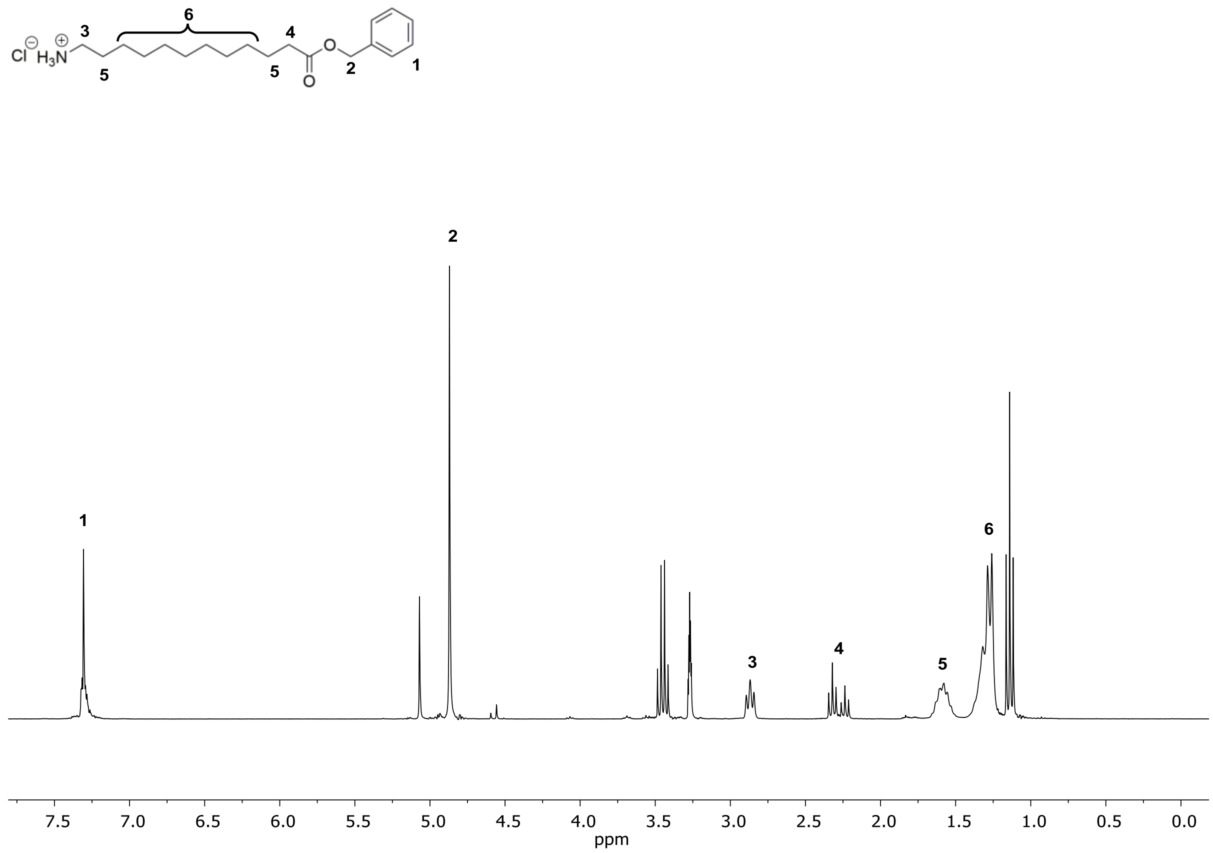

*Supplementary Figure 14. Proton NMR of compound **6c** measured in CD_3OD.*

N-Formylation

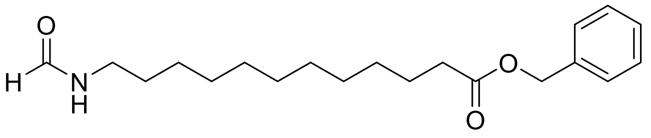

*Supplementary Figure 15. Chemical structure of compound **8c**.*

The ammonium salt **6c** (13.5 g, 33.4 mmol, 1.00 eq.) was stirred with trimethyl orthoformate **7** (48.3 mL, 46.8 g, 441 mmol, 11.2 eq.) under reflux at 105 °C overnight. Subsequently, the orthoformate **7** was evaporated under reduced pressure. The crude product **8c** (12.4 g, 37.2 mmol) was obtained as a yellow oil in a quantitative yield and was used without further purification.

^{1}H-NMR (300 MHz, $CDCl_3$) δ/ppm: 8.17 – 7.96 (m, 1H, CH, [1]), 7.41 – 7.21 (m, 5H, CH aromatic, [2]), 5.09 (s, 2H, CH_2, [3]), 3.79 – 3.09 (m, 4H,CH_2, [4]), 2.38 – 2.30 (m, 2H, CH_2, [5]), 1.70 – 1.56 (m, 2H, CH_2, [6]), 1.39 – 1.13 (m, 14H, CH_2, [7]).

^{13}C-NMR (126 MHz, $CDCl_3$) δ/ppm: 173.77, 161.34, 136.16, 128.59, 128.21, 128.18, 66.11, 38.24, 34.36, 29.55, 29.50, 29.47, 29.40, 29.25, 29.13, 26.88, 24.98.

HRMS-FAB-MS of $[C_{20}H_{32}O_3N]^+$ calculated: 334.2377 found: 334.2384.

IR (ATR platinum diamond): ν/cm^{-1} = 3284.9, 3198.7, 3033.9, 2914.6, 2848.7, 1734.8, 1654.2, 1548.1, 1518.5, 1497.3, 1470.9, 1416.7, 1381.7, 1326.9, 1289.5, 1262.1, 1233.8, 1202.2, 1158.9, 1097.8, 1073.2, 1027.6, 1001.5, 945.3, 899.3, 869.3, 823.8, 775.7, 749.2, 726.5, 695.6, 608.5, 577.0, 527.3, 488.7, 460.1, 432.7, 407.4.

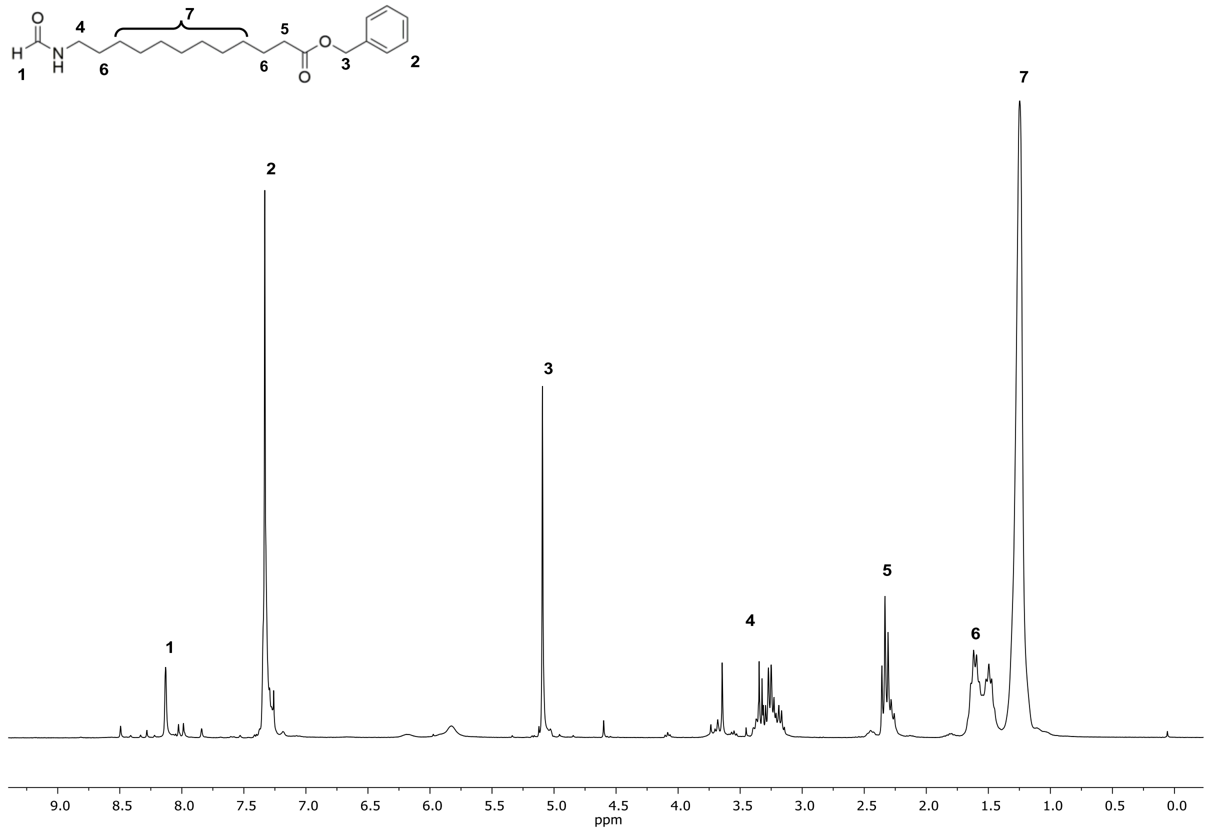

*Supplementary Figure 16. Proton NMR of compound **8c** measured in $CDCl_3$.*

Dehydration

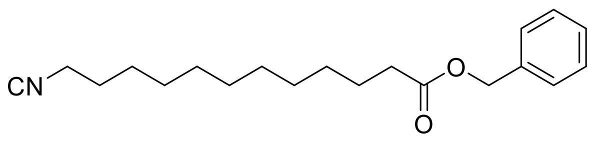

*Supplementary Figure 17. Chemical structure of compound **M3**.*

The formamide **8c** (12.4 g, 37.2 mmol, 1.00 eq.) was dissolved in DCM (115 mL) and diisopropylamine **12** (16.2 mL, 11.7 g, 115 mmol, 3.10 eq.) was added. The solution was cooled to 0 °C with an ice bath. Then, phosphoryl trichloride **9** (5.50 mL, 9.30 g, 55.7 mmol, 1.50 eq.) was added dropwise to the reaction mixture. The yellow solution was stirred for two hours at room temperature and was cooled to 0 °C again. The reaction was quenched by the addition of a sodium carbonate solution (20 wt%, 90 mL) and stirred for another 30 minutes at room temperature. DCM (100 mL) and water (100 mL) were added to the mixture and the organic layer was separated. The organic layer was washed with water (2 × 80 mL) and brine (80 mL), dried over sodium sulfate and the solvent was evaporated under reduced pressure. The crude product was purified by column chromatography (cyclohexane/ethyl acetate 12:1 → 4:1). Monomer **M3** (3.73 g, 11.8 mmol) was obtained as a yellow oil in a yield of 32%.

^{1}H-NMR (300 MHz, $CDCl_3$) *δ*/ppm: 7.41 – 7.30 (m, 5H, CH aromatic, [1]), 5.12 (s, 2H, CH_2, [2]), 3.41 – 3.35 (m, 2H, CH_2, [3]), 2.40 – 2.29 (m, 2H, CH_2, [4]), 1.70 – 1.60 (m, 4H, CH_2, [5]), 1.30 – 1.25 (m, 14H, CH_2, [6]).

^{13}C-NMR (101 MHz, $CDCl_3$) *δ*/ppm: 173.80, 136.23, 128.65, 128.27, 66.17, 41.73, 41.67, 41.61, 34.42, 29.50, 29.45, 29.42, 29.30, 29.20, 28.79, 26.41, 25.03.

HRMS-FAB-MS of $[C_{20}H_{30}O_2N]^+$ calculated: 316.2271 found 316.2272.

IR (ATR platinum diamond): ν /cm^{-1} = 3065.7, 3032.9, 2925.1, 2854.3, 2146.5, 1734.1, 1497.7, 1455.2, 1351.0, 1278.8, 1160.4, 1103.0, 1026.9, 994.8, 735.8, 697.1, 661.7, 554.8, 500.1.

R_f:(cyclohexane / ethyl acetate 5:1) = 0.55.

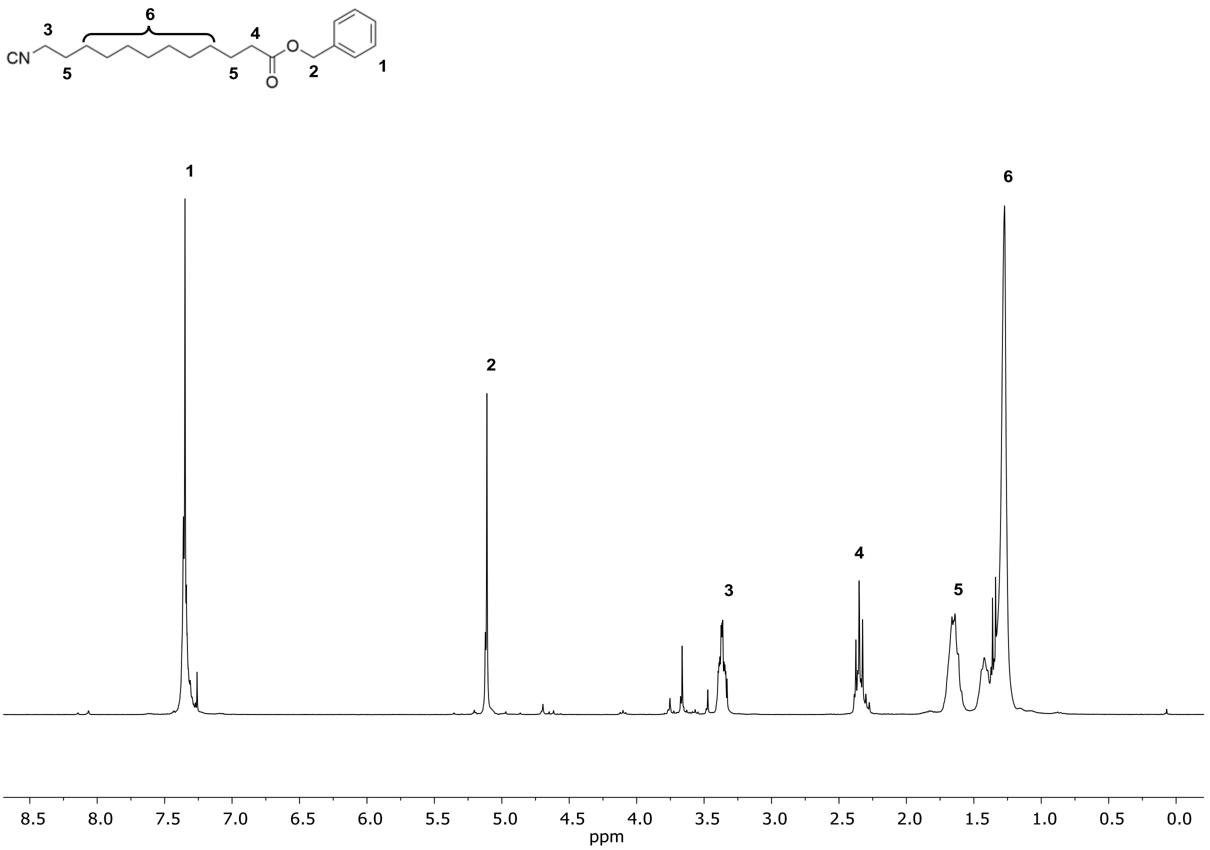

Supplementary Figure 18. Proton NMR of compound ***M3*** *measured in* $CDCl_3$.

1.3.1.4 Evaluation of the reactivity of monomer M3

Passerini reaction

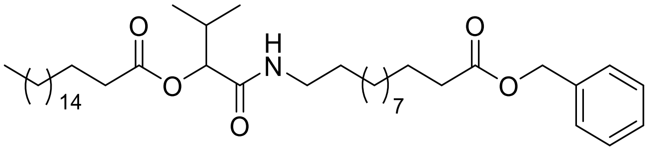

*Supplementary Figure 19. Chemical structure of compound **15**.*

Stearic acid **1** (79.9 mg, 0.281 mmol, 1.00 eq.) was suspended in 0.42 mL DCM. Subsequently, monomer **M3** (133 mg, 0.422 mmol, 1.50 eq.) and isobutyraldehyde **2a** (38.0 µL, 30.4 mg, 0.422 mmol, 1.50 eq.) were added. The colourless reaction mixture was stirred at room temperature for 24 hours. Afterwards, the solvent was removed under reduced pressure and the crude product was purified by column chromatography (cyclohexane/ethyl acetate 18:1→10:1) and the pure product **15** was obtained as white solid in a yield of 71% (133.5 mg, 0.200 mmol).

^{1}**H-NMR** (300 MHz, $CDCl_3$) *δ*/ppm: 7.50 – 7.26 (m, 5H, CH aromatic, [1]), 5.96 – 5.92 (m, 1H, NH, [2]), 5.11 (s, 2H, CH_2, [3]), 5.07 (d, *J* = 4.2 Hz, 1H, CH, [4]), 3.30 – 3.20 (m, 2H, CH_2, [5]), 2.44 – 2.29 (m, 5H, CH, CH_2, [6]), 1.69 – 1.61 (m, 4H, CH_2, [7]), 1.51 – 1.45 (m, 2H, CH_2, [8]), 1.44 – 1.12 (m, 42H, CH_2, [9]), 0.96 – 0.85 (m, 9H, CH_3, [10]).

^{13}**C-NMR** (101 MHz, $CDCl_3$) *δ*/ppm: 174.6, 173.5, 170.2, 137.0, 129.5, 129.1, 78.8, 67.0, 40.1, 35.3, 32.8, 31.4, 30.6, 30.3, 30.1, 27.8, 26.0, 25.7, 23.6, 19.7.

HRMS-FAB-MS of $[C_{42}H_{74}O_5N]^+$calculated: 672.5562, found: 672.5569.

IR (ATR platinum diamond): ν/cm^{-1} = 3255.6, 3090.6, 2916.6, 2849.6, 1740.8, 1649.7, 1571.8, 1466.6, 1382.6, 1159.7, 994.9, 720.9, 696.7, 416.0.

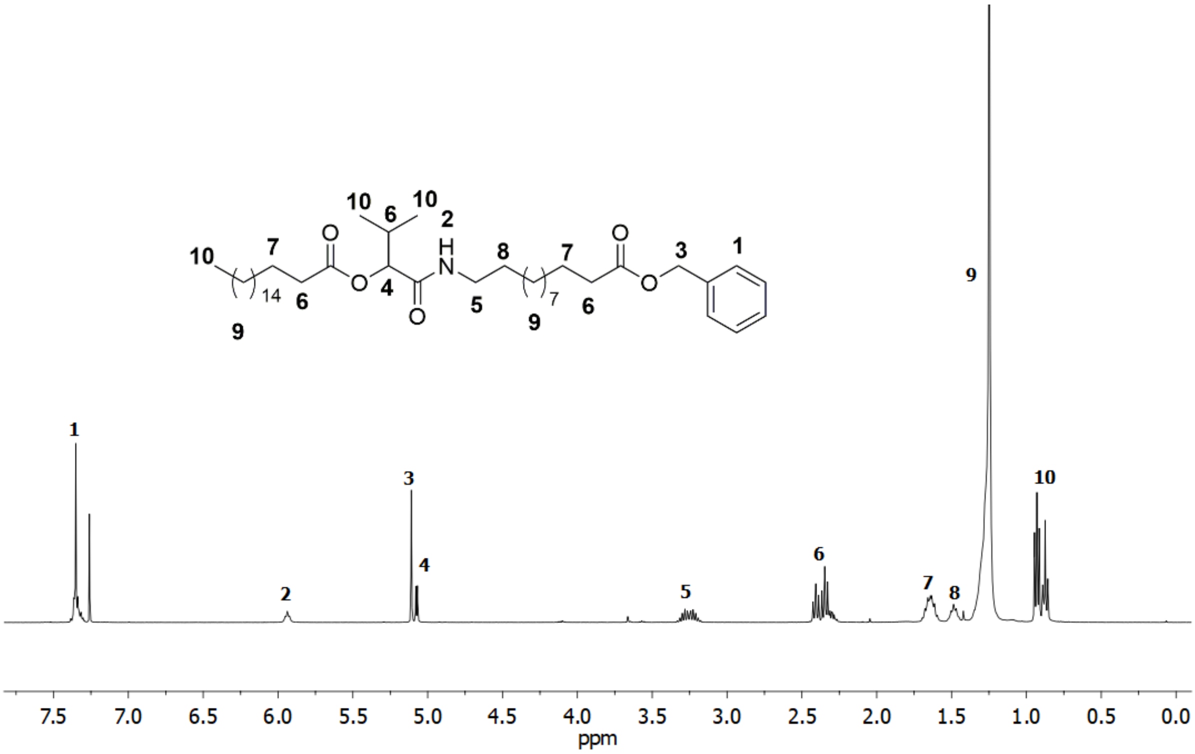

*Supplementary Figure 20. Proton NMR of compound **15** measured in $CDCl_3$.*

Deprotection

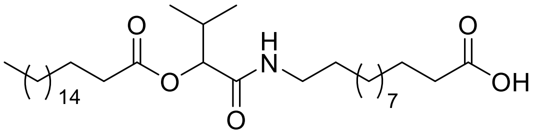

Supplementary Figure 21. Chemical structure of compound ***16****.*

The Passerini product **15** (121 mg, 0.180 mmol, 1.00 eq.) was dissolved in ethyl acetate (0.36 ml) and palladium on activated charcoal **13** (12.1 mg, 10 wt%) was added. The reaction mixture was purged with hydrogen by using a balloon and stirred under hydrogen atmosphere overnight at room temperature. Afterwards, the heterogeneous catalyst was filtered off and the solvent was evaporated under reduced pressure. The desired deprotected product **16** (102 mg, 0.185 mmol) was obtained as white solid in a yield of 97%.

^{1}**H-NMR** (300 MHz, $CDCl_3$) δ/ppm: 5.99 (t, J = 5.9 Hz, 1H, NH, ^{1}), 5.07 (d, J = 4.4 Hz, 1H, CH, ^{2}), 3.39 – 3.15 (m, 2H, CH_2, ^{3}), 2.43 – 2.31 (m, 5H, CH, CH_2, ^{4}), 1.67 – 1.59 (m, 4H, CH_2, ^{5}), 1.51 – 1.46 (m, 2H, CH_2, ^{6}), 1.45 – 1.23 (m, 42H, CH_2, ^{7}), 0.94 – 0.85 (m, 9H, CH_3, ^{8}).

^{13}**C-NMR** (101 MHz, $CDCl_3$) δ/ppm: 178.0, 171.6, 168.4, 38.2, 33.3, 32.9, 30.9, 29.5, 28.6, 28.4, 28.3, 28.1, 28.0, 25.8, 24.0, 23.7.

HRMS-FAB-MS of $[C_{35}H_{68}O_5N]^+$ calculated: 582.5092, found: 582.5099.

IR (ATR platinum diamond): ν/cm^{-1} = 3299.9, 2915.7, 2848.8, 1737.0, 1698.9, 1654.6, 1562.3, 1467.3, 1168.4, 1107.5, 1010.1, 927.8, 720.7, 682.8, 418.3.

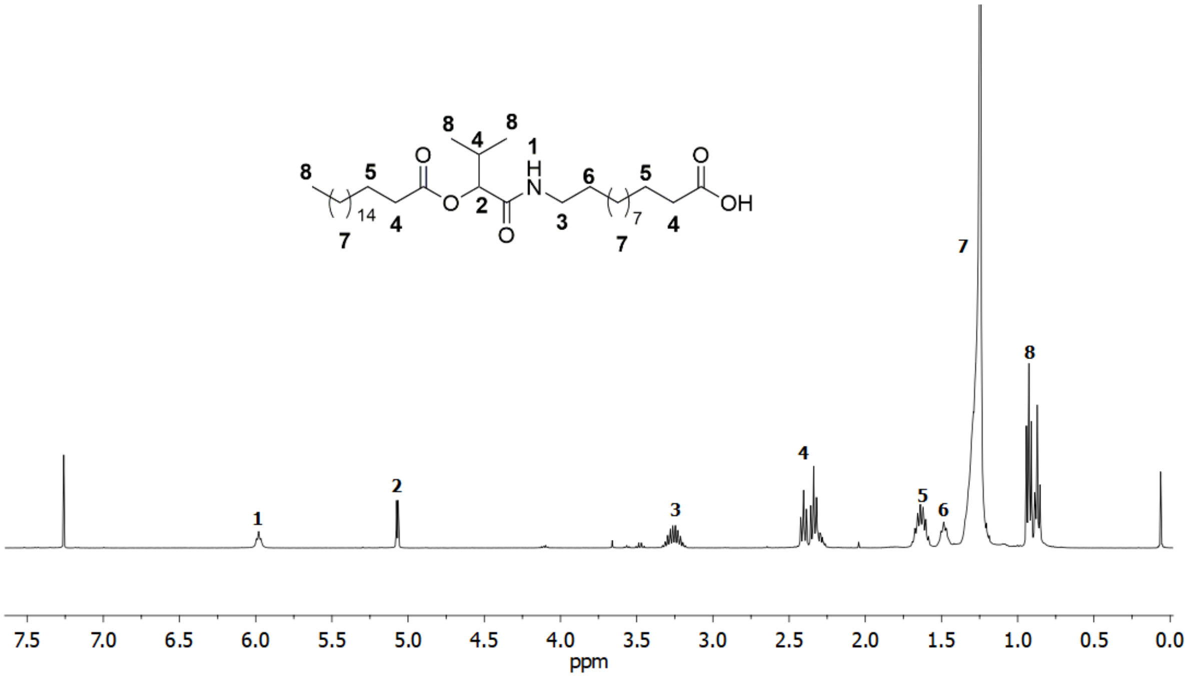

Supplementary Figure 22. Proton NMR of compound ***16*** *measured in* $CDCl_3$*.*

1.3.1.5 Synthesis of monomer M4

Esterification

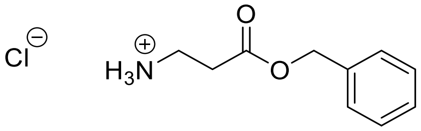

*Supplementary Figure 23. Chemical structure of compound **6d**.*

ß-Alanine **3d** (2.06 g, 23.1 mmol, 1.00 eq.) was suspended in 25 mL THF and benzyl alcohol **4** (27.8 mL, 28.9 g, 266 mol, 11.50 eq.) was added. The suspension was cooled in an ice bath and subsequently thionyl chloride **5** (5.00 mL, 8.16 g, 68.6 mmol, 2.96 eq.) was added dropwise at 0 °C. After addition of the thionyl chloride **5**, the solution was warmed to room temperature and stirred overnight. The yellow solution was then poured into 230 mL diethyl ether and stored in the freezer for one hour. The product was then filtered off and dried under high vacuum. The pure product **6d** was obtained as a white solid in a yield of 81% (4.05 g, 18.7 mmol).

^{1}H-NMR (300 MHz, CD_3OD) δ/ppm: 7.55 – 7.14 (m, 5H, 5 CH aromatic, ^{1}), 5.19 (s, 2H, CH_2, ^{2}), 3.22 (t, J = 5.9 Hz, 2H, CH_2, ^{3}), 2.76 (m, 2H, CH_2, ^{4}).

^{13}C-NMR (75 MHz, CD_3OD) δ/ppm: 171.9, 137.1, 129.6, 129.4, 67.9, 36.4, 32.3.

HRMS-EI-MS of $[C_{10}H_{14}NO_2]^+$: calculated: 180.1019, found: 180.1020.

IR (ATR platinum diamond): ν/cm^{-1} = 3243.9, 2795.8, 2038.6, 1709.8, 1597.0, 1494.9, 1452.4, 1404.8, 1362.8, 1324.6, 1222.6, 1135.4, 1103.6, 1056.5, 981.9, 857.8, 801.7, 748.1, 698.8, 585.4, 569.0, 458.1, 409.1.

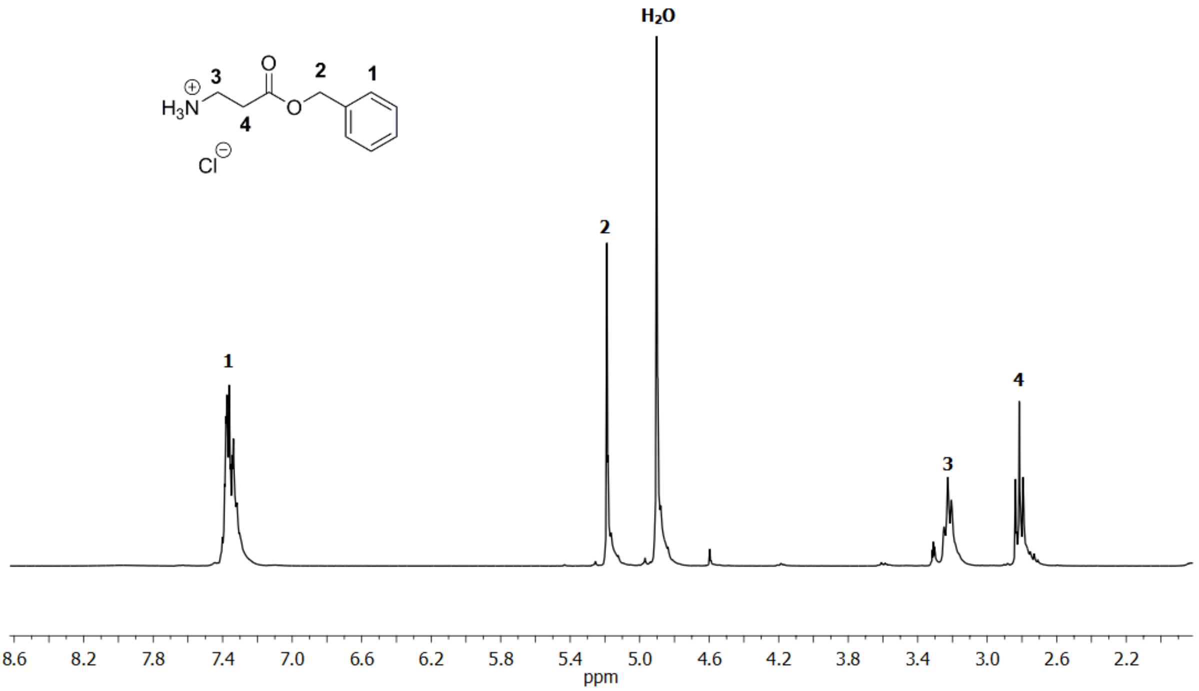

*Supplementary Figure 24. Proton NMR of compound **6d** measured in CD_3OD.*

N-Formylation

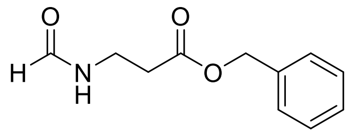

*Supplementary Figure 25. Chemical structure of compound **8d**.*

Substance **6d** (4.05 g, 18.7 mmol, 1.00 eq.) was dissolved in 20.4 mL trimethyl orthoformate **7** (19.8 g, 1.87 mol, 10.0 eq.) and stirred for twelve hours at 100 °C. Trimethyl orthoformate **7** was removed under reduced pressure and the product was purified by column chromatography (hexane/ethyl acetate 2:1 → 0:1), and the yellowish liquid **8d** was obtained in a yield of 52% (2.00 g, 9.72 mmol).

^{1}H-NMR (300 MHz, $CDCl_3$) δ/ppm: 8.01 (s, 1H, CH, [1]), 7.46 – 7.16 (m, 5H, CH aromatic, [2]), 6.63 (s, 1H, NH, [3]), 5.12 (s, 2H, CH_2, [4]), 3.55 - 3.38 (m, 2H, CH_2, [5]), 2.69 - 2.46 (m, 2H, CH_2, [6]).

^{13}C-NMR (75 MHz, $CDCl_3$) δ/ppm: 173.0, 163.8, 137.5, 129.5, 129.2, 67.4, 34.8.

HRMS-EI-MS of $[C_{11}H_{13}NO_3]^{\cdot+}$: calculated: 207.0890, found: 207.0891.

IR (ATR platinum diamond): ν/cm^{-1} = 3291.1, 3033.7, 2947.3, 2869.9, 1729.1, 1658.6, 1521.0, 1454.2, 1383.4, 1315.1, 1213.8, 1166.6, 1066.5, 1002.3, 821.1, 738.0, 696.8, 467.5.

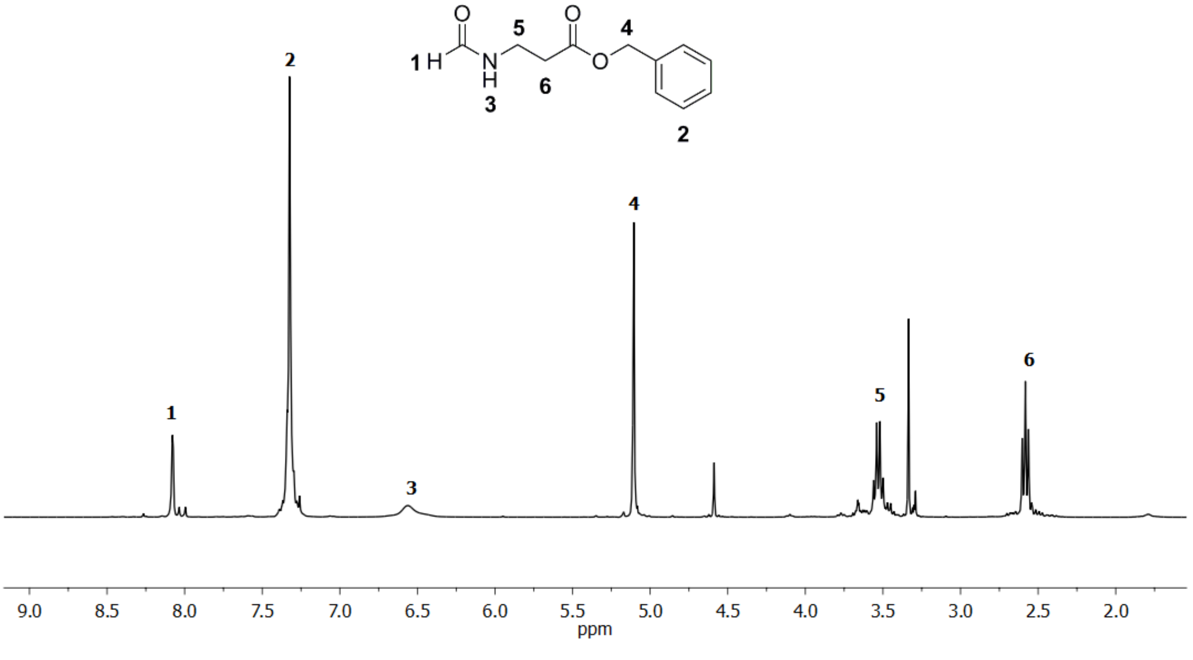

*Supplementary Figure 26. Proton NMR of compound **8d** measured in $CDCl_3$.*

Dehydration

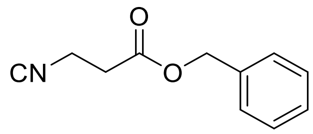

Supplementary Figure 27. Chemical structure of compound ***M4****.*

Benzyl 3-formamidopropanoate **8d** (1.06 g, 5.12 mmol, 1.00 eq.) was dissolved in DCM (25 mL, 0.20 M), diisopropylamine **12** (2.33 mL, 1.68 g, 16.6 mmol, 3.24 eq.) was added and the reaction mixture was cooled to 0 °C. Subsequently, phosphoryl trichloride **9** (0.597 mL, 0.980 g, 6.39 mmol, 1.25 eq.) was added dropwise and the reaction mixture was then stirred at room temperature for two hours. The reaction was quenched by addition of a 20% sodium carbonate solution (9.0 mL) at 0 °C. After stirring this mixture for 30 minutes, 20 mL water and 20 mL DCM were added. The aqueous phase was separated and the organic layer was washed with water (3 x 20 mL) and brine (20 mL). The combined organic layers were dried over sodium sulfate and the solvent was evaporated under reduced pressure. The crude product was then purified by column chromatography (hexane/ethyl acetate 5:1 → 2:1). The product monomer **M4** was obtained as brown oil in a yield of 74% (0.72 g).

^{1}H-NMR (300 MHz, $CDCl_3$) δ/ppm: 7.39 (s, 5H, CH aromatic, ^{1}), 5.18 (s, 2H, CH_2, ^{2}), 3.71 (t, J = 6.8 Hz, 2H, CH_2, ^{3}), 2.78 (t, J = 6.8 Hz, 2H, CH_2, ^{4}).

^{13}C-NMR (75 MHz, $CDCl_3$) δ/ppm: 169.33, 157.59, 135.25, 128.74, 128.64, 128.49, 67.19, 37.17, 34.21.

HRMS-EI-MS of $[C_{11}H_{11}NO_2]^{\cdot+}$: calculated: 189.0790, found: 189.0791.

IR (ATR platinum diamond): ν/cm^{-1} = 3034.3, 2150.2, 1733.7, 1497.9, 1454.6, 1389.5, 1355.3, 1313.0, 1263.0, 1215.2, 1171.1, 1052.7, 986.8, 956.4, 916.1, 832.3, 738.2, 696.8, 577.6, 482.4.

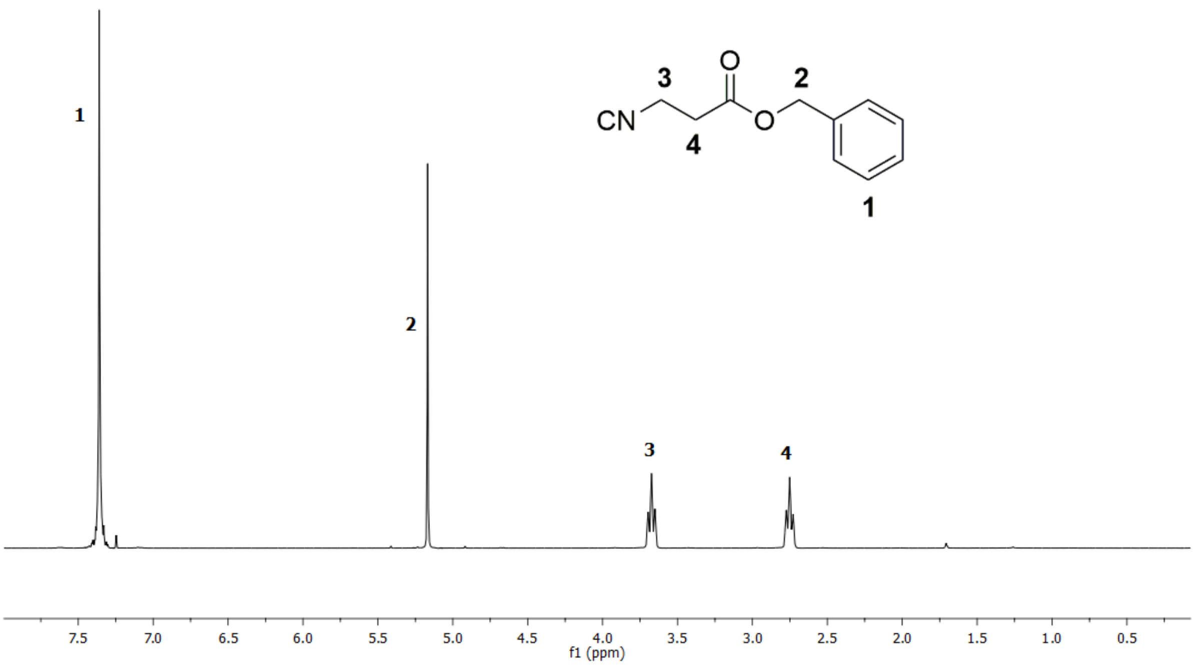

*Supplementary Figure 28. Proton NMR of compound **M4** measured in $CDCl_3$.*

1.3.1.6 Synthesis of monomer M5

Esterification

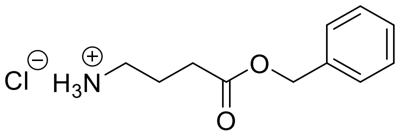

*Supplementary Figure 29. Chemical structure of compound **6e**.*

4-Aminobutyric acid **3e** (15.0 g, 146 mmol, 1.00 eq.) was suspended in THF (145 mL). After the addition of benzyl alcohol **4** (180 mL, 189 g, 1.75 mol, 12.0 eq.), the suspension was cooled in an ice bath to 0 °C and thionyl chloride **5** (32.0 mL, 52.0 g, 437 mmol, 3.00 eq.) was added dropwise. Afterwards, the suspension was stirred at room temperature for 19 hours. Subsequently, 500 mL diethyl ether were added, and the solution was stored in the freezer for 2 hours. The mixture was filtered, and 500 mL diethyl ether were added to the precipitate and stored in the freezer for another two hours. The precipitate was filtered off and the crude product **6e** (29.8 g, 130 mmol) was obtained as a white solid in a yield of 89%.

^{1}H-NMR (300 MHz, CD_3OD) δ/ppm: 7.41 – 7.19 (m, 5H, CH aromatic, [1]), 5.14 (s, 2H, CH_2, [2]), 2.99 (t, *J* = 7.7 Hz, 2H, CH_2, [3]), 2.59 – 2.44 (m, 2H, CH_2, [4]), 2.04 – 1.88 (m, 2H, CH_2, [5]).

^{13}C-NMR (101 MHz, CD_3OD) δ/ppm: 173.77, 137.44, 129.55, 129.29, 127.98, 67.48, 40.03, 31.68, 23.74.

HRMS-EI-MS of $[C_{11}H_{16}O_2N]^+$ calculated: 194.1176 found 194.1181.

IR (ATR platinum diamond): ν /cm^{-1} = 3208.2, 2979.1, 2916.7, 2878.8, 2749.1, 2624.9, 2471.5, 2055.6, 1732.0, 1605.7, 1489.9, 1467.1, 1454.7, 1415.8, 1391.6, 1356.2, 1328.6, 1281.4, 1230.7, 1191.1, 1141.4, 1117.7, 1053.9, 1029.2, 987.7, 949.0, 921.8, 857.6, 771.2, 746.6, 696.8, 579.5, 545.5, 480.1.

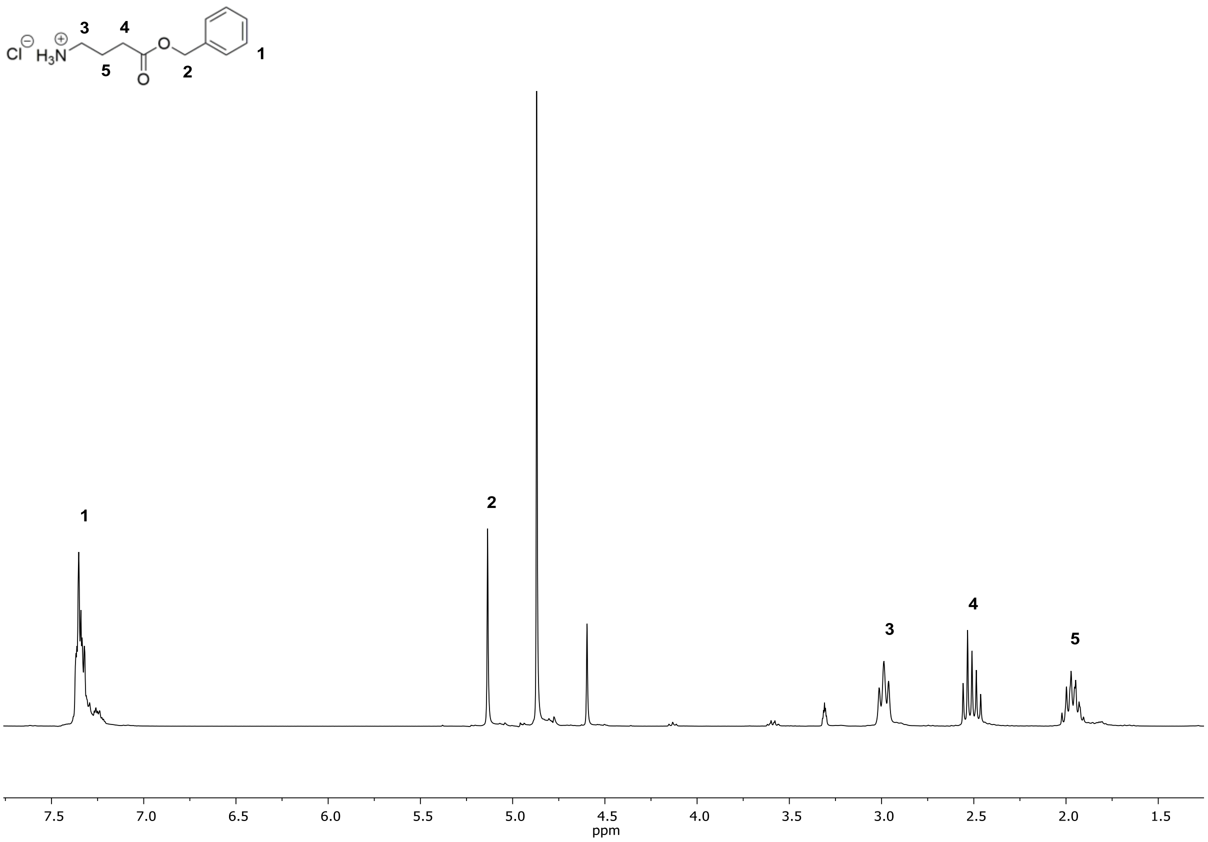

*Supplementary Figure 30. Proton NMR of compound **6e** measured in CD_3OD.*

N-Formylation

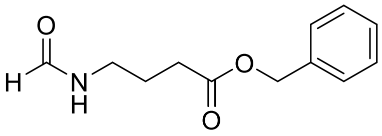

*Supplementary Figure 31. Chemical structure of compound **8e**.*

The ammonium salt **6e** (29.75 g, 130 mmol, 1.00 eq.) was stirred with trimethyl orthoformate **7** (168 mL, 163 g, 1.54 mol, 12.0 eq.) under reflux at 105 °C overnight. Subsequently, the orthoformate **7** was evaporated under reduced pressure. The crude product **8e** (26.0 g, 118 mmol) was obtained as a yellow oil in a yield of 91% and was used without further purification.

^{1}H-NMR (300 MHz, $CDCl_3$) δ/ppm: 8.16 – 7.87 (m, 1H, CH, [1]), 7.38 – 7.24 (m, 5H, CH aromatic, [2]), 6.53 (broad s, 1H, NH, [3]), 5.09 (s, 2H, CH_2, [4]), 3.32 – 3.20 (m, 2H, CH_2, [5]), 2.47 – 2.30 (m, 2H, CH_2, [6]), 1.91 – 1.74 (m, 2H, CH_2, [7]).

^{13}C-NMR (101 MHz, $CDCl_3$) δ/ppm: 172.55, 161.51, 135.48, 128.17, 128.15, 127.82, 65.82, 36.89, 31.06, 24.18.

HRMS-EI-MS of $[C_{12}H_{15}O_3N]^{\cdot+}$ calculated: 221.1052 found 221.1050.

IR (ATR platinum diamond): ν/cm^{-1} = 3283.1, 3062.2, 3034.5, 2941.6, 2874.6, 2753.5, 1730.2, 1659.4, 1531.2, 1497.9, 1454.0, 1384.5, 1352.7, 1320.7, 1235.3, 1161.4, 1082.5, 1028.1, 972.6, 737.5, 696.7, 578.7, 500.1.

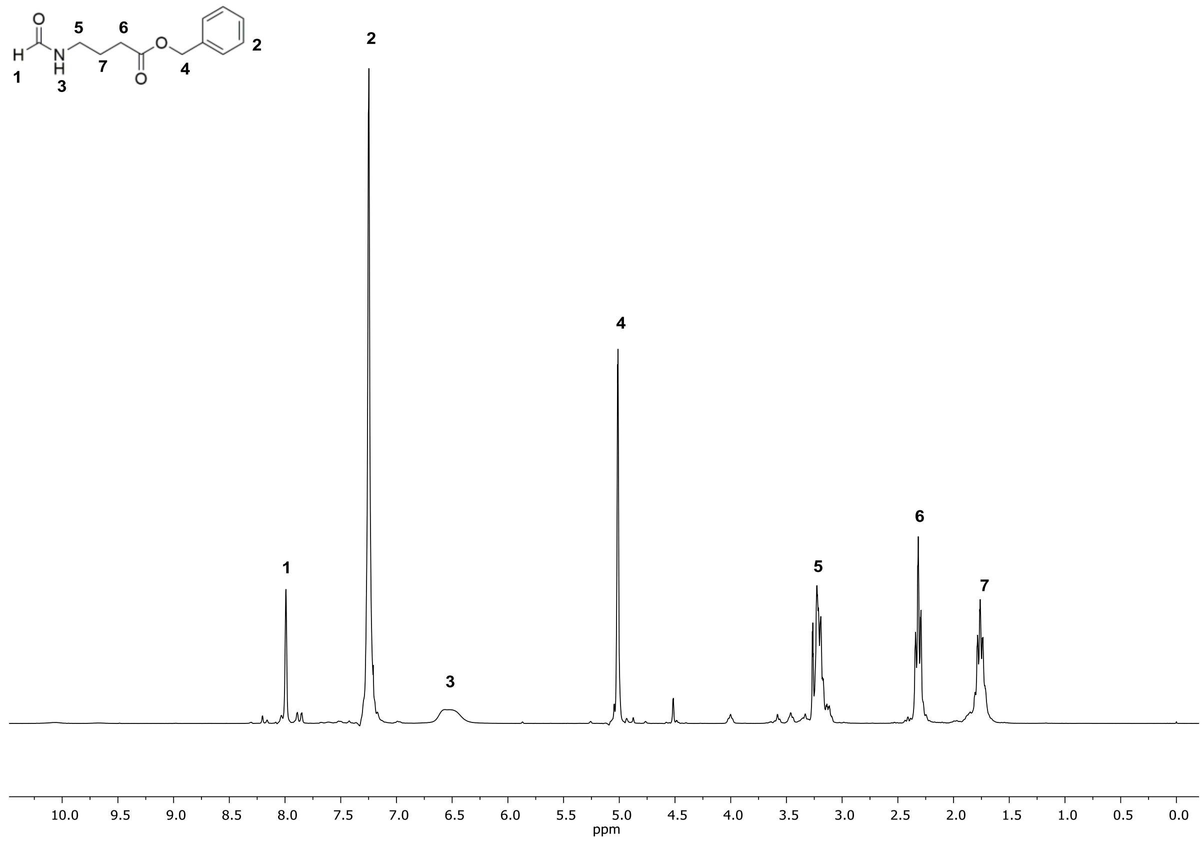

*Supplementary Figure 32. Proton NMR of compound **8e** measured in $CDCl_3$.*

Dehydration

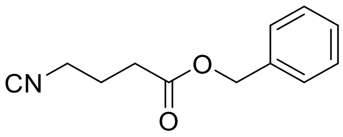

*Supplementary Figure 33. Chemical structure of compound **M5**.*

The formamide **8e** (26.0 g, 118 mmol, 1.00 eq.) was dissolved in DCM (400 mL) and diisopropylamine **12** (70.2 mL, 50.5 g, 500 mmol, 4.20 eq.) was added. The solution was cooled to 0 °C with an ice bath. Then, phosphoryl trichloride **9** (15.2 mL, 25.5 g, 167 mmol, 1.40 eq.) was added dropwise to the reaction mixture. The yellow solution was stirred for two hours at room temperature and was cooled to 0 °C again. The reaction was quenched by the addition of a 20 wt% sodium carbonate solution (150 mL) and stirred for another 45 minutes at room temperature. 80 mL DCM and 80 mL water were added to the mixture and the organic layer was separated. The organic layer was washed with water (3 × 80 mL) and brine (80 mL), dried over sodium sulfate and the solvent was evaporated under reduced pressure. The crude product was purified by column chromatography (cyclohexane/ethyl acetate 10:1 → 3:1). Monomer **M5** (16.5 g, 81.3 mmol) was obtained as a yellow oil in a yield of 69%.

^{1}H-NMR (300 MHz, $CDCl_3$) δ/ppm: 7.37 – 7.19 (m, 5H, CH aromatic, [1]), 5.07 (s, 2H, CH_2, [2]), 3.45 – 3.36 (m, 2H, CH_2, [3]), 2.53 – 2.42 (m, 2H, CH_2, [4]), 1.99 – 1.89 (m, 2H, CH_2, [5]).

^{13}C-NMR (101 MHz, $CDCl_3$) δ/ppm: 171.94, 135.64, 128.63, 128.39, 128.26, 66.57, 40.81, 30.46, 24.29.

HRMS-FAB-MS of $[C_{12}H_{14}O_2N]^+$ calculated: 204.1019 found 204.1024.

IR (ATR platinum diamond): ν/cm^{-1} = 3089.3, 3066.3, 3034.0, 2951.2, 2893.3, 2148.1, 1730.5, 1607.3, 1586.4, 1497.7, 1454.4, 1418.8, 1387.8, 1354.8, 1320.7, 1254.5, 1163.3, 1081.2, 1017.4, 969.7, 901.3, 860.5, 737.9, 697.0, 578.5, 499.9.

R_f:(cyclohexane / ethyl acetate 3:1) = 0.43.

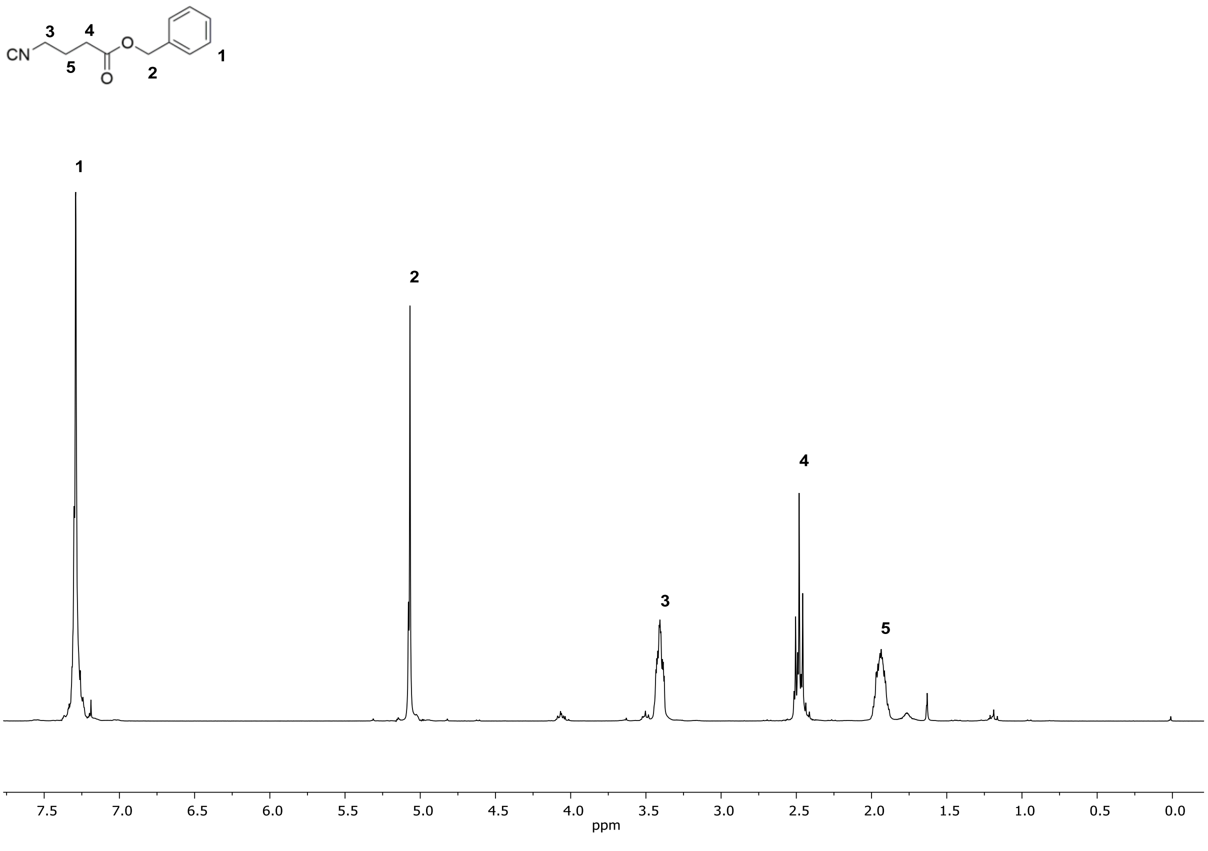

Supplementary Figure 34. Proton NMR of compound ***M5*** *measured in* $CDCl_3$.

1.3.1.7 Evaluation of the reactivity of monomer M5

Passerini reaction

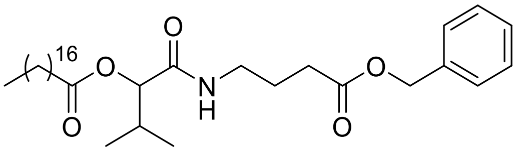

*Supplementary Figure 35. Chemical structure of compound **17**.*

Stearic acid **1** (700 mg, 2.46 mmol, 1.00 eq.) was suspended in DCM (5.0 mL). Subsequently, monomer **M5** (750 mg, 3.69 mmol, 1.50 eq.) and isobutyraldehyde **2a** (337 µL, 266 mg, 3.69 mmol, 1.50 eq.) were added. The reaction mixture was stirred for 48 hours at room temperature and the crude product was purified by column chromatography (cyclohexane/ethyl acetate 10:1 → 4:1), resulting in a yield of 94% of the desired Passerini product **17** (1.30 g, 2.32 mmol), which was obtained as a white solid.

^{1}H-NMR (400 MHz, $CDCl_3$) δ/ppm: 7.40 – 7.30 (m, 5H, CH aromatic, ^{1}), 6.30 (s, 1H, NH, ^{2}), 5.11 (s, 2H, CH_2, ^{3}), 5.07 (d, J = 4.2 Hz, 1H, CH, ^{4}), 3.39 – 3.25 (m, 2H, CH_2, ^{5}), 2.48 – 2.37 (m, 2H, CH_2, ^{6}), 2.35 – 2.22 (m, 1H, CH, ^{7}), 1.86 (quint, J = 6.9 Hz, 2H, CH_2, ^{8}), 1.73 – 1.58 (m, 2H, CH_2, ^{9}), 1.42 – 1.19 (m, 30H, CH_2, ^{10}), 0.96 – 0.84 (m, 9H, CH_3, ^{11}).

^{13}C-NMR (101 MHz, $CDCl_3$) δ/ppm: 173.45, 172.81, 169.74, 128.74, 128.48, 128.36, 77.83, 66.63, 38.82, 34.41, 32.06, 31.86, 30.66, 29.84, 29.79, 29.74, 29.61, 29.50, 29.41, 29.31, 25.12, 24.45, 22.83, 18.94, 16.99, 14.27.

HRMS-FAB-MS of $[C_{34}H_{58}O_5N]^+$ calculated: 560.4310 found: 560.4314.

IR (ATR platinum diamond): ν/cm^{-1} = 3325.5, 3288.6, 3071.3, 2916.3, 2849.6, 1728.9, 1650.6, 1537.7, 1466.8, 1438.9, 1416.3, 1383.5, 1366.0, 1288.3, 1223.2, 1201.2, 1161.7, 1030.7, 1014.4, 980.8, 930.6, 869.5, 798.1, 744.0, 722.4, 696.5, 575.1, 519.7, 474.6, 443.8.

***R*$_f$**:(cyclohexane / ethyl acetate 2:1) = 0.54.

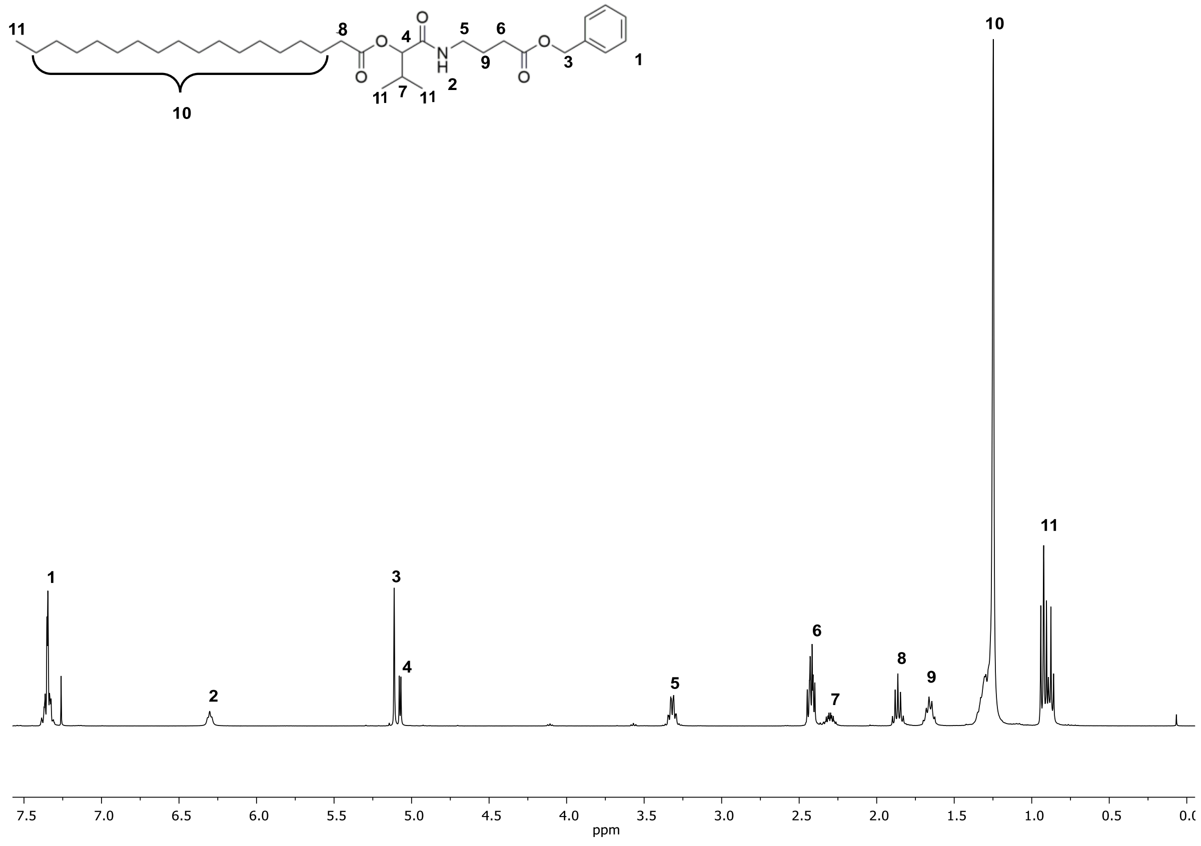

*Supplementary Figure 36. Proton NMR of compound **17** measured in $CDCl_3$.*

Deprotection

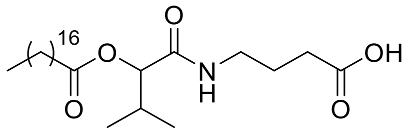

*Supplementary Figure 37. Chemical structure of compound **18**.*

The Passerini product **17** (1.30 g, 2.32 mmol, 1.00 eq.) was dissolved in ethyl acetate (4.70 mL) and palladium on activated charcoal **13** (130 mg, 10 wt%) was added. Subsequently, the reaction mixture was purged with hydrogen using a balloon and stirred under hydrogen atmosphere for 24 hours. Afterwards, the heterogeneous catalyst was filtered off and the solvent was evaporated under reduced pressure. The desired deprotected carboxylic acid **18** (1.03 g, 2.32 mmol) was obtained as a white solid in a yield of 100%.

^{1}H-NMR (300 MHz, $CDCl_3$) δ/ppm: 6.34 (m, 1H, NH, ^{1}), 5.04 (d, J = 4.4 Hz, 1H, CH, ^{2}), 3.40 – 3.26 (m, 2H, CH_2, ^{3}), 2.49 – 2.33 (m, 2H, CH_2, ^{4}), 2.32 – 2.20 (m, 1H, CH, ^{5}), 1.92 – 1.76 (m, 2H, CH_2, ^{6}), 1.73 – 1.52 (m, 2H, CH_2, ^{7}), 1.49 – 1.05 (m, 30H, CH_2, ^{8}), 0.96 – 0.81 (m, 9H, CH_3, ^{9}).

^{13}C-NMR (101 MHz, $CDCl_3$) δ/ppm: 177.76, 170.18, 77.91, 38.67, 34.37, 32.03, 31.43, 30.62, 29.81, 29.76, 29.72, 29.58, 29.47, 29.38, 29.27, 25.09, 24.54, 22.80, 18.87, 17.04, 14.22.

HRMS-FAB-MS of $[C_{27}H_{52}O_5N]^+$ calculated: 470.3840 found: 470.3841.

IR (ATR platinum diamond): ν/cm^{-1} = 3284.1, 3100.4, 2916.1, 2849.2, 2233.5, 2161.3, 2076.2, 2030.7, 2019.5, 1988.0, 1967.5, 1742.0, 1698.7, 1652.1, 1569.4, 1466.8, 1439.5, 1406.2, 1380.5, 1345.7, 1287.6, 1253.8, 1219.1, 1160.8, 1137.0, 1094.4, 1066.7, 1012.5, 937.5, 862.7, 811.6, 722.1, 676.7, 491.9, 467.2, 410.7.

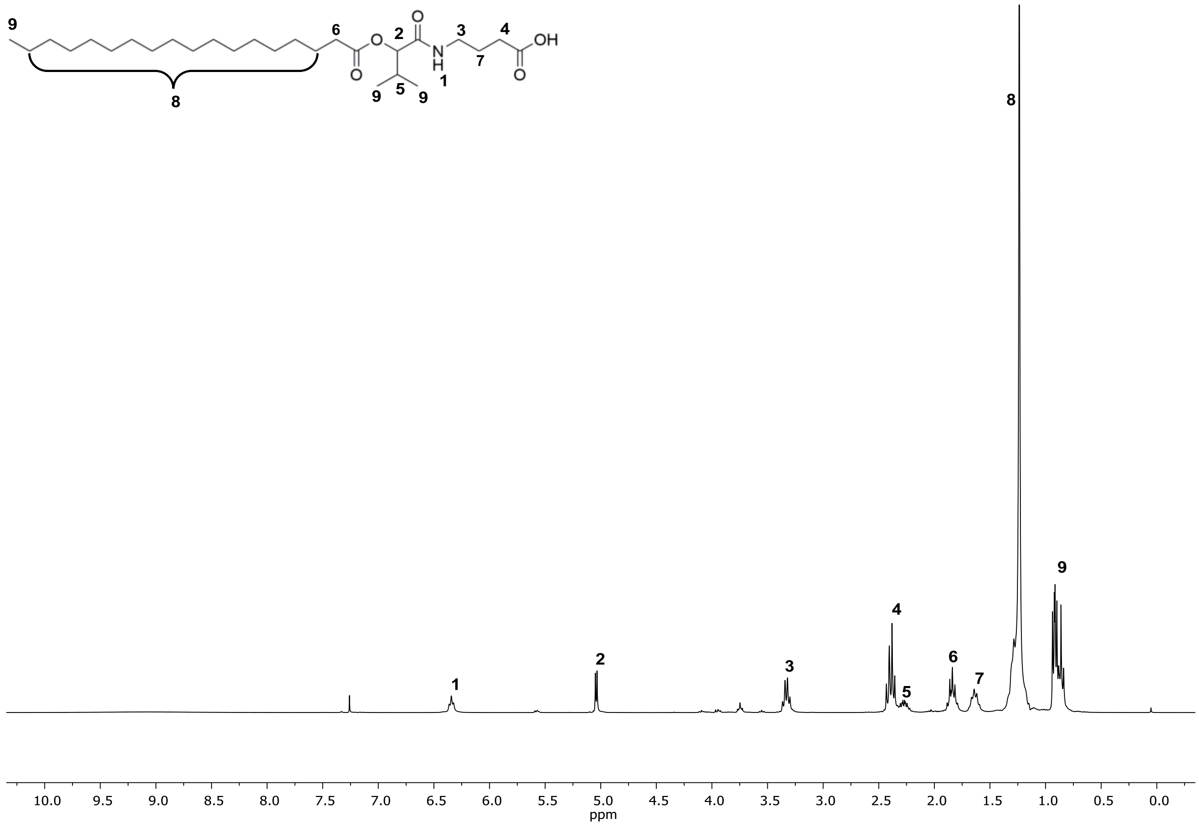

*Supplementary Figure 38. Proton NMR of compound **18** measured in $CDCl_3$.*

1.3.1.8 Synthesis of monomer M6

Esterification

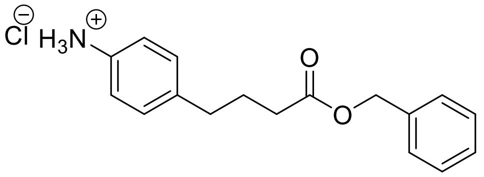

*Supplementary Figure 39. Chemical structure of compound **6f**.*

4-(4-Aminophenyl)butanoic acid **3f** (1.00 g, 5.58 mmol, 1.00 eq.) was dissolved in THF (5.6 mL) and benzyl alcohol **4** (6.38 mL, 6.64 g, 61.4 mmol, 11.0 eq.) was added. Subsequently, the mixture was cooled to 0 °C and thionyl chloride **5** (1.42 mL, 2.32 g, 19.5 mmol, 3.50 eq.) was added dropwise. Afterwards, the solution was stirred at room temperature for 21 hours. Diethyl ether (10 mL) was added and the solution was stored in the freezer overnight. The white precipitate was filtered off and washed with cold diethyl ether (5 mL). The desired product **6f** (1.34 g, 4.37 mmol) was obtained as a white solid in a yield of 78%.

^{1}H-NMR (300 MHz, CD_3OD) δ/ppm: 7.36 – 7.33 (m, 9H, CH aromatic, ^{1}), 5.12 (s, 2H, CH_2, ^{2}), 2.69 (t, J = 7.6 Hz, 2H, CH_2, ^{3}), 2.39 (t, J = 7.2 Hz, 2H, CH_2, ^{4}), 1.94 (quint, J = 7.4 Hz, 2H, CH_2, ^{5}).

^{13}C-NMR (75 MHz, CD_3OD) δ/ppm: 207.7, 174.7, 144.4, 137.7, 131.3, 129.6, 129.3, 127.4, 124.1, 67.2, 35.3, 34.2, 27.6.

HRMS-FAB-MS of $[C_{17}H_{20}O_2N]^+$ calculated: 270.1489, found: 270.1496.

IR (ATR platinum diamond): ν/cm^{-1} = 2947.5, 2920.5, 2803.5, 2597.8, 1719.6, 1567.3, 1532.4, 1607.7, 1454.2, 1351.4, 1190.9, 1106.6, 962.6, 905.0, 812.5, 744.6, 607.3, 573.9, 487.4.

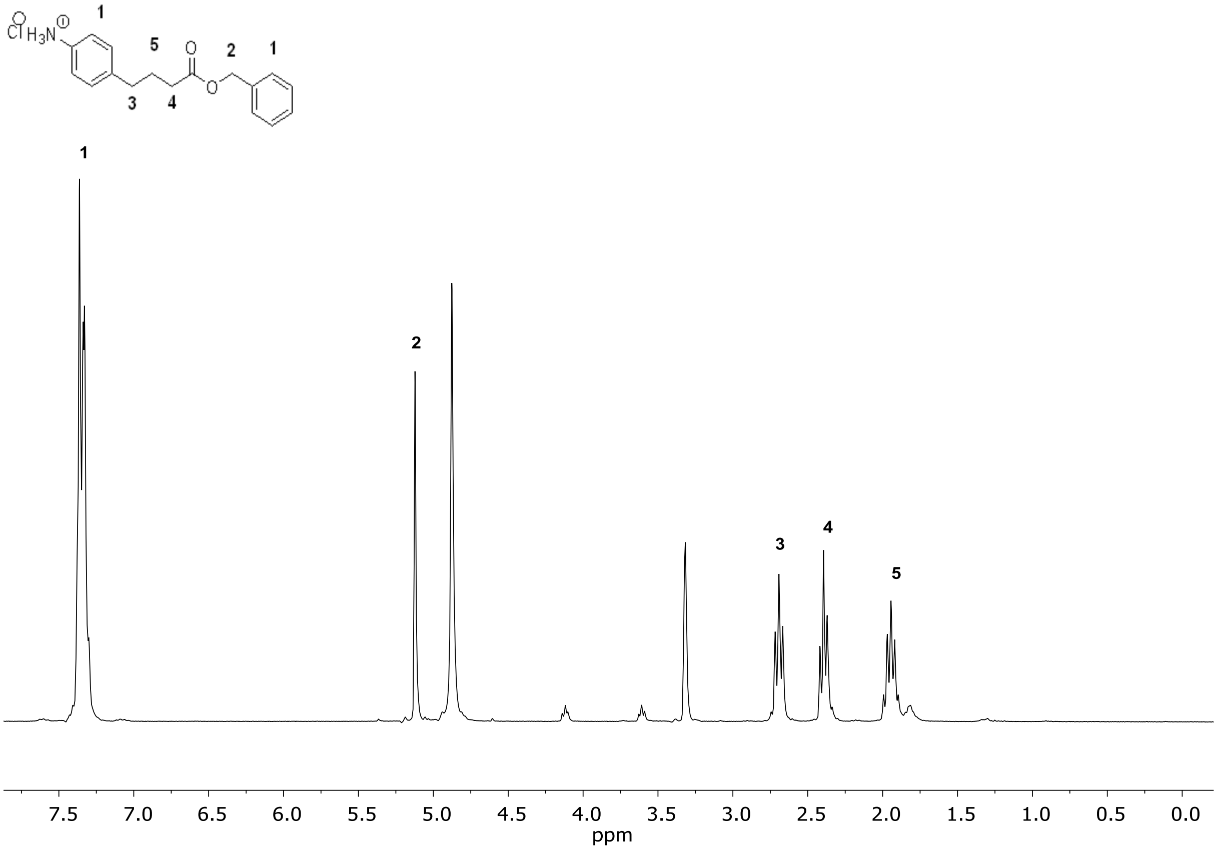

*Supplementary Figure 40. Proton NMR of compound **6f** measured in CD_3OD.*

N-Formylation

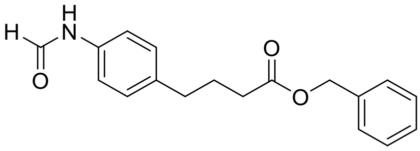

*Supplementary Figure 41. Chemical structure of compound **8f**.*

The ammonium salt **6f** (700 mg, 2.29 mmol, 1.00 eq.) was dissolved in trimethyl orthoformate **7** (2.48 mL, 2.43 g, 22.9 mmol, 10.0 eq.) and heated to 100 °C. The reaction was stirred under reflux overnight. Subsequently, the crude product was purified by column chromatography (cyclohexane/ethyl acetate 5:1 → 1:1). The desired *N*-formamide **8f** (492 mg, 1.65 mmol) was obtained as colourless oil in a yield of 72%.

^{1}H-NMR (300 MHz, CD_3OD) δ/ppm: 8.63 – 8.21 (m, 1H, CH, [1]), 7.46 – 7.43 (m, 2H, CH aromatic, [2]), 7.35 – 7.29 (m, 5H, CH aromatic, [3]), 7.15 – 7.04 (m, 2H, CH aromatic, [4]), 5.09 (s, 2H, CH_2, [5]), 2.57 (t, *J* = 7.6 Hz, 2H, CH_2, [6]), 2.35 (t, *J* = 7.3 Hz, 2H, CH_2, [7]), 1.89 (quint, *J* = 7.4 Hz, 2H, CH_2, [8]).

^{13}C-NMR (75 MHz, CD_3OD) δ/ppm: 174.9, 161.5, 144.0, 139.1, 130.7, 130.0, 129.5, 129.3, 121.2, 119.9, 94.5, 67.2, 35.4, 34.3, 27.8.

HRMS-FAB-MS of $[C_{18}H_{20}O_3N]^+$ calculated: 298.1438, found: 298.1139.

IR (ATR platinum diamond): ν/cm^{-1} = 3307.5, 3274.6, 3164.3, 3062.7, 3031.9, 2937.2, 2865.2, 1729.8, 1674.3, 1608.5, 1518.0, 1454.2, 1411.0, 1299.9, 1254.7, 1139.5, 1001.7, 964.7, 837.2, 736.4, 697.3, 543.0.

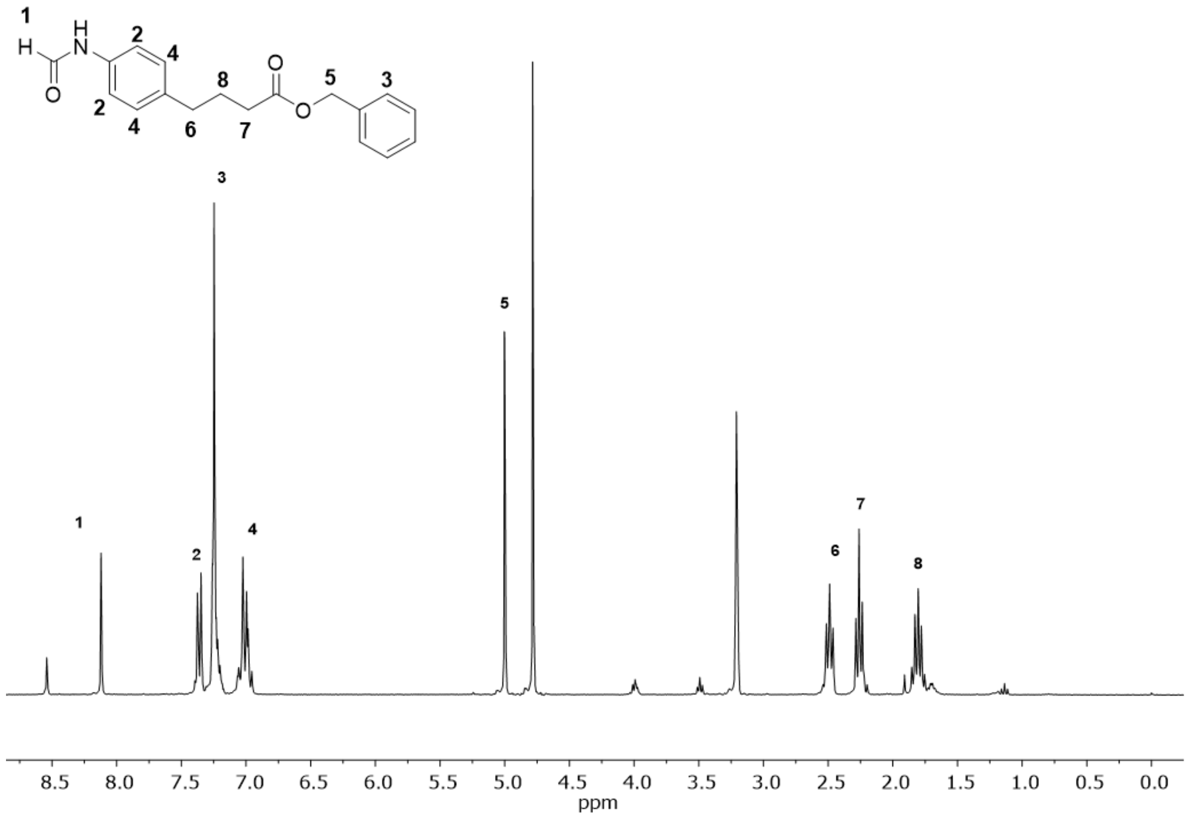

*Supplementary Figure 42. Proton NMR of compound **8f** measured in CD_3OD.*

Dehydration

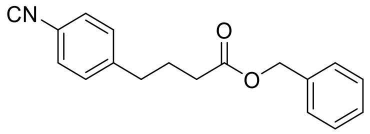

*Supplementary Figure 43. Chemical structure of compound **M6**.*

The formamide **8f** (433 mg, 1.46 mmol, 1.00 eq.) was dissolved in DCM (4.60 mL) and diisopropylamine **12** (0.634 mL, 456 mg, 4.51 mmol, 3.10 eq.) was added. The colourless solution was cooled to 0 °C using an ice bath. Then, phosphoryl trichloride **9** (0.173 mL, 290 mg, 1.89 mmol, 1.30 eq.) was added dropwise. The solution was stirred for two hours at room temperature. Subsequently, the yellow reaction mixture was cooled to 0 °C, quenched by addition of a 20 wt% sodium carbonate solution (1.79 mL) and was stirred for another 30 minutes at room temperature. DCM (2 mL) and water (2 mL) were added to the mixture and the organic layer was separated and washed with water (3x 2 mL) and brine (1x 2 mL). The combined organic layers were dried over sodium sulfate and the solvent was evaporated under reduced pressure. The crude product was purified by column chromatography (cyclohexane/ethyl acetate 18:1 → 6:1). The desired isocyanide monomer **M6** (275 mg, 985 µmol) was obtained as green liquid in a yield of 68%.

^{1}H-NMR (300 MHz, $CDCl_3$) *δ*/ppm: 7.40 – 7.33 (m, 5H, CH aromatic, ^{1}), 7.29 – 7.26 (m, 2H, CH aromatic, ^{2}), 7.17 – 7.14 (m, 2H, CH aromatic, ^{3}), 5.11 (s, 2H, CH_2, ^{4}), 2.65 (t, *J* = 7.7 Hz, 2H, CH_2, ^{5}), 2.37 (t, *J* = 7.3 Hz, 2H, CH_2, ^{6}), 1.95 (quint, *J* = 7.5 Hz, 2H, CH_2,^{7}).

^{13}C-NMR (75 MHz, $CDCl_3$) *δ*/ppm: 173.0, 163.5 143.2, 136.0, 129.5, 128.7, 128.4, 128.3, 126.5, 101.2, 66.4, 34.8, 33.5, 26.3.

HRMS-FAB-MS of $[C_{18}H_{18}O_2N]^+$ calculated: 280.1332, found: 280.1336.

IR (ATR platinum diamond): ν/cm^{-1} = 3064.8, 3033.9, 2943.4, 2867.3, 2122.7, 1731.9, 1505.6, 1454.2, 1384.3, 1141.6, 1040.8, 1003.8, 964.7, 845.4, 822.8, 736.4, 697.2, 547.1, 512.2, 483.4.

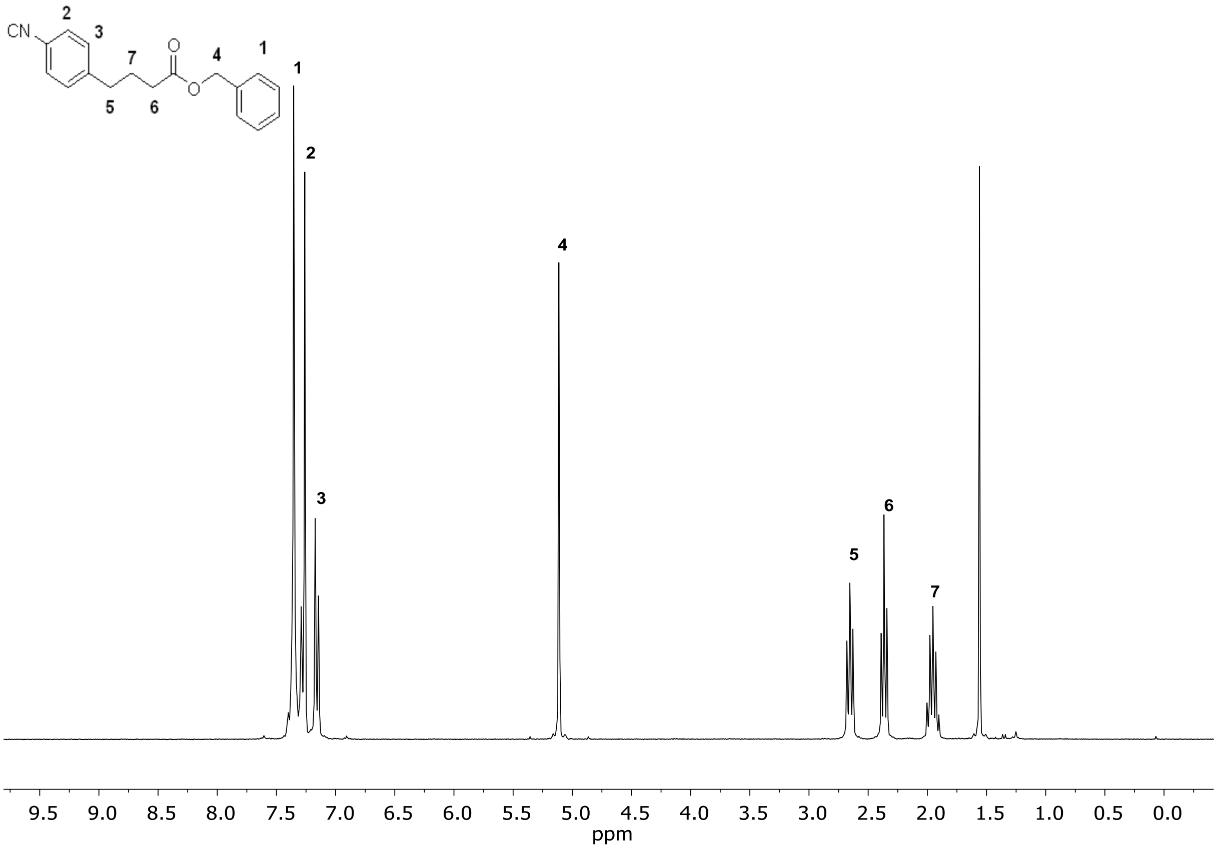

*Supplementary Figure 44. Proton NMR of compound **M6** measured in $CDCl_3$.*

1.3.1.9 Evaluation of the reactivity of monomer M6

Passerini reaction

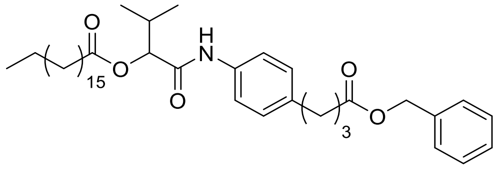

*Supplementary Figure 45. Chemical structure of compound **19**.*

Stearic acid **1** (199 mg, 699 µmol, 1.00eq.) was suspended in DCM (1.75 ml). Subsequently, monomer **M6** (195 mg, 699 µmol, 1.00 eq.) and isobutyraldehyde **2a** (63.8 µL, 50.4 mg, 699 µmol, 1.00 eq.) were added. Then, the reaction mixture was stirred for 24 hours at room temperature. The crude product was purified by column chromatography (cyclohexane/ethyl acetate 15:1 → 10:1) and the desired Passerini product **19** (365 mg, 574 µmol) was obtained as a white solid in a yield of 82%.

^{1}H-NMR (300 MHz, $CDCl_3$) δ/ppm: 7.61 (s, 1H, NH, [1]), 7.41 (d, J = 8.4 Hz, 2H, CH aromatic, [2]), 7.38 – 7.31 (m, 5H, CH aromatic, [3]), 7.11 (d, J = 8.4 Hz, 2H, CH aromatic, [4]), 5.19 (d, J = 4.6 Hz, 1H, CH, [5]), 5.11 (s, 2H, CH_2, [6]), 2.61 (t, J = 7.5 Hz, 2H, CH_2, [7]), 2.47 (t, J = 7.5 Hz, 2H, CH_2, [8]), 2.42 – 2.34 (m, 3H, CH_2, CH, [9]), 1.94 (quint, J = 7.5 Hz, 2H, CH_2, [10]), 1.75 – 1.64 (m, 2H, CH_2, [11]), 1.42 – 1.17 (m, 28H, CH_2, [12]), 1.00 (d, J = 6.8 Hz, 6H, CH_3, [13]), 0.88 (t, J = 6.5 Hz, 3H, CH_3, [14]).

^{13}C-NMR (75 MHz, $CDCl_3$) δ/ppm: 172.6, 167.6, 163.1, 157.0, 150.8, 136.2, 135.0, 129.2, 128.7, 128.4, 120.4, 101.3, 78.2, 66.3, 34.6, 34.5, 33.7, 32.1, 30.9, 29.8, 29.6, 29.5, 29.4, 29.3, 26.7, 25.2, 24.2, 22.8, 18.9, 17.2, 14.3.

FAB-MS of $[C_{40}H_{62}O_5N]^+$ calculated: 636.4623, found: 636.4627.

IR (ATR platinum diamond): ν/cm^{-1} = 3311.6, 2955.7, 2916.7, 2850.8, 1723.7, 1672.2, 1598.2, 1532.4, 1468.6, 1458.3, 1415.1, 1386.3, 1357.5, 1316.4, 1260.9, 1238.3, 1213.6, 1184.8, 1172.4, 1145.7, 1108.7, 1030.5, 995.5, 977.0, 929.7, 835.1, 789.8, 775.4, 736.4, 719.9, 697.3, 672.6, 510.1.

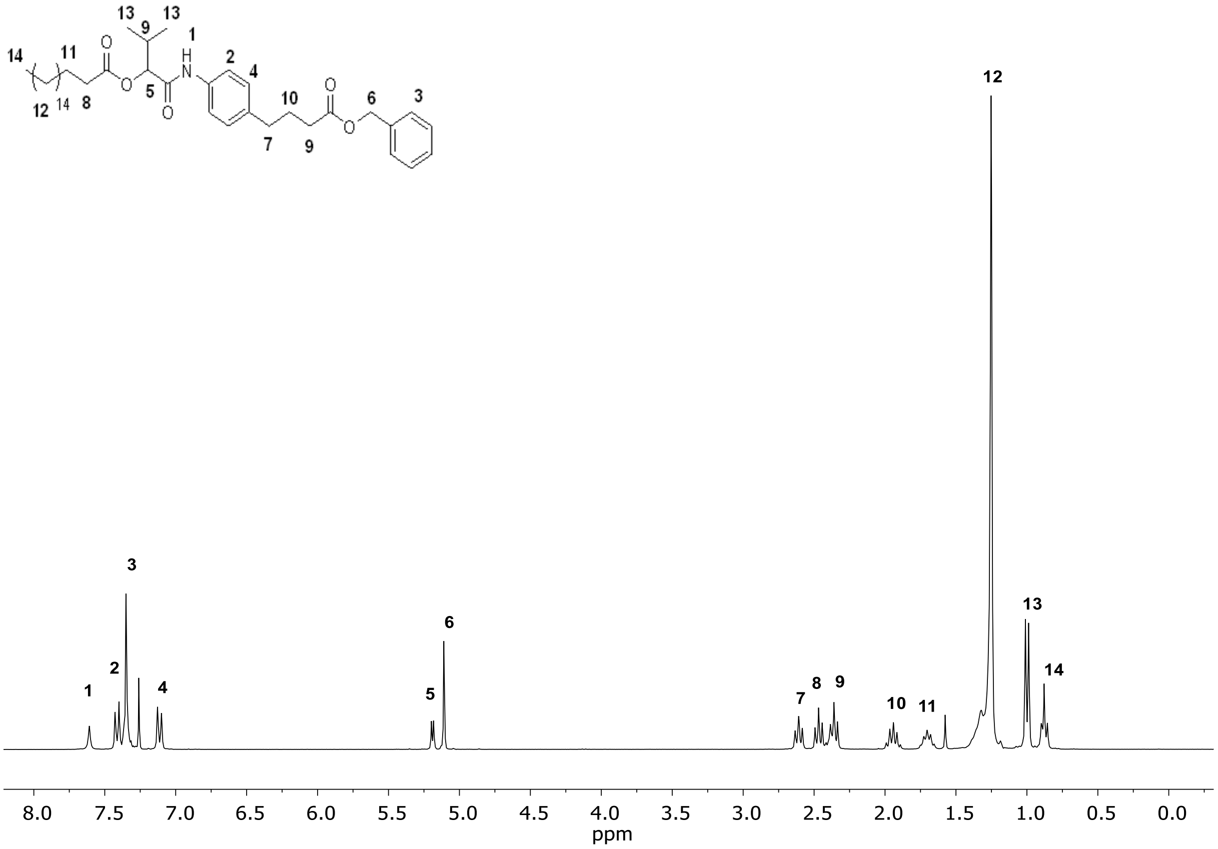

*Supplementary Figure 46. Proton NMR of compound **19** measured in $CDCl_3$.*

Deprotection

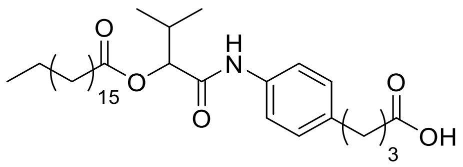

*Supplementary Figure 47. Chemical structure of compound **20**.*

The Passerini product **19** (302 mg, 474 µmol, 1.00 eq.) was dissolved in ethyl acetate (3.20 mL) and palladium on activated charcoal **13** (30.2 mg, 10 wt%) was added. Subsequently, the reaction mixture was purged with hydrogen using a balloon and stirred under hydrogen atmosphere overnight. Afterwards, the heterogeneous catalyst was filtered off and the solvent was evaporated under reduced pressure. The desired deprotected carboxylic acid **20** (237 mg, 434 µmol) was obtained as a white solid in a yield of 92%.

^{1}H-NMR (300 MHz, $CDCl_3$) δ/ppm: 7.64 (s, 1H, NH, [1]), 7.43 (d, *J* = 8.3 Hz, 2H, CH aromatic, [2]), 7.15 (d, *J* = 8.3 Hz, 2H, CH aromatic, [3]), 5.19 (d, *J* = 4.6 Hz, 1H, CH, [4]), 2.64 (t, *J* = 7.5 Hz, 2H, CH_2, [5]), 2.47 (t, *J* = 7.5 Hz, 2H, CH_2, [6]), 2.41 – 2.33 (m, 3H, CH_2, CH, [7]), 1.98 – 1.84 (m, 2H, CH_2, [8]), 1.70 (t, *J* = 7.2 Hz, 2H, CH_2, [9]), 1.40 – 1.17 (m, 28H, CH_2, [10]), 1.00 (d, *J* = 6.8 Hz, 6H, CH_3, [11]), 0.87 (t, *J* = 6.8 Hz, 3H, CH_3 [12]).

^{13}C-NMR (75 MHz, $CDCl_3$) δ/ppm: 179.2, 172.8, 167.7, 138.0, 135.0, 129.1, 120.5, 78.2, 34.5, 34.4, 33.3, 32.0, 30.9, 29.8, 29.7, 29.6, 29.5, 29.4, 29.3, 26.4, 25.2, 22.8, 18.9, 17.3, 14.2.

HR-FAB-MS of $[C_{33}H_{56}O_5N]^+$ calculated: 546.4153, found: 546.4157.

IR (ATR platinum diamond): ν/cm^{-1} = 3336.3, 2953.7, 2910.7, 2848.8, 1744.2, 1699.0, 1672.2, 1596.1, 1522.1, 1468.6, 1413.1, 1382.2, 1310.2, 1248.5, 1213.6, 1153.9, 1104.5, 1038.7, 1014.0, 923.5, 837.2, 794.0, 722.0, 672.6, 569.8, 516.3.

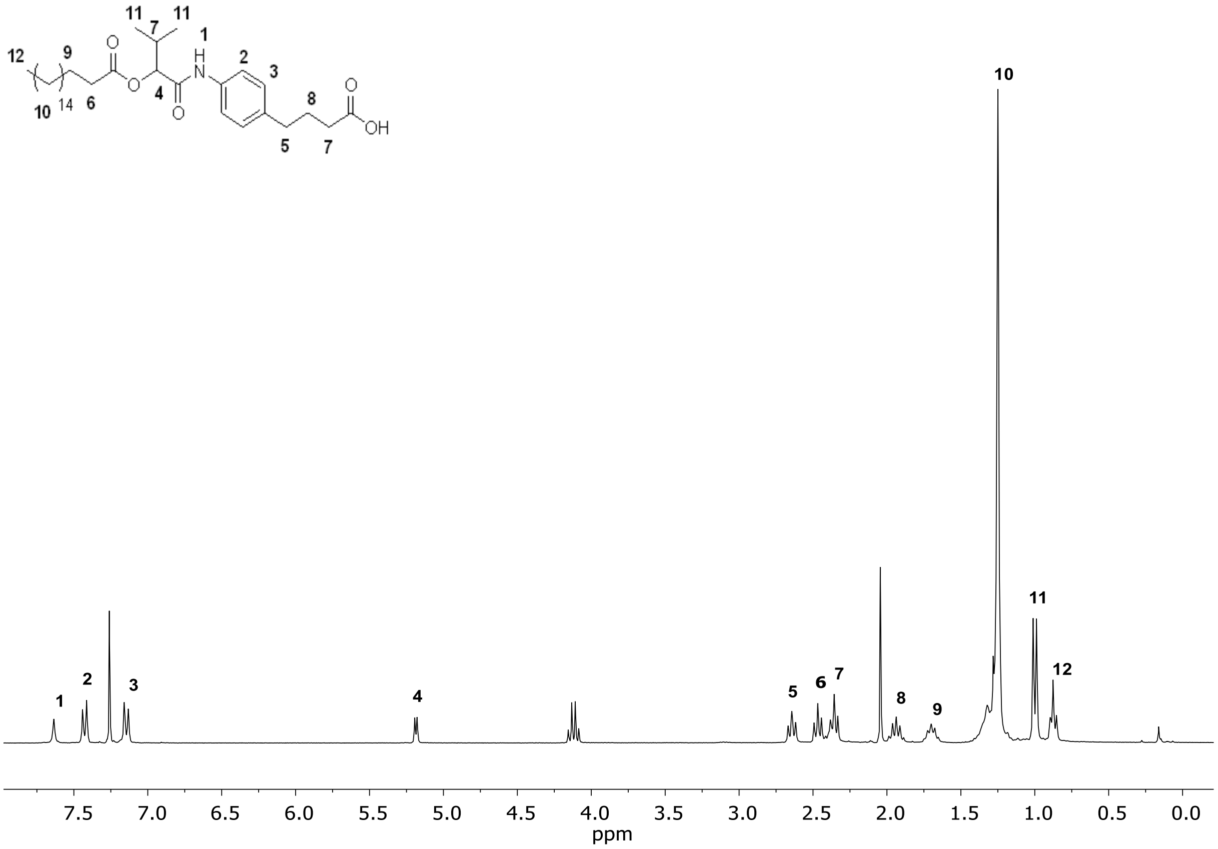

*Supplementary Figure 48. Proton NMR of compound **20** measured in $CDCl_3$.*

1.3.1.10 Synthesis of monomer M7

Esterification

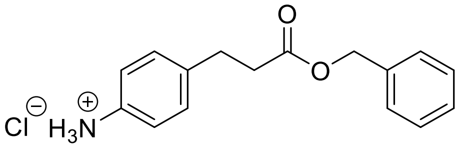

*Supplementary Figure 49. Chemical structure of compound **6g**.*

3-(4-Aminophenyl) propanoic acid **3g** (12.6 g, 75 mmol, 1.00 eq.) was suspended in THF (75 mL). After the addition of benzyl alcohol **4** (85.4 mL, 89.2 g, 825 mmol, 11.0 eq.), the suspension was cooled with an ice bath to 0 °C and thionyl chloride **5** (19.2 mL, 31.2 g, 263 mmol, 3.50 eq.) was added dropwise. Afterwards, the suspension was stirred at room temperature for 15 hours. Subsequently, 500 mL diethyl ether were added, and the solution was stored in the freezer for 2 hours. The mixture was filtered, and 500 mL diethyl ether were added to the precipitate and stored in the freezer for another 2 hours. The precipitate was filtered off and the crude product **6g** (20.3 g, 69.6 mmol) was obtained as a white solid in a yield of 93%.

^{1}H-NMR (300 MHz, CD_3OD) δ/ppm: 7.36 – 7.25 (m, 9H, CH aromatic, [1]), 5.07 (s, 2H, CH_2, [2]), 2.97 (t, J = 7.4 Hz, 2H, CH_2, [3]), 2.69 (t, J = 7.4 Hz, 2H, CH_2, [4]).

^{13}C-NMR (126 MHz, CD_3OD) δ/ppm: 173.88, 143.36, 137.48, 131.21, 129.92, 129.51, 129.26, 129.21, 124.12, 67.28, 36.33, 31.24.

HRMS-FAB-MS of $[C_{16}H_{18}O_2N]^+$ calculated: 256.1332 found: 256.1337.

IR (ATR platinum diamond): ν/cm^{-1} = 2914.7, 2837.1, 2601.3, 1963.0, 1738.3, 1620.9, 1580.7, 1566.6, 1527.8, 1508.2, 1453.0, 1379.0, 1345.4, 1305.8, 1277.7, 1258.4, 1212.3, 1193.0, 1147.1, 1109.4, 1081.0, 1043.2, 1025.1, 990.9, 953.0, 933.0, 912.9, 834.5, 812.6, 757.7, 732.1, 695.0, 641.8, 573.3, 546.4, 499.1, 481.6, 461.1.

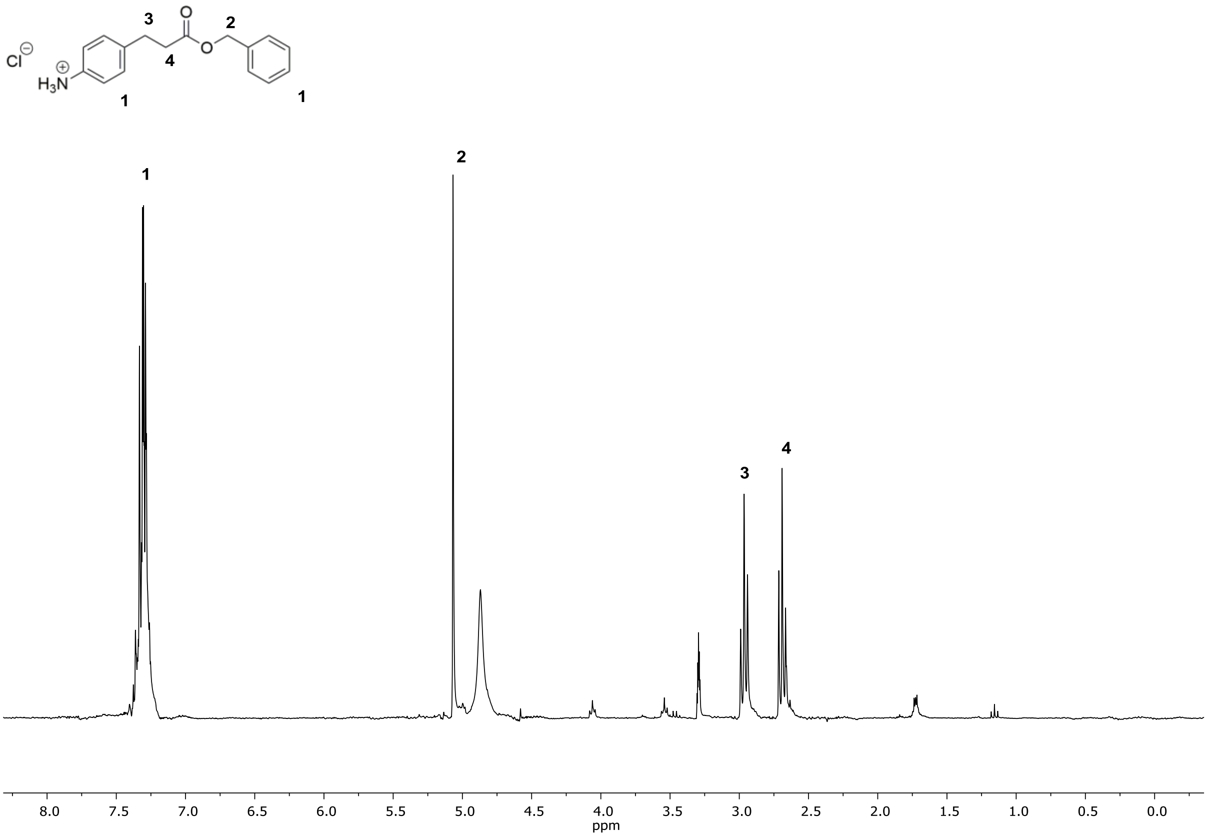

*Supplementary Figure 50. Proton NMR of compound **6g** measured in CD_3OD.*

N-Formylation

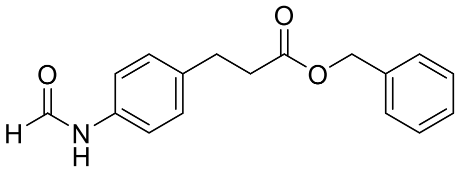

*Supplementary Figure 51. Chemical structure of compound **8g**.*

The ammonium salt **6g** (20.3 g, 69.6 mmol, 1.00 eq.) was stirred with trimethyl orthoformate **7** (78.0 mL, 75.7 g, 716 mmol, 10.2 eq.) under reflux at 105 °C overnight. Subsequently, the orthoformate **7** was evaporated under reduced pressure. The crude product **8g** (19.5 g, 68.9 mmol) was obtained as a yellow oil in a yield of 99% and was used without further purification.

^{1}H-NMR (300 MHz, $CDCl_3$) δ/ppm: 8.66 – 8.56 (m, 0.5H, CH, [1]), 8.33 – 8.01 (m, 0.5H, CH, [1]), 7.45 – 6.95 (m, 9H, CH aromatic,[2]), 5.08 (s, 2H, CH_2, [3]), 2.96 – 2.86 (m, 2H, CH_2, [4]), 2.68 – 2.57 (m, 2H, CH_2, [5]).

^{13}C-NMR (101 MHz, $CDCl_3$) δ/ppm: 172.76, 163.05, 159.29, 137.43, 136.78, 135.86, 135.37, 135.15, 120.21, 119.12, 66.38, 35.97, 30.36.

HRMS-FAB-MS of $[C_{17}H_{18}O_3N]^+$ calculated: 284.1281 found: 284.1288.

IR (ATR platinum diamond): ν/cm^{-1} = 3306.5, 3200.4, 3113.5, 3033.0, 2944.7, 2867.0, 2775.0, 1897.5, 1728.7, 1682.1, 1603.0, 1518.5, 1454.1, 1412.0, 1381.9, 1352.7, 1291.4, 1256.5, 1147.4, 1109.0, 1025.9, 984.9, 908.4, 823.7, 735.6, 696.1, 578.9, 536.1, 454.8, 411.1.

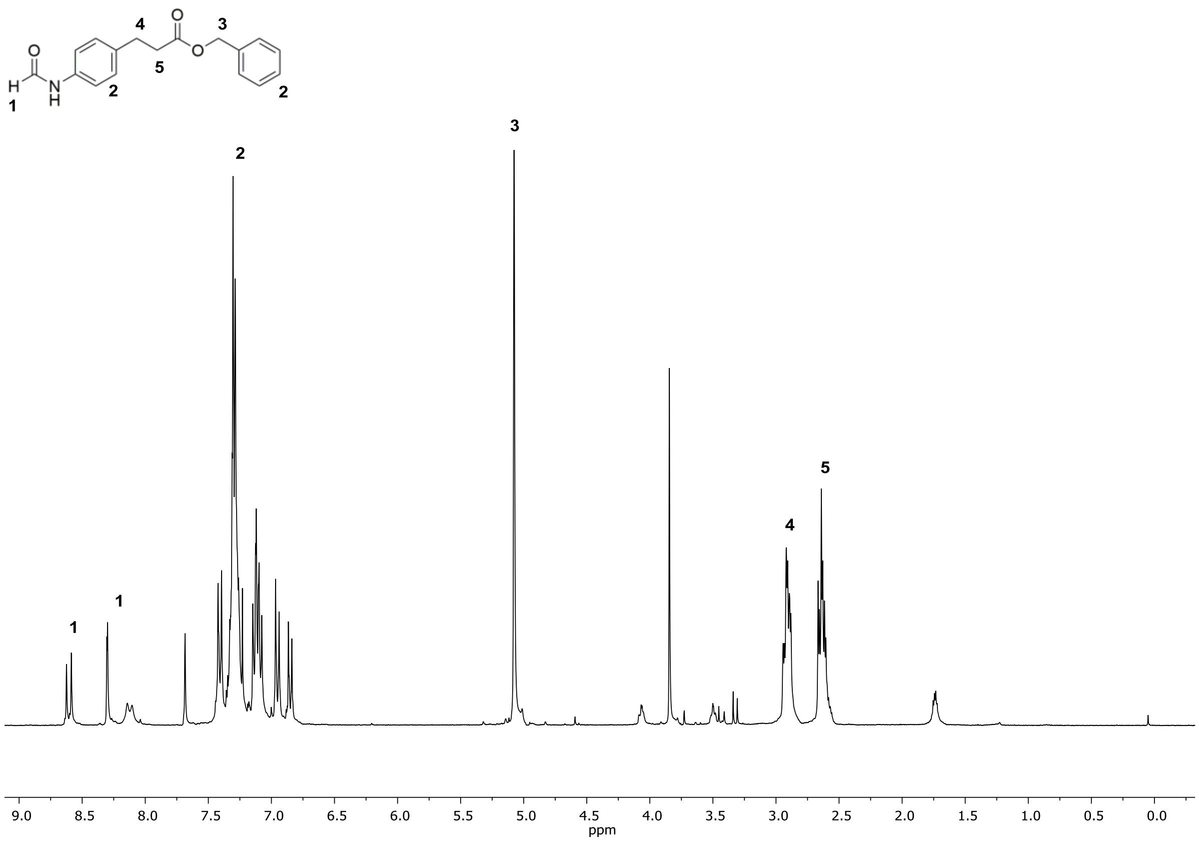

Supplementary Figure 52. Proton NMR of compound ***8g*** *measured in* $CDCl_3$.

Dehydration

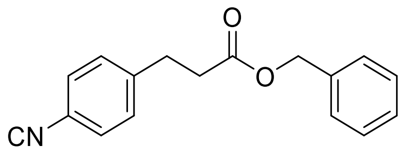

Supplementary Figure 53. Chemical structure of compound ***M7****.*

The formamide **8g** (19.5 g, 68.9 mmol, 1.00 eq.) was dissolved in DCM (210 mL) and diisopropylamine **12** (30.0 mL, 21.6 g, 213 mmol, 3.10 eq.) was added. The solution was cooled to 0 °C with an ice bath. Then, phosphoryl trichloride **9** (8.90 mL, 14.9 g, 89.6 mmol, 1.30 eq.) was added dropwise to the reaction mixture. The yellow solution was stirred for two hours at room temperature and was cooled to 0 °C again. The reaction was quenched by the addition of a 20 wt% solution of sodium carbonate (150 mL) and stirred for another 30 minutes at room temperature. DCM (150 mL) and water (150 mL) were added to the mixture and the organic layer was separated. The organic layer was washed with water (3 × 150 mL) and brine (150 mL), dried over sodium sulfate and the solvent was evaporated under reduced pressure. The crude product was purified by column chromatography (cyclohexane/ ethyl acetate 10:1 → 3:1). Monomer **M7** (10.1 g, 38.1 mmol) was obtained as a green oil in a yield of 55%.

^{1}H-NMR (300 MHz, $CDCl_3$) δ/ppm: 7.31 – 7.08 (m, 9H, CH aromatic, [1]), 5.03 (s, 2H, CH_2, [2]), 2.91 (t, J = 7.5 Hz, 2H, CH_2, [3]), 2.60 (t, J = 7.5 Hz, 2H, CH_2, [4]).

^{13}C-NMR (126 MHz, $CDCl_3$) δ/ppm: 172.11, 142.12, 135.72, 129.38, 128.58, 128.35, 128.31, 126.45, 66.43, 35.34, 30.53.

HRMS-FAB-MS of $[C_{17}H_{16}O_2N]^+$ calculated: 266.1176 found: 266.1182.

IR (ATR platinum diamond): ν/cm^{-1} = 3065.5, 3034.5, 2949.3, 2122.9, 1730.9, 1606.7, 1505.6, 1454.4, 1418.7, 1382.8, 1353.3, 1289.5, 1150.6, 1105.3, 1019.7, 976.3, 909.9, 827.5, 736.0, 696.6, 578.6, 533.9, 512.6, 484.2, 461.6.

***R*$_f$**: (cyclohexane/ethyl acetate 4:1) = 0.56.

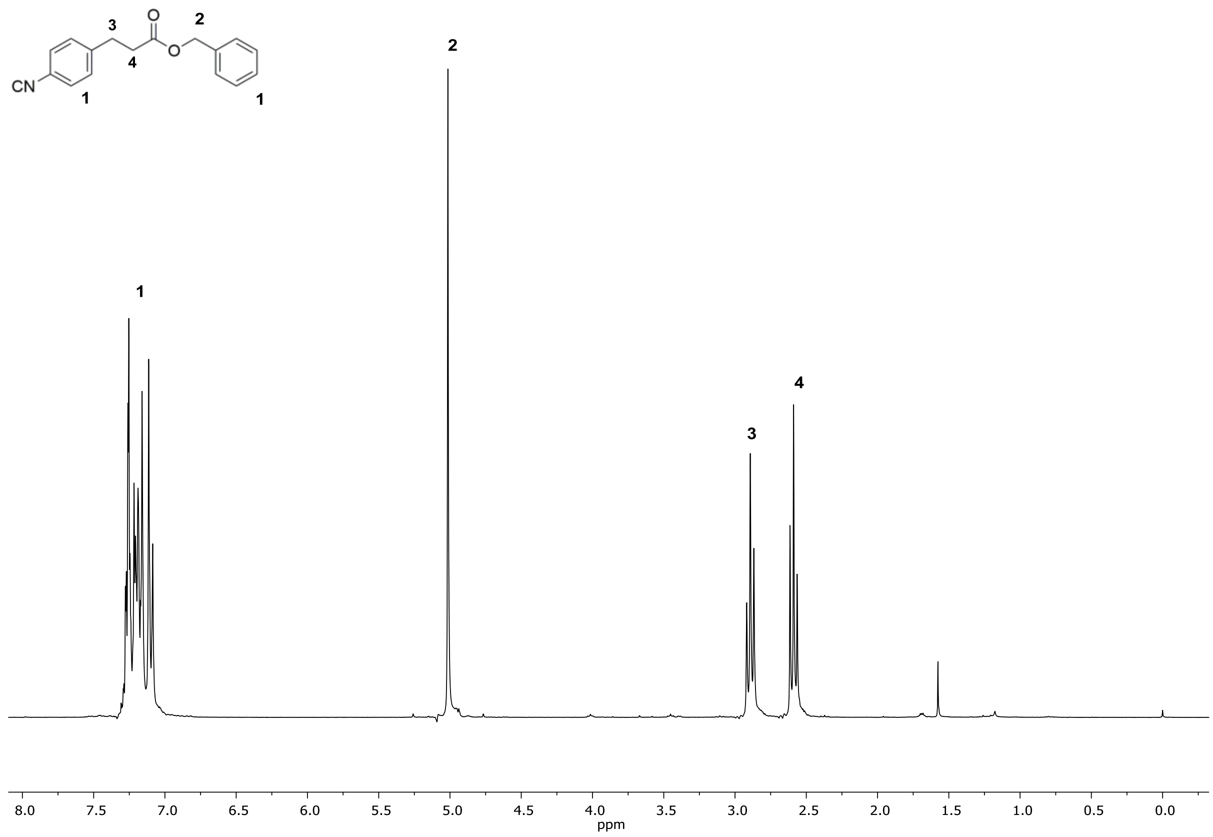

*Supplementary Figure 54. Proton NMR of compound **M7** measured in $CDCl_3$.*

1.3.1.11 Evaluation of the reactivity of monomer M7

Passerini reaction

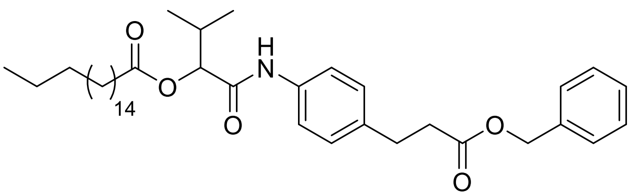

*Supplementary Figure 55. Chemical structure of compound **21**.*

Stearic acid **1** (0.210 g, 0.742 mmol, 1.00 eq.) was suspended in 1.10 mL DCM. Subsequently, monomer **M7** (0.291 g, 1.11 mmol, 1.50 eq.) and isobutyraldehyde **2a** (100 µL, 80.0 mg, 1.11 mmol, 1.50 eq.) were added. The yellow reaction mixture was stirred at room temperature for 24 hours. Afterwards, the solvent was removed under reduced pressure and the crude product was purified by column chromatography (cyclohexane/ethyl acetate 15:1→8:1) to obtain the desired Passerini product **21** as white solid in a yield of 48% of (0.222 g, 0.356 mmol). At the same time, the monomer **M7** from the other fraction was partially recovered (0.066 g, 0.34 eq.) and can be reused.

^{1}**H-NMR** (300 MHz, $CDCl_3$) δ/ppm: 7.73 (s, 1H, NH, ^{1}), 7.42 (d, J = 8.4 Hz, 2H, CH aromatic, ^{2}), 7.36 – 7.28 (m, 5H, CH aromatic, ^{3}), 7.14 (d, J = 8.4 Hz, 2H, CH aromatic, ^{4}), 5.17 (d, J = 4.7 Hz, 1H, CH, ^{5}), 5.10 (s, 2H, CH_2, ^{6}), 2.93 (t, J = 7.7 Hz, 2H, CH_2, ^{7}), 2.65 (t, J = 7.7 Hz, 2H, CH_2, ^{8}), 2.47 (t, J = 7.5 Hz, 2H, CH_2, ^{9}), 1.72 – 1.63 (m, 2H, CH_2, ^{10}), 2.48 - 1.21 (m, 28H, CH_2, ^{11}), 1.00 (d, J = 6.9 Hz, 6H, CH_3, ^{12}), 0.94 – 0.79 (m, 3H, CH_3, ^{13}).

^{13}**C-NMR** (101 MHz, $CDCl_3$) δ/ppm: 173.5, 168.5, 137.8, 136.8, 136.1, 129.4, 121.2, 78.9, 67.22, 36.8, 35.2, 32.8, 31.7, 31.3, 30.6, 30.3, 30.1, 26.0, 23.6, 19.7, 18.1, 15.1.

HRMS-FAB-MS of $[C_{39}H_{60}O_5N]^+$calculated: 622.4466, found: 622.4470.

IR (ATR platinum diamond): ν/cm^{-1} = 2916.9, 2848.6, 1738.2, 1671.6, 1535.3, 1516.7, 1470.8, 1414.0, 1388.5, 1295.8, 1252.1, 1166.3, 1019.4, 850.0, 803.9, 734.5, 719.1, 577.4, 534.1.

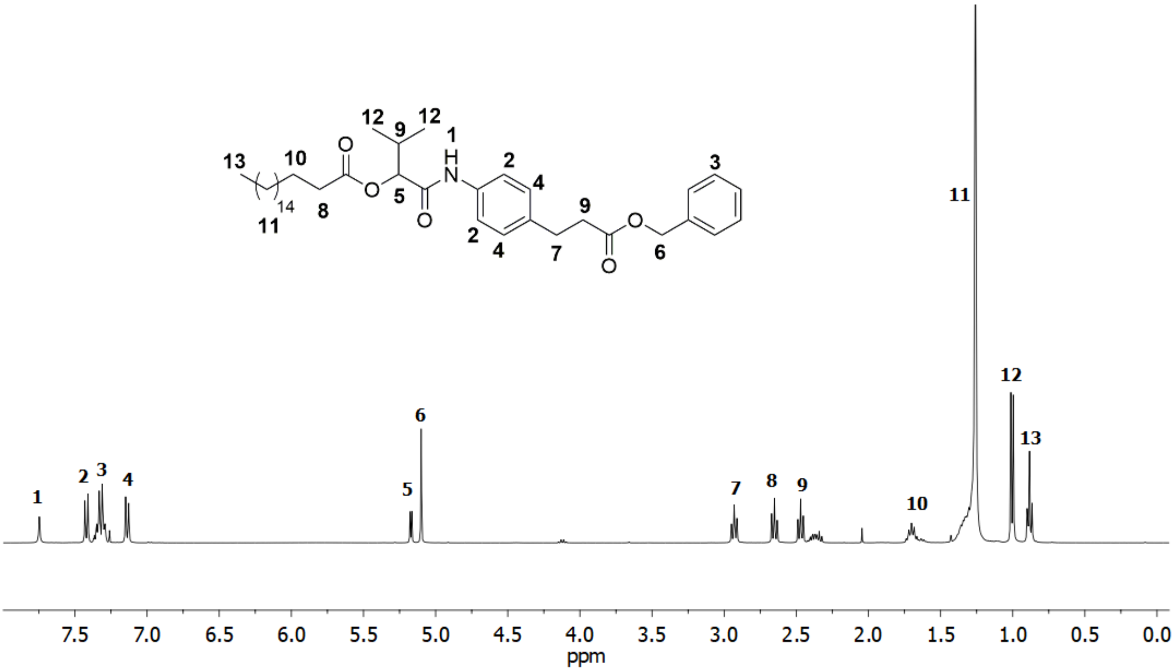

*Supplementary Figure 56. Proton NMR of compound **21** measured in $CDCl_3$.*

Deprotection

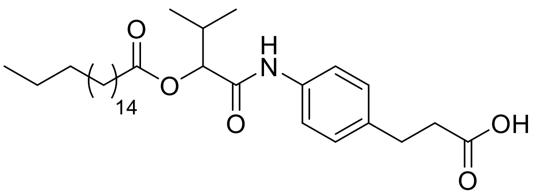

*Supplementary Figure 57. Chemical structure of compound **22**.*

The Passerini product **21** (110 mg, 0.180 mmol, 1.00 eq.) was dissolved in ethyl acetate (0.36 ml, 0.50 M) and palladium on activated charcoal **13** (11.0 mg, 10 wt%) was added. The reaction mixture was purged with hydrogen by using a balloon and stirred under hydrogen atmosphere overnight at room temperature. Afterwards, the heterogeneous catalyst was filtered off and the solvent was evaporated under reduced pressure. The desired deprotected product **22** (85.1 mg, 0.162 mmol) was obtained as white solid in a yield of 90%.

^{1}H-NMR (300 MHz, $CDCl_3$) δ/ppm: 7.74 (s, 1H, NH, [1]), 7.36 (d, J = 8.4 Hz, 2H, aromatic, [2]), 7.09 (d, J = 8.4 Hz, 2H, aromatic, [3]), 5.09 (d, J = 4.8 Hz, 1H, CH, [4]), 2.84 (t, J = 7.6 Hz, 2H, CH_2, [5]), 2.57 (t, J = 7.6 Hz, 2H, CH_2, [6]), 2.39 (t, J = 7.5 Hz, 2H, CH_2, [7]), 1.64 – 1.57 (m, 2H, CH_2, [8]), 1.47 - 1.10 (m, 28H, CH_2, [9]), 0.93 (d, J = 6.8 Hz, 6H, CH_3, [10]), 0.85 – 0.76 (m, 3H, CH_3, [11]).

^{13}C-NMR (101 MHz, $CDCl_3$) δ/ppm: 177.5, 171.8, 166.7, 135.7, 134.2, 128.1, 127.8, 119.4, 78.9, 67.2, 34.6, 33.1, 29.8, 28.7, 28.3, 28.1, 24.0, 23.7, 21.9, 21.7.

HRMS-FAB-MS of $[C_{32}H_{54}O_5N]^+$calculated: 532.3997, found: 532.4003.

IR (ATR platinum diamond): ν/cm^{-1} = 2917.2, 2848.8, 1737.2, 1708.9, 1667.6, 1603.6, 1530.9, 1469.8, 1414.3, 1297.1, 1255.2, 1169.5, 1018.7, 948.0, 829.3, 808.9, 719.6, 662.9, 547.7, 511.9, 416.9.

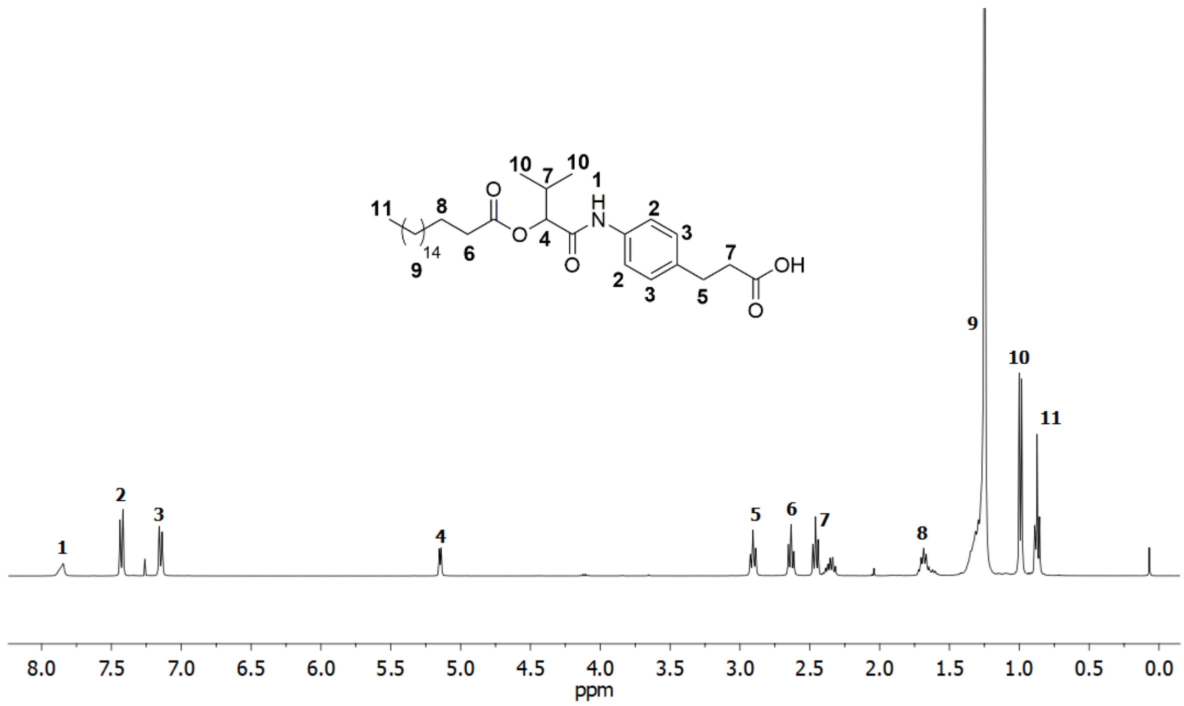

*Supplementary Figure 58. Proton NMR of compound **21** measured in $CDCl_3$.*

1.3.1.12 Synthesis of monomer M8

Esterification

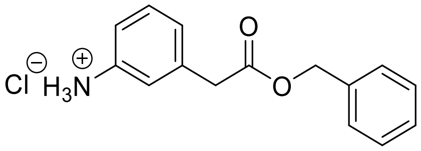

*Supplementary Figure 59. Chemical structure of compound **6h**.*

3-Aminophenylacetic acid **3h** (10.0 g, 66.4 mmol, 1.00 eq.) was suspended in THF (70 mL). After the addition of benzyl alcohol **4** (75.6 mL, 79.0 g, 731 mmol, 11.0 eq.), the suspension was cooled in an ice bath to 0 °C and thionyl chloride **5** (17.0 mL, 28.0 g, 235 mmol, 3.54 eq.) was added dropwise. Afterwards, the suspension was stirred at room temperature for 20 hours. Subsequently, 500 mL diethyl ether were added, and the solution was stored in the freezer for two hours. The precipitate was filtered off and the crude product **6h** (10.4 g, 37.4 mmol) was obtained as a slightly brown solid in a yield of 56%.

^{1}H-NMR (300 MHz, CD_3OD) δ/ppm: 7.54 – 7.14 (m, 9H, CH aromatic, ^{1}), 5.08 (s, 2H, CH_2, ^{2}), 3.76 (s, 2H, CH_2, ^{3}).

^{13}C-NMR (101 MHz, CD_3OD) δ/ppm: 173.06, 142.63, 138.28, 131.98, 131.33, 131.29, 129.54, 129.32, 129.18, 128.24, 127.97, 125.17, 122.76, 65.17, 41.00.

HRMS-FAB-MS of $[C_{15}H_{16}O_2N]^+$ calculated: 242.1176 found: 242.1181.

IR (ATR platinum diamond): ν/cm^{-1} = 3031.5, 2964.2, 2900.3, 2714.5, 2629.1, 2546.8, 2236.5, 1904.7, 1722.3, 1603.3, 1571.4, 1492.9, 1455.8, 1420.5, 1377.8, 1335.7, 1300.4, 1250.5, 1213.4, 1190.5, 1166.9, 1150.1, 1108.4, 1064.1, 1030.7, 992.5, 972.6, 947.5, 907.7, 794.3, 761.1, 741.5, 717.6, 694.4, 683.9, 600.0, 576.9, 530.9, 519.6, 484.9, 443.3.

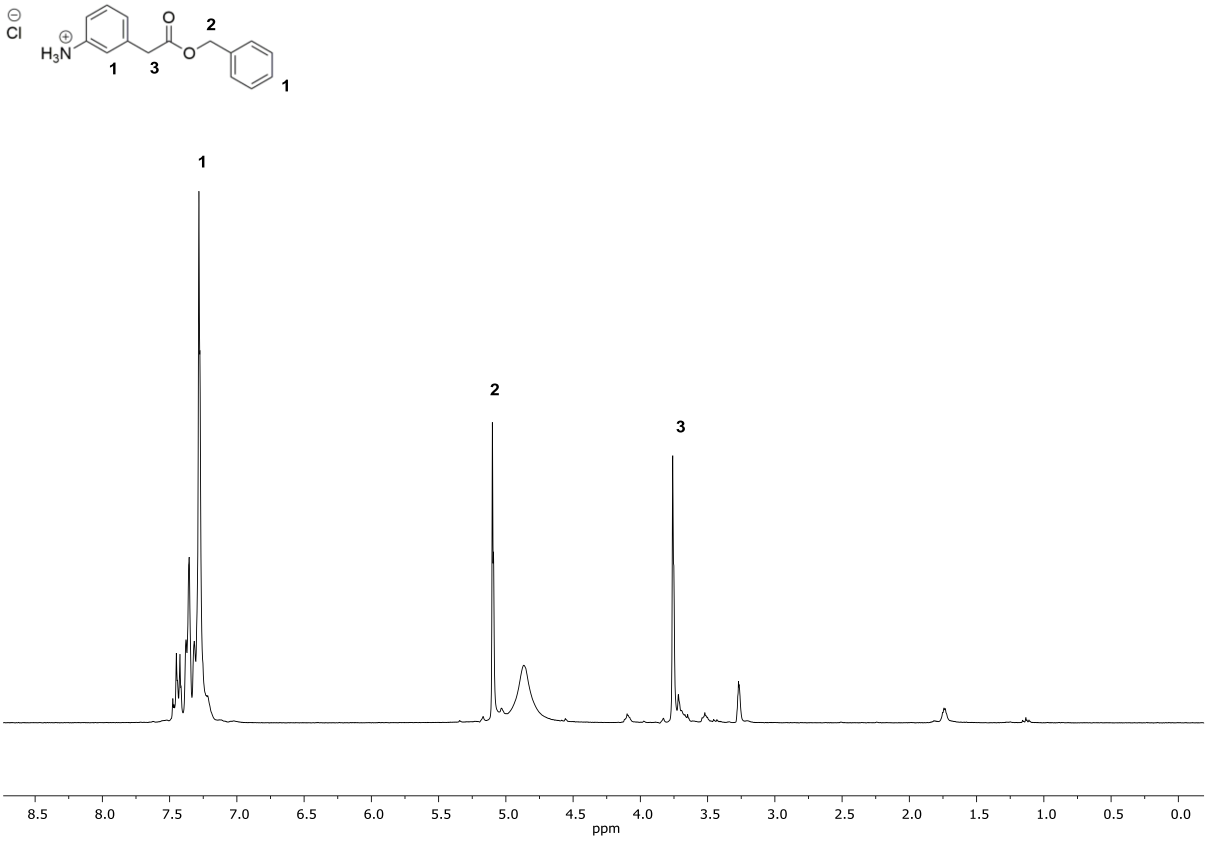

*Supplementary Figure 60. Proton NMR of compound **6h** measured in CD_3OD.*

N-Formylation

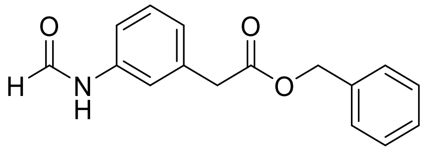

*Supplementary Figure 61. Chemical structure of compound **8h**.*

The ammonium salt **6h** (10.3 g, 37.0 mmol, 1.00 eq.) was stirred with trimethyl orthoformate **7** (40.5 mL, 39.3 g, 370 mmol, 10.0 eq.) under reflux at 105 °C overnight. Subsequently, the orthoformate **7** was evaporated under reduced pressure. The crude product **8h** (9.96 g, 37.0 mmol) was obtained as a yellow oil in quantitative yield and was used without further purification.

^{1}H-NMR (300 MHz, $CDCl_3$) δ/ppm: 8.56 (d, J = 11.4 Hz, 0.5H, CH, ^{1}), 8.18 (s, 0.5H, CH, ^{1}), 7.70 – 6.72 (m, 9H, CH aromatic, ^{2}), 5.06 (s, 2H, CH_2, ^{3}), 3.57 (s, 2H, CH_2, ^{4}).

^{13}C-NMR (101 MHz, $CDCl_3$) δ/ppm: 171.46, 162.82, 135.80, 135.70, 134.84, 129.34, 128.63, 128.34, 128.25, 126.20, 122.51, 120.85, 66.79.

HRMS-FAB-MS of $[C_{16}H_{16}O_3N]^+$ calculated: 270.1125 found: 270.1132.

IR (ATR platinum diamond): ν/cm^{-1} = 3318.4, 3146.2, 3031.9, 2944.3, 2891.9, 1729.4, 1688.6, 1644.5, 1595.2, 1546.7, 1491.5, 1441.6, 1376.7, 1262.6, 1211.7, 1144.6, 999.8, 894.2, 785.8, 735.8, 694.8, 580.7, 499.2.

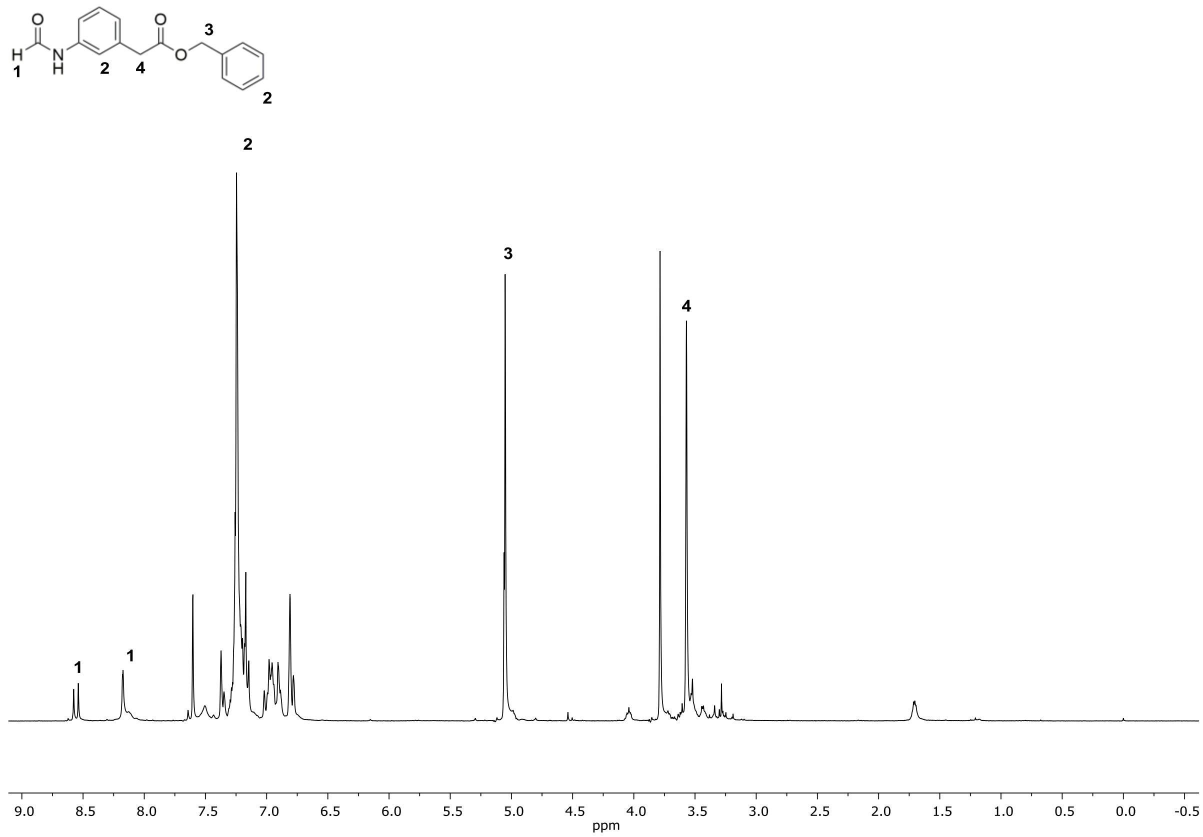

*Supplementary Figure 62. Proton NMR of compound **8h** measured in $CDCl_3$.*

Dehydration

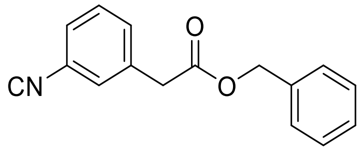

*Supplementary Figure 63. Chemical structure of compound **M8**.*

The formamide **8h** (10.7 g, 39.7 mmol, 1.00 eq.) was dissolved in DCM (120 mL) and diisopropylamine **12** (14.1 mL, 19.5 g, 139 mmol, 3.50 eq.) was added. The solution was cooled to 0 °C in an ice bath. Then, phosphoryl trichloride **9** (5.10 mL, 8.60 g, 51.6 mmol, 1.30 eq.) was added dropwise to the reaction mixture. The yellow solution was stirred for two hours at room temperature and was afterwards cooled to 0 °C again. The reaction was quenched by the addition of a 20 wt% sodium carbonate solution (100 mL) and stirred for another 30 minutes at room temperature. 100 mL DCM and 100 mL water were added to the mixture and the organic layer was separated. The organic layer was washed with water (3 × 100 mL) and brine (100 mL), dried over sodium sulfate and the solvent was evaporated under reduced pressure. The crude product was purified by column chromatography (cyclohexane/ethyl acetate 10:1 → 3:1). Monomer **M8** (3.90 g, 15.6 mmol) was obtained as a green oil in a yield of 39%.

^{1}H-NMR (300 MHz, $CDCl_3$) δ/ppm: 7.36 – 7.07 (m, 9H, CH aromatic, ^{1}), 5.06 (s, 2H, CH_2, ^{2}), 3.58 (s, 2H, CH_2, ^{3}).

^{13}C-NMR (101 MHz, $CDCl_3$) δ/ppm: 170.42, 135.67, 135.51, 130.53, 129.64, 128.69, 128.52, 128.35, 127.33, 125.23, 67.06, 40.65.

HRMS-FAB-MS of $[C_{16}H_{14}O_2N]^+$ calculated: 252.1019 found: 252.1024.

IR (ATR platinum diamond): ν/cm^{-1} = 3058.5, 3029.1, 2973.7, 2944.7, 2917.8, 2889.7, 2128.6, 1959.6, 1724.0, 1601.4, 1586.2, 1485.9, 1453.2, 1423.1, 1377.2, 1338.3, 1289.7, 1236.0, 1219.2, 1191.0, 1179.1, 1153.3, 1080.8, 1029.2, 1002.8, 970.0, 940.8, 907.5, 878.9, 829.9, 796.6, 760.8, 740.2, 712.8, 696.4, 681.2, 601.5, 579.3, 553.1, 499.2, 473.7, 449.1.

R_f: (cyclohexane / ethyl acetate 4:1) = 0.47.

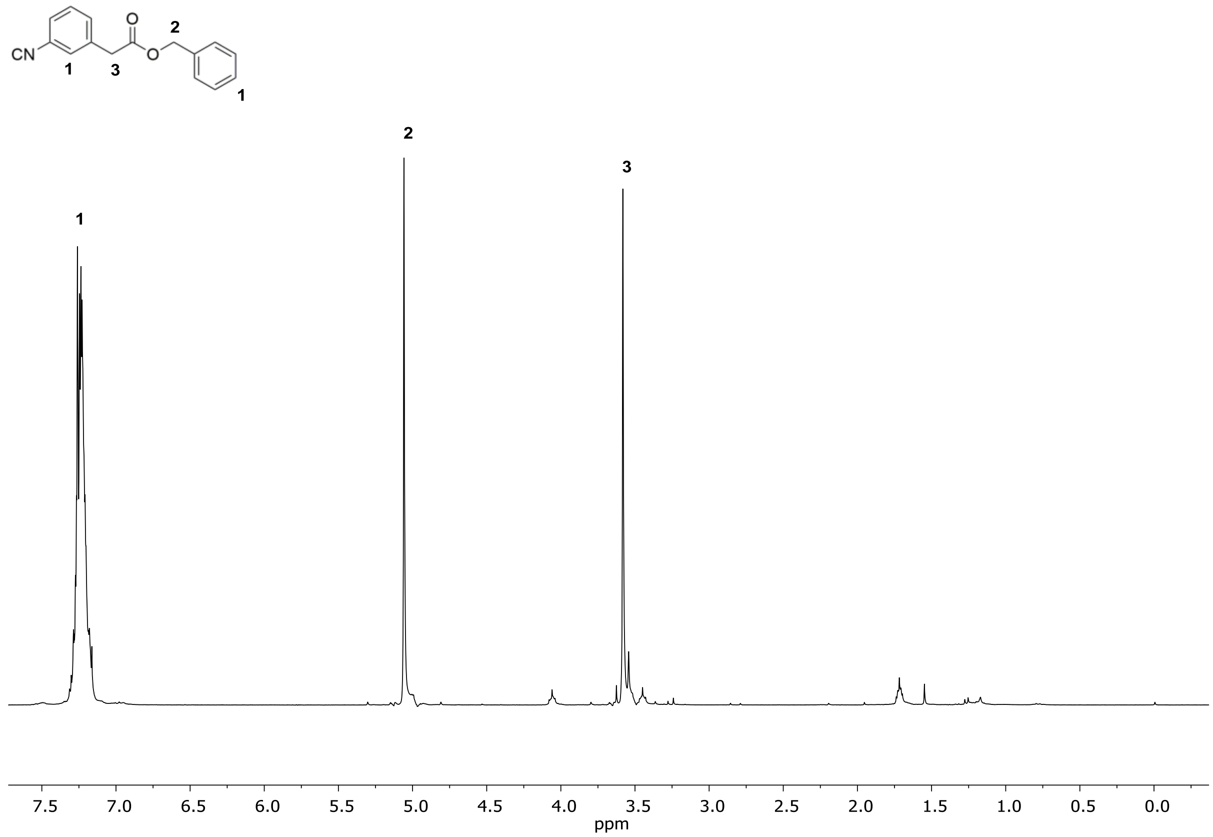

*Supplementary Figure 64. Proton NMR of compound **M8** measured in $CDCl_3$.*

1.3.1.13 Synthesis of monomer M9

Esterification

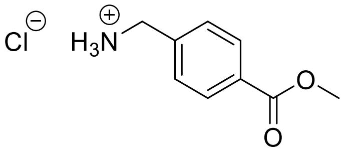

*Supplementary Figure 65. Chemical structure of compound **11**.*

Methanol (74 mL) was added to 4-(aminomethyl)benzoic acid **3i** (5.64 g, 37.0 mmol, 1.00 eq.). Then, the suspension was cooled in an ice bath to 0 °C and thionyl chloride **5** (9.53 mL, 15.5 g, 131 mmol, 3.50 eq.) was added dropwise. Afterwards, the solution was stirred at room temperature for 26 hours. Subsequently, diethyl ether (150 mL) was added and the solution was stored in the freezer overnight. The white precipitate was filtered off and washed with diethyl ether (*ca.* 10 mL). The mother liquor was stored in the freezer once again and the precipitate was filtered off and washed with diethyl ether (*ca.* 10 mL). The desired product **11** (7.10 g, 32.5 mmol) was obtained as a white solid in a yield of 88%.

^{1}H-NMR (300 MHz, CD_3OD) δ/ppm: 8.08 (d, J = 8.4 Hz, 2H, CH aromatic, [1]), 7.56 (d, J = 8.1 Hz, 2H, aromatic, [2]), 4.19 (s, 3H, CH_3, [3]), 3.91 (s, 2H, CH_2, [4]).

^{13}C-NMR (75 MHz, CD_3OD) δ/ppm: 158.3, 139.5, 134.2, 131.2, 130.1, 52.8, 43.9.

HRMS-FAB-MS of $[C_9H_{12}O_2N]^+$ calculated: 166.0863, found: 166.0863.

IR (ATR platinum diamond): ν/cm^{-1} = 3007.7, 2959.9, 2877.6, 2752.1, 2686.2, 2573.2, 1719.6, 1596.1, 1577.6, 1476.8, 1464.5, 1435.7, 1380.2, 1281.4, 1188.9, 1110.7, 1075.8, 1024.3, 960.6, 878.3, 863.9, 835.1, 787.8, 763.1, 701.4, 623.2, 530.7, 475.1.

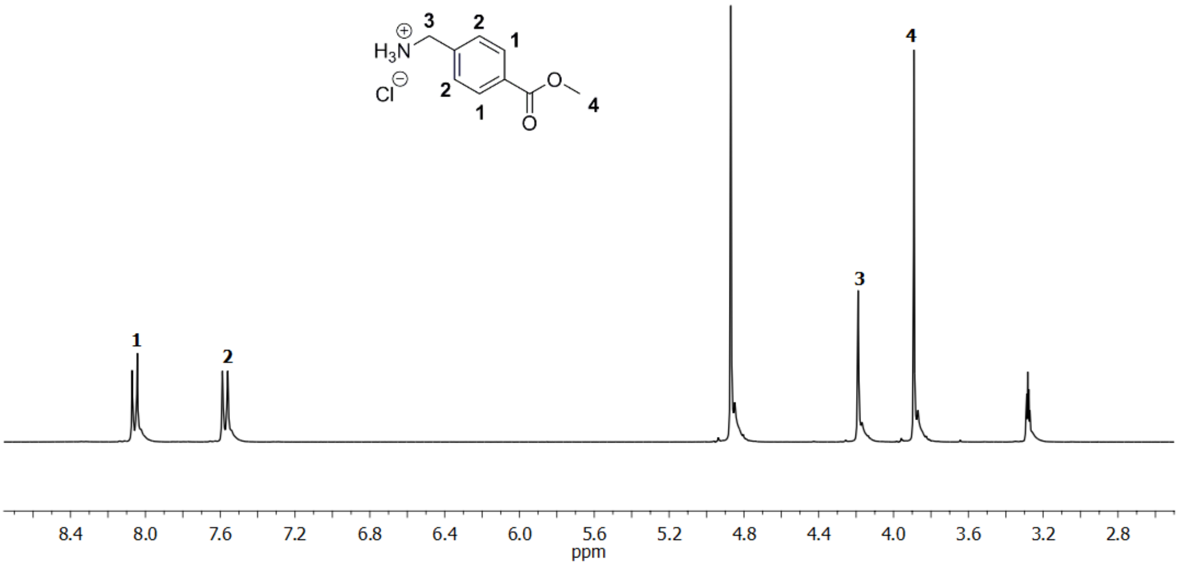

*Supplementary Figure 66. Proton NMR of compound **11** measured in CD_3OD.*

N-Formylation

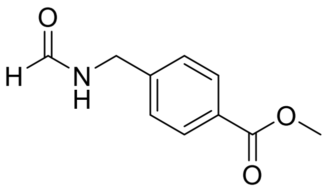

*Supplementary Figure 67. Chemical structure of compound **23**.*

The ammonium salt **11** (7.02 g, 34.8 mmol, 1.00 eq.) was stirred with trimethyl orthoformate **7** (37.7 ml, 36.9 g, 348 mmol, 10.0 eq.) under reflux at 100 °C overnight. Subsequently, the excess of orthoformate **7** was evaporated under reduced pressure to obtain needle-like white crystals. The crude product **23** (6.28 g, 32.5 mmol) was obtained in a yield of 93% and was used without further purification.

^{1}H-NMR (300 MHz, CD_3OD) δ/ppm: 8.25 – 8.18 (m, 1H, CH, [1]), 8.07 – 7.90 (d, J = 8.4 Hz, 2H, CH aromatic, [2]), 7.49 – 7.32 (d, J = 8.1 Hz, 2H, aromatic, [3]), 4.47 (s, 2H, CH_2, [4]), 3.89 (s, 3H, CH_3, [5]).

^{13}C-NMR (75 MHz, CD_3OD) δ/ppm: 163.78, 145.08, 130.76, 130.26, 128.77, 128.51, 52.58, 42.27.

HRMS-EI-MS of $[C_{10}H_{11}O_3N]^{\cdot+}$ calculated: 193.0733, found: 193.0736.

IR (ATR platinum diamond): ν/cm^{-1} = 3268.4, 3198.5, 3044.2, 3019.5, 2959.9, 2885.9, 2859.1, 2766.5, 1719.6, 1653.7, 1629.1, 1610.5, 1538.6, 1456.3, 1448.0, 1429.5, 1413.1, 1392.5, 1347.2, 1330.8, 1310.2, 1275.3, 1236.2, 1217.7, 1193.0, 1174.5, 1100.4, 1018.2, 952.3, 843.3, 765.2, 750.8, 724.0, 701.4, 514.2, 489.5.

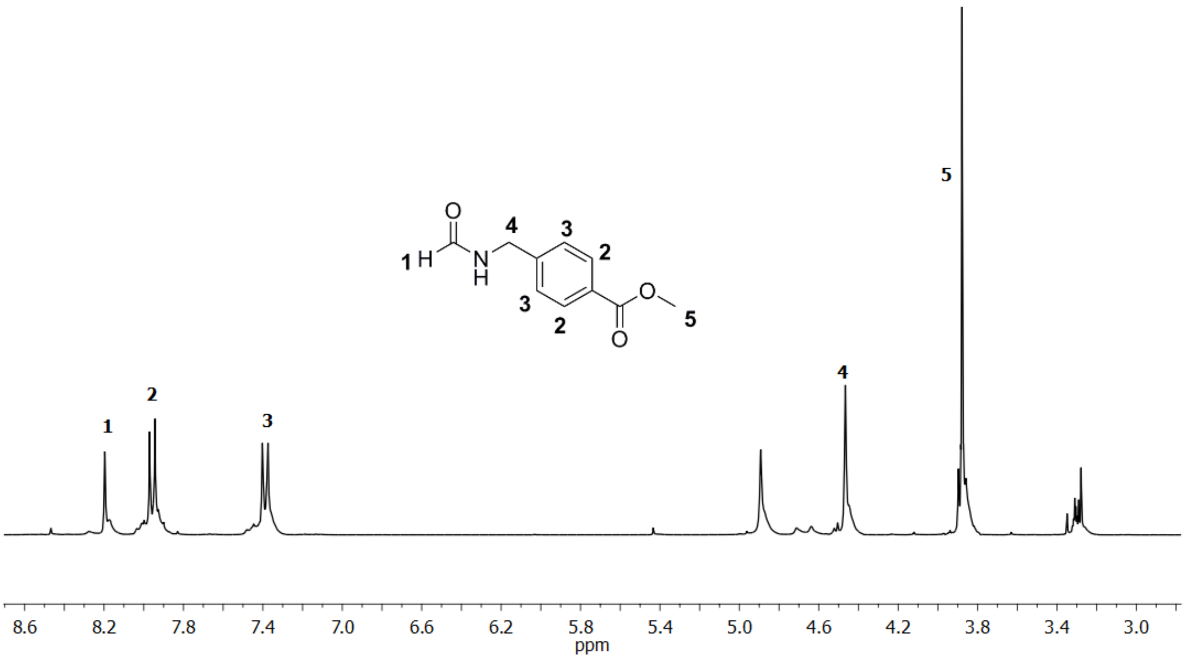

*Supplementary Figure 68. Proton NMR of compound **23** measured in CD_3OD.*

Transesterification

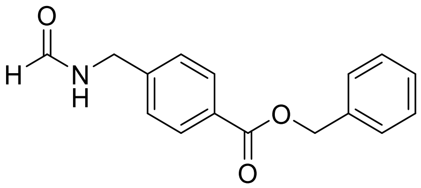

Supplementary Figure 69. Chemical structure of compound ***8i****.*

The methyl ester **23** (6.17 g, 31.9 mmol, 1.00 eq.) was dissolved in benzyl alcohol **4** (13.3 mL, 13.8 g, 128 mmol, 4.00 eq.) and DBU **14** (971 mg, 6.38 mmol, 20 mol%) was added. Subsequently, the colourless solution was heated to 150 °C and stirred for 22 hours. Then, the brown solution was purified by column chromatography (cyclohexane/ethyl acetate 7:1 → 0:1). The benzyl ester **8i** (3.74 g, 13.9 mmol) was obtained as a yellowish solid in a yield of 44%.

^{1}H-NMR (300 MHz, CD_3OD) δ/ppm: 8.16 – 8.12 (m, 1H, CH, [1]), 8.00 – 7.98 (m, 2H, CH aromatic, [2]), 7.44 – 7.28 (m, 7H, CH aromatic, [3]), 5.41 – 5.22 (m, 2H, CH_2, [4]), 4.52 – 4.29 (m, 2H, CH_2, [5]).

^{13}C-NMR (75 MHz, CD_3OD) δ/ppm: 163.8, 130.89, 129.6, 129.2, 128.6, 115.5, 107.1, 94.8, 92.3, 67.8, 42.3, 33.0.

HRMS-FAB-MS of $[C_{16}H_{16}O_3N]^+$ calculated: 270.1125, found: 270.1132.

IR (ATR platinum diamond): ν/cm^{-1} = 3266.3, 3031.9, 2955.7, 2887.9, 1715.4, 1649.6, 1627.0, 1612.6, 1530.3, 1495.4, 1448.0, 1417.2, 1384.3, 1365.8, 1267.0, 1224.8, 1176.5, 1094.3, 1010.2, 940.0, 915.3, 849.5, 822.8, 754.9, 693.2, 625.3, 596.5, 512.2.

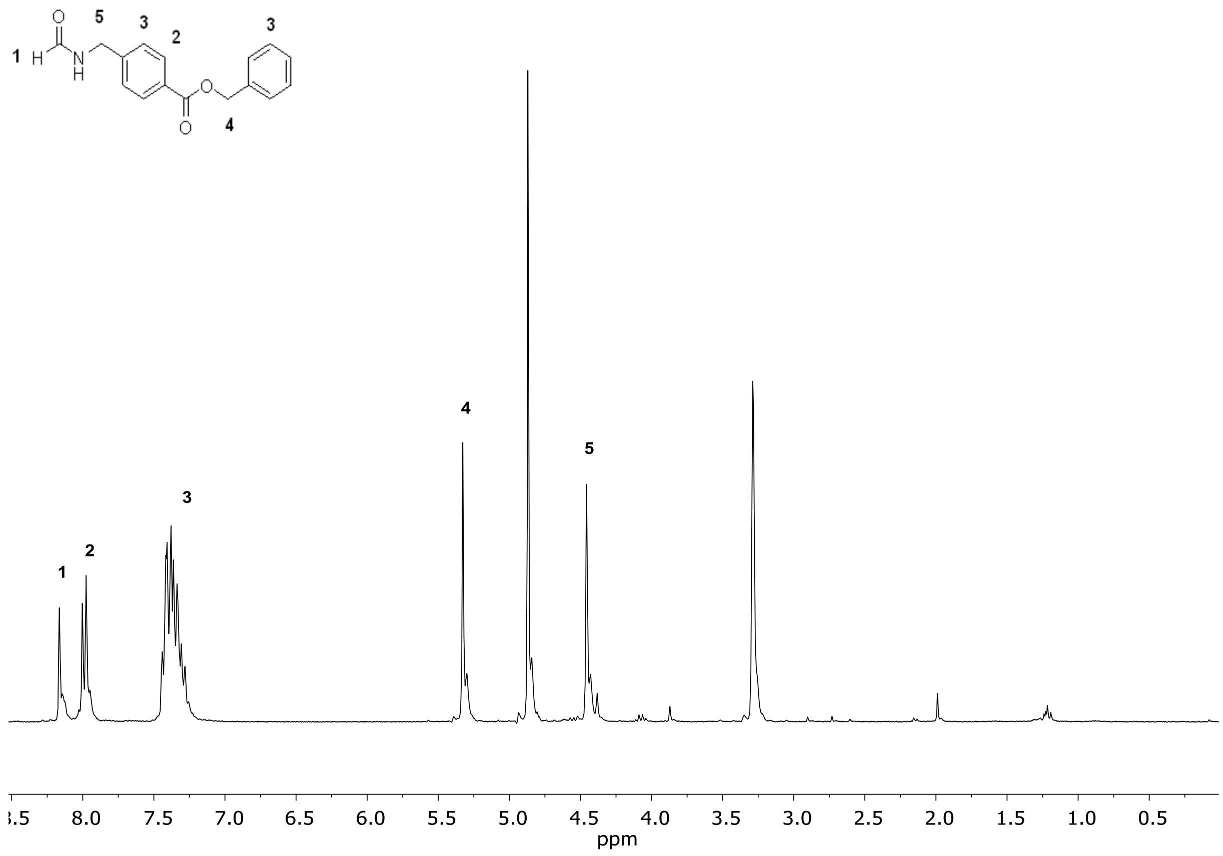

*Supplementary Figure 70. Proton NMR of compound **8i** measured in CD_3OD.*

Dehydration

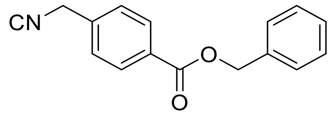

Supplementary Figure 71. Chemical structure of compound ***M9****.*

The formamide **8i** (3.74 g, 13.9 mmol, 1.00 eq.) was dissolved in DCM (47 mL) and diisopropylamine **12** (6.06 mL, 4.36 g, 43.1 mmol, 3.10 eq.) was added. The slightly yellow solution was cooled to 0 °C using an ice bath. Then, phosphoryl trichloride **9** (1.65 mL, 2.78 g, 18.1 mmol, 1.30 eq.) was added dropwise to the reaction mixture. The reaction mixture was stirred for two hours at room temperature and was cooled to 0 °C again. The reaction was quenched by the addition of a 20 wt% solution of sodium carbonate (17 mL) and stirred for another 30 minutes at room temperature. 15 mL DCM and 15 mL water were added to the mixture and the organic layer was separated. The aqueous phase was extracted with DCM (10 mL). The combined organic layers were washed with water (2x 35 mL) and brine (20 mL), dried over sodium sulfate and the solvent was evaporated under reduced pressure. The crude product was purified by column chromatography (cyclohexane/ethyl acetate 12:1 → 0:1). The isocyanide monomer **M9** (2.66 g, 10.6 mmol) was obtained as an amber solid in a yield of 76%.

^{1}H-NMR (300 MHz, CD_3OD) δ/ppm: 8.06 (d, *J* = 8.4 Hz, 2H, CH aromatic, [1]), 7.50 – 7.31 (m, 7H, CH aromatic, [2]), 5.34 (s, 2H, CH_2, [3]), 4.84 (s, 2H, CH_2, [4]).

^{13}C-NMR (75 MHz, CD_3OD) δ/ppm: 165.8, 163.0, 158.8, 150.7, 137.2, 135.9, 130.4, 128.7, 128.3, 126.6, 67.0, 45.3.

HRMS-FAB-MS of $[C_{16}H_{14}O_2N]^+$ calculated: 252.1019, found: 252.1026.

IR (ATR platinum diamond): ν/cm^{-1} = 3052.4, 2935.8, 2149.4, 1713.4, 1612.6, 1495.4, 1456.3, 1427.4, 1417.2, 1380.2, 1320.5, 1273.2, 1178.6, 1104.5, 1016.1, 977.0, 950.3, 915.3, 839.2, 828.9, 734.3, 699.3, 602.7, 508.1, 479.3, 452.5.

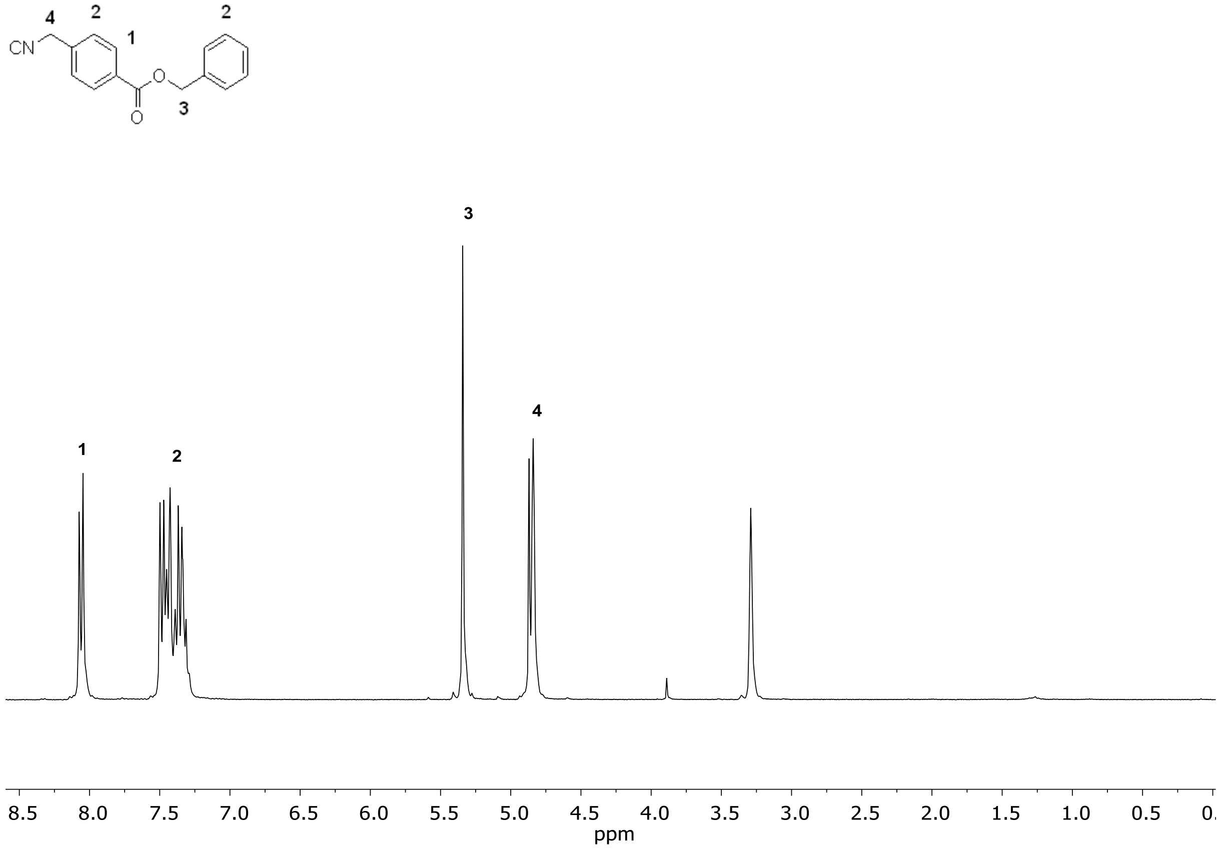

Supplementary Figure 72. Proton NMR of compound ***M9*** *measured in CD_3OD.*

1.3.1.14 Evaluation of the reactivity of monomer M9

Passerini reaction

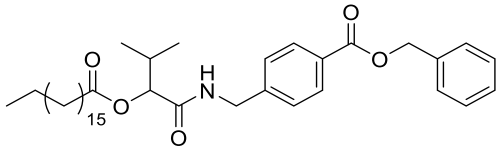

*Supplementary Figure 73. Chemical structure of compound **24**.*

Stearic acid **1** (75.5 mg, 265 µmol, 1.00 eq.) was suspended in DCM (1.15 ml). Subsequently, monomer **M9** (100 mg, 398 µmol, 1.50 eq.) and isobutyraldehyde **2a** (36.0 µL, 28.7 mg, 398 µmol, 1.50 eq.) were added. The reaction mixture was stirred for 48 hours at room temperature, the solvent was evaporated under reduced pressure and the crude product was purified by column chromatography (cyclohexane/ethyl acetate 17:1 → 2:1). The Passerini product **24** (108 mg, 177 mmol) was obtained as a white solid in a yield of 45%.

^{1}H-NMR (300 MHz, $CDCl_3$) δ/ppm: 8.04 (d, J = 8.2 Hz, 2H, CH aromatic, [1]), 7.47 – 7.29 (m, 7H, CH aromatic, [2]), 6.29 (t, J = 5.7 Hz, 1H, NH, [3]), 5.36 (s, 2H, CH_2, [4]), 5.13 (d, J = 4.4 Hz, 1H, CH, [5]), 4.62 – 4.45 (m, 2H, CH_2, [6]), 2.41 – 2.28 (m, 3H, CH_2, CH, [7]), 1.69 – 1.57 (m, 2H, CH_2, [8]), 1.36 – 1.16 (m, 28H, CH_2, [9]), 0.95(dd, J = 6.8 Hz, J = 3.8 Hz, 6H, CH_3, [10]), 0.88 (t, J = 6.6 Hz, 3H, CH_3, [11]).

^{13}C-NMR (75 MHz, $CDCl_3$) δ/ppm: 172.8, 169.7, 157.1, 143.4, 138.6, 130.3, 128.8, 128.4, 128.3, 127.6, 66.9, 42.9, 39.9, 34.4, 32.1, 30.8, 29.8, 29.7, 29.6, 29.5, 29.4, 29.3, 25.2, 22.8, 18.9, 17.2, 14.3.

HRMS-FAB-MS of $[C_{38}H_{58}O_5N]^+$ calculated: 608.4310, found: 608.4308.

IR (ATR platinum diamond): ν /cm-1 = 3280.7, 2914.6, 2848.8, 1719.6, 1655.8, 1552.95, 1468.6, 1376.1, 1269.1, 1234.1, 1174.5, 1098.4, 1020.2, 756.9, 717.9, 695.2.

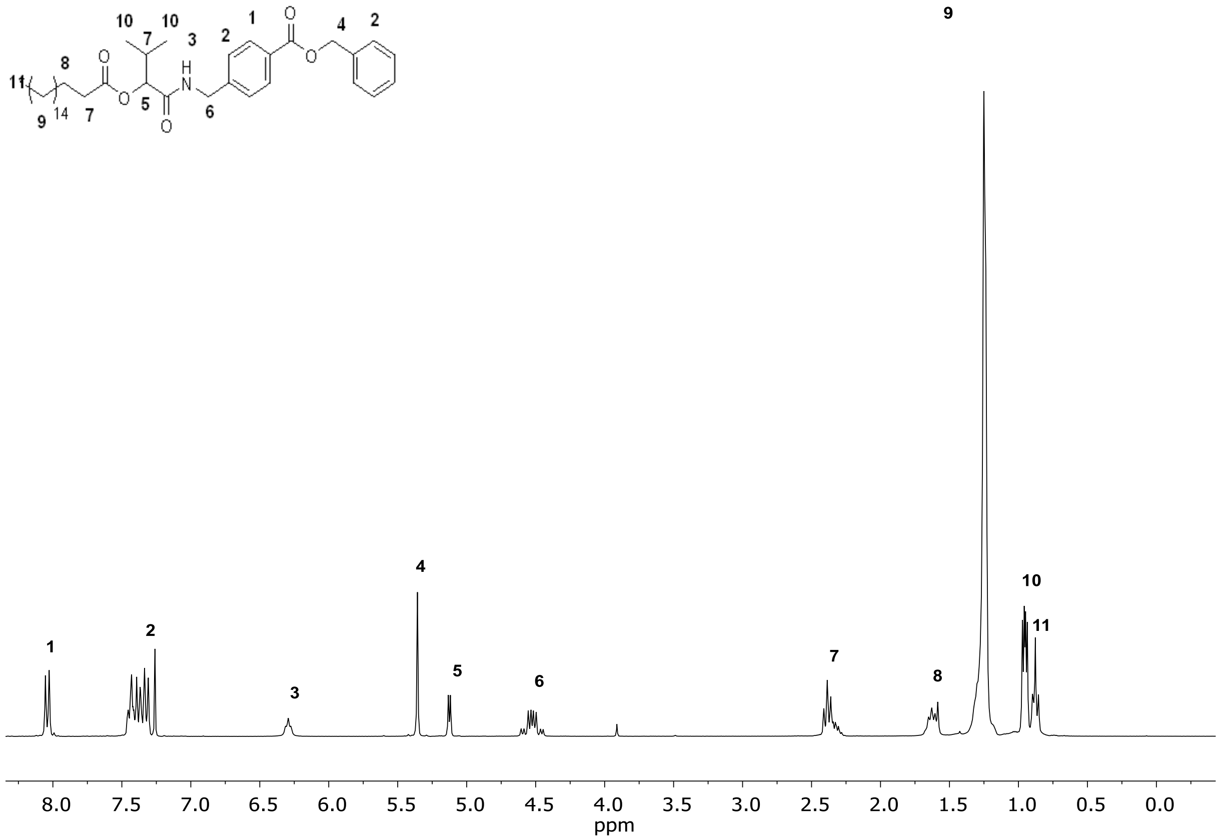

*Supplementary Figure 74. Proton NMR of compound **24** measured in $CDCl_3$.*

Deprotection

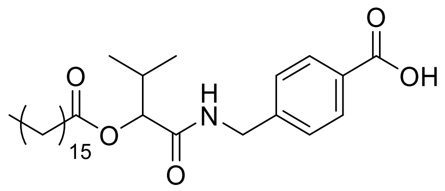

*Supplementary Figure 75. Chemical structure of compound **25**.*

The Passerini product **24** (81.0 mg, 133 µmol, 1.00 eq.) was dissolved in ethyl acetate (2.2 mL) and palladium on activated charcoal **13** (8.10 mg, 10 wt%) was added. Subsequently, the reaction mixture was purged with hydrogen using a balloon and stirred under hydrogen atmosphere for three hours. Afterwards, the heterogeneous catalyst was filtered off and the solvent was evaporated under reduced pressure. The desired deprotected carboxylic acid **25** (34.9 mg, 67.4 µmol) was obtained as a white solid in a yield of 51%.

^{1}H-NMR (300 MHz, $CDCl_3$) δ/ppm: 8.05 (d, J = 8.0 Hz, 2H, CH aromatic, [1]), 7.35 (d, J = 8.1 Hz, 2H, CH aromatic, [2]), 6.42 (t, J = 5.9 Hz, 1H, NH, [3]), 5.13 (d, J = 4.5 Hz, 1H, CH, [4]), 4.62 – 4.47 (m, 2H, CH_2, [5]), 2.42 – 2.27 (m, 3H, CH_2, CH, [6]), 1.71 – 1.56 (m, 2H, CH_2, [7]), 1.37 – 1.15 (m, 28H, CH_2, [8]), 0.96 (dd, J = 6.6 Hz, J = 2.8 Hz, 6H, CH_3, [9]), 0.87 (t, J = 6.3 Hz, 3H CH_3, [10]).

^{13}C-NMR (75 MHz, $CDCl_3$) δ/ppm: 173.0, 171.1, 163.2, 144.1, 130.8, 128.8, 127.7, 78.1, 43.0, 34.4, 32.1, 30.8, 29.8, 29.7, 29.6, 29.5, 29.4, 29.3, 25.1, 22.8, 18.9, 17.2, 14.2.

HRMS-FAB-MS of $[C_{31}H_{52}O_5N]^+$ calculated: 518.3840, found: 518.3848.

IR (ATR platinum diamond): ν /cm^{-1} = 3280.7, 2914.6, 2848.8, 1734.0, 1690.8, 1655.8, 1612.6, 1553.0, 1468.6, 1431.6, 1378.1, 1348.5, 1293.8, 1236.2, 1172.4, 1110.7, 1005.8, 929.7, 763.1, 719.9, 697.3, 547.1.

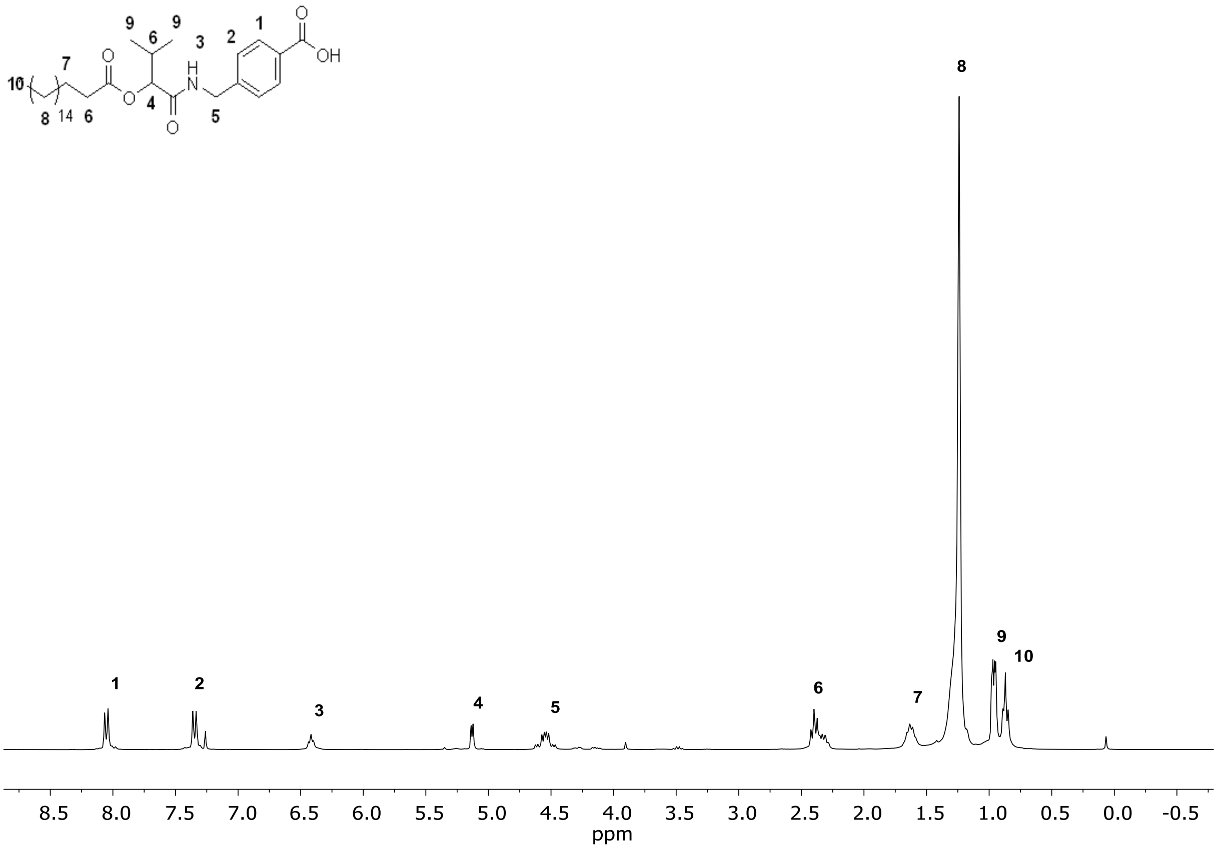

*Supplementary Figure 76. Proton NMR of compound **25** measured in $CDCl_3$.*

1.3.2 Summary of the monomer syntheses

*Supplementary Table 1. Summary of the results of the monomer syntheses of monomers **M1** – **M9** with all their intermediates.*

Monomer	Reaction step	Yield [%]	m/z calc.	m/z found
**Monomer M1**	Esterification	95[a]	292.2271	292.2271
	N-formylation	quant.[b]	320.2220	320.2222
	Dehydration	67[c]	302.2115	302.2113
Overall yield		64		
**Monomer M2**	Esterification	96[a]	222.1489	222.1489
	N-formylation	73[c]	250.1438	250.1437
	Dehydration	74[c]	231.1254	231.1255
Overall yield		52		
**Monomer M3**	Esterification	75[a]	306.2428	306.2431
	N-formylation	quant.[b]	334.2377	334.2384
	Dehydration	32[c]	316.2271	316.2272
Overall yield		24		
**Monomer 4**	Esterification	81[a]	180.1019	180.1020
	N-formylation	52[c]	207.0890	207.0891
	Dehydration	74[c]	189.0790	189.0791
Overall yield		31		
**Monomer M5**	Esterification	89[a]	194.1176	194.1181
	N-formylation	91[b]	221.1152	221.1050
	Dehydration	69[c]	204.1019	204.1024
Overall yield		56		
**Monomer M6**	Esterification	78[a]	270.1489	270.1496
	N-formylation	72[c]	298.1438	298.1439
	Dehydration	68[c]	280.1332	280.1336
Overall yield		38		
**Monomer M7**	Esterification	93[a]	256.1332	256.1337
	N-formylation	99[b]	284.1281	284.1288
	Dehydration	55[c]	266.1176	266.1182
Overall yield		51		
**Monomer M8**	Esterification	56[a]	242.1176	242.1181
	N-formylation	quant.[b]	270.1125	270.1132

	Dehydration	39[c]	252.1019	252.1024
		22		
**Monomer M9**	Esterification	88[a]	166.0863	166.0863
	N-formylation	93[b]	193.0733	193.0736
	Transesterification	44[c]	270.1125	270.1132
	Dehydration	76[c]	252.1019	252.1026
Overall yield [%]		27		

[a] after purification by washing, [b] crude, [c] after purification by column chromatography.

1.3.3 Overview of the established library of selectable monomers and aldehyde components

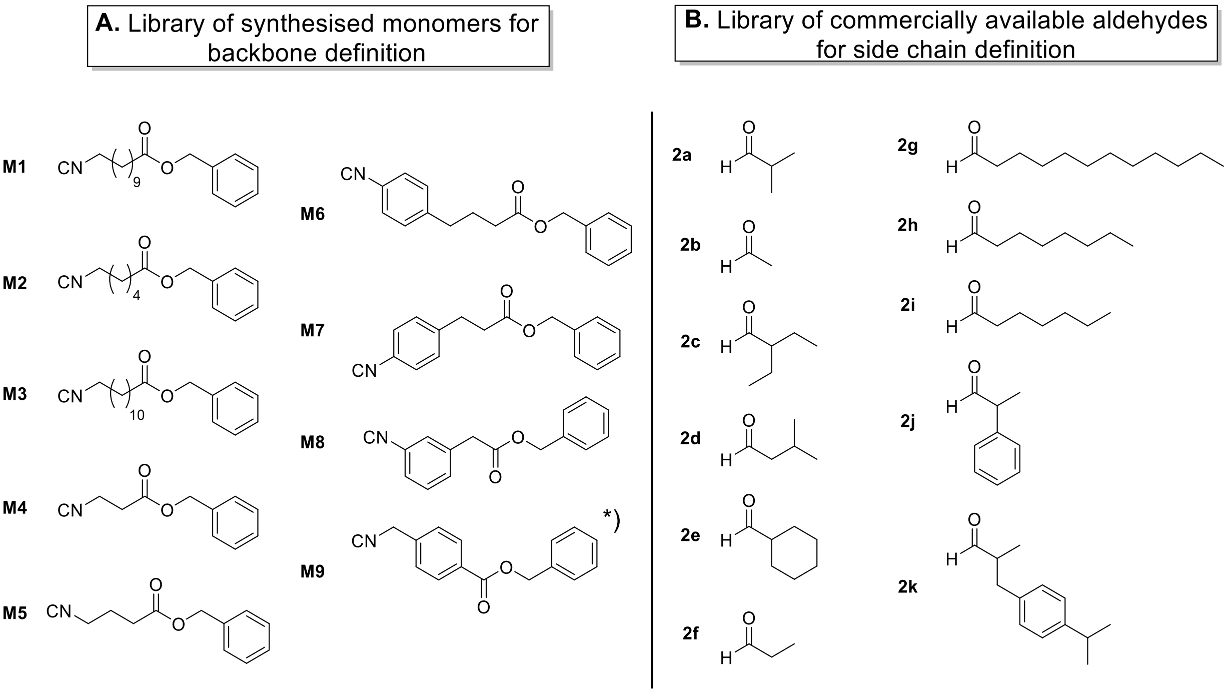

*Supplementary Figure 77 A. library of the AB-type monomers **M1** – **M9** that were synthesised from the corresponding amino acid in three steps; (*) in case of **M9**, the synthesis is performed via a four-step procedure. By variation of the monomers, different backbone moieties can be introduced to the macromolecules. B. set of commercially available aldehydes applied to introduce different side chains to the oligomers.*

1.3.4 Oligomer synthesis

1.3.4.1 Backbone variation

1st Passerini reaction (B1)

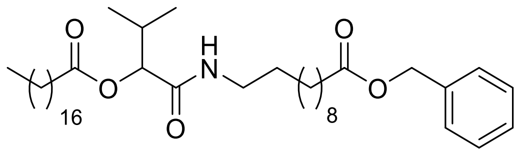

*Supplementary Figure 78. Chemical structure of compound **B1**.*

Stearic acid **1** (1.50 g, 5.27 mmol, 1.00 eq.) was suspended in DCM (5.3 mL, 1. M). Subsequently, isobutyraldehyde **2a** (722 µL, 570 mg, 7.91 mmol, 1.50 eq.) and monomer **M1** (2.38 g, 7.91 mmol, 1.50 eq.) were added and the reaction mixture was stirred at room temperature for 24 hours. The solvent was removed under reduced pressure and the crude product was purified by column chromatography (cyclohexane / ethyl acetate 15:1 → 11:1) to obtain the desired product **B1** in a yield of 98% (3.40 g, 5.17 mmol) as a white solid.

^{1}H NMR (300 MHz, $CDCl_3$) δ / ppm: 7.41 – 7.28 (m, 5H, CH aromatic, ^{1}), 6.00 – 5.90 (m, 1H, NH, ^{2}), 5.11 (s, J = 5.4 Hz, 2H, CH, ^{3}), 5.07 (d, J = 4.4 Hz, 1H, CH_2, ^{4}), 3.36 – 3.15 (m, 2H, CH_2, ^{5}), 2.48 – 2.24 (m, 5H, CH_2, ^{6}), 1.72 – 1.53 (m, 4H, CH_2, ^{7}), 1.53 – 1.42 (m, 2H, CH_2, ^{8}), 1.40 – 1.17 (m, 40H, CH_2, ^{9}), 1.01 – 0.79 (m, 9H, CH_3^{10}).

^{13}C NMR (75 MHz, $CDCl_3$) δ / ppm: 173.78, 172.66, 169.37, 136.25, 128.65, 128.27, 78.00, 66.17, 39.26, 34.43, 32.05, 30.63, 29.82, 29.78, 29.73, 29.70, 29.59, 29.55, 29.48, 29.46, 29.39, 29.32, 29.29, 29.22, 26.96, 25.17, 25.05, 22.81, 18.91, 17.04, 14.25.

HRMS-FAB-MS of $[C_{41}H_{72}N_1O_5]^+$: calculated: 658.5405 found: 658.5404.

IR (ATR platinum diamond): ν / cm^{-1} = 3286.3, 3091.2, 2916.3, 2848.8, 1737.5, 1649.5, 1551.1, 1498.2, 1469.7, 1416.1, 1379.3, 1294.7, 1272.0, 1254.6, 1233.1, 1212.7, 1157.0, 1108.5, 1031.8, 1013.1, 986.7, 927.3, 721.6, 693.6, 578.5, 521.2, 474.1, 414.3.

***R*$_f$**: (cyclohexane / ethyl acetate 5:1) = 0.41.

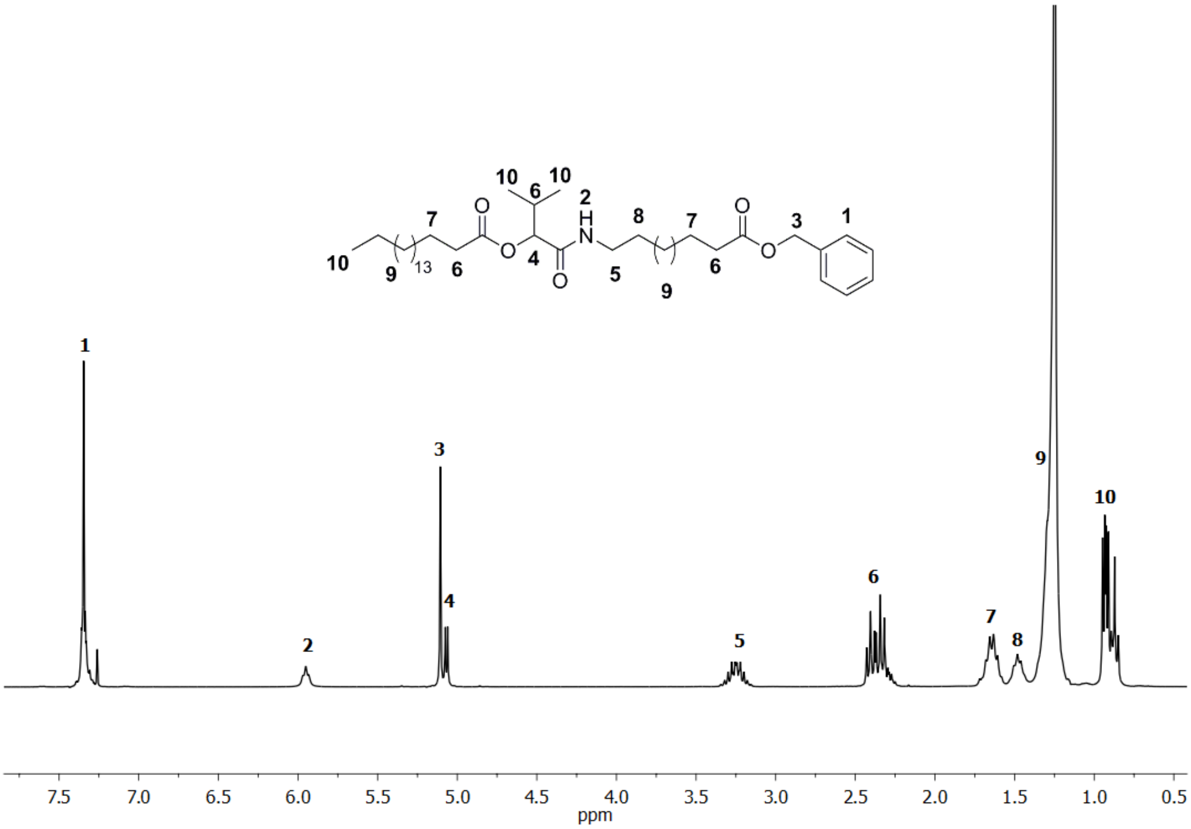

*Supplementary Figure 79. Proton NMR of compound **B1** measured in $CDCl_3$.*

1st deprotection (B1_deprotected)

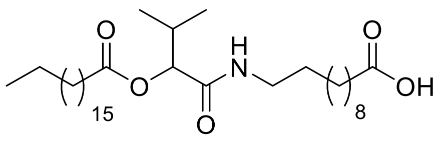

*Supplementary Figure 80. Chemical structure of compound **B1_deprotected**.*

Substance **B1** (2.10 g, 3.19 mmol, 1.00 eq.) was dissolved in ethyl acetate (6.4 mL, 0.5 M). Subsequently, palladium on activated charcoal (0.210 g, 10 wt%.) was suspended in the solution. The reaction mixture was purged with hydrogen (2 balloons) and stirred under hydrogen atmosphere overnight. The heterogeneous catalyst was filtered off and the solvent was evaporated under reduced pressure to obtain the desired product **B1_deprotected** in a yield of 87% (1.57 g, 2.77 mmol) as a white solid.

^{1}H NMR (300 MHz, $CDCl_3$) δ / ppm: 6.11 – 5.89 (m, 1H, NH [1]), 5.06 (d, J = 4.4 Hz, 1H, CH, [2]), 3.40 – 3.14 (m, 2H, CH_2, [3]), 2.45 – 2.23 (m, 5H,CH, CH_2, [4]), 1.70 – 1.54 (m, 4H, CH_2, [5]), 1.52 – 1.37 (m, 2H, CH_2, [6]), 1.36 – 1.16 (m, 40H, CH_2, [7]), 0.97 – 0.73 (m, 9H, CH_3, [8]).

^{13}C NMR (75 MHz, $CDCl_3$) δ / ppm: 179.36, 172.72, 169.54, 132.72, 78.01, 39.30, 34.44, 34.12, 32.05, 30.62, 29.82, 29.78, 29.73, 29.64, 29.59, 29.48, 29.39, 29.28, 29.25, 29.11, 27.50, 27.10, 26.92, 25.17, 24.79, 22.81, 18.89, 17.04, 14.24.

HRMS-FAB-MS of $[C_{34}H_{66}N_1O_5]^+$: calculated: 568.4936 found: 568.4935.

IR (ATR platinum diamond): ν / cm^{-1} = 3288.8, 2913.5, 2849.4, 2341.8, 1736.2, 1701.5, 1653.4, 1561.1, 1470.5, 1431.5, 1410.1, 1373.7, 1349.8, 1326.2, 1271.2, 1253.1, 1234.7, 1215.2, 1199.7, 1098.2, 1023.5, 920.6, 716.4, 682.7, 534.2, 454.0, 410.8.

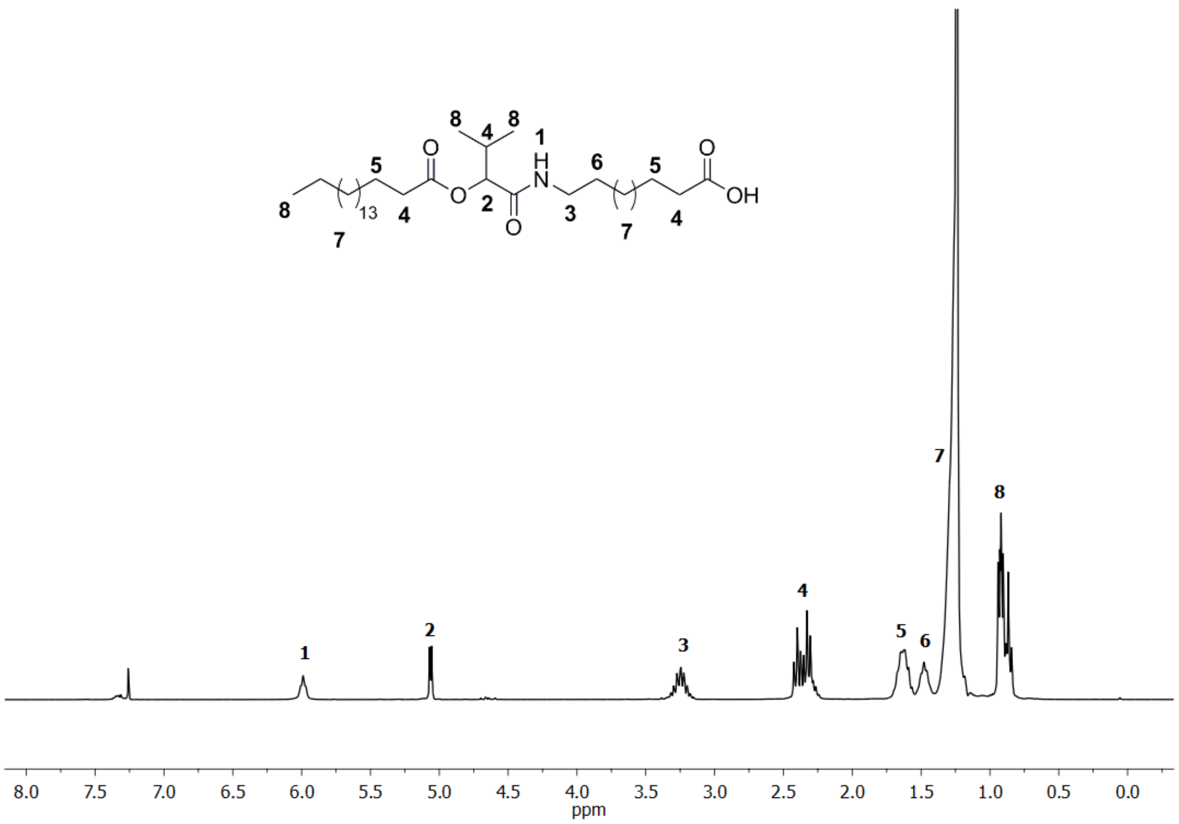

*Supplementary Figure 81. Proton NMR of compound **B1_deprotected** measured in $CDCl_3$.*

2nd Passerini reaction (B2)

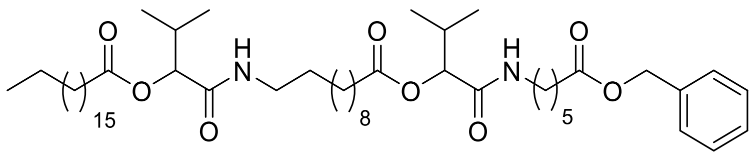

*Supplementary Figure 82. Chemical structure of compound **B2**.*

Substance **B1_deprotected** (1.49 g, 2.62 mmol, 1.00 eq.) was dissolved in DCM (5.2 mL, 0.5 M). Subsequently, isobutyraldehyde **2a** (359 µL, 283 mg, 3.93 mmol, 1.50 eq.) and monomer **M2** (910 mg, 3.93 mmol, 1.50 eq.) were added and the reaction mixture was stirred at room temperature for 24 hours. The solvent was removed under reduced pressure and the crude product was purified by column chromatography (cyclohexane / ethyl acetate 10:1 → 0:1) to obtain the desired product **B2** in a yield of 98% (2.24 g, 2.57 mmol) as a white solid.

^{1}H NMR (300 MHz, $CDCl_3$) δ / ppm: 7.39 – 7.19 (m, 5H, CH aromatic, [1]), 6.08 – 5.90 (m, 2H, NH, [2]), 5.09 (s, 2H, CH, [3]), 5.06 – 5.01 (m, 2H, CH_2, [4]), 3.33 – 3.09 (m, 4H, CH_2, [5]), 2.44 – 2.18 (m, 8H, CH, CH_2, [6]), 1.72 – 1.40 (m, 6H, CH_2, [7]), 1.39 – 1.29 (m, 4H, CH_2, [8]), 1.28 – 1.03 (m, 42H, CH_2, [9]), 1.01 – 0.61 (m, 15H, CH_3, [10]).

^{13}C NMR (75 MHz, $CDCl_3$) δ / ppm: 173.40, 172.65, 169.42, 169.37, 136.09, 128.64, 128.30, 128.26, 77.97, 66.23, 39.21, 38.94, 34.39, 34.34, 34.12, 32.00, 30.60, 29.77, 29.67, 29.54, 29.50, 29.42, 29.32, 29.24, 29.19, 26.90, 26.35, 25.12, 25.06, 24.49, 22.77, 18.86, 17.03, 14.20.

HRMS-ESI-MS of $[M+H]^+$ $[C_{52}H_{91}N_2O_8]^+$: calculated: 871.6770 found: 871.6764.

IR (ATR platinum diamond): ν / cm^{-1} = 3277.4, 3091.4, 2916.4, 2849.9, 1739.2, 1650.7, 1562.7, 1467.6, 1371.6, 1271.5, 1253.6, 1233.8, 1214.1, 1160.5, 1101.8, 995.9, 924.3, 721.0, 697.0.

R_f: (cyclohexane / ethyl acetate 2:3) = 0.55.

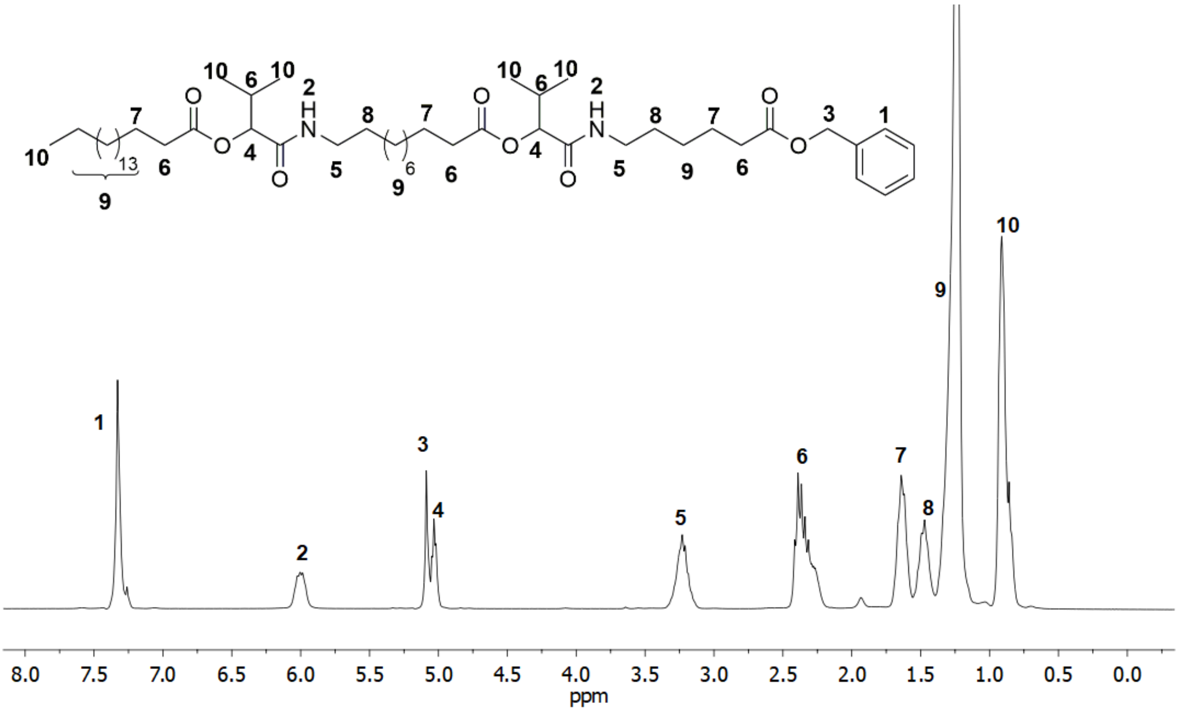

*Supplementary Figure 83. Proton NMR of compound **B2** measured in $CDCl_3$.*

2nd deprotection (B2_deprotected)

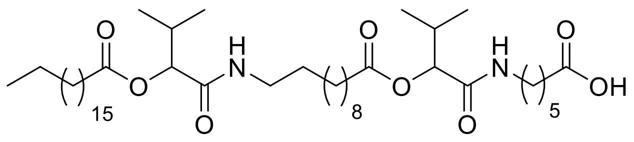

*Supplementary Figure 84. Chemical structure of compound **B2_deprotected**.*

Substance **B2** (2.08 g, 2.41 mmol, 1.00 eq.) was dissolved in ethyl acetate (5.0 mL, 0.5 M). Subsequently, palladium on activated charcoal (0.20 g, 10 wt%.) was suspended in the solution. The reaction mixture was purged with hydrogen (2 balloons) and stirred under hydrogen atmosphere overnight. The heterogeneous catalyst was filtered off and the solvent was evaporated under reduced pressure to obtain the desired product **B2deprotected** in a yield of 93% (1.74 g, 2.23 mmol) as a white solid.

^{1}H NMR (300 MHz, $CDCl_3$) δ / ppm: 6.26 – 6.03 (m, 2H, NH, [1]), 5.07 – 5.01 (m, 2H, CH, [2]), 3.34 – 3.14 (m, 4H, CH_2, [3]), 2.36 (dt, J = 15.2, 7.0 Hz, 8H, CH, CH_2, [4]), 1.76 – 1.63 (m, 6H, CH_2, [5]), 1.64 – 1.41 (m, 4H, CH_2, [6]), 1.41 – 1.12 (m, 42H, CH_2, [7]), 0.99 – 0.72 (m, 15H, CH_3, [8]).

^{13}C NMR (75 MHz, $CDCl_3$) δ / ppm: 177.69, 172.75, 172.73, 169.63, 77.97, 39.30, 39.00, 34.38, 34.33, 33.84, 32.00, 30.58, 29.77, 29.73, 29.68, 29.60, 29.54, 29.50, 29.43, 29.34, 29.28, 29.23, 29.20, 26.87, 26.32, 25.11, 25.07, 24.37, 22.76, 18.84, 17.05, 17.03, 14.19..

HRMS-ESI-MS of $[C_{45}H_{85}N_2O_8]^+$: calculated: 781.6300 found: 781.6297.

IR (ATR platinum diamond): ν / cm^{-1} = 3258.9, 3091.2, 2916.9, 2850.0, 1742.1, 1650.4, 1544.7, 1466.8, 1415.8, 1370.8, 1271.8, 1233.7, 1213.8, 1191.0, 1160.8, 1103.4, 1011.4, 927.6, 720.9, 414.4.

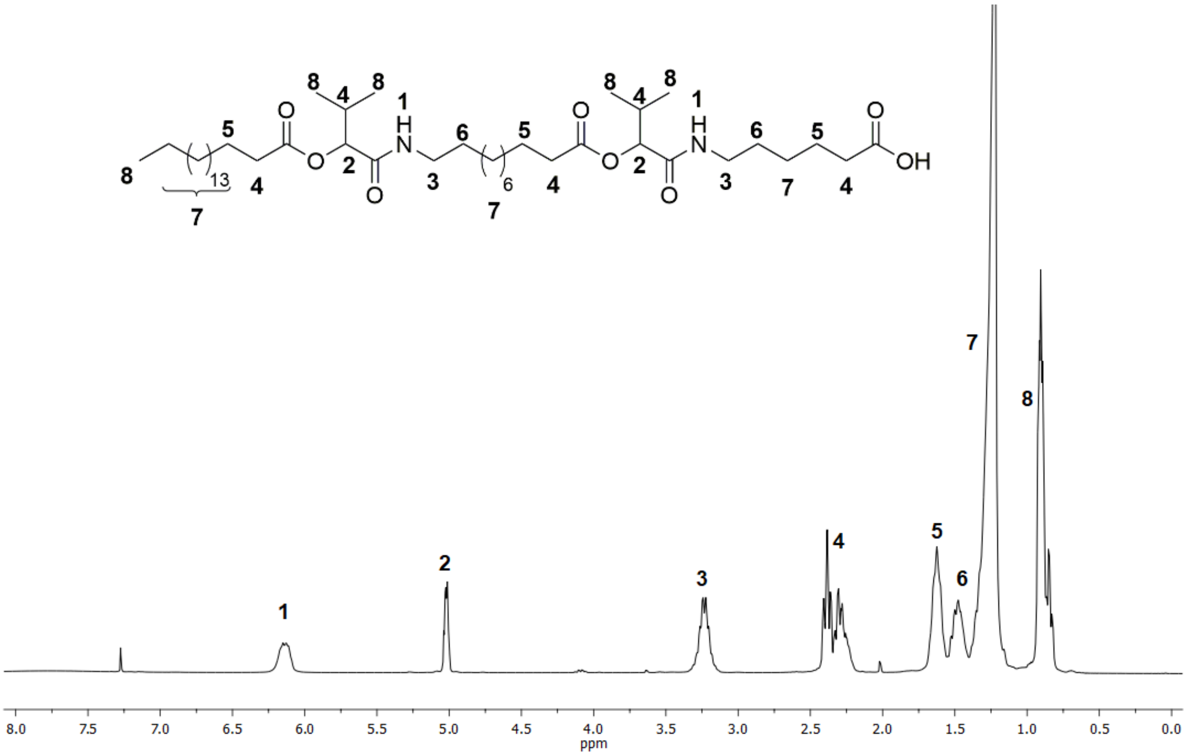

Supplementary Figure 85. Proton NMR of compound ***B2_deprotected*** *measured in $CDCl_3$.*

3rd Passerini reaction (B3)

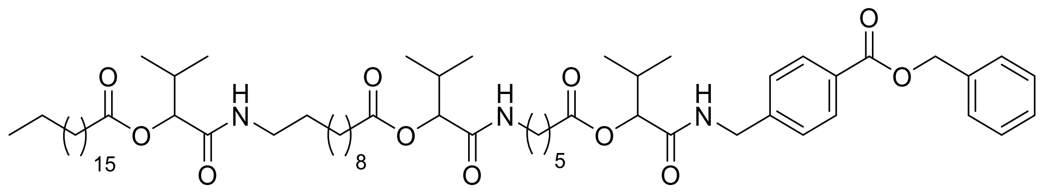

*Supplementary Figure 86. Chemical structure of compound **B3**.*

Substance **B2_deprotected** (1.47 g, 1.89 mmol, 1.00 eq.) was dissolved in DCM (5.4 mL, 0.3 M). Subsequently, isobutyraldehyde **2a** (283 µL mL, 204 mg, 2.83 mmol, 1.50 eq.) and monomer **M9** (0.71 g, 2.83 mmol, 1.50 eq.) were added and the reaction mixture was stirred at room temperature for 48 hours. The solvent was removed under reduced pressure and the crude product was purified by column chromatography (cyclohexane / ethyl acetate 7:1 → 0:1) to obtain the desired product **B3** in a yield of 99% (2.24 g, 1.88 mmol) as a yellowish oil.

^{1}H NMR (300 MHz, $CDCl_3$) δ / ppm: 8.03 (d, J = 8.2 Hz, 2H, CH aromatic, [1]), 7.48 – 7.29 (m, 7H, CH aromatic, [2]), 6.49 (s, 1H, NH, [3]), 5.99 (m, 2H, NH, [4]), 5.35 (s, 2H, CH_2, [5]), 5.12 – 4.96 (m, 3H, CH, [6]), 4.65 – 4.43 (m, 2H, CH_2, [7]), 3.38 – 3.17 (m, 4H, CH_2, [8]), 2.48 – 2.18 (m, 9H, CH, CH_2, [9]), 1.77 – 1.57 (m, 6H, CH_2, [10]), 1.63 – 1.38 (m, 11.2 Hz, 4H, CH_2, [11]), 1.38 – 1.07 (m, 42H, CH_2, [12]), 1.00 – 0.77 (m, 21H, CH_3, [13]).

^{13}C NMR (75 MHz, $CDCl_3$) δ / ppm: 172.76, 172.70, 172.60, 169.69, 169.54, 169.42, 166.23, 143.65, 136.05, 129.60, 129.38, 128.93, 128.51, 128.07, 127.82, 127.37, 77.92, 66.82, 39.24, 34.34, 32.01, 30.66, 30.55, 29.78, 29.25, 28.19, 27.32, 26.17, 25.14, 24.41, 22.78, 21.14, 18.92, 18.32, 14.14.

HRMS-ESI-MS $[M+H]^+$of $[C_{65}H_{105}N_3O_{11}]$: calculated: 1104.7822 found: 1104.7820.

IR (ATR platinum diamond): ν / cm^{-1} = 3274.4, 3090.9, 2918.7, 2850.9, 1741.7, 1650.4, 1612.8, 1533.3, 1465.8, 1416.5, 1371.2, 1270.1, 1161.4, 1102.1, 1018.0, 747.6, 721.6, 696.8, 525.4, 411.0.

R_f: (cyclohexane / ethyl acetate 1:1) = 0.58.

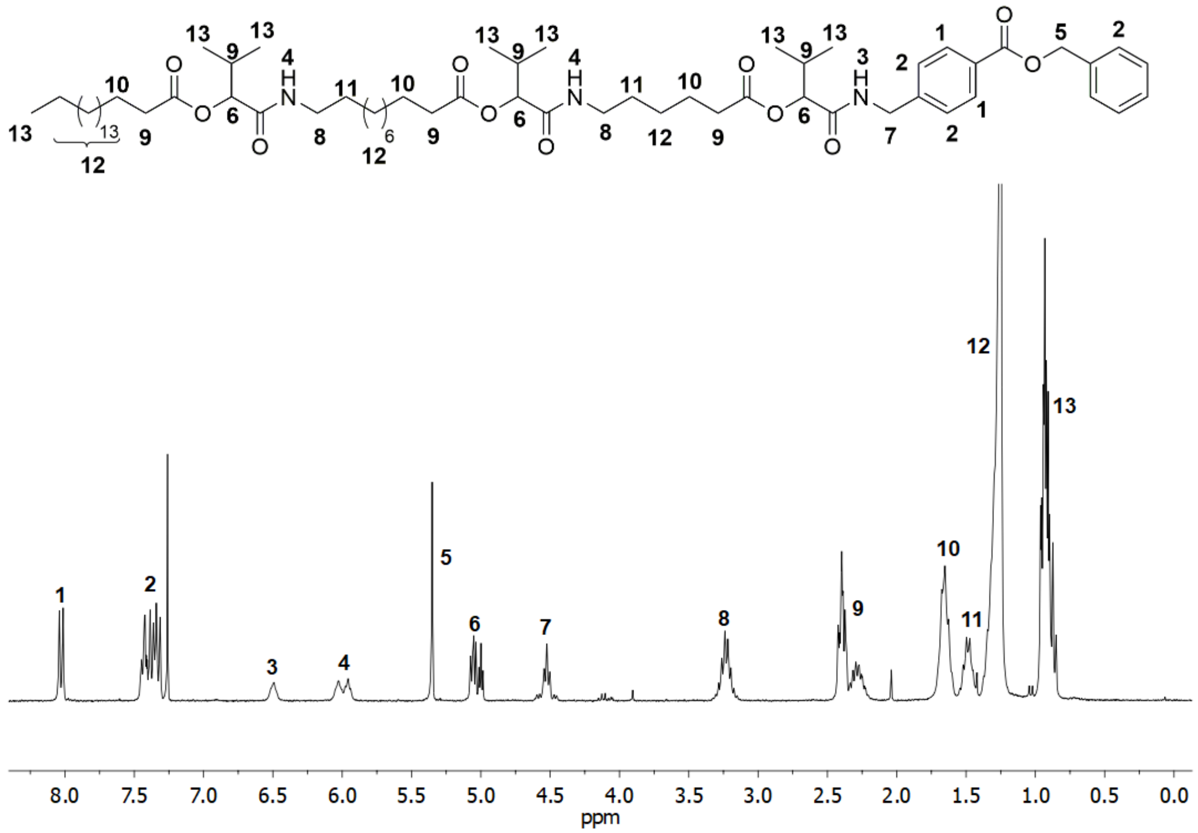

Supplementary Figure 87. Proton NMR of compound ***B3*** *measured in* $CDCl_3$.

3rd deprotection (B3_deprotected)

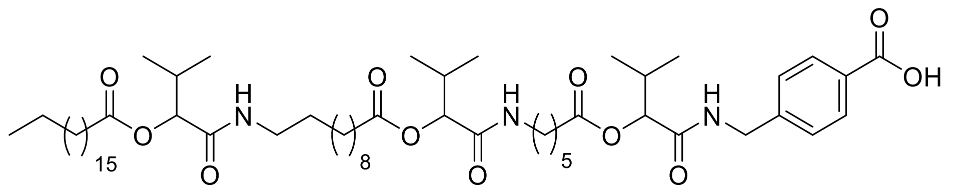

*Supplementary Figure 88. Chemical structure of compound **B3_deprotected**.*

Substance **B3** (2.07 g, 1.87 mmol, 1.00 eq.) was dissolved in ethyl acetate (3.80 mL, 0.5 M). Subsequently, palladium on activated charcoal (0207 mg, 10 wt%.) was suspended in the solution. The reaction mixture was purged with hydrogen (3 balloons) and stirred under hydrogen atmosphere overnight. The heterogeneous catalyst was filtered off and the solvent was evaporated under reduced pressure to obtain the desired product **B3_deprotected** in a yield of 81% (1.53 g, 1.51 mmol) as a white solid.

^{1}H NMR (400 MHz, $CDCl_3$) δ / ppm: 8.04 – 7.92 (m, 2H, CH aromatic, [1]), 7.36 – 7.28 (m, 2H, CH aromatic, [2]), 6.79 (s, 1H, NH, [3]), 6.20 (s, 2H, NH, [4]), 5.04 (d, J = 1.6 Hz, 1H, CH, [5]), 5.00 – 4.94 (m, 2H, CH, [6]), 4.60 – 4.40 (m, 2H, CH, [7]), 3.38 – 3.11 (m, 4H, CH, [8]), 2.48 – 2.17 (m, 9H, CH, CH_2, [9]), 1.70 – 1.54 (m, 6H, CH_2, [10]), 1.47 (d, J = 6.3 Hz, 4H, CH_2, [11]), 1.47 – 1.11 (m, 42H, CH_2, [12]), 1.05 – 0.70 (m, 21H, CH_3, [13]).

^{13}C NMR (101 MHz, $CDCl_3$) δ / ppm: 172.94, 172.78, 169.82, 169.76, 169.68, 143.97, 130.55, 129.02, 127.60, 78.24, 78.05, 77.97, 77.36, 42.90, 39.31, 38.84, 34.40, 34.33, 34.00, 32.01, 30.70, 30.60, 29.78, 29.74, 29.69, 29.61, 29.56, 29.49, 29.45, 29.41, 29.36, 29.25, 29.21, 29.18, 26.88, 26.14, 25.12, 25.05, 24.39, 22.78, 18.87, 17.29, 17.12, 17.03, 14.22.

HRMS-ESI-MS $[M+H]^+$ of $[C_{58}H_{99}N_3O_{11}]$: calculated: 1014.7352 found: 1014.7343.

IR (ATR platinum diamond): ν / cm^{-1} = 3276.6, 3091.0, 2917.1, 2850.2, 1740.2, 1692.1, 1651.0, 1613.2, 1547.0, 1466.4, 1433.1, 1370.1, 1317.1, 1294.0, 1234.1, 1164.4, 1017.0, 929.3, 854.2, 762.1, 720.9, 548.8, 414.9.

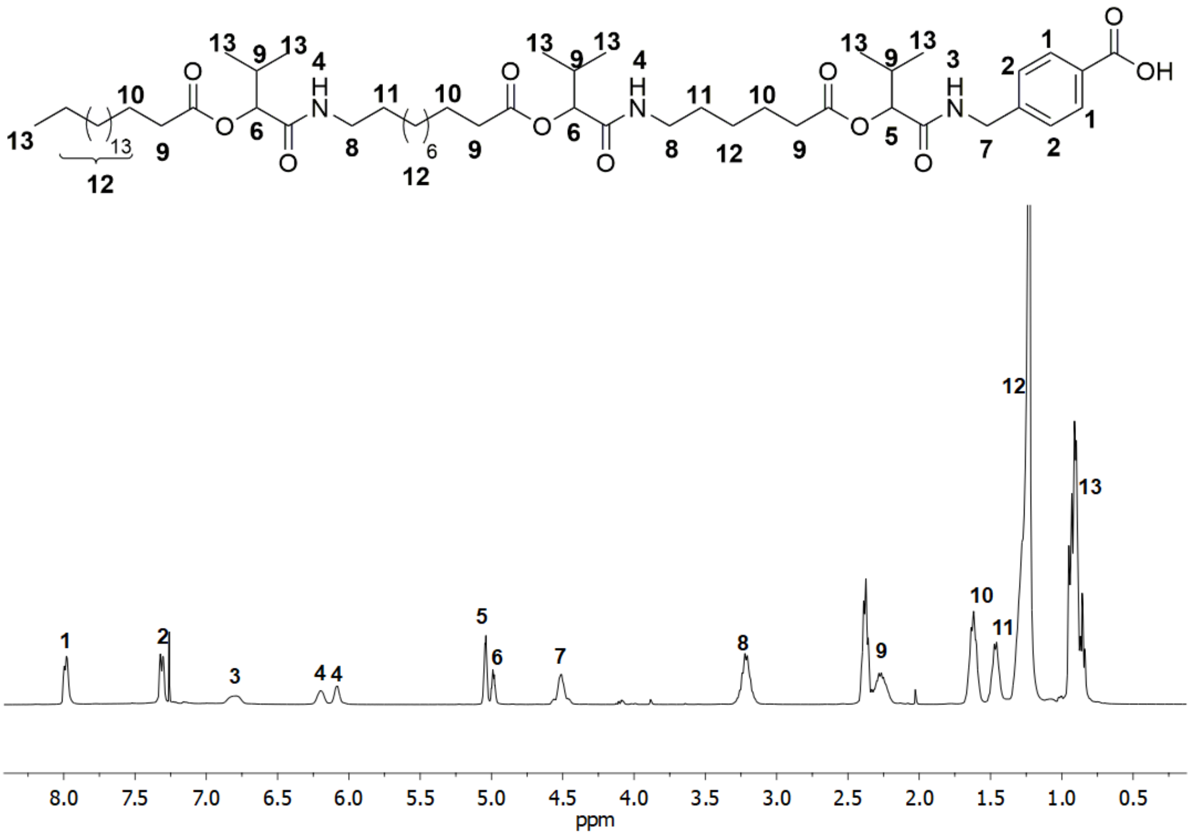

Supplementary Figure 89. Proton NMR of compound ***B3_deprotected*** *measured in* $CDCl_3$.

4th Passerini reaction (B4)

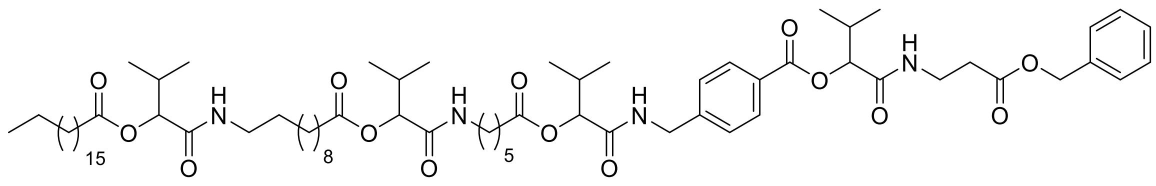

*Supplementary Figure 90. Chemical structure of compound **B4**.*

Substance **B3_deprotected** (1.35 g, 1.35 mmol, 1.00 eq.) was dissolved in DCM (2.70 mL, 0.5 M). Subsequently, isobutyraldehyde **2a** (185 µL, 146 mg g, 2.03 mmol, 1.50 eq.) and monomer **M4** (380 mg, 2.03 mmol, 1.50 eq.) were added and the reaction mixture was stirred at room temperature for 48 hours. The solvent was removed under reduced pressure and the crude product was purified by column chromatography (cyclohexane / ethyl acetate 3:1 → 1:3) to obtain the desired product **B4** in a yield of 92% (1.57 g, 1.23 mmol) as a yellowish oil.

^{1}H NMR (400 MHz, $CDCl_3$) δ / ppm: 8.00 (d, J = 8.1 Hz, 2H, CH aromatic, [1]), 7.39 – 7.22 (m, 7H, CH aromatic, [2]), 6.79 – 6.64 (m, 2H, NH, [3]), 6.04 (dt, J = 11.1, 4.7 Hz, 2H, NH, [4]), 5.26 – 4.92 (m, 4H, CH, [5]), 4.62 – 4.42 (m, 2H, CH_2, [6]), 3.62 – 3.41 (m, 2H, CH_2, [7]), 3.32 – 3.09 (m, 5H, CH, CH_2, [8]), 2.68 – 2.18 (m, 11H, CH, CH_2, [9]), 1.70 – 1.57 (m, 6H, CH_2, [10]), 1.54 – 1.38 (m, 4H, CH_2, [11]), 1.36 – 1.15 (m, 42H, CH_2, [12]), 1.03 – 0.72 (m, 27H, [13]).

^{13}C NMR (101 MHz, $CDCl_3$) δ / ppm: 172.77, 172.68, 172.62, 172.27, 169.74, 169.70, 169.51, 169.45, 169.39, 165.26, 144.30, 135.59, 130.24, 128.67, 128.53, 128.45, 128.28, 127.76, 127.73, 78.61, 78.16, 77.99, 77.94, 66.59, 42.82, 39.21, 38.72, 34.69, 34.38, 34.32, 34.02, 33.92, 31.99, 30.84, 30.71, 30.58, 29.76, 29.72, 29.67, 29.65, 29.54, 29.49, 29.43, 29.41, 29.34, 29.26, 29.23, 29.18, 26.89, 26.12, 25.11, 25.05, 24.38, 24.32, 22.76, 18.95, 18.85, 17.26, 17.05, 17.01, 14.27, 14.20.

HRMS-ESI-MS $[M+H]^+$ of $[C_{73}H_{118}N_4O_{14}]$: calculated: 1275.8717 found: 1275.8743.

IR (ATR platinum diamond): ν / cm^{-1} = 3289.2, 3090.0, 2918.5, 2850.8, 1738.0, 1650.8, 1533.3, 1465.8, 1416.6, 1369.9, 1254.6, 1162.5, 1101.8, 1017.8, 927.8, 747.1, 721.0, 697.5, 410.2.

***R*$_f$**: (cyclohexane / ethyl acetate 1:3) = 0.48.

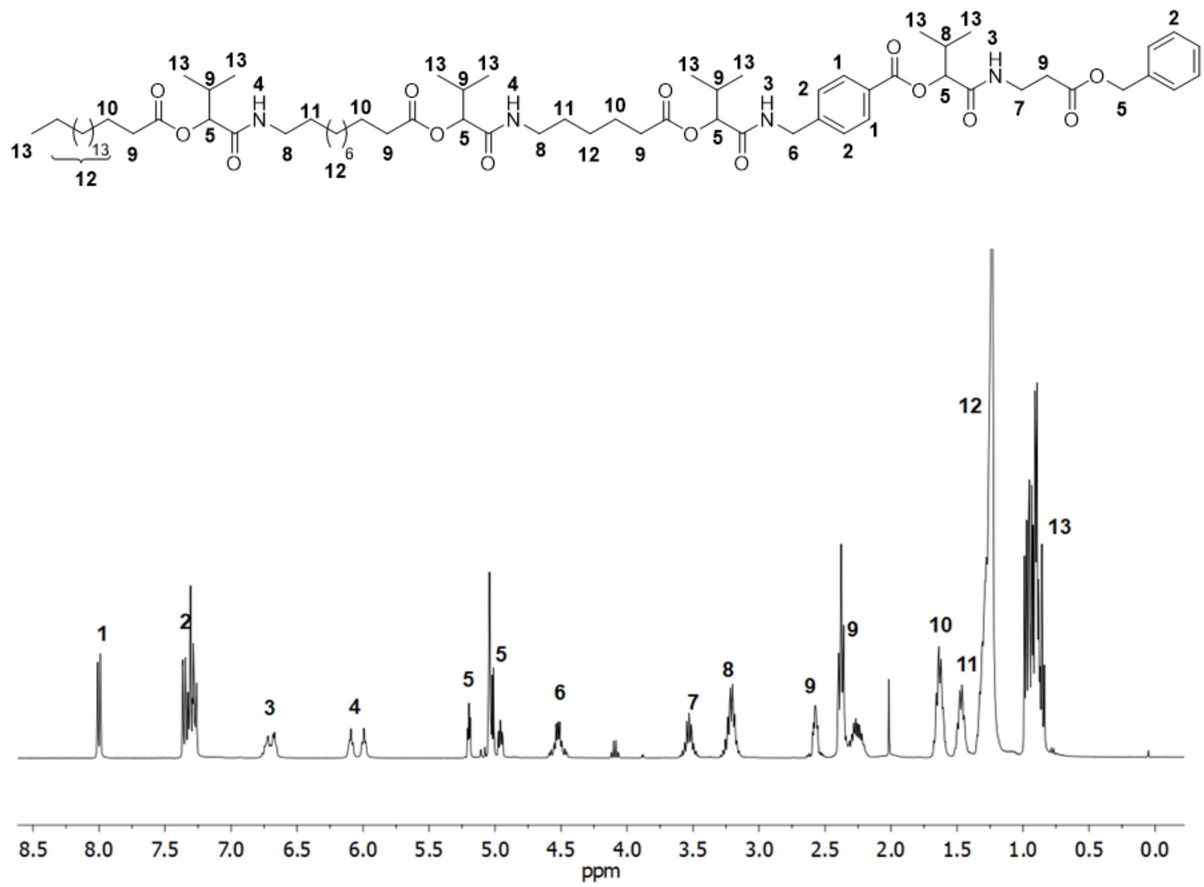

*Supplementary Figure 91. Proton NMR of compound **B4** measured in $CDCl_3$.*

4th deprotection (B4_deprotected)

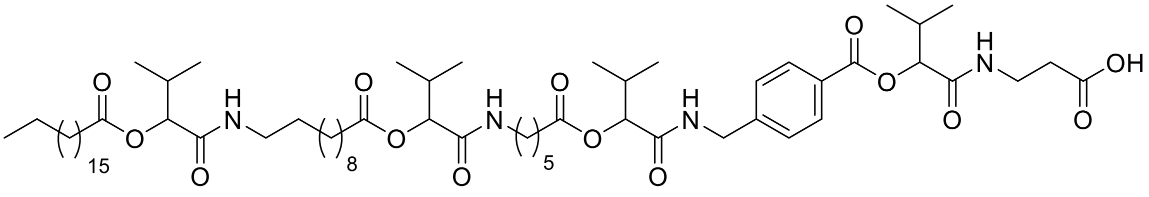

Supplementary Figure 92. Chemical structure of compound ***B4_deprotected****.*

Substance **B4** (1.49 g, 1.16 mmol, 1.00 eq.) was dissolved in ethyl acetate (5.0 mL, 0.25 M). Subsequently, palladium on activated charcoal (116 mg, 10 wt%.) was suspended in the solution. The reaction mixture was purged with hydrogen (3 balloons) and stirred under hydrogen atmosphere overnight. The heterogeneous catalyst was filtered off and the solvent was evaporated under reduced pressure to obtain the desired product **B4_deprotected** in a yield of 99% (1.41 g, 1.16 mmol) as a highly viscous oil.

^{1}H NMR (300 MHz, $CDCl_3$) δ / ppm: 8.00 (d, J = 8.1 Hz, 2H, CH aromatic, [1]), 7.35 (t, J = 7.6 Hz, 2H, CH aromatic, [2]), 7.12 – 6.83 (m, 2H, NH, [3]), 6.38 – 5.95 (m, 2H, NH, [4]), 5.32 – 5.16 (m, 1H, CH, [5]), 5.13 – 4.89 (m, 3H, CH, [6]), 4.61 – 4.32 (m, 2H, CH_2, [7]), 3.57 – 3.40 (m, 2H, CH_2, [8]), 3.31 – 3.05 (m, 4H, CH_2, [9]), 2.59 – 2.13 (m, 12H, CH, CH_2, [10]), 2.12 – 1.53 (m, 6H, CH_2, [11]), 1.53 – 1.32 (m, 4H, CH_2, [12]), 1.32 – 1.15 (m, 42H, CH_2, [13]), 1.14 – 0.79 (m, 27H, CH_3, [14]).

^{13}C NMR (101 MHz, $CDCl_3$) δ / ppm: 174.60, 172.91, 172.74, 170.02, 169.97, 169.64, 165.26, 144.22, 130.22, 128.45, 127.67, 107.71, 106.44, 103.88, 100.08, 78.46, 78.35, 77.91, 68.03, 67.74, 67.51, 67.42, 67.10, 66.10, 42.83, 41.17, 39.27, 38.86, 34.52, 34.38, 34.30, 33.94, 33.56, 32.36, 32.00, 30.92, 30.59, 29.78, 29.73, 29.68, 29.60, 29.55, 29.49, 29.44, 29.41, 29.35, 29.26, 29.24, 29.22, 29.18, 28.94, 26.88, 26.08, 25.68, 25.11, 25.04, 24.29, 23.92, 23.48, 22.77, 18.99, 18.86, 17.39, 17.30, 17.06, 17.01, 14.22.

HRMS-ESI-MS $[M+H]^+$ of $[C_{66}H_{112}N_4O_{14}]$: calculated: 1185.8248 found: 1185.8254.

IR (ATR platinum diamond): ν / cm^{-1} = 3271.1, 3090.3, 2918.3576, 3.7671, 1741.1, 1650.3, 1537.7, 1465.8, 1416.5, 1370.0, 1254.2, 1162.1, 1102.0, 994.5, 926.7, 720.1.

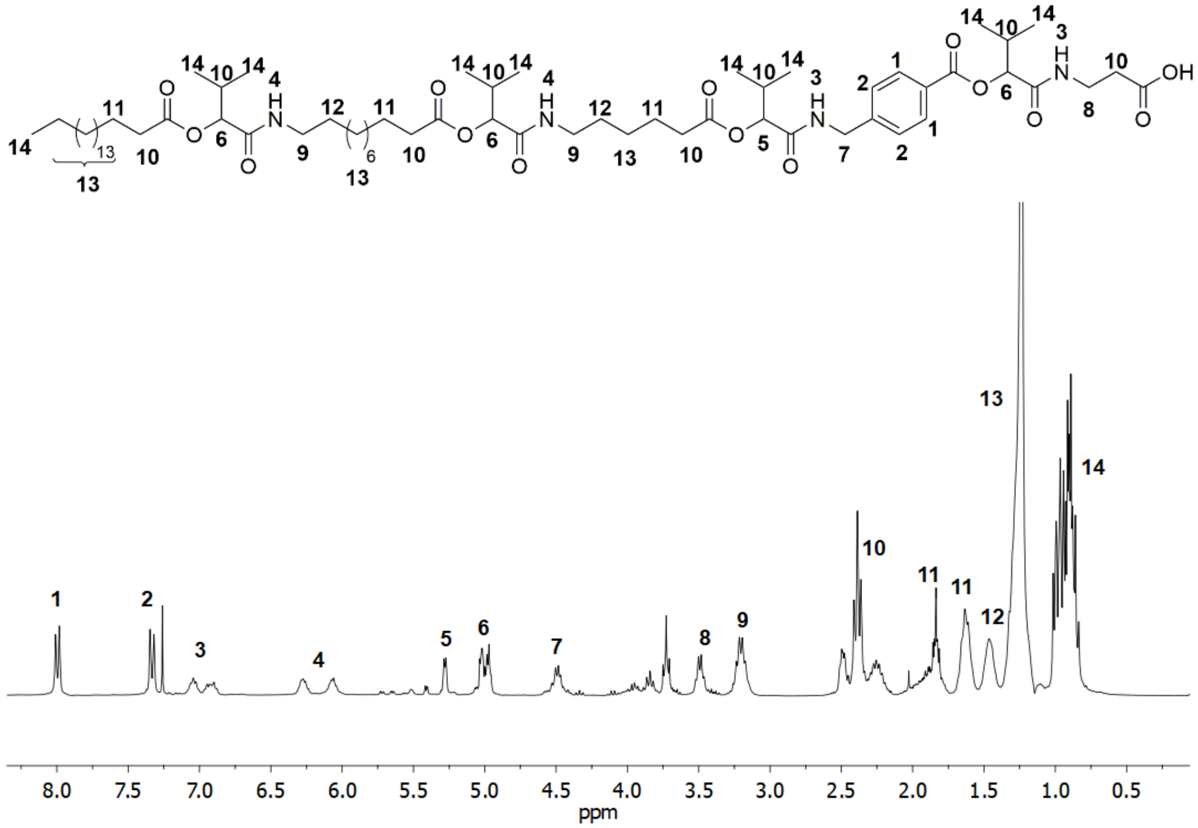

Supplementary Figure 93. Proton NMR of compound **B4_deprotected** measured in $CDCl_3$.

5th Passerini reaction (B5)

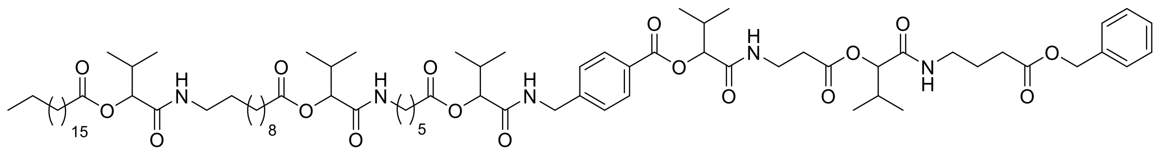

*Supplementary Figure 94. Chemical structure of compound **B5**.*

Substance **B4_deprotected** (1.39 g, 1.18 mmol, 1.00 eq.) was dissolved in DCM (4.0 mL, 0.3 M). Subsequently, isobutyraldehyde **2a** (164 µL, 129 mg, 1.80 mmol, 1.50 eq.) and monomer **M5** (366 mg, 1.80 mmol, 1.50 eq.) were added and the reaction mixture was stirred at room temperature for 48 hours. The solvent was removed under reduced pressure and the crude product was purified by column chromatography (cyclohexane / ethyl acetate 2:1 → 1:4) to obtain the desired product **B5** in a yield of 82% (1.40 g, 0.968 mmol) as a highly viscous oil.

^{1}H NMR (400 MHz, $CDCl_3$) δ / ppm: 8.06 – 7.94 (m, 2H, CH aromatic, [1]), 7.46 – 7.26 (m, 7H, CH aromatic, [2]), 7.08 – 6.59 (m, 3H, NH, [3]), 6.21 – 5.91 (m, 2H, CH_2, [4]), 5.27 – 4.73 (m, 5H, CH, [5]), 4.67 – 4.33 (m, 2H, CH_2, [6]), 3.79 – 3.40 (m, 4H, CH_2, [7]), 3.36 – 3.02 (m, 4H, CH_2, [8]), 2.75 – 1.72 (m, 15H, CH, CH_2, [9]), 1.71 – 1.56 (m, 8H, CH_2, [10]), 1.55 – 1.39 (m, 4H, CH_2, [11]), 1.39 – 1.15 (m, 42H, CH_2, [12]), 1.09 – 0.74 (m, 33H, CH_3, [13]).

^{13}C NMR (101 MHz, $CDCl_3$) δ / ppm: 173.71, 172.83, 172.72, 171.65, 169.79, 169.74, 169.56, 169.44, 169.34, 165.53, 165.29, 144.28, 135.80, 130.30, 130.23, 128.71, 128.43, 128.27, 127.76, 127.67, 78.96, 78.63, 78.21, 77.97, 77.36, 66.65, 66.61, 58.52, 42.87, 39.25, 38.79, 34.58, 34.42, 34.36, 34.20, 34.02, 32.03, 31.83, 30.88, 30.74, 30.62, 29.80, 29.76, 29.71, 29.68, 29.57, 29.53, 29.46, 29.38, 29.30, 29.26, 29.22, 26.92, 26.18, 25.15, 25.09, 24.49, 24.41, 24.17, 22.80, 19.01, 18.89, 18.54, 17.34, 17.11, 17.03, 16.93, 16.85, 14.24.

HRMS-ESI-MS $[M+H]^+$of $[C_{82}H_{133}N_5O_{17}]$: calculated: 1460.9769 found: 1460.9792.

IR (ATR platinum diamond): ν / cm^{-1} = 3304.1, 2963.7, 2924.6, 2853.7, 1735.9, 1654.0, 1533.2, 1463.7, 1417.2, 1369.8, 1246.5, 1162.6, 1099.1, 1018.2, 745.9, 697.5, 638.4.

***R*$_f$**: (cyclohexane / ethyl acetate 1:4) = 0.52.

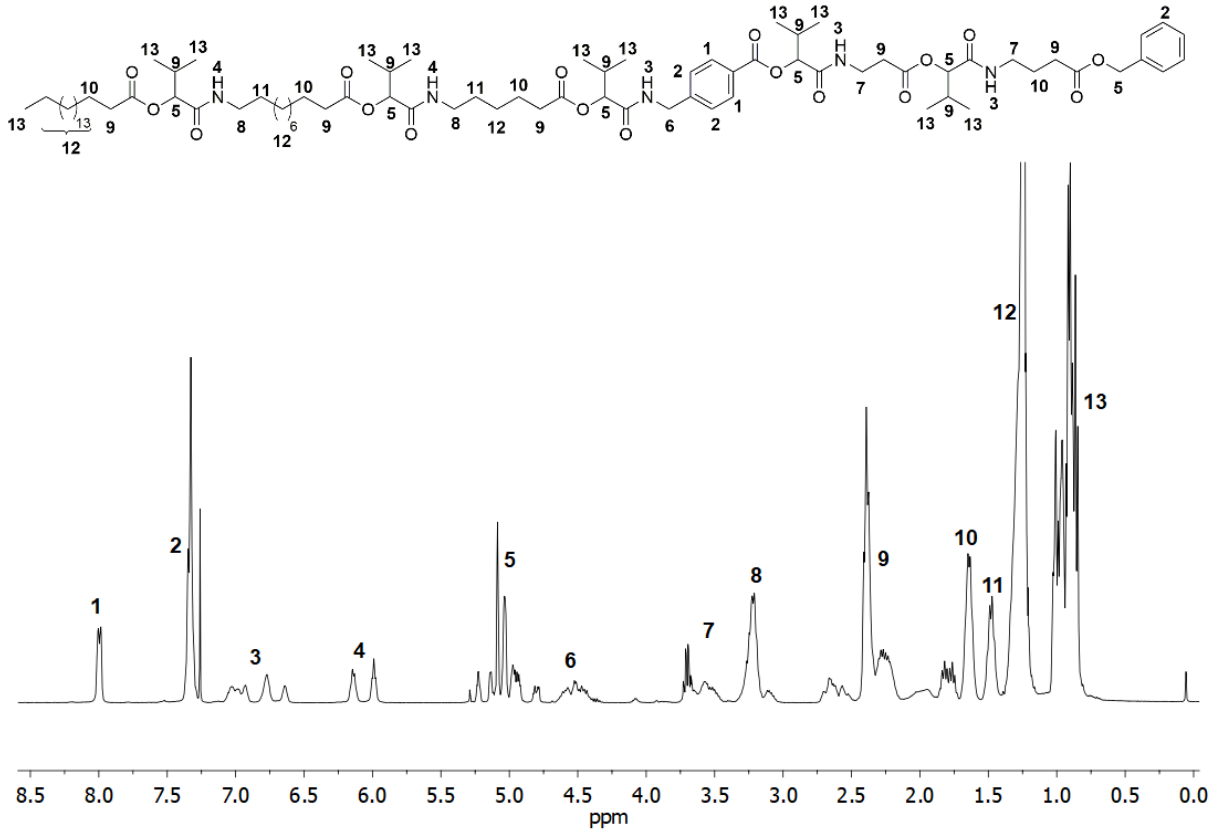

*Supplementary Figure 95. Proton NMR of compound **B5** measured in $CDCl_3$.*

5th deprotection (B5_deprotected)

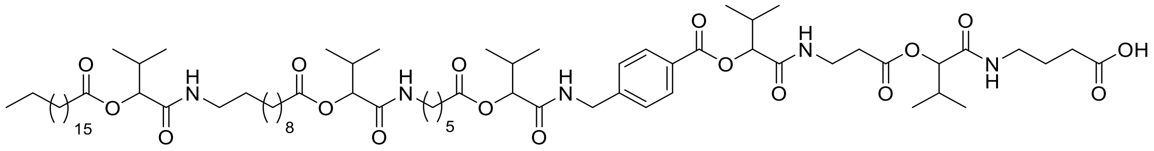

*Supplementary Figure 96. Chemical structure of compound **B5_deprotected**.*

Substance **B5** (1.33 g, 0.910 mmol, 1.00 eq.) was dissolved in THF (6.0 mL, 0.15 M). Subsequently, palladium on activated charcoal (133 mg, 10 wt%.) was suspended in the solution. The reaction mixture was purged with hydrogen (3 balloons) and stirred under hydrogen atmosphere overnight. The heterogeneous catalyst was filtered off and the solvent was evaporated under reduced pressure to obtain the desired product **B5_deprotected** in a yield of 97% (1.21 g, 0.883 mmol) as a highly viscous oil.

^{1}H NMR (400 MHz, $CDCl_3$) δ / ppm: 8.02 - 7.88 (m, 2H, CH aromatic, [1]), 7.40 - 7.27 (m, 2H, CH aromatic, [2]), 7.23 – 6.90 (m, 3H, NH, [3]), 6.25 – 5.88 (m, 2H, CH_2, [4]), 5.22 – 4.74 (m, 5H, CH, [5]), 4.64 – 4.32 (m, 2H, CH_2, [6]), 3.63 – 3.41 (m, 2H, CH_2, [7]), 3.30 – 3.00 (m, 6H, CH_2, [8]), 2.97 – 2.74 (m, 2H, CH_2, [9]), 2.59 – 2.09 (m, 13H, CH, CH_2, [10]), 1.87 – 1.67 (m, 2H, CH_2, [11]), 1.65 – 1.52 (m, 6H, CH_2, [12]), 1.50 – 1.32 (m, 4H, CH_2, [13]), 1.31 – 1.03 (m, 42H, CH_2, [14]), 1.05 – 0.62 (m, 33H, CH_3, [15]).

^{13}C NMR (101 MHz, $CDCl_3$) δ / ppm: 176.54, 176.38, 172.91, 172.74, 171.41, 170.66, 170.32, 169.88, 169.75, 169.54, 165.60, 165.41, 144.44, 130.28, 130.24, 128.39, 127.76, 127.65, 78.83, 78.73, 78.26, 77.93, 42.87, 39.86, 39.62, 39.26, 38.80, 35.05, 34.40, 34.34, 34.01, 33.95, 33.72, 32.16, 32.01, 30.90, 30.70, 30.61, 30.52, 29.79, 29.74, 29.69, 29.64, 29.56, 29.51, 29.45, 29.43, 29.36, 29.28, 29.25, 29.20, 29.11, 26.90, 26.11, 25.13, 25.06, 24.36, 23.36, 23.02, 22.78, 18.94, 18.87, 17.34, 17.25, 17.07, 17.02, 16.83, 16.71, 16.68, 14.23.

HRMS-ESI-MS $[M+H]^+$ of $[C_{75}H_{127}N_5O_{17}]$: calculated: 1370.9300 found: 1370.9318.

IR (ATR platinum diamond): ν / cm^{-1} = 3296.6, 3089.9, 2963.3, 2920.5, 2851.4, 1737.3, 1650.6, 1537.0, 1465.2, 1416.6, 1369.9, 1245.3, 1162.7, 1101.7, 1017.9, 928.0, 720.1, 639.6, 409.3.

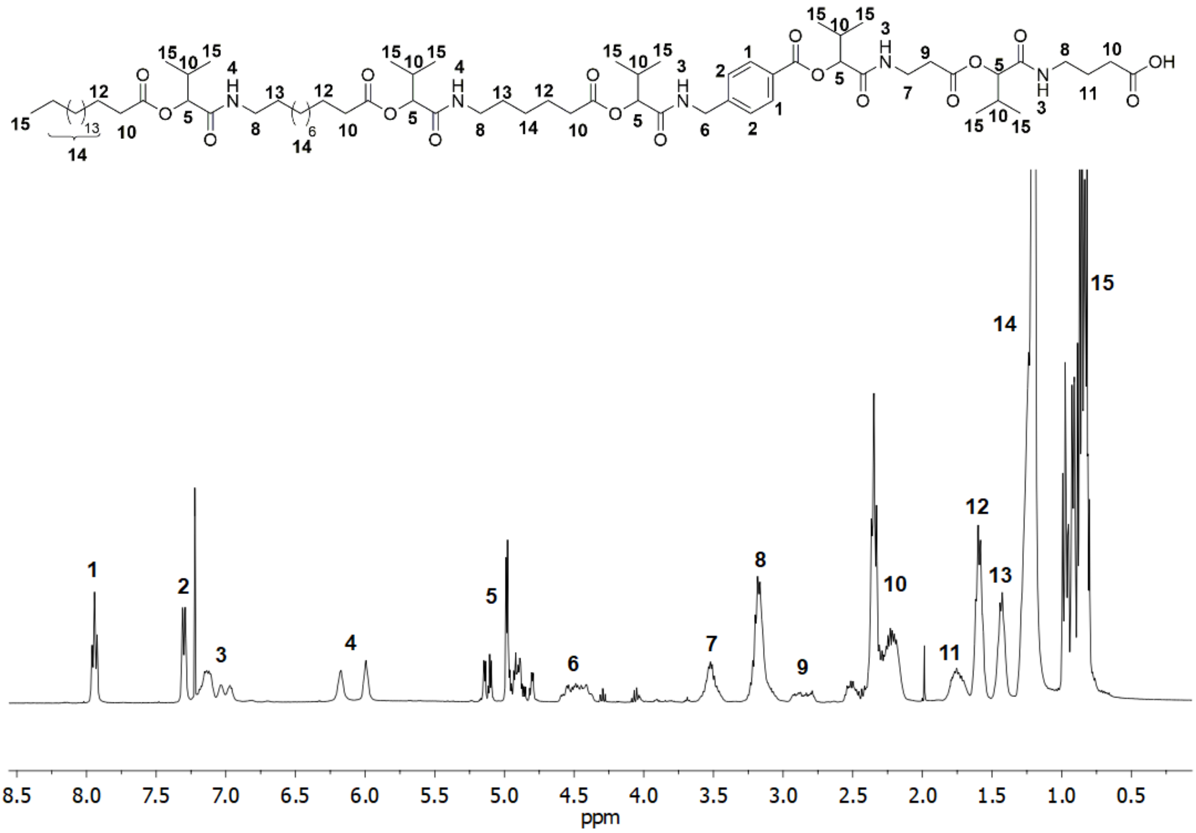

*Supplementary Figure 97. Proton NMR of compound **B5_deprotected** measured in $CDCl_3$.*

6th Passerini reaction (B6)

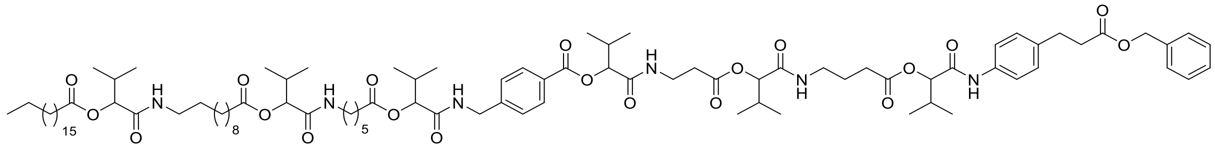

Supplementary Figure 98. Chemical structure of compound ***B6****.*

Substance **B5_deprotected** (1.14 g, 0.833 mmol, 1.00 eq.) was dissolved in DCM (5.0 mL, 0.15 M). Subsequently, isobutyraldehyde **2a** (230 µL, 180 mg, 2.50 mmol, 1.50 eq.) and monomer **M7** (0.66 g, 2.50 mmol, 1.50 eq.) were added and the reaction mixture was stirred at room temperature for 48 hours. The solvent was removed under reduced pressure and the crude product was purified by column chromatography (cyclohexane / ethyl acetate 3:2 → 1:2) to obtain the desired product **B6** in a yield of 85% (1.27 g, 0.745 mmol) as a yellowish solid.

^{1}H NMR (400 MHz, $CDCl_3$) δ / ppm: 9.08 – 8.78 (m, 1H, NH, [1]), 8.03 – 7.84 (m, 2H, CH aromatic, [2]), 7.63 – 7.46 (m, 2H, CH aromatic, [3]), 7.43 – 7.16 (m, 7H, CH aromatic, [4]), 7.16 – 7.03 (m, 2H, CH aromatic, [5]), 7.00 – 6.80 (m, 3H, NH, [6]), 6.24 – 5.94 (m, 2H, NH, [7]), 5.22 – 5.12 (m, 1H, CH, [9]), 5.06 (s, 2H, CH_2, [8]), 5.04 – 4.67 (m, 7H, CH, CH_2, [9]), 4.64 – 4.31 (m, 2H, CH_2, [10]), 3.38 – 2.83 (m, 8H, CH_2, [11]), 2.80 – 2.08 (m, 18H, CH, CH_2, [12]), 1.82 – 1.56 (m, 8H, CH_2, [13]), 1.55 – 1.37 (m, 4H, CH_2, [14]), 1.36 – 1.10 (m, 42H, CH_2, [15]), 1.09 – 0.60 (m, 39H, CH_3, [16]).

^{13}C NMR (101 MHz, $CDCl_3$) δ / ppm: 172.78, 172.71, 169.93, 169.59, 169.45, 136.05, 135.93, 130.25, 130.17, 128.78, 128.64, 128.29, 127.58, 120.93, 120.81, 120.22, 79.10, 78.25, 77.94, 77.48, 77.16, 76.84, 66.38, 39.23, 38.73, 36.05, 34.39, 34.34, 34.01, 32.00, 30.88, 30.67, 30.60, 30.47, 29.77, 29.73, 29.68, 29.66, 29.55, 29.50, 29.44, 29.35, 29.28, 29.24, 29.19, 26.90, 26.10, 25.12, 25.06, 24.39, 22.77, 18.97, 18.87, 17.32, 17.16, 17.08, 17.01, 14.22.

HRMS-ESI-MS $[M+H]^+$of $[C_{96}H_{150}N_6O_{20}]$: calculated: 1708.0978 found: 1708.0978.

IR (ATR platinum diamond): ν / cm^{-1} = 3308.4, 2964.1, 2925.0, 2853.7, 1736.5, 1655.9, 1611.3, 1530.9, 1464.0, 1415.6, 1369.9, 1245.6, 1158.8, 1126.5, 1105.2, 1018.0, 833.5, 746.8, 697.6, 648.0.

***R*$_f$**: (cyclohexane / ethyl acetate 1:4) = 0.37.

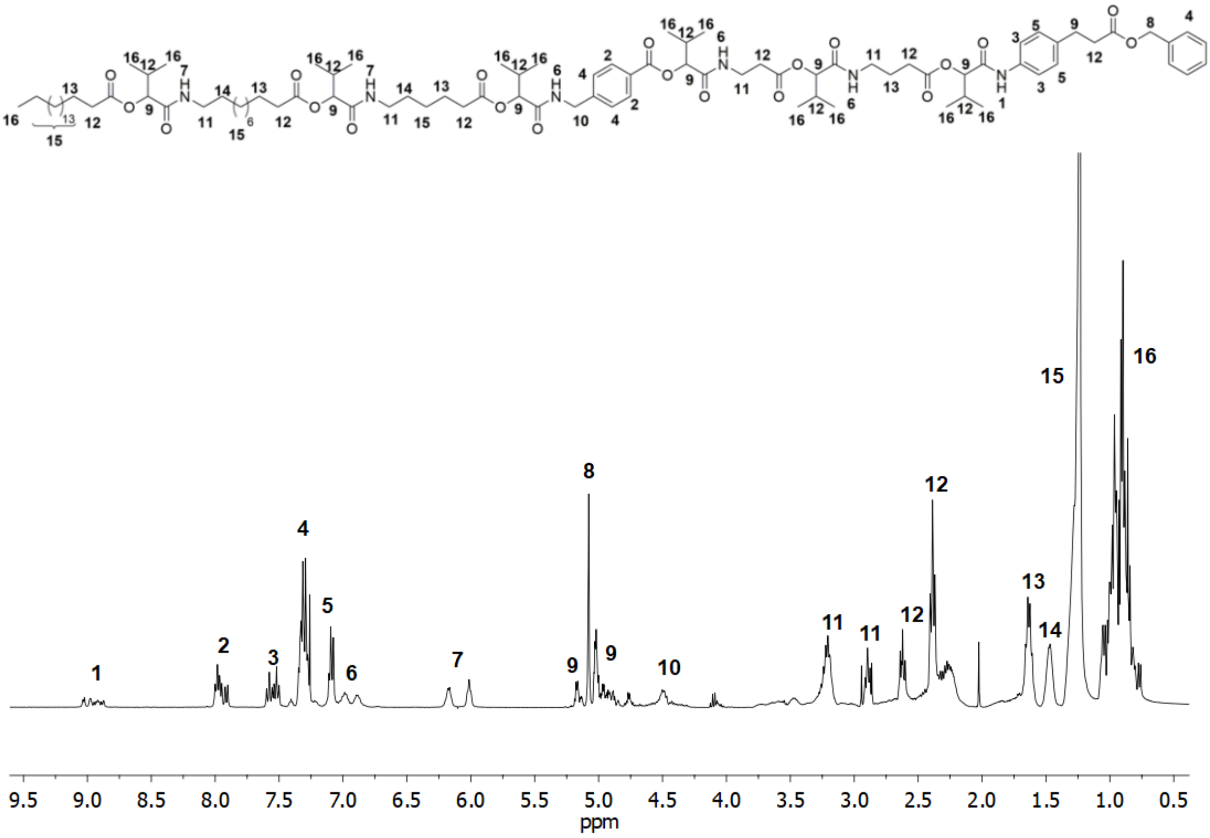

*Supplementary Figure 99. Proton NMR of compound **B6** measured in $CDCl_3$.*

6th deprotection (B6_deprotected)

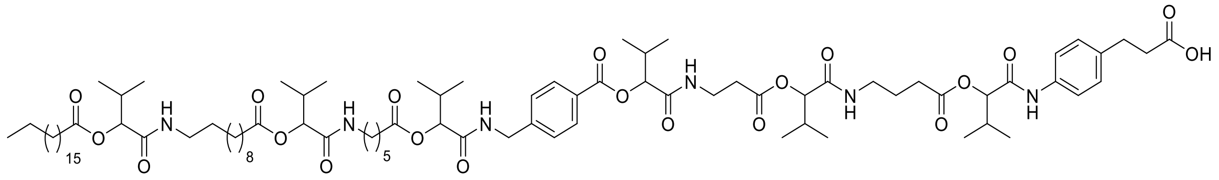

*Supplementary Figure 100. Chemical structure of compound **B6_deprotected**.*

Substance **B6** (1.15 g, 0.670 mmol, 1.00 eq.) was dissolved in THF (5.0 mL, 0.15 M). Subsequently, palladium on activated charcoal (115 mg, 10 wt%.) was suspended in the solution. The reaction mixture was purged with hydrogen (3 balloons) and stirred under hydrogen atmosphere overnight. The heterogeneous catalyst was filtered off and the solvent was evaporated under reduced pressure to obtain the desired product **B6_deprotected** in a yield of 99% (1.07 g, 0.663 mmol) as a highly viscous oil.

^{1}H NMR (500 MHz, $CDCl_3$) δ / ppm: 9.12 – 8.78 (m, 1H, NH, ^{1}), 8.10 – 7.82 (m, 2H, CH aromatic, ^{2}), 7.59 – 7.42 (m, 2H, CH aromatic, ^{3}), 7.38 – 7.27 (m, 2H, CH aromatic, ^{4}), 7.15 – 7.04 (m, 2H, CH aromatic, ^{5}), 7.05 – 6.78 (m, 3H, NH, ^{6}), 6.26 – 5.99 (m, 2H, NH, ^{7}), 5.25 – 5.04 (m, 2H, CH, ^{8}), 5.04- -4.96 (m, 2H, CH_2, ^{9}), 4.96 – 4.70 (m, 4H, CH, ^{8}), 4.63 – 4.38 (m, 2H, CH_2, ^{10}), 4.10 – 2.68 (m, 8H, CH_2, ^{11}), 2.66 – 2.13 (m, 18H, CH, CH_2, ^{12}), 2.10 – 1.53 (m, 8H, CH_2, ^{13}), 1.53 – 1.40 (m, 4H, CH_2, ^{14}), 1.39 – 1.14 (m, 42H, CH_2, ^{15}), 1.08 – 0.73 (m, 39H, CH_3, ^{16}).

^{13}C NMR (126 MHz, $CDCl_3$) δ / ppm: 202.62, 175.37, 173.24, 172.96, 172.78, 170.28, 170.21, 170.14, 170.09, 169.80, 169.59, 168.34, 144.38, 135.91, 130.27, 130.24, 130.17, 130.12, 128.79, 128.73, 128.69, 127.77, 127.60, 121.07, 121.04, 120.96, 120.46, 107.73, 106.47, 103.91, 100.10, 78.98, 78.85, 78.74, 78.68, 78.59, 78.51, 78.30, 78.27, 78.02, 77.97, 77.41, 77.16, 76.91, 68.66, 68.06, 67.76, 67.54, 67.44, 67.13, 67.06, 66.13, 42.87, 41.19, 39.29, 38.87, 38.82, 38.21, 35.61, 34.92, 34.79, 34.41, 34.34, 34.16, 33.99, 33.96, 32.55, 32.39, 32.38, 32.02, 31.19, 30.87, 30.75, 30.71, 30.61, 30.50, 30.33, 29.79, 29.77, 29.75, 29.70, 29.64, 29.60, 29.56, 29.52, 29.45, 29.43, 29.37, 29.29, 29.25, 29.20, 29.11, 29.07, 29.01, 27.91, 26.90, 26.17, 26.11, 26.05, 25.70, 25.13, 25.06, 24.91, 24.35, 23.93, 23.91, 23.54, 23.50, 23.42, 22.79, 22.76, 22.28, 18.97, 18.90, 18.87, 17.64, 17.48, 17.40, 17.34, 17.28, 17.18, 17.11, 17.04, 16.91, 16.61, 16.58, 14.23.

HRMS-ESI-MS $[M+H]^+$of $[C_{89}H_{144}N_6O_{20}]$: calculated: 1618.0508 found: 1618.0494.

IR (ATR platinum diamond): ν / cm^{-1} = 3312.2, 2963.9, 2924.7, 2854.0, 1733.1, 1655.7, 1611.9, 1533.8, 1463.7, 1415.7, 1370.0, 1246.4, 1164.3, 1106.0, 1018.0, 923.1, 837.1, 635.2, 410.5.

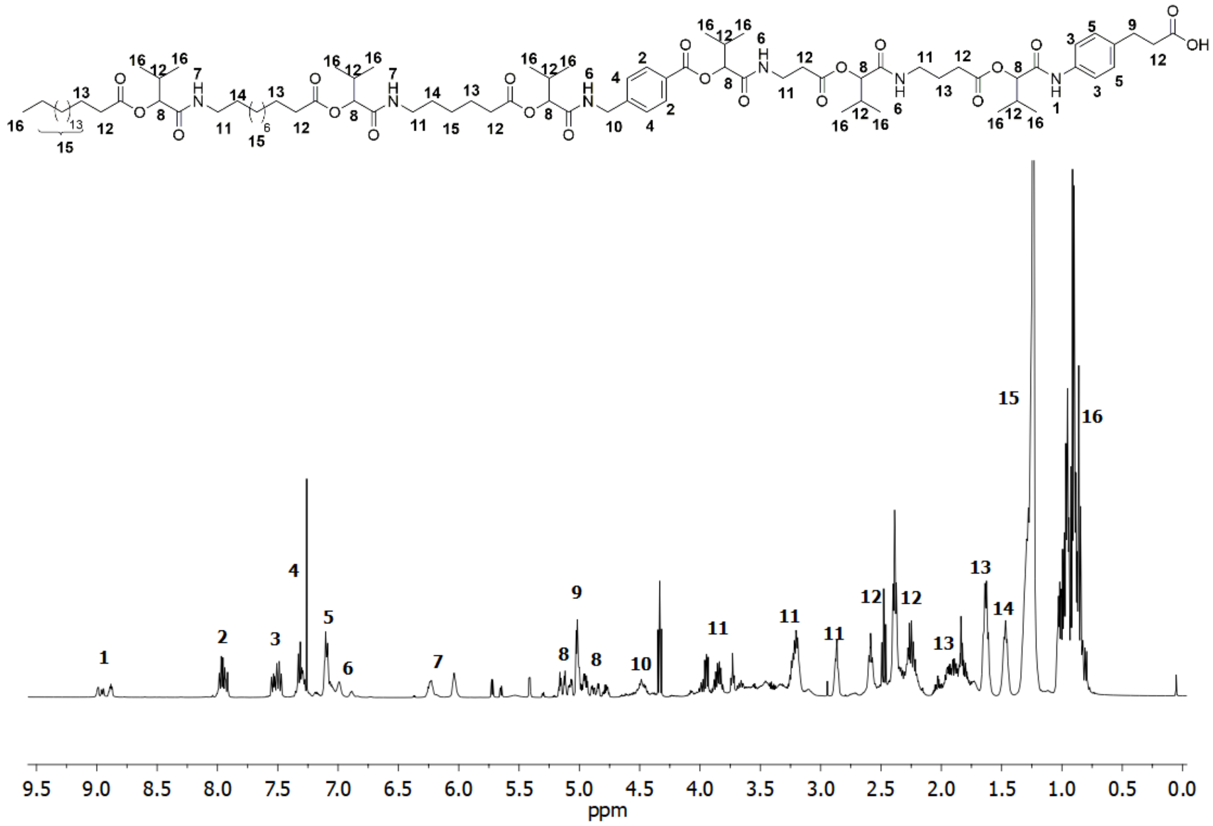

Supplementary Figure 101. Proton NMR of compound ***B6_deprotected*** *measured in $CDCl_3$.*

7th Passerini reaction (B7)

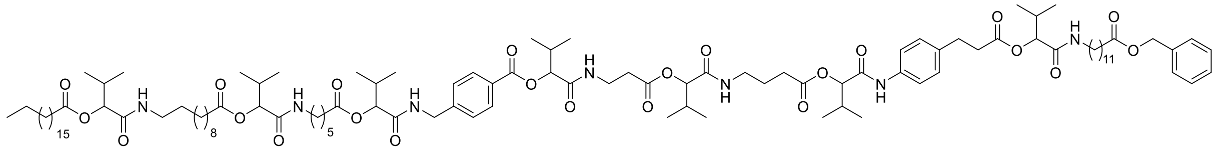

*Supplementary Figure 102. Chemical structure of compound **B7**.*

Substance **B6_deprotected** (0.989 g, 0.607 mmol, 1.00 eq.) was dissolved in DCM (2.0 mL, 0.3 M). Subsequently, isobutyraldehyde **2a** (79.0 µL, 131 mg, 1.82 mmol, 3.00 eq.) and monomer **M3** (0.57 g, 1.82 mmol, 3.00 eq.) were added and the reaction mixture was stirred at room temperature for 48 hours. The solvent was removed under reduced pressure and the crude product was purified by column chromatography (cyclohexane / ethyl acetate 3:1 → 1:5, with 5% of triethyl amine) to obtain the desired product **B7** in a yield of 92% (1.12 g, 0.557 mmol) as a highly viscous oil.

^{1}H NMR (400 MHz, $CDCl_3$) δ / ppm: 9.16 – 8.92 (m, 1H, NH, [1]), 8.03 – 7.84 (m, 2H, CH aromatic, [2]), 7.66–- 7.47 (m, 2H, CH aromatic, [3]), 7.38 – 7.22 (m, 7H, CH aromatic, [4]), 7.18 – 7.06 (m, 2H, CH aromatic, [5]), 7.06 – 6.81 (m, 3H, NH, [6]), 6.25 – 5.78 (m, 3H, NH, [7]), 5.21 – 5.11 (m, 2H, CH, [8]), 5.07 (s, 2H, CH_2, [9]), 5.03 – 4.73 (m, 7H, CH, CH_2, [8]), 4.61 – 4.31 (m, 2H, CH_2, [10]), 3.75 – 2.65 (m, 10H, CH_2, [11]), 2.65 – 2.07 (m, 21H, CH, CH_2, [12]), 1.92 – 1.53 (m, 10H, CH_2, [13]), 1.53 – 1.36 (m, 6H, CH_2, [14]), 1.35 – 1.12 (m, 56H, CH_2, [15]), 1.11 – 0.66 (m, 45H, CH_3, [16]).

^{13}C NMR (101 MHz, $CDCl_3$) δ / ppm: 173.81, 172.70, 171.77, 169.95, 169.59, 169.44, 169.21, 136.16, 130.19, 128.59, 128.50, 128.22, 128.20, 120.98, 78.23, 77.92, 77.36, 66.12, 60.47, 39.21, 35.68, 34.38, 34.36, 34.31, 33.98, 31.98, 30.86, 30.68, 30.58, 30.31, 29.75, 29.71, 29.66, 29.63, 29.53, 29.48, 29.46, 29.41, 29.33, 29.29, 29.26, 29.21, 29.17, 26.88, 25.10, 25.03, 25.00, 22.75, 21.12, 18.95, 18.88, 18.85, 18.79, 17.31, 17.07, 17.00, 14.26, 14.19.

HRMS-ESI-MS $[M+H]^+$ of $[C_{113}H_{181}N_7O_{23}]$: calculated: 2005.3282 found: 2005.3289.

IR (ATR platinum diamond): ν / cm^{-1} = 3301.4, 2963.7, 2924.9, 2853.8, 1737.3, 1654.8, 1611.7, 1531.4, 1463.8, 1415.8, 1369.9, 1244.9, 1159.2, 1126.1, 1106.0, 1003.4, 834.1, 697.4, 636.1, 412.4.

R_f: (cyclohexane / ethyl acetate 1:4, with 4% triethyl amine) = 0.45.

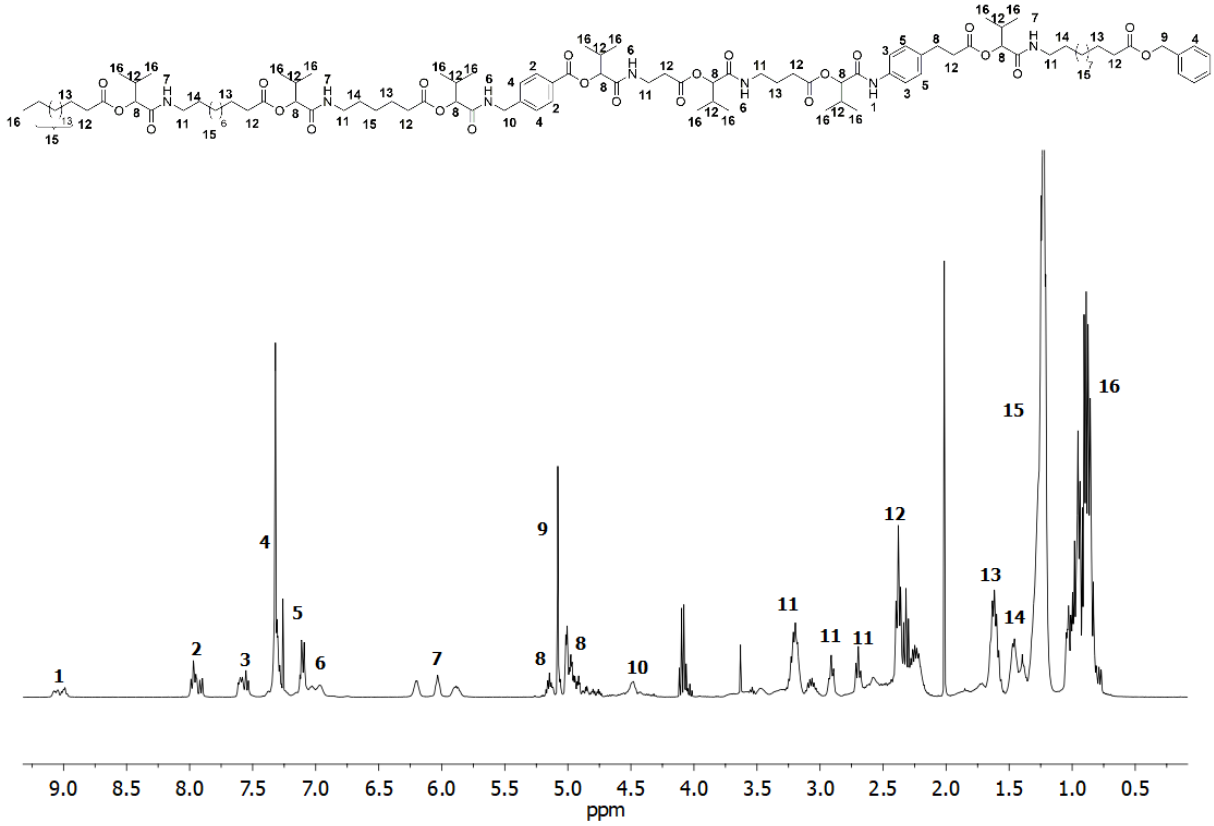

*Supplementary Figure 103. Proton NMR of compound **B7** measured in $CDCl_3$.*

7th deprotection (B7_deprotected)

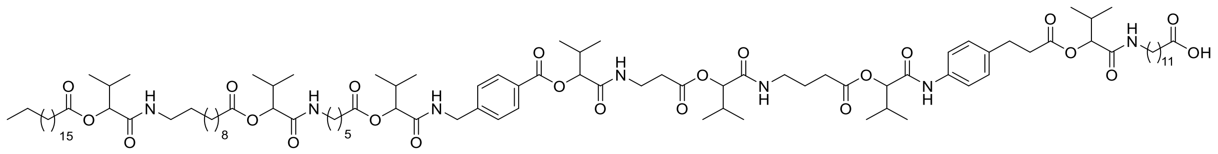

*Supplementary Figure 104. Chemical structure of compound **B7_deprotected**.*

Substance **B7** (0.994 g, 0.495 mmol, 1.00 eq.) was dissolved in THF (2.5 mL, 0.2 M). Subsequently, palladium on activated charcoal (994 mg, 10 wt%.) was suspended in the solution. The reaction mixture was purged with hydrogen (4 balloons) and stirred under hydrogen atmosphere overnight. The heterogeneous catalyst was filtered off and the solvent was evaporated under reduced pressure to obtain the desired product **B7_deprotected** in a yield of 81% (760 mg, 0.397 mmol) as a highly viscous oil.

^{1}H NMR (400 MHz, $CDCl_3$) δ / ppm: 9.14 – 8.90 (m, 1H, NH, [1]), 8.04 – 7.84 (m, 2H, CH aromatic, [2]), 7.62 – 7.48 (m, 2H, CH aromatic, [3]), 7.38 – 7.22 (m, 2H, CH aromatic, [4]), 7.21 – 6.98 (m, 5H, NH, CH aromatic, [5]), 6.29 – 5.78 (m, 3H, NH, [6]), 5.19 – 4.68 (m, 9H, CH, CH_2, [7]), 4.63 – 4.37 (m, 2H, CH_2, [8]), 3.29 - 2.54 (m, 10H, CH_2, [9]), 2.39 – 1.86 (m, 21H, CH, CH_2, [10]), 1.84 – 1.50 (m, 10H, CH_2, [11]), 1.49 – 1.33 (m, 6H, CH_2, [12]), 1.33 – 1.04 (s, 56H, CH_2, [13]), 1.04 – 0.67 (m, 45H, CH_3, [14]).

^{13}C NMR (101 MHz, $CDCl_3$) δ / ppm: 176.55, 172.89, 172.73, 171.78, 170.01, 169.70, 169.53, 169.33, 130.09, 130.00, 128.55, 128.46, 127.52, 120.97, 107.88, 107.62, 106.37, 78.48, 78.19, 77.88, 68.61, 67.67, 67.60, 67.44, 42.77, 39.19, 38.72, 35.61, 34.30, 34.23, 33.95, 32.31, 31.92, 30.79, 30.52, 30.25, 29.70, 29.65, 29.61, 29.54, 29.50, 29.47, 29.43, 29.36, 29.28, 29.20, 29.16, 29.11, 29.00, 27.83, 26.82, 26.74, 26.08, 25.03, 24.97, 24.81, 24.26, 23.93, 23.84, 23.83, 22.69, 22.18, 18.83, 18.78, 18.73, 17.26, 17.04, 16.96, 14.14.

HRMS-ESI-MS $[M+H]^+$of $[C_{106}H_{175}N_7O_{23}]$: calculated: 1915.2812 found: 1915.2852.

IR (ATR platinum diamond): ν / cm^{-1} = 3335.9, 2928.1, 1727.7, 1659.9, 1537.5, 1460.2, 1369.7, 1240.9, 1175.8, 1066.1, 1034.7, 990.8, 924.1, 850.1, 534.5, 410.3.

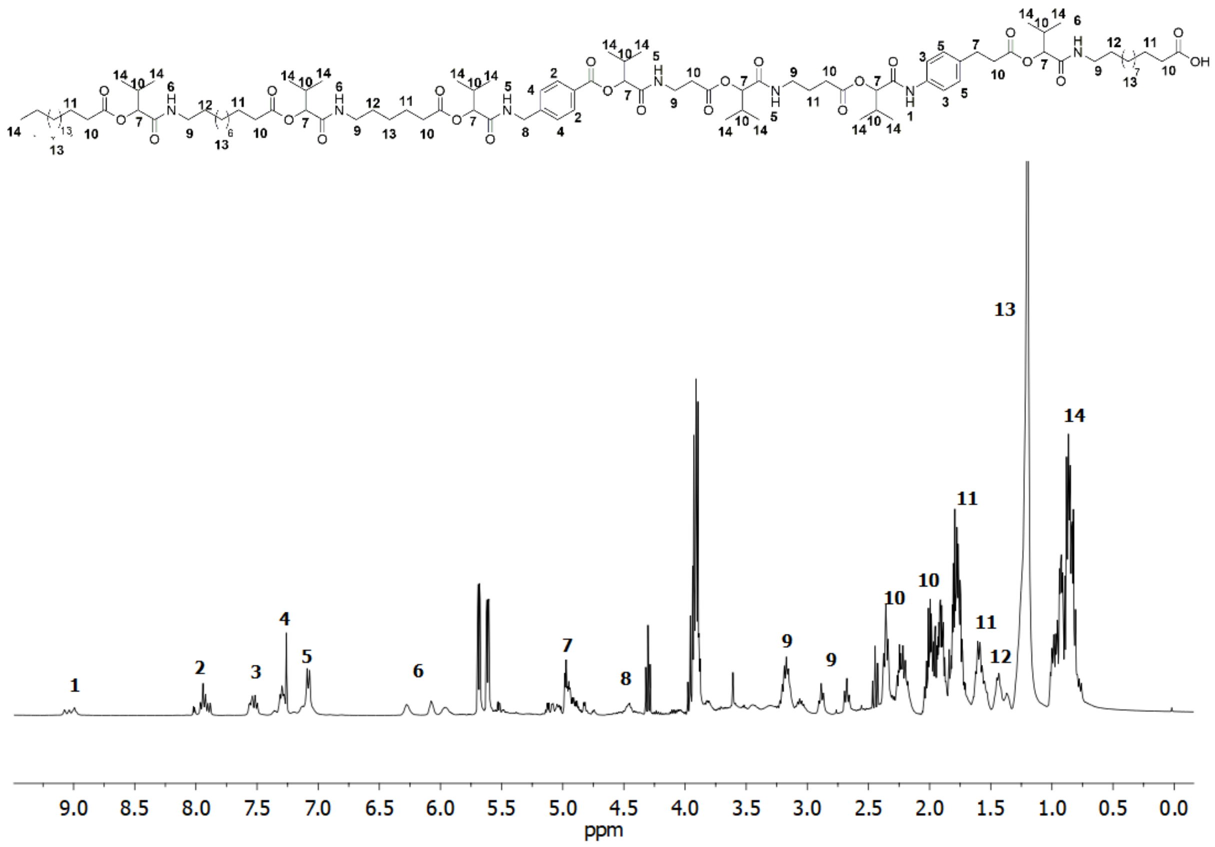

*Supplementary Figure 105. Proton NMR of compound **B7_deprotected** measured in $CDCl_3$.*

1.3.4.2 Summary of the synthesis of the backbone defined oligomer – SEC and ESI-MS characterisation

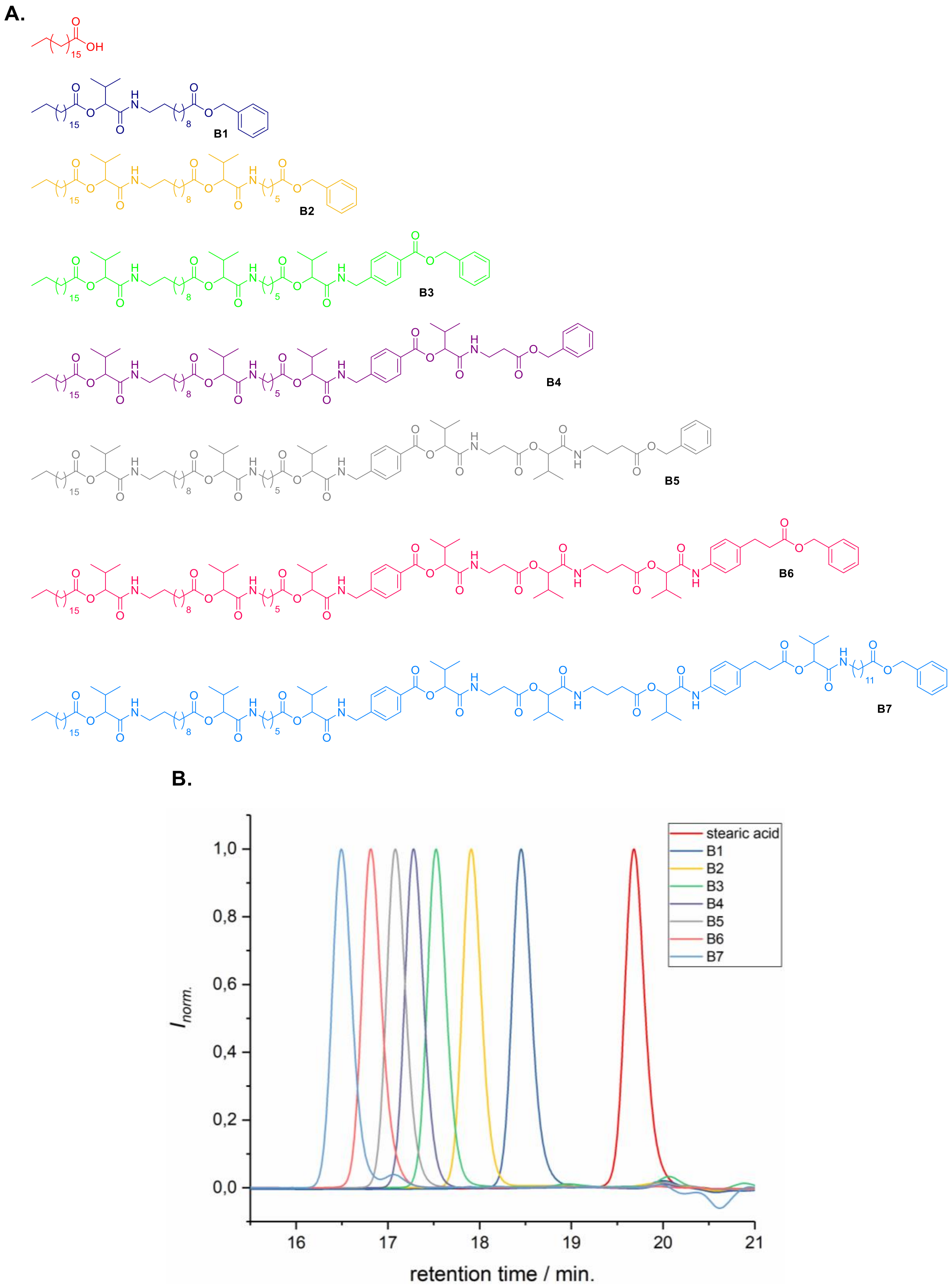

Supplementary Figure 106 A: Structure of the growing oligomers after each P-3CR up to the heptamer stage, where the monomers were inserted step by step in the following order: ***M1***, ***M2***, ***M9***, ***M4***, ***M5***, ***M7***, *and* ***M3***. *B. SEC results after each P-3CR verify the purity of the products. For the assignment of the oligomers, refer to the colour code.*

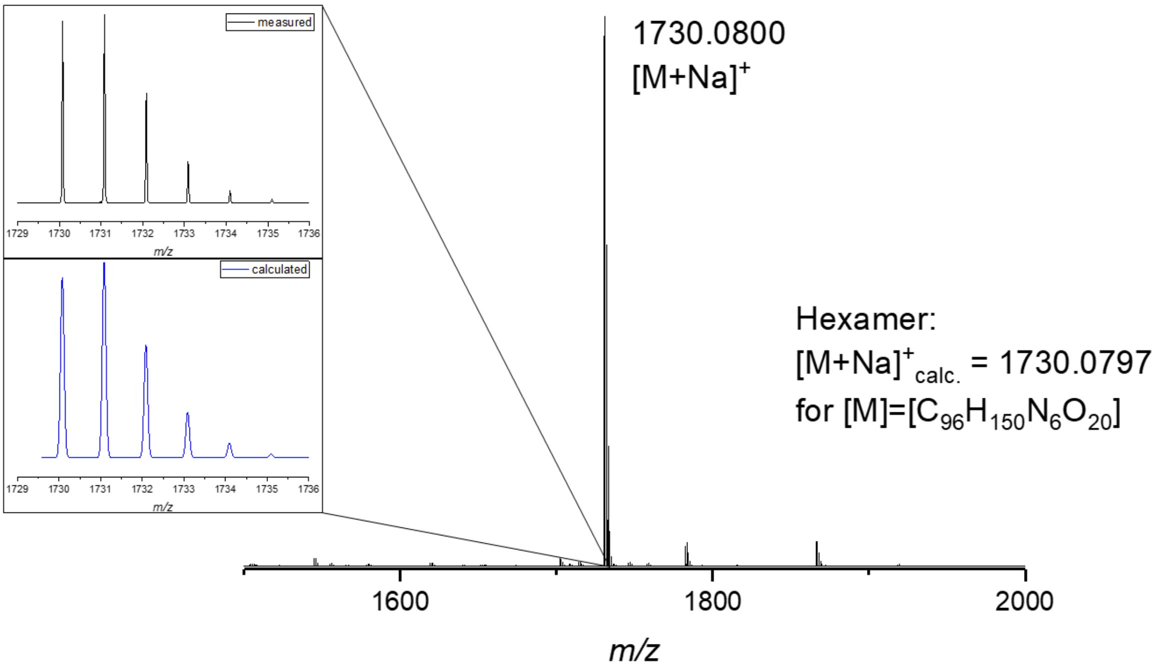

*Supplementary Figure 107. Mass spectrum of the backbone-defined hexamer **B6**: the singly charged sodium ion was found to be the main peak, further indicating high purity of the product. The measured isotopic pattern (black) can be compared with the calculated one obtained by mMass (blue), revealing that they are in good agreement.*

Supplementary Table 2. Summary of the backbone-defined oligomer synthesis.

Product	Applied monomer	Yield [%]	$m/z_{\text{calc.}}$	m/z_{found}
1st P-3CR **B1**	**M1**	98[a]	658.5405	658.5404
1st deprotection		87[b]	568.4936	568.4935
2nd P-3CR **B2**	**M2**	98[a]	871.6770	871.6764
2nd deprotection		93[b]	781.6300	781.6297
3rd P-3CR **B3**	**M9**	99[a]	1104.7822	1104.7826
3rd deprotection		81[b]	1014.7352	1014.7343
4th P-3CR **B4**	**M4**	92[a]	1275.8717	1275.8743
4th deprotection		99[b]	1185.8248	1185.8254
5th P-3CR **B5**	**M5**	82[a]	1460.9769	1460.9792
5th deprotection		97[b]	1370.9300	1370.9318
6th P-3CR **B6**	**M7**	85[a]	1708.0978	1708.0978
6th deprotection		99[b]	1618.0508	1618.0494
7th P-3CR **B7**	**M3**	92[a]	2005.3282	2005.3289
7th deprotection		81[b]	1915.2812	1915.2852
Overall yield	31% over 14 reaction steps			

[a] *after column chromatography,* [b] *after filtration.*

1.3.4.3 Dual sequence-definition

1st Passerini reaction (DS1)

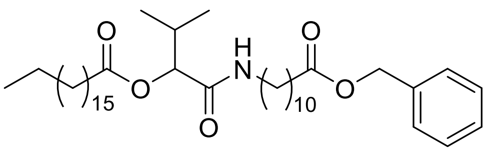

*Supplementary Figure 108. Chemical structure of compound **DS1**.*

Stearic acid **1** (1.50 g, 5.27 mmol, 1.00 eq.) was suspended in DCM (5.3 mL, 1. M). Subsequently, isobutyraldehyde **2a** (721 µL, 570 mg, 7.91 mmol, 1.50 eq.) and monomer **M1** (2.38 g, 7.91 mmol, 1.50 eq.) were added and the reaction mixture was stirred at room temperature for 24 hours. The solvent was removed under reduced pressure and the crude product was purified by column chromatography (cyclohexane / ethyl acetate 15:1 → 11:1) to obtain the desired product **DS1** in a yield of 98% (3.40 g, 5.17 mmol) as a white solid.

^{1}H NMR (400 MHz, $CDCl_3$) δ / ppm: 7.40 – 7.28 (m, 5H, CH aromatic, [1]), 5.96 (t, J = 5.5 Hz, 1H, NH, [2]), 5.10 (s, 2H, CH_2, [3]), 5.06 (d, J = 4.4 Hz, 1H, CH, [4]), 3.34 – 3.15 (m, 2H, CH_2, [5]), 2.46 – 2.23 (m, 5H, CH, CH_2, [6]), 1.74 – 1.56 (m, 4H, CH_2, [7]), 1.54 – 1.40 (m, 2H, CH_2, [8]), 1.39 – 1.12 (m, 40H, CH_2, [9]), 0.99 – 0.79 (m, 9H, CH_3, [10]).

^{13}C NMR (101 MHz, $CDCl_3$) δ / ppm: 173.77, 172.65, 169.38, 136.22, 128.64, 128.26, 77.97, 66.16, 39.25, 34.42, 32.03, 30.62, 29.81, 29.78, 29.77, 29.76, 29.71, 29.68, 29.58, 29.54, 29.47, 29.44, 29.38, 29.31, 29.27, 29.20, 26.94, 25.15, 25.04, 22.80, 18.89, 17.03, 14.24.

HRMS-ESI-MS $[M+H]^+$ of $[C_{41}H_{71}NO_5]$: calculated: 658.5405 found: 658.5391.

IR (ATR platinum diamond): ν / cm^{-1} = 3286.0, 2916.0, 2848.6, 1737.5, 1649.3, 1550.4, 1498.2, 1469.6, 1415.9, 1378.9, 1294.7, 1272.1, 1254.6, 1212.7, 1157.5, 1108.5, 1031.7, 1013.0, 986.5, 927.3, 722.0, 693.6, 578.8, 473.8.

R_f: (cyclohexane / ethyl acetate 5:1) = 0.42.

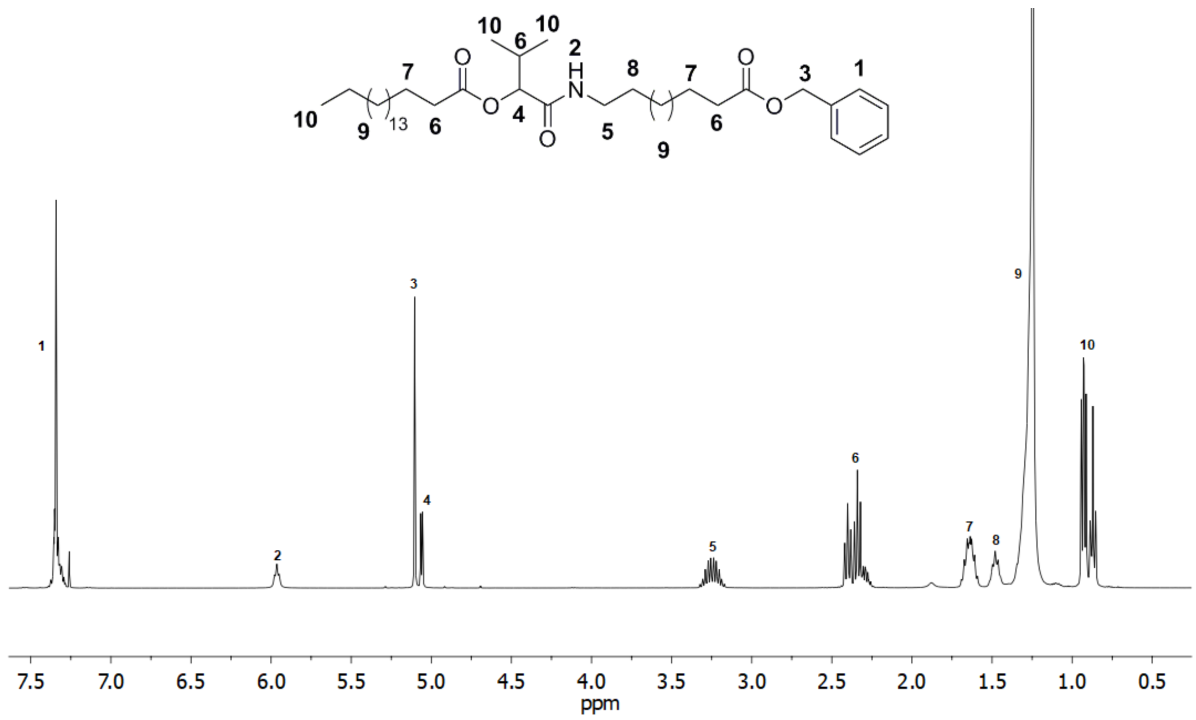

*Supplementary Figure 109. Proton NMR of compound **DS1** measured in $CDCl_3$.*

1st deprotection (DS1_deprotected)

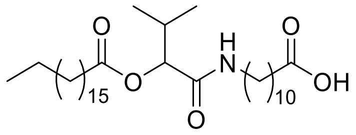

*Supplementary Figure 110. Chemical structure of compound **DS1_deprotected**.*

Substance **DS1** (3.31 g, 5.03 mmol, 1.00 eq.) was dissolved in THF (17 mL, 0.3 M). Subsequently, palladium on activated charcoal (331 mg, 10 wt%.) was suspended in the solution. The reaction mixture was purged with hydrogen (3 balloons) and stirred under hydrogen atmosphere overnight. The heterogeneous catalyst was filtered off and the solvent was evaporated under reduced pressure to obtain the desired product **DS1_deprotected** in a yield of 99% (2.68 g, 4.98 mmol) as a white solid.

^{1}H NMR (400 MHz, $CDCl_3$) δ / ppm: 5.99 (t, J = 5.1 Hz, 1H, NH, ^{1}), 5.06 (d, J = 4.4 Hz, 1H, CH, ^{2}), 3.40 – 3.14 (m, 2H, CH_2, ^{3}), 2.45 – 2.23 (m, 5H, CH, CH_2, ^{4}), 1.70 – 1.54 (m, 4H, CH_2, ^{5}), 1.52 – 1.37 (m, 2H, CH_2, ^{6}), 1.36 – 1.15 (m, 40, CH_2H, ^{7}), 0.97 – 0.73 (m, 9H, CH_2, ^{8}).

^{13}C NMR (101 MHz, $CDCl_3$) δ / ppm: 179.08, 172.74, 169.58, 128.27, 108.06, 107.73, 106.46, 77.98, 68.65, 67.80, 67.77, 67.55, 39.30, 34.42, 34.08, 32.04, 30.61, 29.81, 29.77, 29.72, 29.61, 29.58, 29.48, 29.38, 29.36, 29.27, 29.23, 29.09, 27.92, 26.90, 25.15, 24.79, 24.03, 23.94, 23.92, 22.81, 22.30, 18.89, 17.03, 14.24.

HRMS-ESI-MS $[M+H]^+$ of $[C_{34}H_{65}NO_5]$: calculated: 568.4936 found: 568.4922.

IR (ATR platinum diamond): ν / cm^{-1} = 3287.3, 2916.4, 2848.9, 1744.5, 1704.0, 1651.1, 1551.1, 1468.7, 1435.6, 1378.0, 1294.9, 1272.2, 1253.7, 1233.6, 1214.4, 1190.8, 1159.5, 1108.4, 1069.6, 1035.5, 991.7, 928.2, 722.1, 685.6.

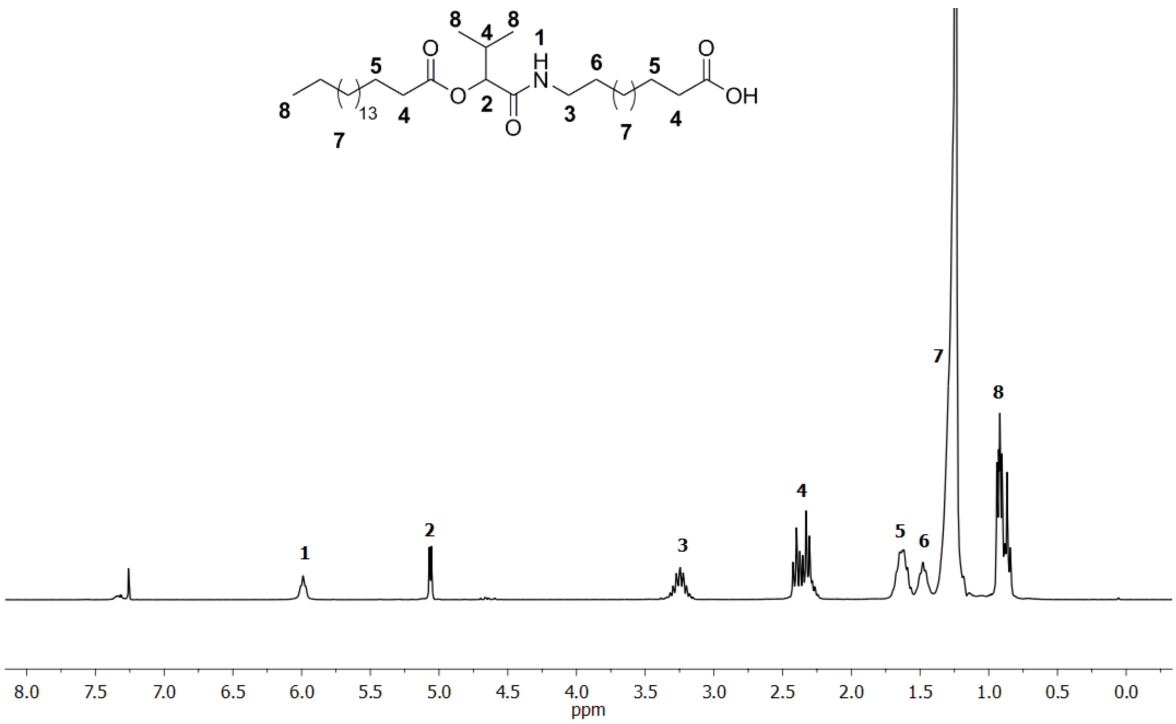

Supplementary Figure 111. Proton NMR of compound ***DS1_deprotected*** *measured in* $CDCl_3$.

2nd Passerini reaction (DS2)

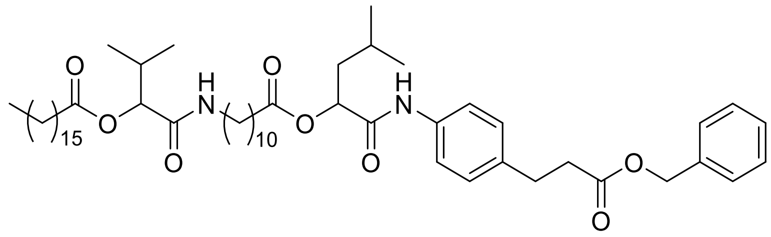

Supplementary Figure 112. Chemical structure of compound ***DS2****.*

Substance **DS1_deprotected** (3.25 g, 6.03 mmol, 1.00 eq.) was dissolved in DCM (12 mL, 0.5 M). Subsequently, isovaleraldehyde **2d** (969 µL, 778 mg, 9.04 mmol, 1.50 eq.) and monomer **M7** (2.40 g, 9.04 mmol, 1.50 eq.) were added and the reaction mixture was stirred at room temperature for 24 hours. The solvent was removed under reduced pressure and the crude product was purified by column chromatography (cyclohexane / ethyl acetate 15:1 → 2:1) and a second time using another gradient (cyclohexane / ethyl acetate 20:1 → 2:1, with 5% of triethyl amine) to obtain the desired product **DS2** in a yield of 65% (3.50 g, 3.93 mmol) as a highly viscous oil.

^{1}H NMR (400 MHz, $CDCl_3$) δ / ppm: 7.89 (s, 1H, NH, [1]), 7.50 – 7.04 (m, 9H, CH aromatic, [2]), 6.02 (s, 1H, NH, [3]), 5.36 – 5.24 (m, 1H, CH, [4]), 5.08 (s, 2H, CH_2, [5]), 5.05 (d, J = 4.4 Hz, 1H, CH, [6]), 3.33 – 3.15 (m, 2H, CH_2, [7]), 2.91 (t, J = 7.6 Hz, 2H, CH_2, [8]), 2.68 – 2.56 (m, 2H, CH_2, [9]), 2.45 – 2.22 (m, 6H, CH, CH_2, [10]), 1.91 – 1.59 (m, 6H, CH_2, [11]), 1.47 – 1.41 (m, 2H, CH_2, [12]), 1.39 – 1.14 (m, 40H, CH_2, [13]), 1.02 – 0.78 (m, 15H, CH_3, [14]).

^{13}C NMR (101 MHz, $CDCl_3$) δ / ppm: 172.86, 172.70, 172.68, 169.41, 168.44, 136.87, 135.90, 135.47, 128.93, 128.80, 128.61, 128.30, 120.51, 120.26, 119.87, 77.95, 77.48, 77.16, 76.84, 72.89, 66.37, 40.79, 39.21, 35.98, 34.37, 34.33, 32.26, 31.99, 30.58, 30.42, 29.77, 29.73, 29.67, 29.62, 29.54, 29.46, 29.43, 29.37, 29.34, 29.22, 29.13, 26.88, 25.11, 24.97, 24.62, 23.53, 23.16, 22.76, 21.91, 18.86, 17.01, 14.21.

HRMS-ESI-MS $[M+H]^+$ of $[C_{56}H_{90}N_2O_8]$: calculated: 919.6770 found: 919.6747.

IR (ATR platinum diamond): ν / cm^{-1} = 3275.3, 2917.5, 2849.8, 1740.5, 1674.9386, 2.4158, 1606.5, 1537.2, 1466.9, 1414.7, 1370.0, 1306.2205, 1251.2, 1213.5, 1159.8, 1069.5, 828.4, 723.4, 697.5, 536.1.

***R*$_f$**: (cyclohexane / ethyl acetate 2:1) = 0.45.

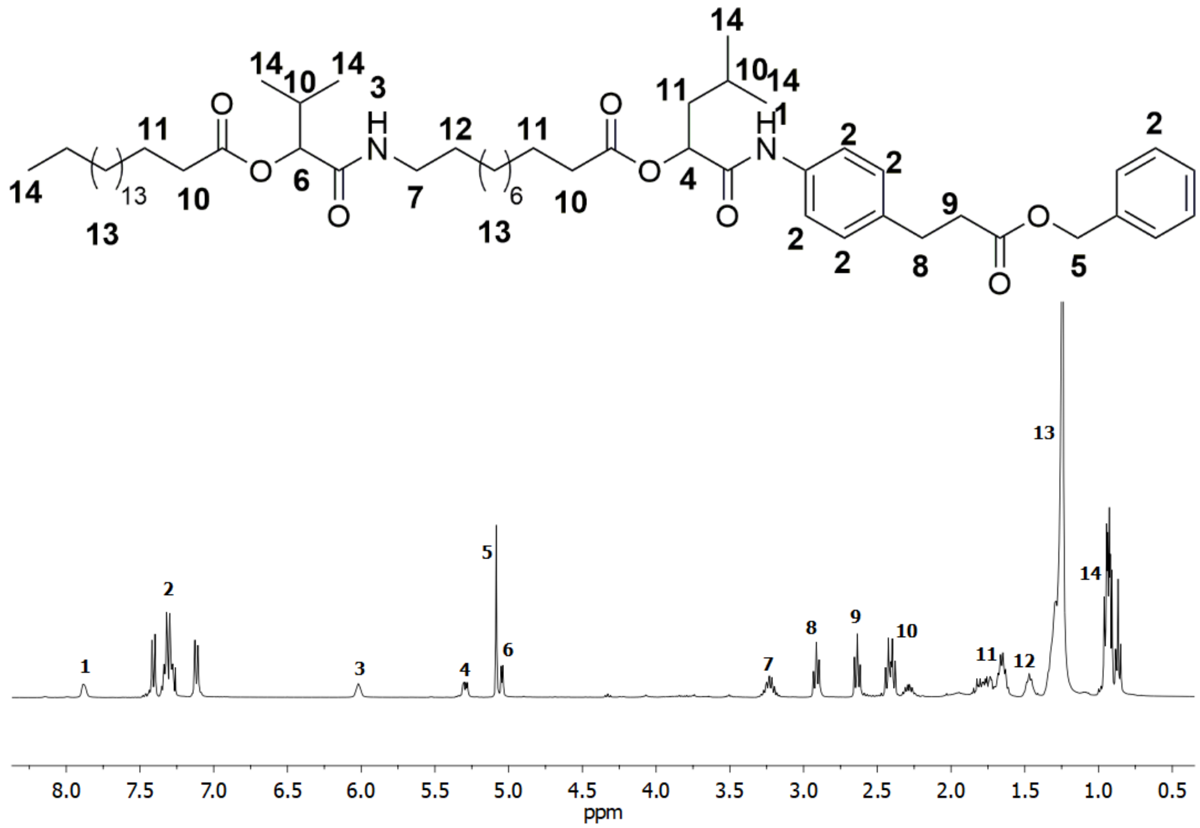

Supplementary Figure 113. Proton NMR of compound ***DS2*** *measured in* $CDCl_3$.

2nd deprotection (DS2_deprotected)

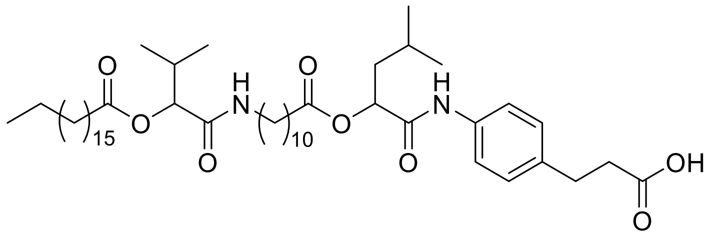

*Supplementary Figure 114. Chemical structure of compound **DS2_deprotected**.*

Substance **DS2** (2.83 g, 3.09 mmol, 1.00 eq.) was dissolved in THF (10.0 mL, 0.3 M). Subsequently, palladium on activated charcoal (283 mg, 10 wt%.) was suspended in the solution. The reaction mixture was purged with hydrogen (4 balloons) and stirred under hydrogen atmosphere overnight. The heterogeneous catalyst was filtered off and the solvent was evaporated under reduced pressure to obtain the desired product **DS2_deprotected** in a yield of 99% (2.45 g, 3.05 mmol) as a highly viscous oil.

^{1}H NMR (400 MHz, $CDCl_3$) δ / ppm: 7.91 (s, 1H, NH, [1]), 7.42 (d, J = 8.4 Hz, 2H, CH aromatic, [2]), 7.14 (d, J = 8.4 Hz, 2H, CH aromatic, [2]), 6.04 (t, J = 4.7 Hz, 1H, NH, [3]), 5.31 – 5.28 (m, 1H, CH, [4]), 5.08 – 4.99 (m, 1H, CH, [5]), 3.37 – 3.14 (m, 2H, CH_2, [6]), 2.90 (t, J = 7.6 Hz, 2H, CH_2, [7]), 2.62 (t, J = 7.6 Hz, 2H, CH_2, [8]), 2.49 – 2.21 (m, 6H, CH, CH_2, [9]), 2.11 – 1.57 (m, 6H, CH_2, [10]), 1.53 – 1.40 (m, 2H, CH_2, [11]), 1.38 – 1.04 (m, 40H, CH_2, [12]), 1.02 – 0.77 (m, 15H, CH_3, [13]).

^{13}C NMR (101 MHz, $CDCl_3$) δ / ppm: 177.35, 173.00, 172.79, 172.77, 169.66, 168.53, 136.89, 135.49, 128.96, 120.33, 107.72, 106.45, 77.96, 77.48, 77.16, 76.84, 72.95, 67.76, 67.54, 40.79, 39.32, 35.64, 34.40, 34.36, 32.02, 30.59, 30.19, 29.80, 29.76, 29.71, 29.59, 29.57, 29.46, 29.37, 29.26, 29.14, 26.88, 25.13, 25.01, 24.64, 23.93, 23.18, 22.79, 21.93, 18.87, 17.03, 14.24.

HRMS-ESI-MS $[M+H]^+$ of [$C_{49}H_{84}N_2O_8$]: calculated: 829.6300 found: 829.6279.

IR (ATR platinum diamond): ν / cm^{-1} = 3277.4, 2917.5, 2849.8, 1742.7, 1673.5, 1652.7, 1607.0, 1538.5, 1465.0, 1415.2, 1369.6, 1309.1, 1252.1, 1162.3, 1111.9, 1066.9, 1035.2, 991.5, 926.3, 834.8, 721.9, 677.9, 531.1.

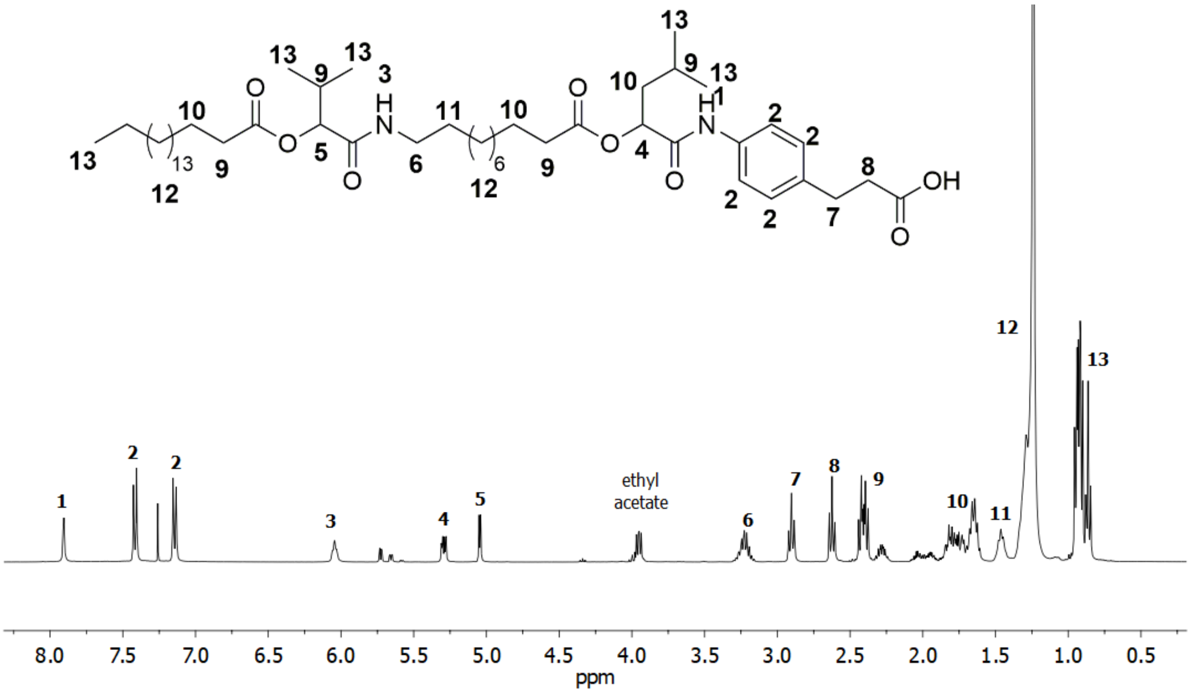

*Supplementary Figure 115. Proton NMR of compound **DS2_deprotected** measured in $CDCl_3$.*

3rd Passerini reaction (DS3)

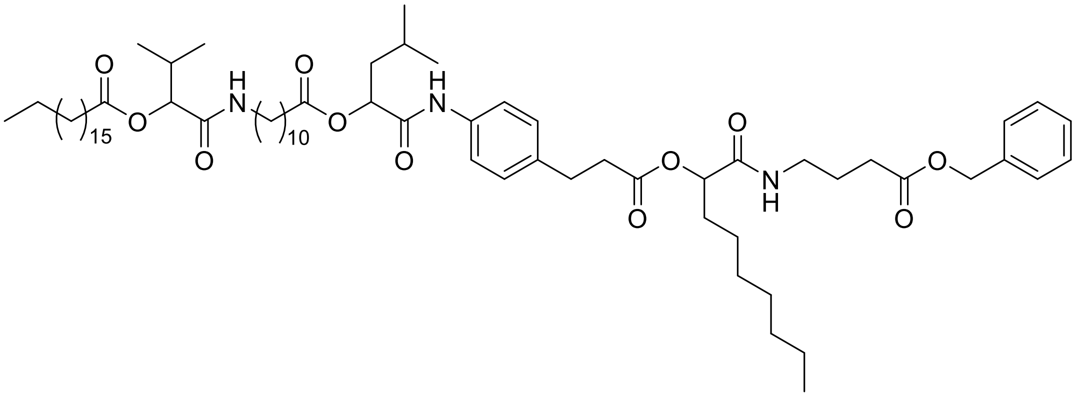

Supplementary Figure 116. Chemical structure of compound ***DS3****.*

Substance **DS2_deprotected** (3.36 g, 4.19 mmol, 1.00 eq.) was dissolved in DCM (8.2 mL, 0.5 M). Subsequently, octanal **2h** (982 µL, 807 mg, 6.30 mmol, 1.50 eq.) and monomer **M5** (1.28 g, 6.30 mmol, 1.50 eq.) were added and the reaction mixture was stirred at room temperature for 48 hours. The solvent was removed under reduced pressure and the crude product was purified by column chromatography (cyclohexane / ethyl acetate 15:1 → 2:1, with 5% triethyl amine) to obtain the desired product **DS3** in a yield of 47% (2.24 g, 1.93 mmol) as a highly viscous oil.

^{1}H NMR (400 MHz, $CDCl_3$) δ / ppm: 8.05 (s, 1H, NH, [1]), 7.54 – 7.05 (m, 9H, CH aromatic, [2]), 6.22 – 5.90 (m, 2H, NH, [3]), 5.29 – 5-25 (m, 1H, CH, [4]), 5.15 – 5.06 (s, 2H, CH_2, [5]), 5.03 (d, J = 4.3 Hz, 2H, CH, [6]), 3.38 – 3.05 (m, 2H, CH_2, [7]), 3.06 – 2.90 (m, 2H, CH_2, [8]), 2.75 – 2.61 (m, 2H, CH_2, [9]), 2.50 – 2.15 (m, 8H, CH, CH_2, [10]), 2.02 – 1.55 (m, 10H, CH_2, [11]), 1.54 – 1.37 (m, 2H, CH_2, [12]), 1.36 – 1.04 (m, 50H, CH_2, [13]), 1.01 – 0.66 (m, 18H, CH_3, [14]).

^{13}C NMR (101 MHz, $CDCl_3$) δ / ppm: 173.58, 172.90, 172.68, 172.66, 171.64, 170.04, 169.38, 168.60, 136.41, 135.78, 135.71, 128.78, 128.64, 128.36, 128.18, 120.42, 77.90, 74.08, 72.81, 66.94, 66.51, 40.75, 39.18, 38.65, 35.57, 34.33, 34.24, 32.34, 31.95, 31.77, 31.66, 30.54, 30.21, 29.73, 29.69, 29.64, 29.59, 29.50, 29.42, 29.40, 29.35, 29.31, 29.19, 29.10, 29.08, 26.85, 25.07, 24.91, 24.82, 24.58, 24.33, 23.51, 23.13, 22.73, 22.64, 21.84, 18.83, 16.98, 14.18, 14.14.

HRMS-ESI-MS $[M+H]^+$ of $[C_{69}H_{113}N_3O_{11}]$: calculated: 1160.8448 found: 1160.8435.

IR (ATR platinum diamond): ν / cm^{-1} = 3293.8, 2921.9, 2852.4, 1738.6, 1653.7, 1608.5, 1533.4, 1464.9, 1414.8, 1370.0, 1243.4, 1160.9, 1067.5, 1001.6732, 829.1, 722.3, 697.0, 528.5.

***R*$_f$**: (cyclohexane / ethyl acetate 7:3, with 5% triethyl amine) = 0.38.

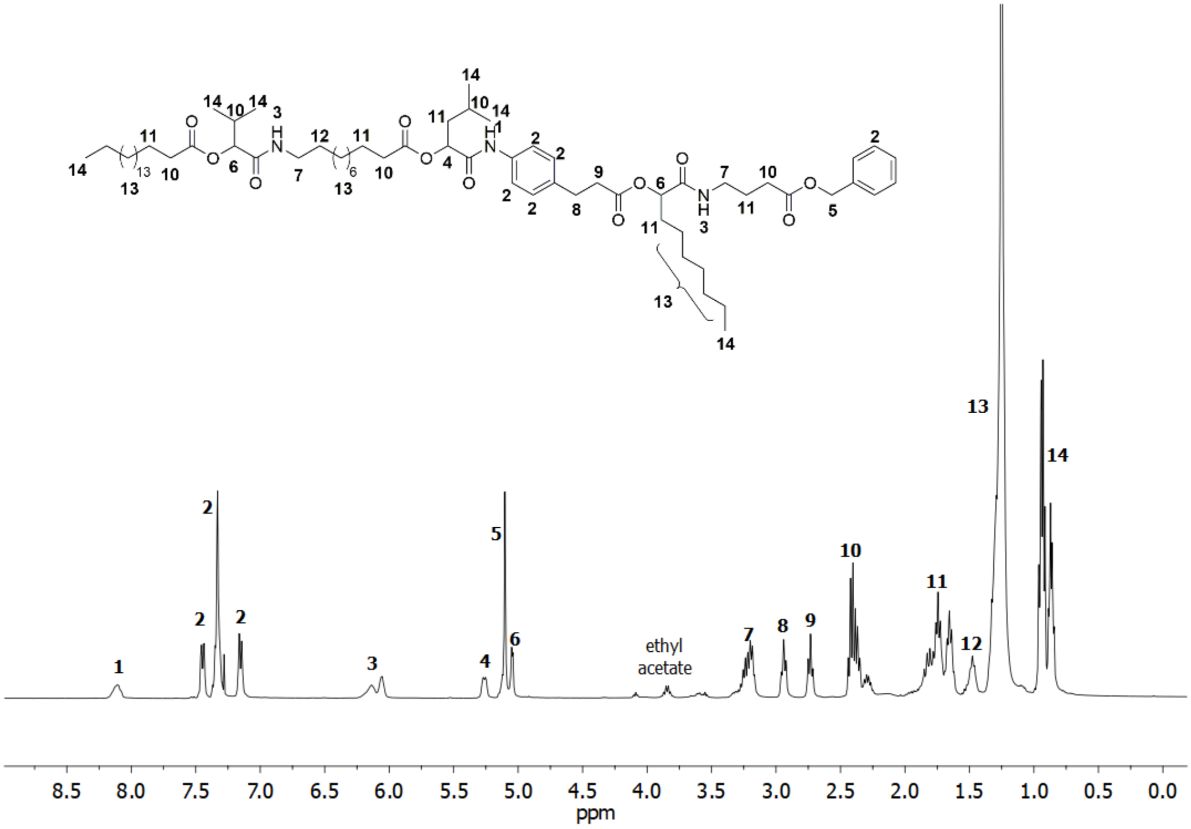

*Supplementary Figure 117. Proton NMR of compound **DS3** measured in $CDCl_3$.*

3rd deprotection (DS3_deprotected)

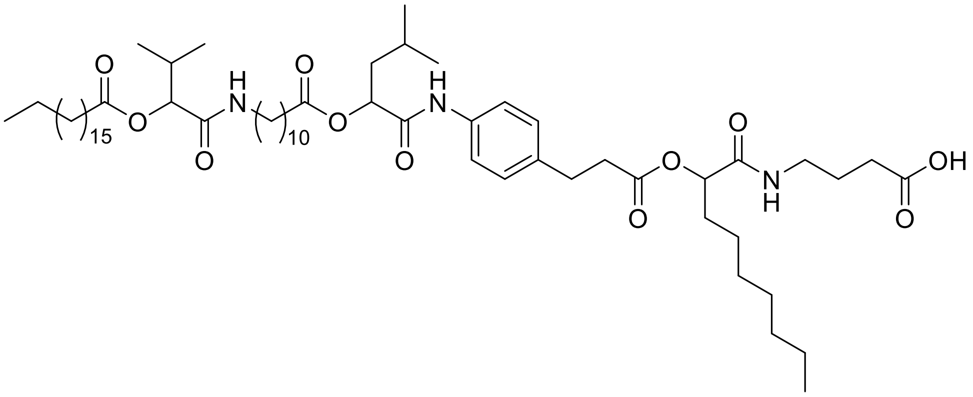

*Supplementary Figure 118. Chemical structure of compound **DS3_deprotected**.*

Substance **DS3** (2.20 g, 2.02 mmol, 1.00 eq.) was dissolved in THF (10 mL, 0.2 M). Subsequently, palladium on activated charcoal (202 mg, 10 wt%.) was suspended in the solution. The reaction mixture was purged with hydrogen (4 balloons) and stirred under hydrogen atmosphere overnight. The heterogeneous catalyst was filtered off and the solvent was evaporated under reduced pressure to obtain the desired product **DS3_deprotected** in a yield of 99% (2.08 g, 2.00 mmol) as a highly viscous oil.

^{1}H NMR (400 MHz, $CDCl_3$) δ / ppm: 8.15 (s, 1H, NH, ^{1}), 7.50 – 7.00 (m, 4H, CH aromatic, ^{2}), 6.26 – 5.99 (m, 2H, NH, ^{3}), 5.28 – 5.20 (m, 1H, CH, ^{4}), 5.13 – 4.96 (m, 2H, CH, ^{5}), 3.32 – 3.04 (m, 2H, CH_2, ^{6}), 2.92 (t, J = 7.1 Hz, 2H, CH_2, ^{7}), 2.72 (t, J = 7.2 Hz, 2H, CH_2, ^{8}), 2.59 – 2.14 (m, 8H, CH, CH_2, ^{9}), 2.12 – 1.52 (m, 10H, CH_2, ^{10}), 1.51 – 1.37 (m, 2H, CH_2, ^{11}), 1.36 – 1.02 (m, 50H, CH_2, ^{12}), 1.00 – 0.71 (m, 18H, CH_3, ^{13}).

^{13}C NMR (101 MHz, $CDCl_3$) δ / ppm: 176.43, 173.39, 172.75, 171.79, 170.49, 169.69, 168.80, 136.53, 135.73, 128.82, 120.64, 107.68, 106.42, 77.89, 74.13, 72.90, 67.73, 67.50, 40.74, 39.28, 38.65, 35.48, 34.37, 34.30, 31.98, 31.94, 31.80, 31.31, 30.58, 30.19, 29.76, 29.72, 29.67, 29.59, 29.54, 29.42, 29.34, 29.22, 29.17, 29.11, 29.09, 26.83, 25.10, 24.91, 24.89, 24.61, 24.37, 23.90, 23.17, 22.76, 22.67, 21.82, 18.85, 16.99, 14.20, 14.16.

HRMS-ESI-MS $[M+H]^+$ of $[C_{62}H_{107}N_3O_{11}]$: calculated: 1070.7978 found: 1070.7958.

IR (ATR platinum diamond): ν / cm^{-1} = 3314.0, 2923.6, 2853.7, 1738.5, 1657.5, 1609.1, 1536.9, 1462.9, 1415.0, 1369.6, 1241.5, 1166.9, 1121.6, 1067.4905, 1036.0, 992.3, 928.4, 833.7, 721.5, 531.3, 411.6.

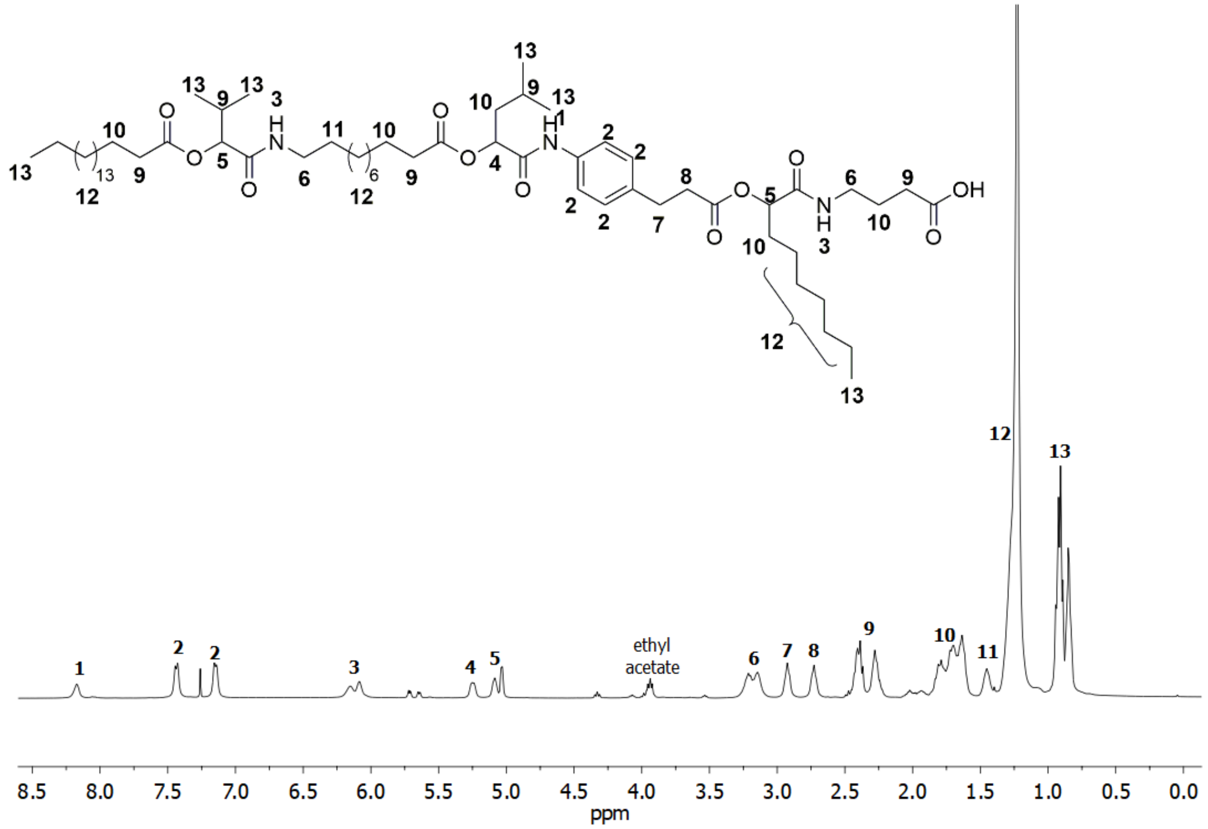

*Supplementary Figure 119. Proton NMR of compound **DS3_deprotected** measured in $CDCl_3$.*

4th Passerini reaction (DS4)

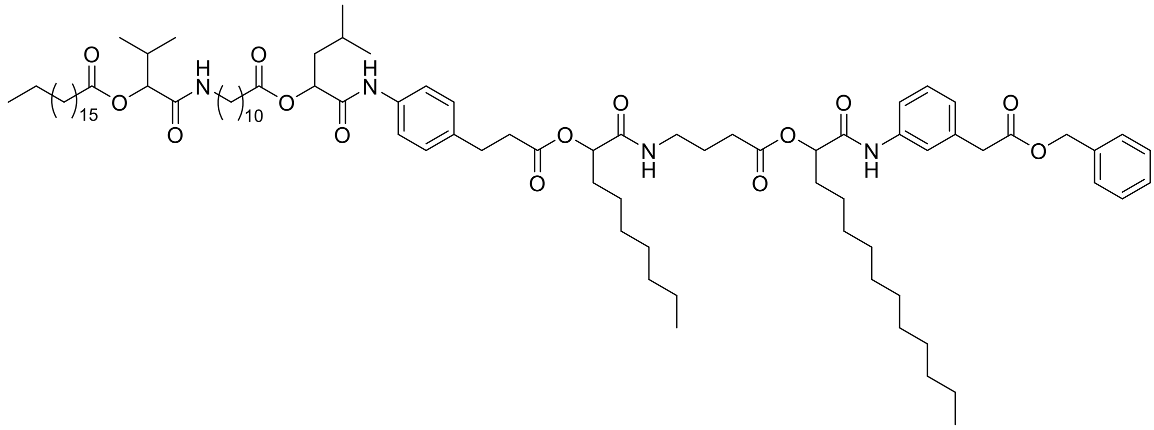

Supplementary Figure 120. Chemical structure of compound ***DS4****.*

Substance **DS3_deprotected** (2.13 g, 2.00 mmol, 1.00 eq.) was dissolved in DCM (4.0 mL, 0.5 M). Subsequently, dodecanal **2g** (666 µL, 553 mg, 3.00 mmol, 1.50 eq.) and monomer **M8** (0.754 g, 3.00 mmol, 1.50 eq.) were added and the reaction mixture was stirred at room temperature for 48 hours. The solvent was removed under reduced pressure and the crude product was purified by column chromatography (cyclohexane / ethyl acetate 12:1 → 1:1, with 5% of triethyl amine) to obtain the desired product **DS4** in a quantitative yield (2.96 g, 2.00 mmol) as a yellow highly viscous oil.

^{1}H NMR (400 MHz, $CDCl_3$) δ / ppm: 8.83 – 6.65 (m, 1H, NH, [1]), 8.03 (s, 1H, NH, [2]), 7.69 – 6.91 (m, 13H, CH aromatic, [3]), 6.24 – 5.93 (m, 2H, NH, [4]), 5.24 (m, 2H, CH, [5]),5.11 – 5.06 (s, 2H, CH_2, [6]), 5.05 – 5.03 (m, 2H, CH, [7]), 3.71 – 3.44 (m, 2H, CH_2, [8]), 3.39 – 2.99 (m, 4H, CH_2, [9]), 3.24 – 2.84 (m, 2H, CH_2, [10]), 2.83 – 2.63 (m, 2H, CH_2, [10]), 2.55 – 2.15 (m, 8H, CH, CH_2, [11]), 2.13 – 1.53 (m, 12H, CH_2, [12]), 1.56 – 1.38 (m, 2H, CH_2, [13]), 1.38 – 1.02 (m, 68H, CH_2, [14]), 1.01 – 0.65 (m, 21H, CH_3, [15]).

^{13}C NMR (101 MHz, $CDCl_3$) δ / ppm: 173.02, 172.71, 172.57, 171.78, 171.71, 171.36, 170.71, 169.44, 168.81, 168.66, 138.00, 137.94, 136.37, 135.82, 134.51, 128.98, 128.92, 128.83, 128.58, 128.26, 128.18, 125.38, 121.64, 120.42, 120.37, 119.49, 77.94, 74.51, 74.26, 72.87, 66.70, 41.25, 40.74, 39.22, 37.91, 35.52, 34.37, 34.29, 32.15, 31.98, 31.89, 31.79, 30.95, 30.58, 30.21, 30.13, 29.75, 29.70, 29.66, 29.61, 29.53, 29.41, 29.33, 29.22, 29.12, 26.87, 25.10, 24.95, 24.61, 23.14, 22.75, 22.66, 21.84, 18.85, 17.01, 14.20, 14.16.

HRMS-ESI-MS $[M+H]^+$ of $[C_{90}H_{144}N_4O_{14}]$: calculated: 1506.0752 found: 1506.0745.

IR (ATR platinum diamond): ν / cm^{-1} = 3306.6, 2923.0, 2853.2, 1738.8, 1659.8, 1610.9, 1536.8, 1493.2, 1444.1, 1415.0, 1371.6, 1240.1, 1149.2889, 1002.3, 828.3, 775.6, 722.5, 695.5, 492.6.

***R*$_f$**: (cyclohexane / ethyl acetate 7: 5) = 0.58.

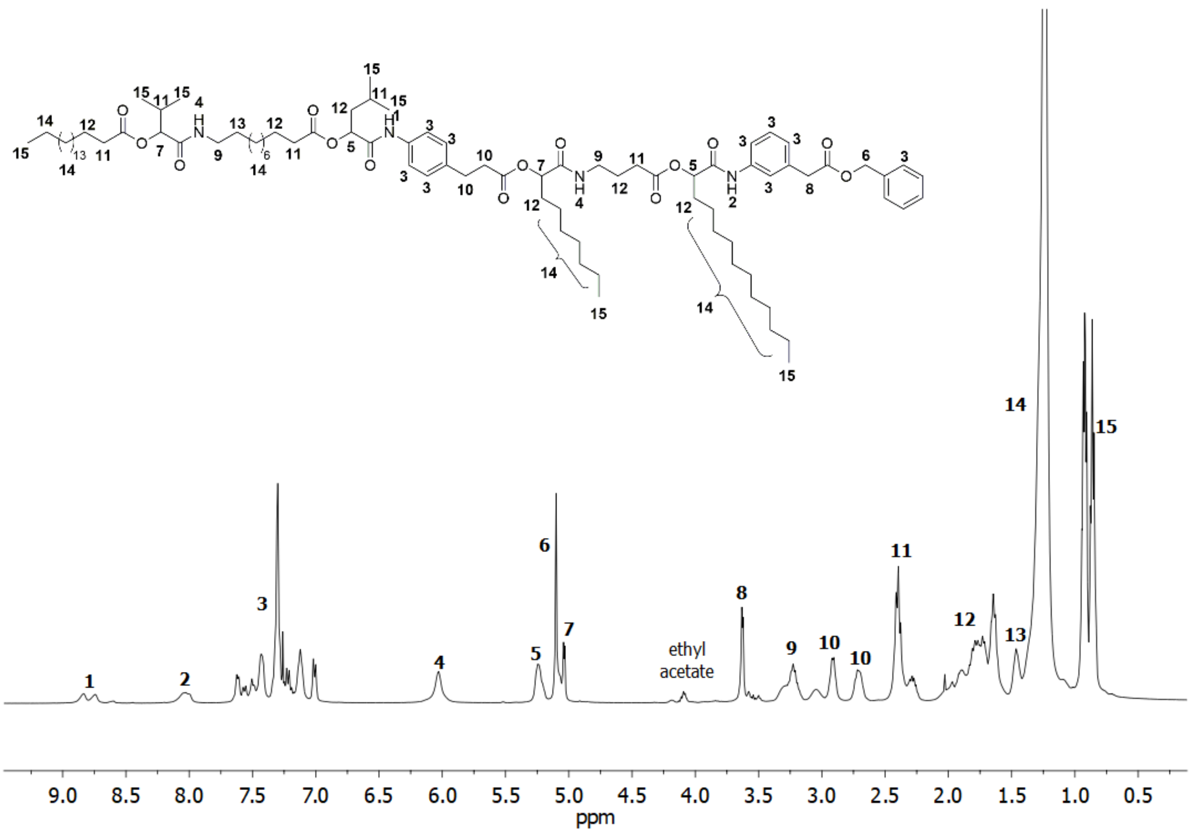

*Supplementary Figure 121. Proton NMR of compound **DS4** measured in $CDCl_3$.*

4th deprotection (DS4_deprotected)

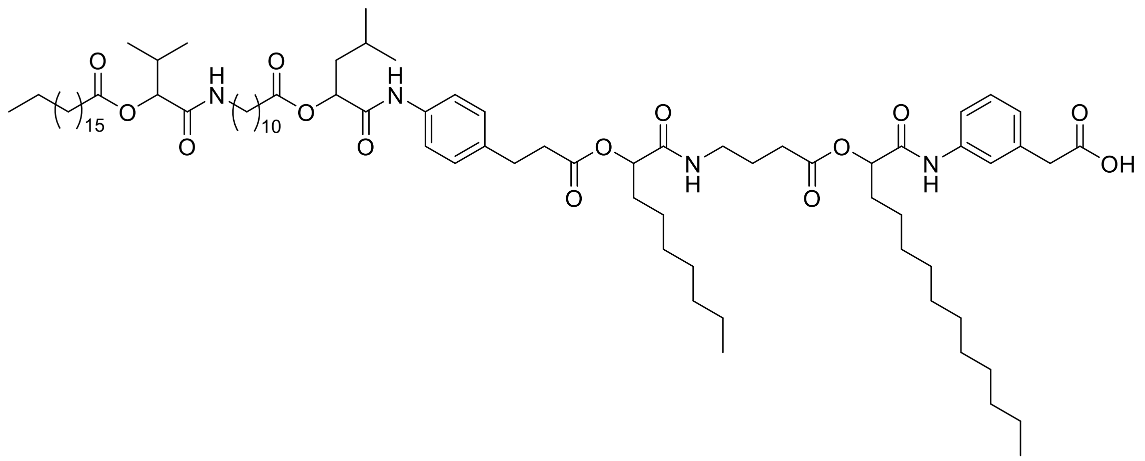

*Supplementary Figure 122. Chemical structure of compound **DS4_deprotected**.*

Substance **DS4** (2.16 g, 1.46 mmol, 1.00 eq.) was dissolved in THF (10.0 mL, 0.15 M). Subsequently, palladium on activated charcoal (216 mg, 10 wt%.) was suspended in the solution. The reaction mixture was purged with hydrogen (3 balloons) and stirred under hydrogen atmosphere overnight. The heterogeneous catalyst was filtered off and the solvent was evaporated under reduced pressure to obtain the desired product **DS4_deprotected** in a yield of 99% (2.01 g, 1.44 mmol) as a highly viscous oil.

^{1}H NMR (500 MHz, $CDCl_3$) δ / ppm: 8.98 – 8.67 (m, 1H, NH, ^{1}), 8.23 – 8.02 (m, 1H, NH, ^{2}), 7.68 – 6.95 (m, 8H, CH aromatic, ^{3}), 6.27 – 5.98 (m, 2H, NH, ^{4}), 5.33 – 5.10 (m, 2H, CH, ^{5}), 5.10 – 4.90 (m, 2H, CH, ^{6}), 3.63 – 3.44 (m, 2H, CH_2, ^{7}), 3.37 – 3.12 (m, 4H, CH_2, ^{8}), 3.12 – 2.59 (m, 4H, CH_2, ^{9}), 2.43 – 2.15 (m, 8H, CH, CH_2, ^{10}), 2.00 – 1.53 (m, 12H, CH_2, ^{11}), 1.51 – 1.32 (m, 2H, CH_2, ^{12}), 1.40 – 1.02 (m, 68H, CH_2, ^{13}), 0.98 – 0.69 (m, 21H, CH_3, ^{14}).

^{13}C NMR (126 MHz, $CDCl_3$) δ / ppm: 174.70, 173.35, 173.30, 172.80, 172.78, 172.74, 172.03, 172.00, 171.36, 170.91, 170.86, 170.81, 170.77, 169.66, 169.02, 169.00, 168.85, 168.82, 137.96, 137.88, 136.49, 136.44, 135.76, 135.72, 134.54, 134.52, 129.03, 129.01, 128.82, 128.80, 125.57, 125.53, 121.59, 121.55, 120.55, 120.51, 119.63, 119.57, 107.70, 106.44, 77.97, 77.96, 74.61, 74.57, 74.28, 72.95, 68.67, 67.74, 67.52, 60.53, 41.01, 40.72, 39.29, 38.02, 37.90, 35.52, 35.47, 34.38, 34.29, 32.14, 32.12, 32.06, 32.00, 31.99, 31.91, 31.81, 31.81, 31.00, 30.92, 30.60, 30.21, 30.14, 29.78, 29.76, 29.73, 29.72, 29.70, 29.69, 29.64, 29.61, 29.58, 29.55, 29.44, 29.35, 29.24, 29.19, 29.13, 27.90, 26.85, 26.06, 26.03, 25.15, 25.11, 24.99, 24.94, 24.86, 24.63, 23.91, 23.89, 23.15, 22.77, 22.68, 22.25, 21.84, 21.13, 18.85, 17.02, 14.27, 14.21, 14.17.

HRMS-ESI-MS $[M+H]^+$ of $[C_{83}H_{138}N_4O_{14}]$: calculated: 1416.0282 found: 1416.0226.

IR (ATR platinum diamond): ν / cm^{-1} =3304.0, 2922.9, 2853.1, 1739.3, 1659.3, 1611.9, 1537.8, 1493.0, 1444.5, 1415.0, 1370.4, 1240.8, 1153.3, 829.1, 775.9, 720.7, 531.2, 444.4.

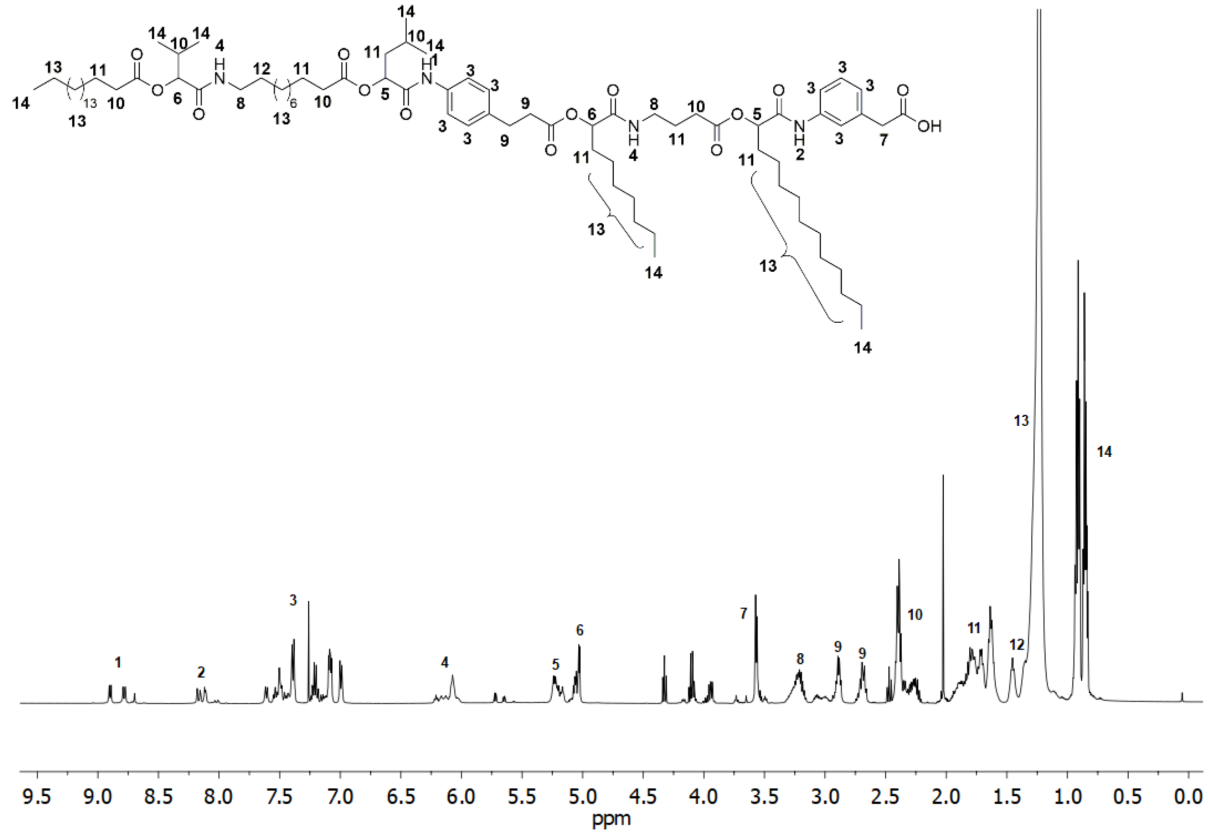

*Supplementary Figure 123. Proton NMR of compound **DS4_deprotected** measured in $CDCl_3$.*

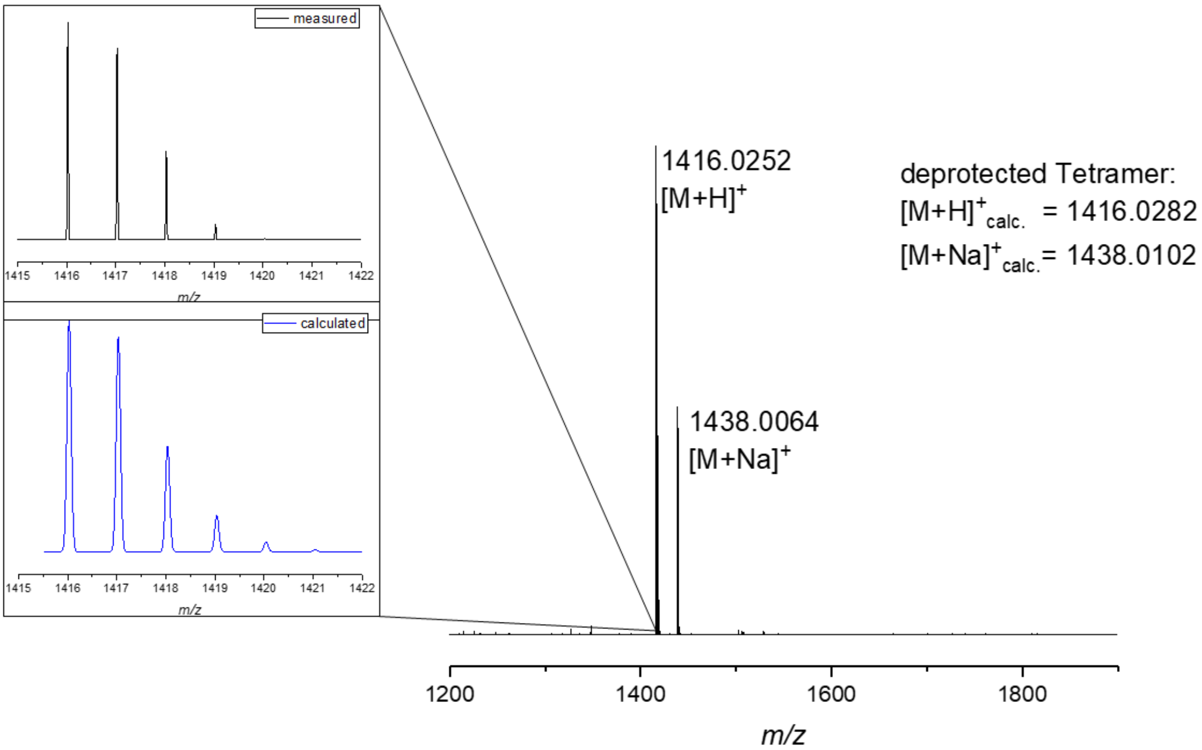

*Supplementary Figure 124. Mass spectrum of the dual sequence-defined deprotected tetramer **DS4_deprotected**: the protonated ion and the singly charged sodium ion was found in the spectrum, further indicating the high purity of the product. The measured isotopic pattern (black) can be compared with the calculated one obtained by mMass (blue), revealing that both spectra are in very good agreement.*

5th Passerini reaction (DS5)

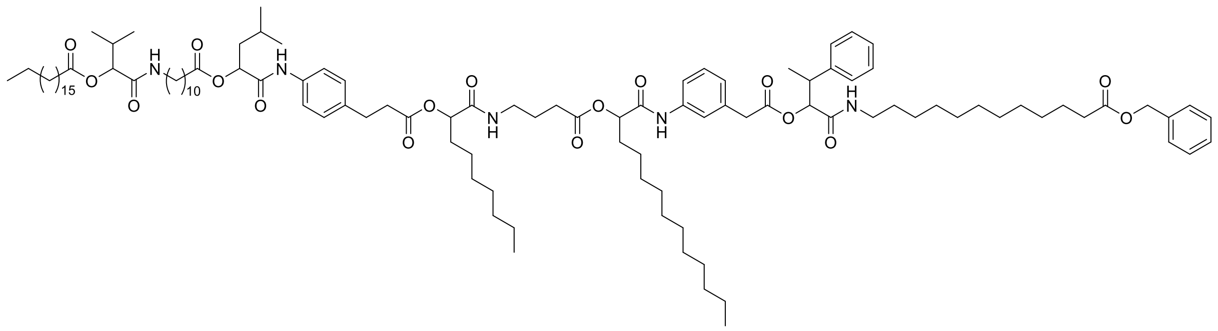

Supplementary Figure 125. Chemical structure of compound ***DS5****.*

Substance **DS4_deprotected** (1.92 g, 1.38 mmol, 1.00 eq.) was dissolved in DCM (4.0 mL, 0.35 M). Subsequently, 2-phenylpropanal **2j** (281 µL, 282 mg, 2.10 mmol, 1.50 eq.) and monomer **M3** (0.662 g, 2.10 mmol, 1.50 eq.) were added and the reaction mixture was stirred at room temperature for 48 hours. The solvent was removed under reduced pressure and the crude product was purified by column chromatography (cyclohexane / ethyl acetate 12:1 → 1:1, with 5% of triethyl amine) to obtain the desired product **DS5** in a yield of 81% (2.05 g, 1.12 mmol) as a white solid.

^{1}H NMR (500 MHz, $CDCl_3$) δ / ppm: 9.14 – 8.88 (m, 1H, NH, [1]), 8.23 – 7.98 (m, 1H, NH, [2]), 7.80 – 6.82 (m, 18H, CH aromatic, [3]), 6.17 – 5.94 (m, 2H, NH, [4]), 5.35 – 5.14 (m, 3H, CH, [5]), 5.12 – 5.05 (m, 2H, CH_2, [6]), 5.03 (m, 2H, CH, [7]), 3.74 – 3.47 (m, 3H, CH, CH_2, [8]), 3.44 – 3.10 (m, 6H, CH_2, [9]), 3.08 – 2.60 (m, 4H, CH_2, [10]), 2.51 – 2.17 (m, 10H, CH, CH_2, [11]), 2.04 – 1.53 (m, 14H, CH_2, [12]), 1.45 (m, 4H, CH_2, [13]), 1.40 – 1.00 (m, 82H, CH_2, [14]), 0.99 – 0.71 (m, 24H, CH_3, [15]).

^{13}C NMR (126 MHz, $CDCl_3$) δ / ppm: 173.79, 172.70, 172.68, 172.51, 171.78, 169.51, 169.45, 169.03, 168.71, 168.68, 141.54, 136.29, 136.15, 135.88, 134.05, 128.79, 128.64, 128.58, 128.39, 128.28, 128.24, 128.21, 128.18, 127.90, 127.84, 126.97, 126.93, 120.39, 119.61, 78.05, 77.95, 77.94, 74.26, 72.86, 66.12, 51.50, 45.87, 41.65, 41.63, 41.59, 41.48, 41.16, 40.73, 39.21, 39.07, 35.51, 35.43, 34.39, 34.35, 34.27, 34.17, 32.14, 32.06, 31.97, 31.95, 31.89, 31.79, 31.78, 30.75, 30.57, 30.23, 30.12, 29.74, 29.72, 29.70, 29.69, 29.66, 29.65, 29.60, 29.56, 29.52, 29.41, 29.32, 29.27, 29.24, 29.21, 29.20, 29.15, 29.12, 26.91, 26.86, 26.77, 26.69, 26.66, 25.32, 25.18, 25.14, 25.09, 25.02, 24.99, 24.94, 24.62, 23.15, 22.74, 22.65, 21.83, 18.84, 17.56, 17.01, 14.74, 14.18, 14.14, 13.22, 8.63.

HRMS-ESI-MS $[M+H]^+$ of $[C_{112}H_{177}N_5O_{17}]$: calculated: 1865.3212 found: 1865.3248.

IR (ATR platinum diamond): ν / cm^{-1} = 3295.8, 2923.7, 2852.7, 1739.7, 1659.5, 1610.7, 1536.5, 1493.9, 1448.5, 1415.5, 1372.2, 1238.2, 1152.2, 1046.7, 827.9, 722.2, 698.7, 536.2.

***R*$_f$**: (cyclohexane / ethyl acetate 5:3) = 0.53.

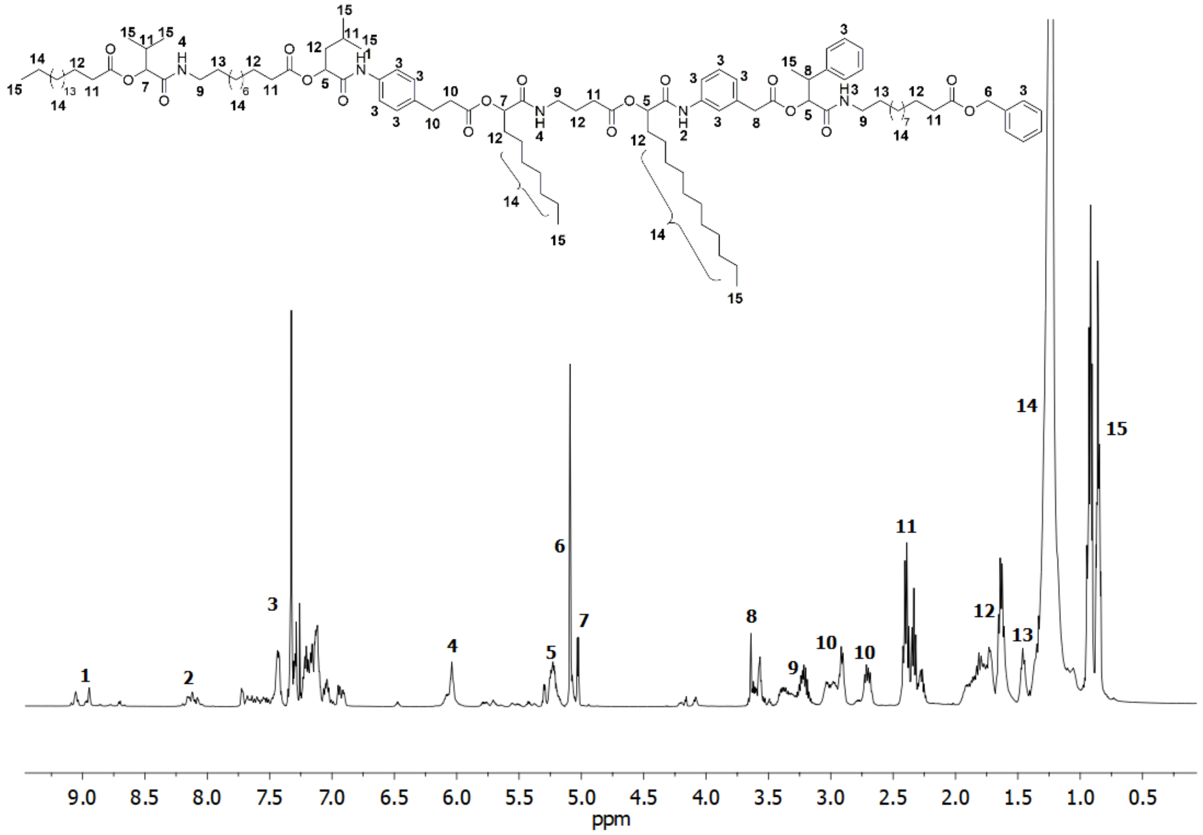

*Supplementary Figure 126. Proton NMR of compound **DS5** measured in $CDCl_3$.*

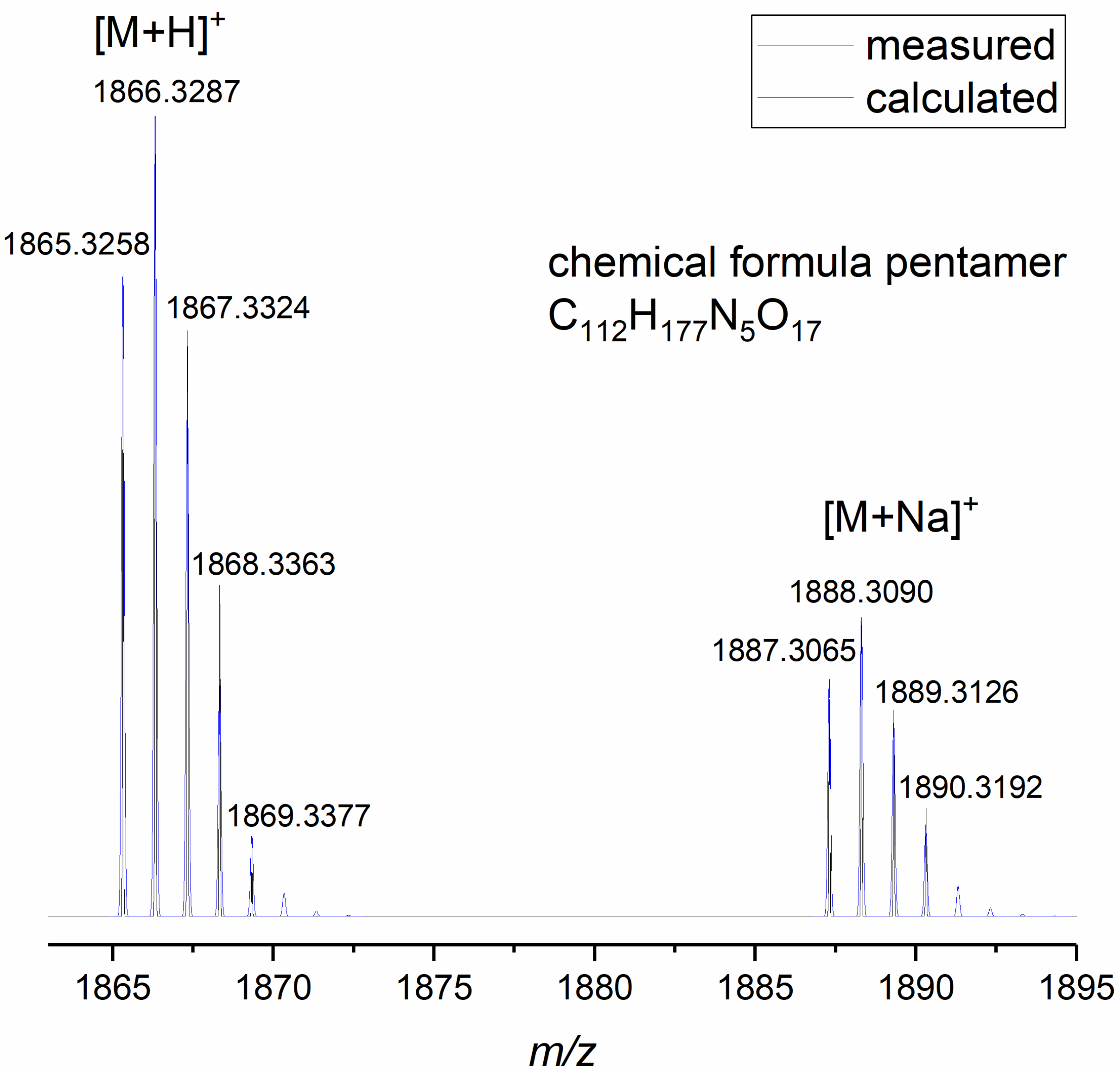

Supplementary Figure 127. HR-ESI-MS measurement of the dual sequence-defined pentamer ***DS5*** *showing isotopic patterns of the sodium and the protonated ion (black). Both isotopic patterns are compared with the calculated ones obtained by mMass (blue), revealing that they are in very good agreement.*

1.3.4.4 Summary of the synthesis of the dual sequence-defined oligomer – SEC and ESI-MS characterisation

A.

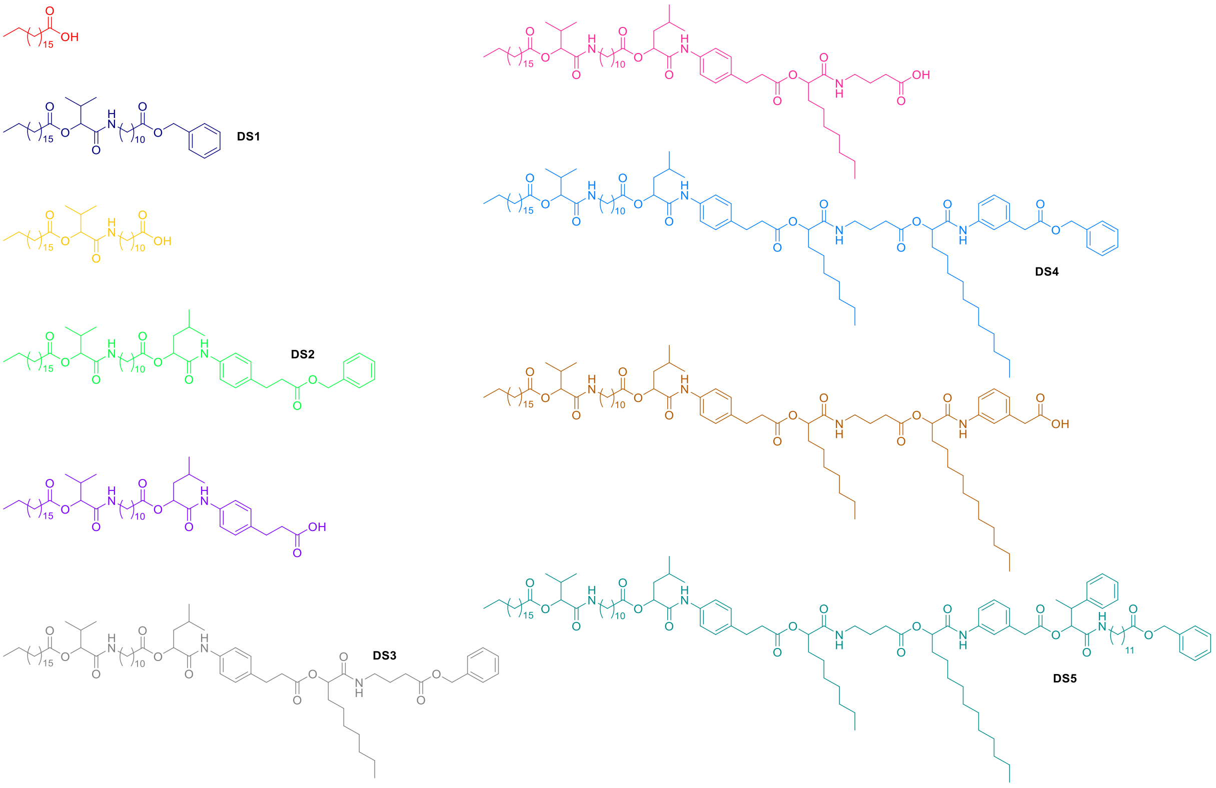

B.

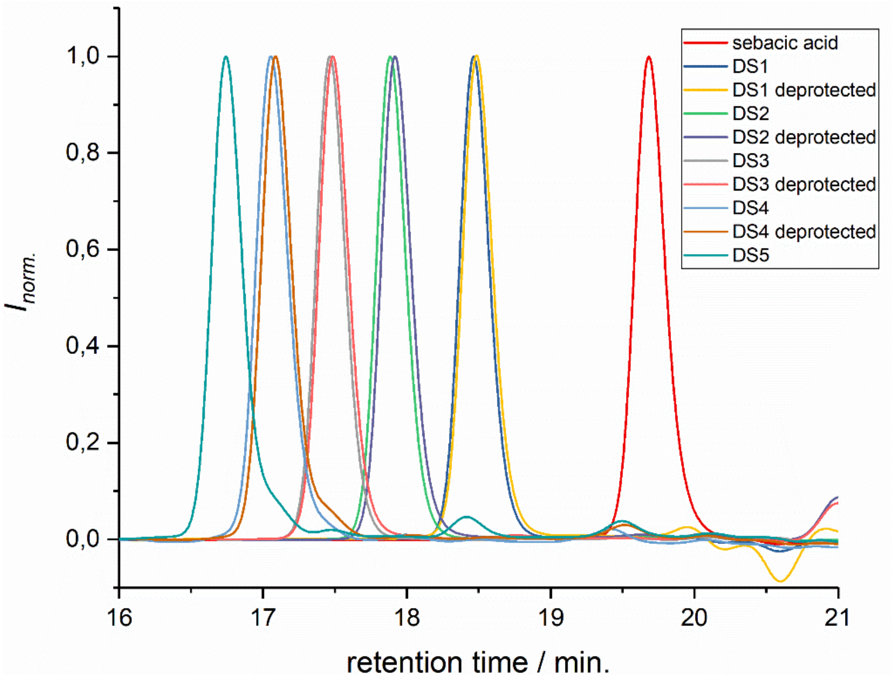

*Supplementary Figure 128 A. Structure of stearic acid **1** and the growing oligomers **DS1** – **DS5** after each P-3CR and deprotection step up to the pentamer stage, where the monomers were inserted step by step in the following order: **M1**, **M7**, **M5**, **M8**, and **M3**. Simultaneously, isobutyraldehyde **2a**, isovaleraldehyde **2d**, octanal **2h**, dodecanal **2g** and 2-phenylpropanal **2j** were used to introduce different side chains. B. SEC results after each P-3CR and deprotection step, verifying the purity of the products. For the assignment of the oligomers, refer to the colour code.*

Supplementary Table 3. Summary of the results obtained in the synthesis of dual sequence-defined macromolecules. The applied monomer and aldehyde component, as well as the yields, applied purification strategy and the calculated and found masses are given.

Product	Applied monomer	Applied aldehyde	Yield [%]	m/z calc.	m/z found
1st P-3CR **DS1**	**M1**	Isobutyraldehyde **2a**	98[a]	658.5405	658.5391
1st deprotection			99[b]	568.4936	568.4922
2nd P-3CR **DS2**	**M7**	Isovaleraldehyde **2d**	65[a]	919.6770	919.6747
2nd deprotection			99[b]	829.6300	829.6279
3rd P-3CR **DS3**	**M5**	Octanal **2h**	47[a]	1160.8448	1160.8435
3rd deprotection			99[b]	1070.7978	1070.7958
4th P-3CR **DS4**	**M8**	Dodecanal **2g**	quant[a]	1506.0752	1506.0745
4th deprotection			99[b]	1416.0282	1416.0226
5th P-3CR **DS5**	**M3**	2-phenyl-propanal **2j**	81[a]	1865.3212	1865.3248
Overall yield	23% over nine reaction steps				

[a] *after purification by column chromatography,* [b] *after filtration.*

1.3.5 Sequential read-out by ESI-MS/MS

1.3.5.1 Different types of fragmentation

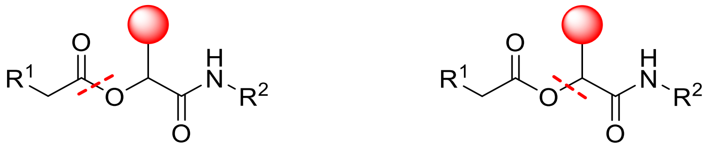

Supplementary Figure 129. Two characteristic fragmentation patterns were found during ESI-MS/MS investigations: Fragmentation next to the carbonyl (left) and fragmentation within the ester group (right). ESI experiments were performed in positive ion mode, thus only positively charged species were observed.

<u>Supplementary Note 1:</u> for each molecule, both types of fragmentation were observed and assigned. Furthermore, fragments with the start and end units as well as the middle fragments (without the start and end units) were observed for the different oligomers. The most prominent fragmentation pattern was the fragmentation next to the carbonyl (Supplementary Figure 129, left). For the dual sequence-defined pentamer, both characteristic fragmentation patterns with the assignment of all peaks are provided as an example. For the other investigated molecules that were used for sequential read-out by ESI-MS/MS, only the most prominent fragmentation (Supplementary Figure 129, left) are depicted (for clarity reasons).

1.3.5.2 Sequencing rules

1.3.5.2.1 Calculation of the mass of the molecule

Supplementary Equation 1

$$[M_{Molecule}+H]^{+}=\left[\left(M_{Start}+\sum_{i=1}^{i=n}M_{Backbone}^{i}+\sum_{i=1}^{i=n}M_{Sidechain}^{i}+M_{End}+y*M(H)\right)+H\right]^{+}$$

x =(n), (n-1), (n-2), (...), 0

y =(n-1)

n = number of repeating units

M_{Start} = M (**1**)

M_{End} = M (C_7H_7)

$M_{Backbone}$ = (M (**M1**) or M (**M2**) or M (**M3**) or M (**M4**) or M (**M5**) or M (**M6**) or M (**M7**) or M (**M8**) or M (**M9**)) – M(C_7H_7)

$M_{Sidechain}$ = M (**2a**) or M (**2b**) or M (**2c**) or M (**2d**) or M (**2e**) or M (**2f**) or M (**2g**) or M (**2h**) or M (**2i**) or M (**2j**) or M (**2k**)

$M_{Backbone}$ is calculated with the mass of the monomer which incorporates the protected acid (benzyl ester); however, in the iterative cycle, the benzyl ester is deprotected and further converted as the free acid compound. In order to take that into consideration in the formula, y is introduced as additional summand.

1.3.5.2.2 Fragmentation

1.3.5.2.2.1 Fragmentation next to the carbonyl

From left to the right:

Supplementary Equation 2

$$[M+H]^{+}=\left[\left((M_{start}-M\,(OH))+\sum_{i=1}^{i=x}M_{Backbone}^{i}+\sum_{i=1}^{i=x}M_{Sidechain}^{i}+(x-1)*M(H)\right)+H\right]^{+}$$

From right to the left:

Supplementary Equation 3

$$[M+H]^{+}=\left[\left(M_{End}+\sum_{i=1}^{i=x}M_{Backbone}^{i}+\sum_{i=1}^{i=x}M_{Sidechain}^{i}+M(OH)+x*M(H)\right)+H\right]^{+}$$

1.3.5.2.2.2 Fragmentation next to the ester

From left to the right:

Supplementary Equation 4

$$[M+H]^{+} = \left[\left((M_{start} - M\,(H)) + \sum_{i=1}^{i=x} M^{i}_{Backbone} + \sum_{i=0}^{i=x} M^{i}_{Sidechain} + (x+1) * M(H)\right) + H\right]^{+}$$

From right to the left:

Supplementary Equation 5

$$[M+H]^{+} = \left[\left(M_{End} + \sum_{i=1}^{i=x} M^{i}_{Backbone} + \sum_{i=0}^{i=x} M^{i}_{Sidechain} + (x-1) * M(H)\right) + H\right]^{+}$$

1.3.5.2.3 Calculation example for the dual sequence-defined pentamer DS5

The first step was to calculate the mass of the molecule and to find the respective mass peak in the mass spectrum.

Supplementary Equation 6

$$M_{Molecule} = M_{Start} + \sum_{i=1}^{i=x} M^{i}_{Backbone} + \sum_{i=1}^{i=x} M^{i}_{Sidechain} + M_{End} + y * M(H)$$

For **DS5**:

M_{DS5}= (284.27153 + (301.20418 - 91.05478) + (265.11028 - 91.05478) + (203.09463 - 91.05478) + (251.09463 - 91.05478) + (315.21983 – 91.05478) + 72.05751 + 86.07316 + 128.12012 + 184.18272 +134.07316 + 91.05478 + 4*1.00783) Da

M_{DS5}= 1864.31395 Da

Fragmentation of **DS5**

Fragmentation next to the carbonyl:

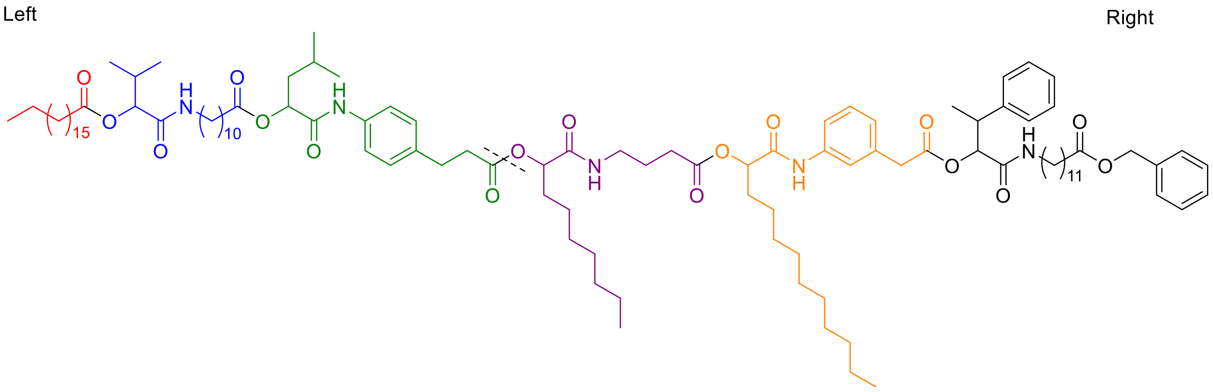

Supplementary Figure 130. Structure of the dual sequence-defined pentamer DS5 is depicted. One fragmentation next to the carbonyl is shown exemplarily and is marked with a dashed line.

Fragmentation pattern calculation:

From left to the right:

M_{Left} = ((284.27153 − 17.00274) + (301.20418 − 91.05478) + (265.11028 − 91.05478) + 72.05751 + 86.07316 + ((2-1) * 1.00783)) Da

M_{Left} = 810.61219 Da

From right to the left:

M_{Right} = (91.05478 + (315.21983 − 91.05478) + (251.09463 − 91.05478) + (203.09463 − 91.05478) + 134.07316 + 184.18272 + 128.12012 + 17.00274 + (3 * 1.00783)) Da

M_{Right} = 1053.70176 Da

1.3.5.3 Side chain defined pentamer S5

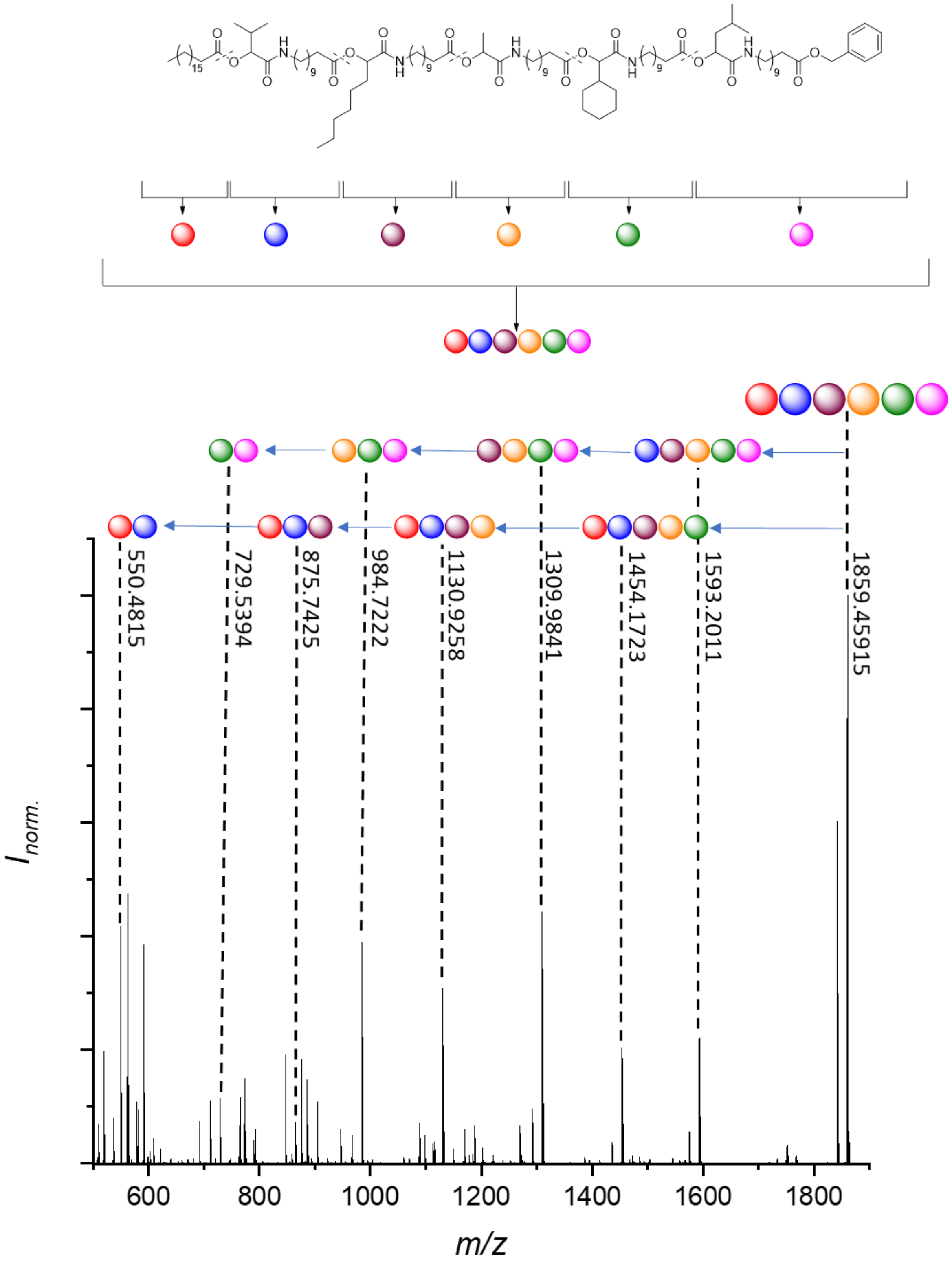

*Supplementary Figure 131. Structure and ESI-MS/MS fragmentation of the side chain-defined pentamer **S5**. The assigned peaks belong to the most prominent fragmentation pattern (in this case: fragmentation next to the carbonyl) from both ends of the molecule. Other intense peaks belong to the other prominent fragmentation pattern and to the middle fragments and can be assigned analogously (for the sake of clarity not shown in this graph).*

1.3.5.4 Side chain defined decamer S10

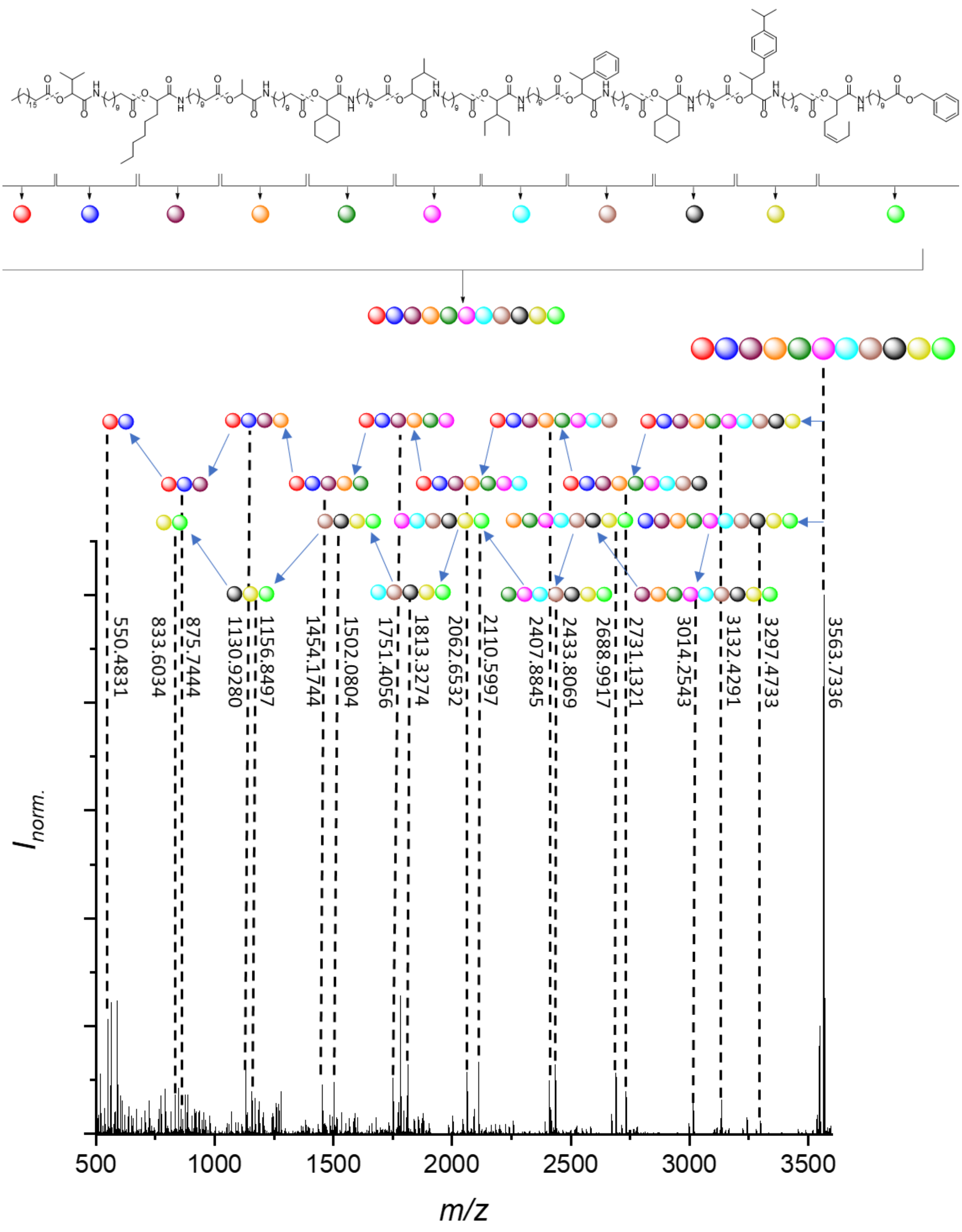

*Supplementary Figure 132 Structure and ESI-MS/MS fragmentation of the side chain-defined decamer **S10**. The assigned peaks belong to the most prominent fragmentation pattern (in this case: fragmentation next to the carbonyl) from both ends of the molecule. Other intense peaks belong to the other prominent fragmentation pattern and to the middle fragments and can be assigned analogously (for the sake of clarity not shown in this graph).*

1.3.5.5 Backbone defined pentamer B5

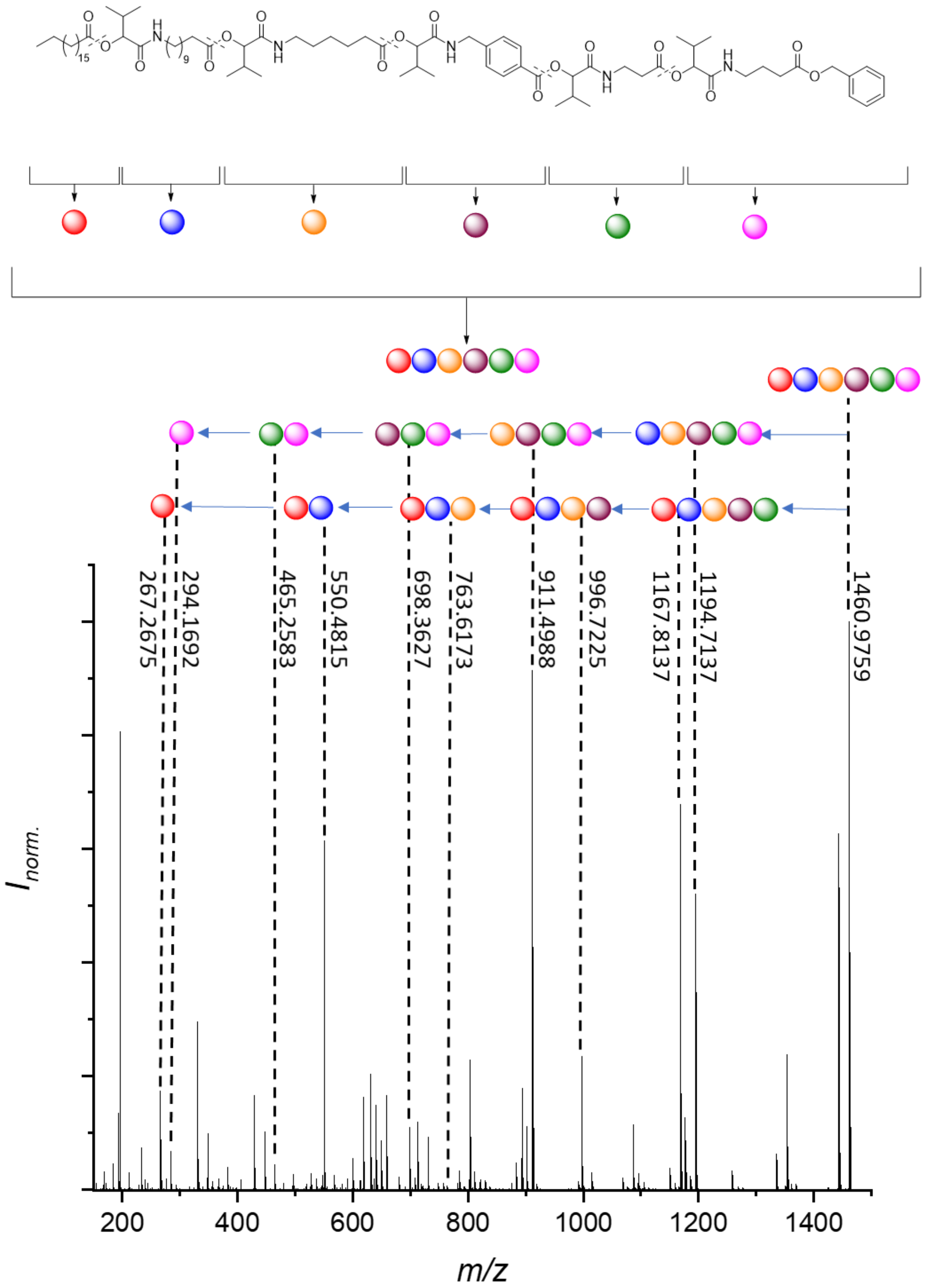

*Supplementary Figure 133. Structure and ESI-MS/MS fragmentation of the backbone-defined pentamer **B5**. The assigned peaks belong to the most prominent fragmentation pattern (in this case: fragmentation next to the carbonyl) from both ends of the molecule. Other intense peaks belong to the other prominent fragmentation pattern and to the middle fragments and can be assigned analogously (for the sake of clarity not shown in this graph).*

1.3.5.6 Backbone defined heptamer B7

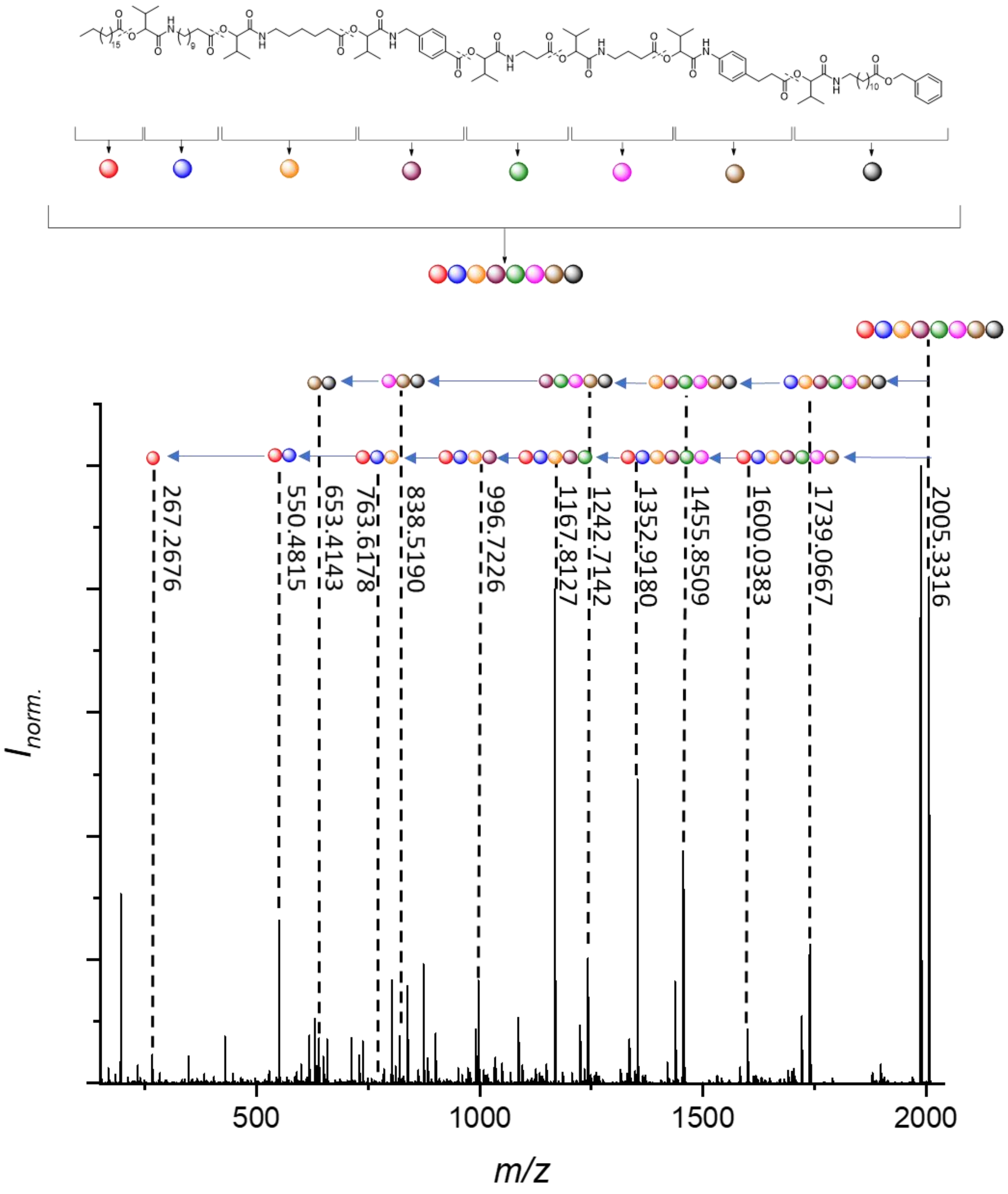

*Supplementary Figure 134. Structure and tandem ESI-MS/MS fragmentation of the backbone-defined heptamer **B7**. The assigned peaks belong to the most prominent fragmentation pattern (in this case: fragmentation next to the carbonyl) from both ends of the molecule. Other intense peaks belong to the other prominent fragmentation pattern and to the middle fragments and can be assigned analogously (for the sake of clarity not shown in this graph).*

1.3.5.7 Dual sequence-defined pentamer DS5

1.3.5.7.1 Fragments with start and end block

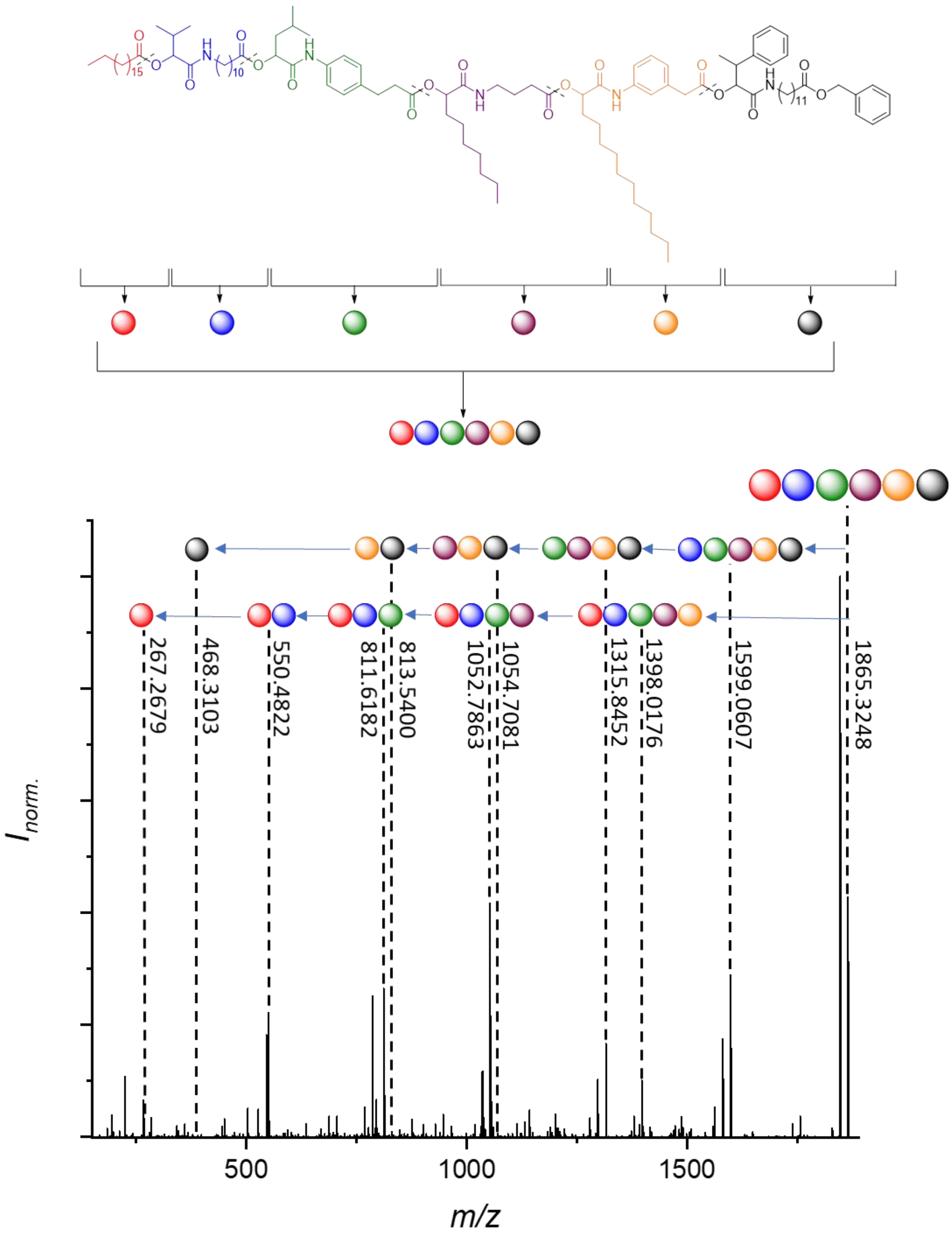

Supplementary Figure 135. Structure and ESI-MS/MS fragmentation of the dual sequence-defined pentamer ***DS5****. The assigned peaks belong to the most prominent fragmentation pattern (in this case: fragmentation next to the carbonyl) from both ends of the molecule. Other intense peaks belong to the other prominent fragmentation pattern and to the middle fragments and can be assigned analogously (for the sake of clarity not shown in this graph).*

1.3.5.7.2 Middle fragments (without start and end block)

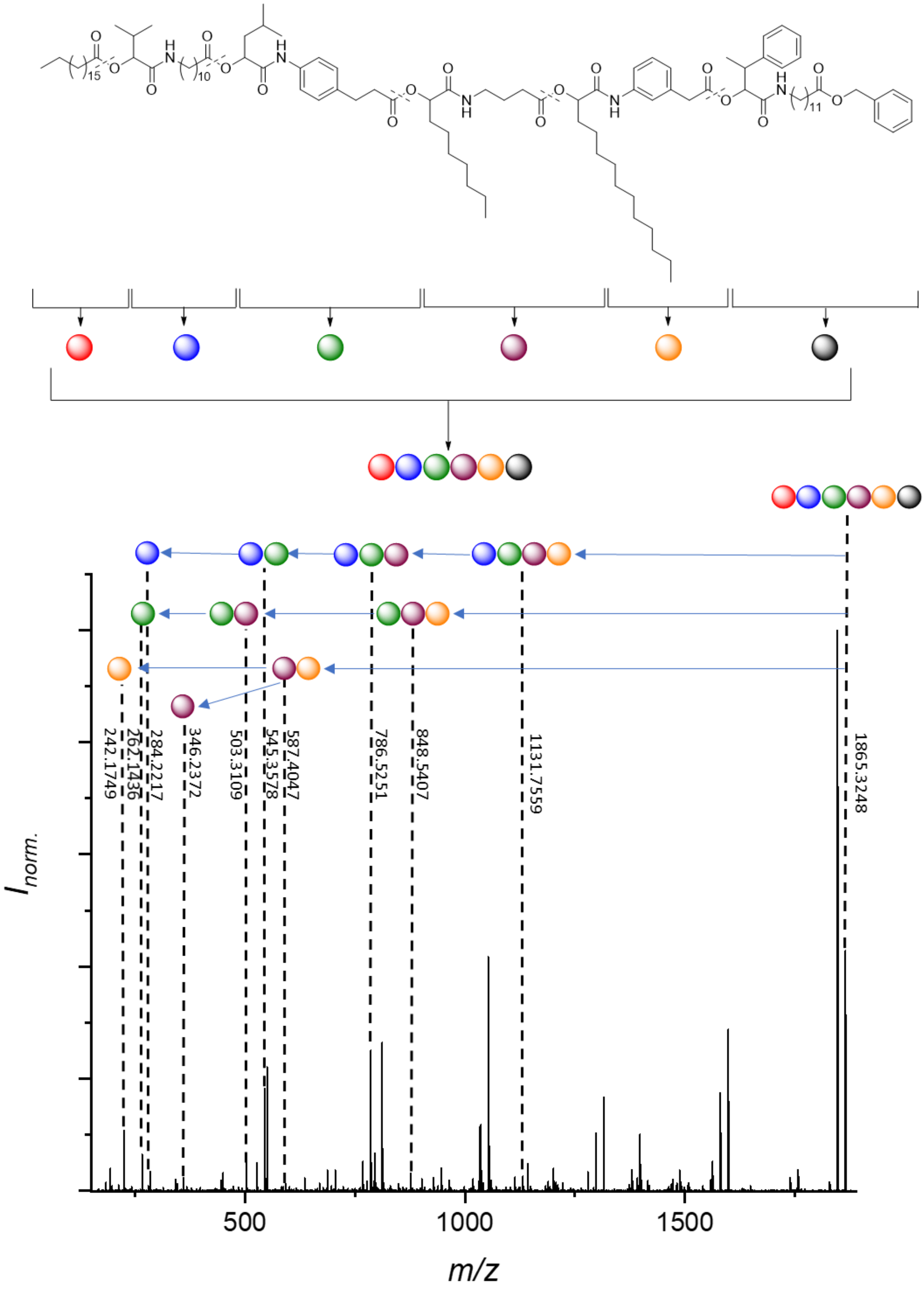

*Supplementary Figure 136. Structure and ESI-MS/MS fragmentation of the dual sequence-defined pentamer **DS5**. The assigned peaks belong to the middle parts of the most prominent fragmentation pattern (in this case: fragmentation next to the carbonyl). Other intense peaks belong to the other prominent fragmentation pattern and to the fragments with start and end block and can be assigned analogously (for the sake of clarity not shown in this graph). For the assignment of the other peaks, please see Figures S12 and S14.*

1.3.5.7.3 Fragmentation next to the ester

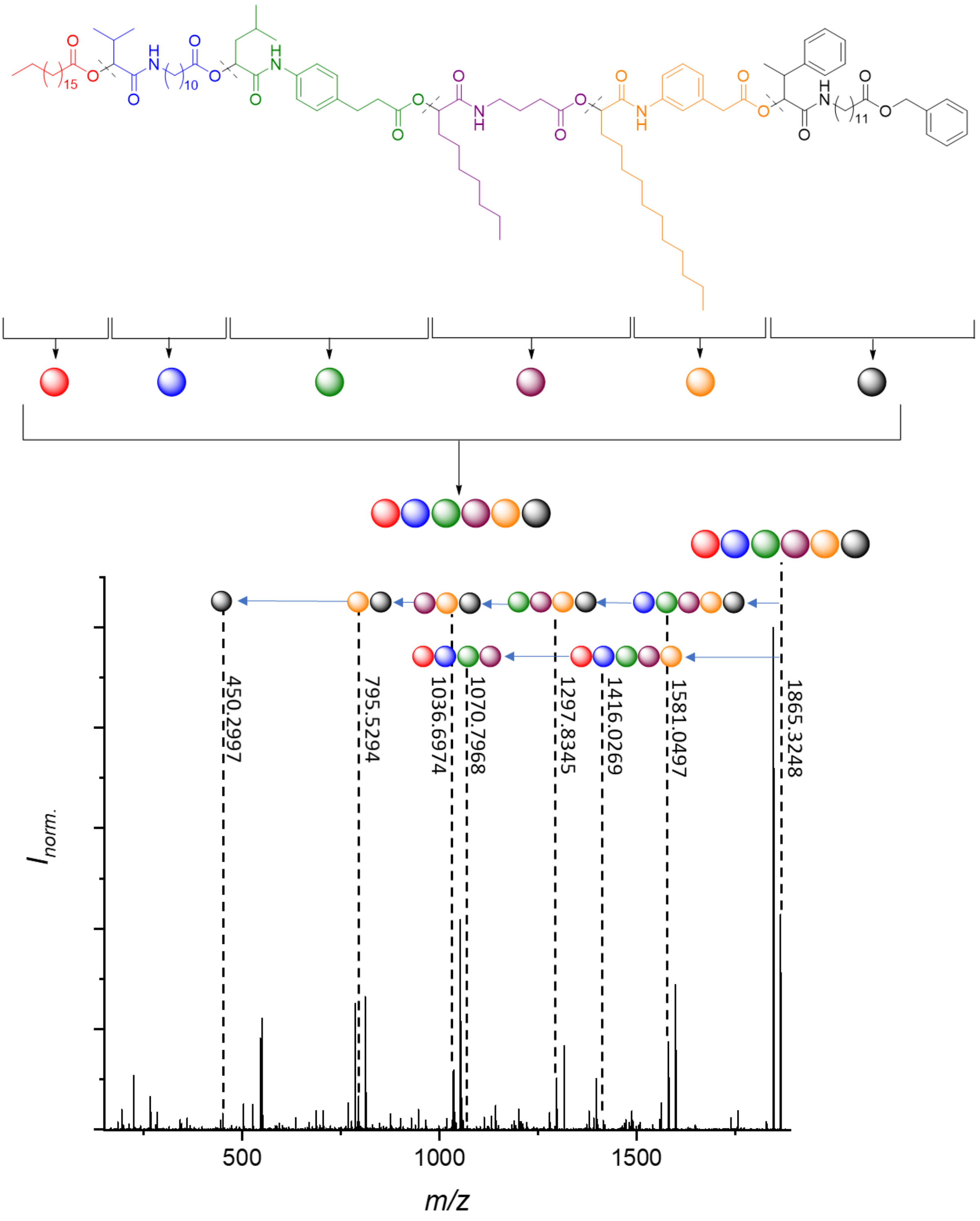

Supplementary Figure 137. Structure and ESI-MS/MS fragmentation of the dual sequence-defined pentamer ***DS5****. The assigned peaks belong to the fragmentation next to the ester. Other more intense peaks belong to the fragmentation next to the carbonyl and are assigned analogously (for the sake of clarity not shown in this graph). For the assignment of the other peaks, please see Figures S12 and S13.*

1.4 Supplementary References

[1] S. C. Solleder, D. Zengel, K. S. Wetzel, M. A. Meier, *Angew. Chem. Int. Ed.* **2016**, *55*, 1204-1207.
